# Synthesis of *N*‑Substituted Hydroxamic Acids through a Pentafluoropyridine-Mediated Procedure

**DOI:** 10.1021/acs.joc.6c00360

**Published:** 2026-05-05

**Authors:** Daniel E. Bonn, Toby J. Blundell, Matthew Jenner, William D. G. Brittain

**Affiliations:** a Department of Chemistry, 3057 Durham University, South Road, Durham DH1 3LE, United Kingdom; b Department of Chemistry, 2707 University of Warwick, Coventry CV4 7AL, United Kingdom

## Abstract

Given the increased interest in hydroxamic acid (HA) derivatives across industry and drug candidates, the need for robust and easy-to-conduct synthetic procedures is required. In this report, we demonstrate the use of *in situ* carboxylic acid (CA) activation to generate benzoyl-protected *N*-substituted HAs in excellent yields. Additionally, we present a one-pot, three-step procedure for the generation of unprotected analogues directly from the parent CAs, requiring no additional purification or intermediate isolation.

## Graphical abstract

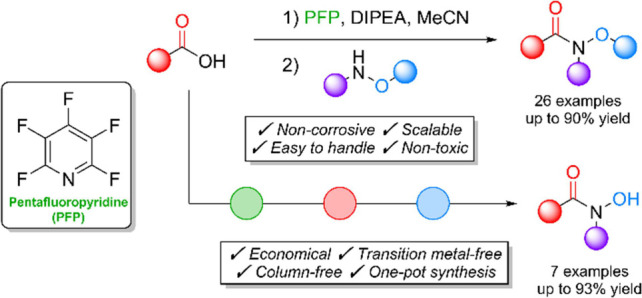

## Introduction

Hydroxamic acids (HAs) were first isolated during the synthesis of oxalohydroxamic acid,^1^ with their unique properties having since been well studied contributing as essential components to a range of natural products and synthetically derived drug compounds.^2^ HAs are weak acids (p*K*_a_∼9)^3^ and due to their structure, *cis*/*trans*-isomerism can be observed relative to the C–N bond, where the less stable *cis*-geometry is adopted when chelated to metals.^4^ This conformational flexibility gives rise to their strong affinity for metals, particularly ferric iron, facilitated through bidentate binding through the oxygen atoms. This interaction is prevalent in nature, exemplified by siderophores, where typically three hydroxamate groups are used to form a polyhedral complex.^5^ This subset of microbial iron chelators possess *N*-substituted HA functionality as key binding motifs, enabling thermodynamic binding constants up to 10^30^ with ferric iron for trishydroxamates.^5^ Inspired by their strong affinity for transition metal ions, many applications have been developed exploiting HAs.^6^ A variety of drug compounds including vorinostat and batimastat contain HA functionality with the number of reported bioactive compounds consistently seeing significant increase.^7^ Similarly, the FDA-approved natural product desferrioximine B (Desferal), has been used to treat iron overload (Scheme 1A).^8^

**Scheme 1:**
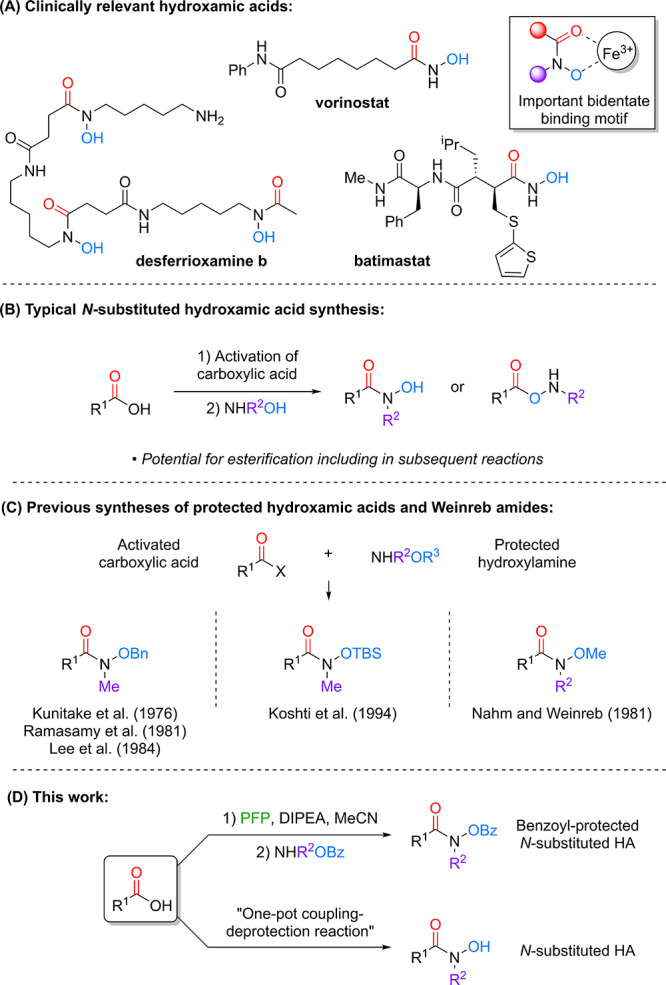
Synthesis of *N*-Substituted HAs

While the utility of HAs is expanding, the ability to install the moiety synthetically remains a challenge with rapid, modular and flexible methods notably lacking. Traditional approaches often require the use of preformed activated carboxylic acid (CA) equivalents, employ time-consuming purification procedures or are incompatible with downstream transformations.^9^ Additionally, when attempting to access *N*-substituted HAs from hydroxylamines, the nature of the *N*-substituent can significantly influence whether acylation or esterification is favored, leading to complex product mixtures (Scheme 1B).^10^ Additionally, exposure of HAs to late-stage modifications can lead to unwanted side reactions with chelation of metals that may be employed as reactants or catalysts making purification and product isolation tedious and difficult to achieve. As such, the use of protected-hydroxylamines has been explored including benzyl (Bn) and *tert*-butyldimethylsilyl (TBS) ethers (Scheme 1C). The earliest examples of Bn protection was in the condensations of CAs with *N*-methyl-*O*-benzylhydroxylamine, facilitated by an acyl chloride, DCC or EDC.^11^ Similarly, TBS-protected hydroxylamines were first applied with acyl chlorides, following initial work using the equivalent unsubstituted hydroxylamine.^12^ However, these approaches require harsh deprotection conditions such as hydrogenation, trifluoroacetic acid or tetrabutylammonium fluoride.^12,13^ Although benzoyl (Bz) protection has been reported, only a handful of select examples use CAs as parent compounds.^14^ Bz groups offer potential for easy removal under basic conditions, and we also believed they would allow flexibility in late-stage functionalization and broad compatibility with CA activation. To this end, we were also convinced that the use of acyl fluorides may address the issues with current approaches, building on our standing interest in fluorinated activated intermediates.^15^ Recently, we have developed the use of pentafluoropyridine (PFP) to generate acyl fluorides under mild conditions, and thus we hypothesized that this approach may facilitate the preparation of HAs (Scheme 1D).^16^ We therefore envisaged the development of a reliable, modular and flexible strategy for the synthesis of *N*-substituted HAs utilizing an activation, coupling, deprotection sequence.

## Results and Discussion

To begin our study, we considered the reaction of commercially available *N*-methylhydroxylamine hydrochloride which could be readily modified with a Bz PG.^17^ We attempted to optimize a one-pot acyl fluoride formation, nucleophilic substitution reaction starting from CA **1a** and Bz-protected hydroxylamine **3a**. Encouragingly, on the addition of **3a** following generation of the acyl fluoride for 4 h in the presence of *N*,*N*-diisopropylethylamine (DIPEA) in MeCN, the desired protected-HA **4a** was obtained in 64% yield (Table 1, Entry 3). An initial base screen revealed improved yields when nitrogen-containing bases were employed in comparison to K_2_CO_3_, with DIPEA giving the best isolated yield (Table 1, Entry 3). The low yield when using K_2_CO_3_ (Table 1, Entry 4) was attributed to the poor solubility of the base in MeCN. Subsequently, the solvent choice was investigated (Table 1, Entries 5–9), revealing MeCN and dimethylformamide (DMF) to be the superior choices (Table 1, Entries 3 and 8). We opted for MeCN given its lower boiling point and general easier handling compared to DMF. In our preliminary optimization, baseline conditions of 1.5 equiv of **1a**, PFP and base were used to drive the formation of acyl fluoride relative to **3a**. Thus, we investigated reducing the equivalents of the reagents for acyl fluoride formation to a slight excess of 1.1 (Table 1, Entry 10). The yield was significantly lower in comparison and only when the activation window was increased to 23 h did the yield return to a comparable value of 60% (Table 1, Entry 11). As no improvements above 64% were seen when combining the ideal base, reactant equiv. and temperature (temp.), the reaction was repeated at a slightly larger scale (1 mmol of **3a** against 0.3 mmol) to minimize any handling or isolation issues. The isolated yield at this scale improved dramatically to 89% (Table 1, Entry 13).

**Table 1:**
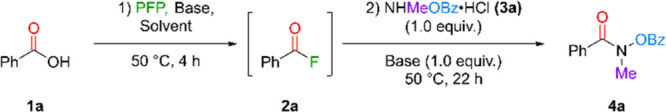
Optimization^a^

Entry	**1a**/PFP/base (equiv)	Base	Solvent	Yield (%)^b^
1	1.5	K_2_CO_3_	MeCN	19
2	1.5	TEA	MeCN	56
3	1.5	DIPEA	MeCN	64
4	1.5	Pyridine	MeCN	21
5^c^	1.5	DIPEA	DCM	18
6	1.5	DIPEA	THF	28
7	1.5	DIPEA	1,4-dioxane	21
8	1.5	DIPEA	DMF	65
9	1.5	DIPEA	Toluene	14
10	1.1	DIPEA	MeCN	28
11^d^	1.1	DIPEA	MeCN	60
12^e^	1.5	DIPEA	MeCN	64
**13** ^f^	**1.5**	**DIPEA**	**MeCN**	**89**
14^g^	1.5	DIPEA	MeCN	85

a Unless otherwise specified, reaction conditions were as follows: **3a** (0.3 mmol, 1.0 equiv) and solvent (1 mL).

b Isolated yield following workup and flash column chromatography.

c Performed at rt.

d Step 1 extended to 23 h.

e **3a** added without additional solvent/base.

f Repeat of Entry 3 with **3a** (1 mmol).

g No workup.

With optimized conditions in hand, we next looked to explore the scope for the synthesis of protected-HAs (Scheme 2A). However, to ensure that a protecting group (PG) was required, the reaction conditions were applied to **1a** and *N*-methylhydroxylamine hydrochloride. Isolation of both **4a** (30%) and **5a** (20%) in poor yields revealed that uncontrolled amidation and esterification were observed and hence a PG strategy was essential for selective product formation. Several benzoic acids were then evaluated, containing electron-donating (**4f** and **4h**) and electron-withdrawing (**4b–e**) substituents. As expected, the more electron poor CAs resulted in lower isolated yield presumably due to slower formation of the activated intermediates.^16b^ Hence, a longer activation time was investigated and an improvement in yield of between 7–24% (**4c** and **4e**) was observed when extended to 24 h. Substrates starting from heteroaromatic (**4k**), primary alkyl (**4g**) and secondary alkyl (**4i**) CAs were compatible with the approach. Coupling to medicinally relevant CAs, Ibuprofen and Naproxen, returned good yields of >77% for the analogues (**4l** and **4m**). Overall, variation of the CA demonstrated broad tolerance with fair to excellent yields of Bz-protected HAs. Finally, we explored potential deprotection conditions to unveil the free hydroxyl moiety. It was found that treating compound **4h** with either NH_3_ in MeOH or aqueous NaOH smoothly led to **5c** in excellent yields (Scheme 2B).

**Scheme 2:**
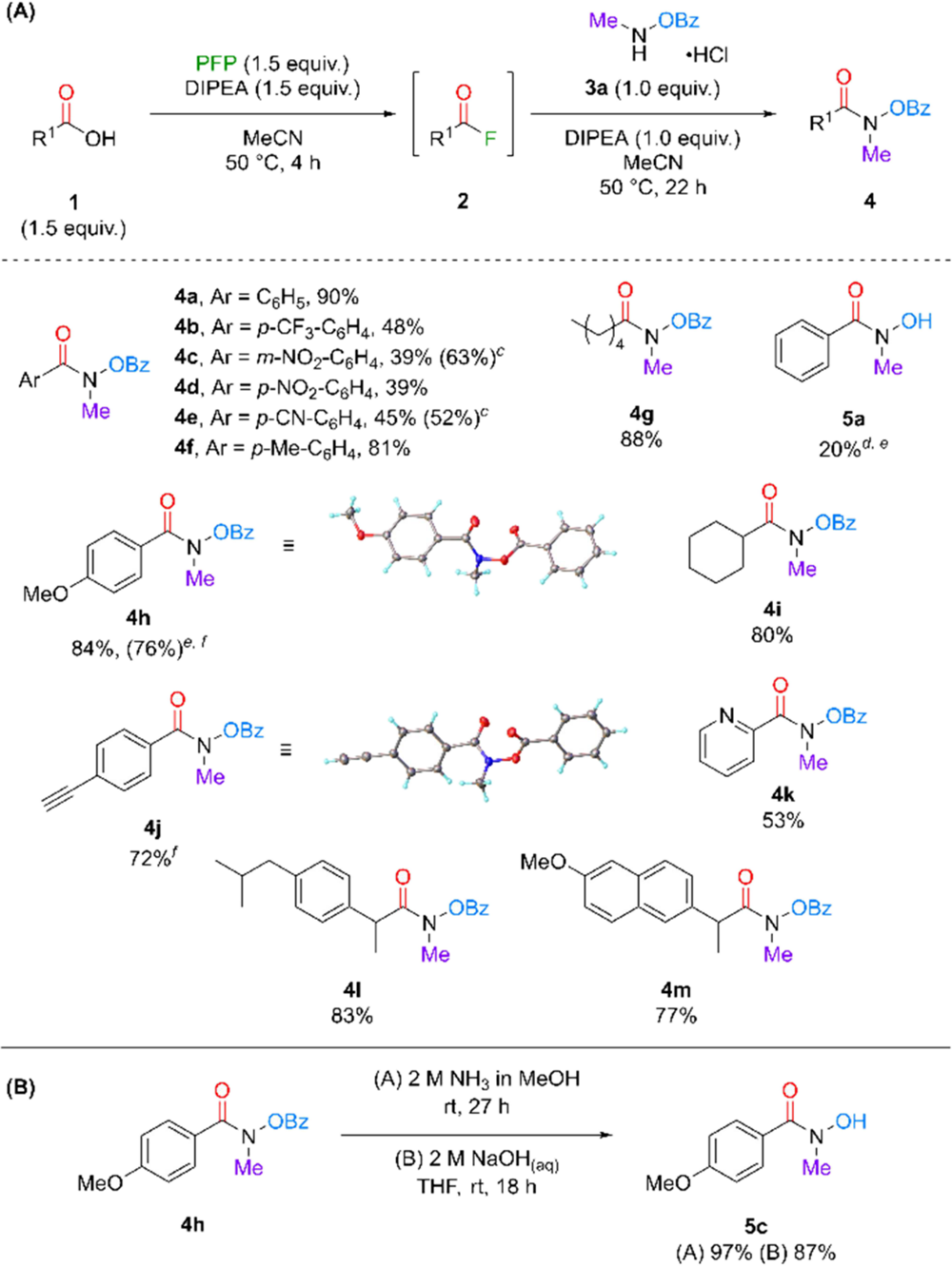
(A) Substrate Scope of CAs in Protected HA Formation Using PFP as a Coupling Reagent; (B) Deprotection Conditions to Form Free HAs

a Reaction conditions: Table 1, entry 13.
b Isolated yield.
c Step 1 extended to 24 h.
d Reaction using unprotected hydroxylamine; 30% yield of **4a** also isolated.
e No workup.
f Gram-scale: **4h** 1.25 g, 4.37 mmol; **4j** 1.90 g, 6.82 mmol.

We then investigated varying the substituent on the hydroxylamine nitrogen to incorporate various groups (Scheme 3). Pleasingly, it was found that these were compatible with the methodology (**4n**–**p** and **4r**). As expected, more electron-poor CAs in combination with more sterically demanding hydroxylamines returned diminished yield, such as with an *^i^* Pr substituent in **4r** being particularly poor (7%). Extending the time of the activation period resulted in an increase in yield despite only rising to 18%. From these results we attempted to push the activation conditions further by heating the solution during activation to 120 °C in a sealed Ace pressure tube, which presented an appreciable increase in yield (56%). It was also observed that the *N*-Bn substituted hydroxylamine hydrochloride underwent slow decomposition when in solution, potentially suggesting a reason for slightly diminished yields in comparison to the *N*-Me analogues (**4e** and **4h**). Unfortunately, even with an electron-rich CA known to give rapid conversion to the reactive acyl fluoride, substitution with the *N*-*^t^* Bu hydroxylamines was not achieved (**4q**), presumably due to the overwhelming steric bulk of the nucleophile. Furthermore, we decided that changing OBz to OMe would give a straightforward route to access Weinreb amides. This class of amide has been widely studied due to its ability to partake in a wide variety of subsequent transformations.^18^ The developed methodology was successful, giving a range of Weinreb amides (**4s**–**4aa**) in good to excellent yield. This approach therefore offers a modular approach to access HAs and other hydroxylated amides through selection of the substituent on the hydroxyl moiety of the hydroxylamine starting material.

**Scheme 3:**
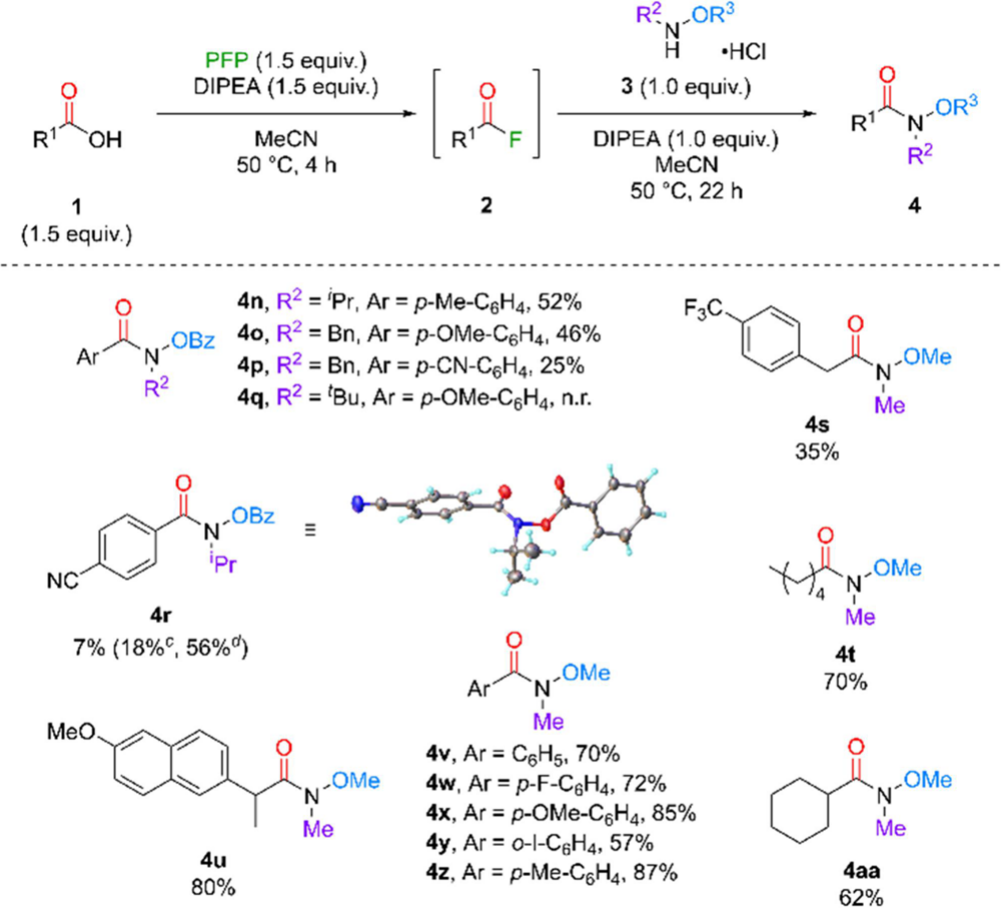
Substrate Scope of Hydroxylamines in Protected-HA Synthesis

a Reaction conditions: Table 1, entry 13.
b Isolated yield.
c Step 1 extended to 24 h.
d Temp. of step 1 increased to 120 °C.

Given our success at isolating protected *N*-Me substituted HAs in combination with what we believed should be inoffensive byproducts from the reaction, we wanted to further investigate a sequential one-pot coupling/deprotection strategy to avoid the need for intermediate purification (Scheme 4A). Therefore, following completion of the optimized coupling procedure from **1a**, a solution of aqueous base was added directly to the reaction mixture and left to stir at room temperature (rt) overnight. To our delight, following an acid–base extraction, HA **5a** was isolated in 76% yield, without the need for any further purification. The lack of chromatography increased the speed and utility of the reaction especially where purification techniques may cause further complications. This factor is exacerbated in the case of HAs where exposure to any trace metals (e.g., in silica/sand) can lead to unwanted chelation and decreased yields. Hence, the same procedure was applied to synthesize a range of compounds (**5a**–**5g**) giving fully deprotected *N*-substituted HAs in up to 93% yield. Based on our understanding developed in previous work,^16a^ we were able to propose a mechanism for the sequential coupling-deprotection reaction (Scheme 5). We believe that the reaction proceeds through attack of a carboxylate towards PFP (II), forming a Meisenheimer intermediate (III), which rapidly collapses to generate a fluoride ion which attacks the activated ester intermediate (IV). From this point, standard addition–elimination chemistry occurs to generate the final product (V–VII). This highlights the formation of a highly fluorinated aromatic byproduct, 2,3,5,6-tetrafluoropyridin-4-olate (TFPO). Despite not falling within the category of PFAS due to its aromaticity, we sought to investigate TFPO’s recovery given the rising concern in the sustainability and environmental impact of fluorine containing compounds.^19^ Based on TLC analysis TFPO removal from the organic phase during the acid–base workup occurs during the NaHCO_3_ wash. Hence, it is expected that TFPO generated from the reactions outlined here could be recovered using lyophilization or previous work in the group has shown that TFPO can be readily isolated using column chromatography from crude reaction mixtures.

**Scheme 4:**
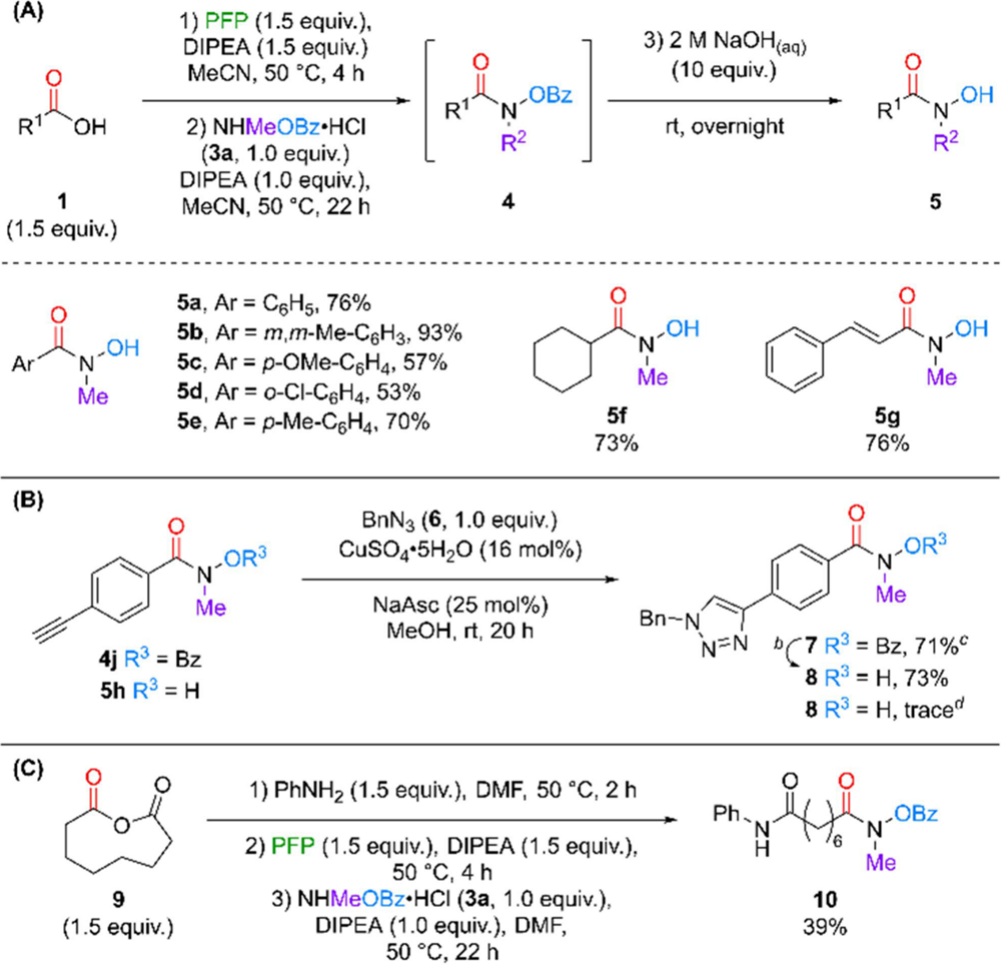
(A) One-Pot Synthesis of Free HAs; (B) Compatibility of Bz Protection with Transition Metals; (C) One-Pot Synthesis of a Vorinostat Analogue

a Isolated yield.
b 2 M NaOH_(aq)_ (10 equiv), THF, rt, 18 h.
c Starting from **4j.**

d Starting from **5h.**

**Scheme 5:**
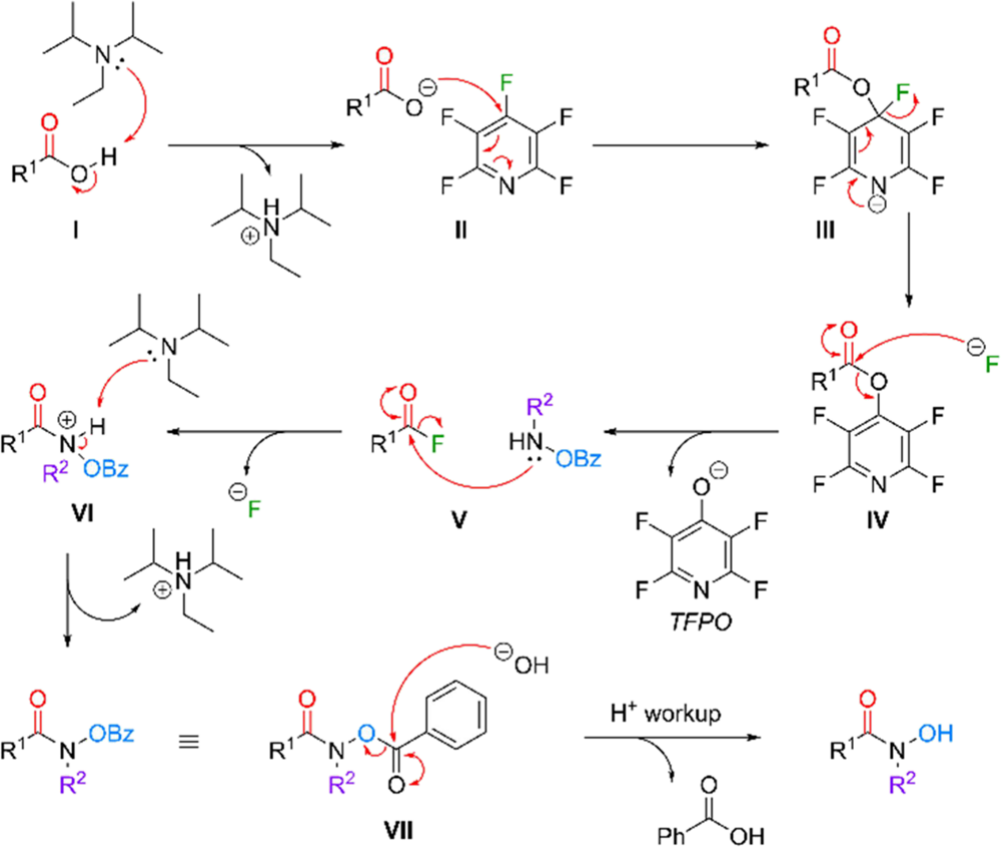
Proposed Mechanism for the Synthesis of *N*-Substituted HAs

The HA functionality has the potential to negatively affect transition metal-catalyzed processes through unwanted chelation. We therefore tested the utility of a protected analogue to increase compatibility in this area. As a test reaction, we selected the ubiquitous copper-catalyzed azide–alkyne cycloaddition (CuAAC, Scheme 4B).^20^ A terminal alkyne compound containing the Bz-protected HA moiety (**4j**) was subjected to typical CuAAC conditions which gave a 71% yield of the target triazole **7** which could then be readily deprotected to unveil triazole **8**. In contrast when we employed HA **5h** as the alkyne component, only a trace amount of triazole **8** was observed, presumably due to unwanted chelation of the active copper species.

As we had been able to successfully demonstrate one-pot sequential reactions to generate *N*-substituted HAs we wished to explore the synthesis of a medicinally relevant HA compound, namely an *N*-Me analogue of vorinostat which has previously been reported to be of biological importance.^21^ We believed that this was an interesting target as we could expand the type of reactions in one sequence going from a commercially available starting material with no intermediate purification or isolation. Starting from anhydride **9**, ring opening with aniline was conducted followed by acyl fluoride generation then a final coupling step with hydroxylamine **3a**. The protected vorinostat analogue target was successfully isolated in 39% yield over 3 sequential one-pot steps. In this example, DMF was used on account of starting material solubility, with the reactions still proceeding smoothly.

## Conclusions

In summary, we have developed a procedure for the synthesis of protected and unprotected *N*-substituted HA analogues directly from CAs. The use of an *in situ* generated acyl fluoride reactive intermediate allows for convenient further reaction with the protected hydroxylamine to generate a wide scope of OBz containing materials. It was then found that sequential addition of base directly into the reaction mixture allowed for removal of the PG and that HAs could be isolated cleanly with simple acid–base extraction. Subsequently, the utility of the PG strategy was assessed to circumvent limitations of HAs in transition metal-catalyzed procedures. This revealed that, in the cases of late-stage functionalization using metals, the reported strategy was important to help maximize yield and prevent termination of catalytic processes. Finally, we showed the synthesis of a medicinally relevant drug analogue in a one-pot process from a commercial starting material. This methodology demonstrates that HA synthesis can be conducted in a modular, robust and high yielding fashion directly from CAs.

## Supplementary Material



## Data Availability

The data underlying this study are available in the published article and its Supporting Information.

## Notes

Experimental procedures, characterization data, X-ray crystallographic data for **3a**, **4h**, **4j**, **4r** and **5h**, NMR spectra (PDF)
