# Supplementary material for: Synthesis of N‑Substituted Hydroxamic Acids through a Pentafluoropyridine-Mediated Procedure
Source: J Org Chem. 2026 May 5;91(19):6695–700. doi: 10.1021/acs.joc.6c00360 (PMC13185108; doi:10.1021/acs.joc.6c00360)
Supplement: Supplementary file 1 [file jo6c00360_si_001.pdf]

Synthesis of *N*-Substituted Hydroxamic Acids through a Pentafluoropyridine-Mediated Procedure

Daniel E. Bonn,[a] Toby J. Blundell,[a] Matthew Jenner,[b] and William D. G. Brittain*[a]

[a] Department of Chemistry, Durham University, South Road, Durham, DH1 3LE, United Kingdom

[b] Department of Chemistry, University of Warwick, Coventry CV4 7AL, United Kingdom

*Corresponding Author email: william.d.brittain@durham.ac.uk

Contents

General……………………………………………………………………………S-1

Synthesis of benzoyl-protected hydroxylamines (**3a-d**)……………………………S-3

Synthesis of benzoyl-protected hydroxamic acids and Weinreb amides (**4a-4aa**)……………………………………………………………………S-6

Synthesis of hydroxamic acids (**5a-h**)………………………………………………S-27

Synthesis of other compounds (**6-10**)………………………………………………S-33

NMR data for synthesised compounds…………………………………………S-38

X-ray Crystallography……………………………………………………………S-128

References………………………………………………………………………S-132

Experimental

General

All starting materials and reagents were purchased from commercial sources and used as received. NaN_3 should be kept away from metal sources to avoid formation of any explosive inorganic azides and should not be heated aggressively due to potential explosive decomposition. Organic azides can be potentially explosive dependent on their C:N ratio. Unless necessary, organic azides should not be isolated if their C:N ratio is below 2:1. Pentafluoropyridine (PFP) has no known toxicity and is not corrosive. However, it has the

potential to generate HF in the presence of aqueous acid. *N*,*N*-Diisopropylethylamine (DIPEA) was handled with appropriate care given its known flammability and toxicity. Material safety data sheets (MSDS) should always be referred to before conducting any chemical reactions. MeCN and DMF were dried over 4 Å molecular sieves which had been dried under reduced pressure at 150 °C for 3 h. All reactions were conducted under an atmosphere of air unless otherwise stated. Column chromatography was carried out on silica purchased from Fluorochem using hexane/ethyl acetate solvent systems or using a Combiflash Nextgen 100 equipped with a 4g/12g redisep column using a hexane/ethyl acetate solvent system. A Radleys Carousel Core+ Stirring Hotplate was used for reactions that required heating.

^{1}H NMR spectra were recorded at 400 and 700 MHz using Bruker Neo-400 and Bruker Neo 700 MHz spectrometers respectively. ^{13}C NMR spectra were recorded at 100, 126, 151 and 176 MHz using a Bruker Neo-400, Varian DD2-500, Varian VNMRS-600 and Bruker Neo 700 MHz spectrometers respectively. ^{19}F NMR spectra were recorded at 376 MHz using a Bruker Avance III spectrometer. All coupling constants are reported in Hertz (Hz). In cases where it was required 2D NMR techniques were used to confirm compound identity. Chemical shifts are reported in ppm and are referenced to residual solvent peaks; $CHCl_3$ (^{1}H 7.26 ppm, ^{13}C 77.16 ppm), CH_3CN (^{1}H 1.94 ppm, ^{13}C 1.32 ppm), MeOH (^{1}H 3.31 ppm, ^{13}C 49.00 ppm) and DMSO (^{1}H 2.50 ppm, ^{13}C 39.52 ppm). The following abbreviations were used for multiplicities: app. = apparent, s = singlet, d = doublet, dd = doublet of doublets, dt = doublet of triplets, t = triplet, td = triplet of doublets, tt = triplet of triplets, q = quartet, p = pentet, hept = heptet, m = unresolved multiplet, br s = broad singlet.

Mass spectra were collected either using LCMS (ESI$^+$), GCMS (EI$^+$) or ASAP (AI$^+$). LCMS in MeCN were collected using a Waters TQD mass spectrometer with a Acquity UPLC BEH C18 1.7 µm (2.1 mm x 50 mm). LCMS was collected using water containing formic acid (0.1% v/v) and MeCN mixture in a 95:5 to 5:95 gradient over 5 min. GCMS experiments were carried out on a Shimadzu QP2010-Ultra with a Rxi-5Sil MS column (0.15 µm x 10m x 0.15 mm). Helium was employed as the carrier gas (0.41 mL/min). EI is carried at 70 ev and the working mass range is 35 – 650 u for all GCMS experiments. HRMS was performed using a Waters QtoF Premier mass spectrometer equipped with an ASAP probe or a Waters LCT Premier XE mass spectrometer combined with Acquity UPLC and ASAP.

Single crystals of **3a**, **4h**, **4j**, **4r** and **5h** were prepared from slow evaporation of DCM. Crystals were mounted on MitiGen micromounts in perfluoroether oil before being placed in the

cryostream of the diffractometer. The X-ray single crystal data have been collected at a temperature of 120.0(2) K using MoKα (λ = 0.71073 Å) or CuKα (λ = 1.54178 Å) radiation on a Bruker D8Venture with a Photon III MM C7 or C14 CPAD detector, IμS-III-microsource, focusing mirrors diffractometer equipped with a Cryostream (Oxford Cryosystems 700+) open-flow nitrogen cryostat. The structures were solved using Olex2[1]with the ShelXT[2] structure solution program using Intrinsic Phasing and refined with Olex2.refine[3] refinement package using Least Squares minimization on F^2. Calculation of non-spherical atomic form factors were obtained through the NoSpherA2[4] plugin in Olex2 using Orca 6.1[5]. For more details see the specific cif files. All non-hydrogen atoms were refined with anisotropic displacement parameters. Hydrogen atoms were located on the difference map and freely refined isotropically apart from the disordered structure **4r** where hydrogen atoms were geometrically placed and refined on a riding model using neutron distances. Crystal data and parameters of refinement are listed in **Table S1** (S-128). Crystallographic data for the structure have been deposited with the Cambridge Crystallographic Data Centre with deposition number CCDC-2512797-2512801 for **3a**, **4h**, **4j**, **4r**, and **5h** respectively.

Infrared spectra were recorded on a PerkinElmer Frontier Infra-Red Spectrometer set-up with Specac Quest Single Reflection ATR accessory with Extended Range Diamond crystal puck (P/N GS10811, S/N U61757).

Synthesis of benzoyl-protected hydroxylamines (3a–d)

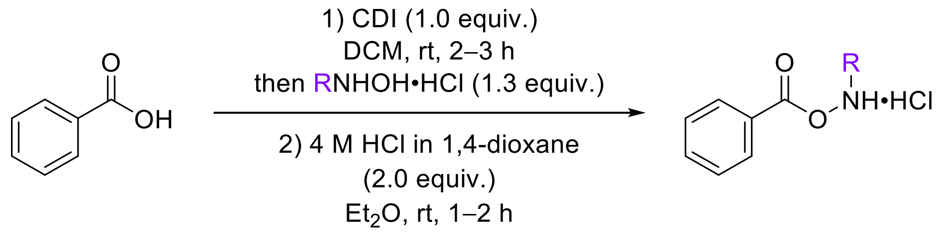

General procedure A

Adapted from a literature procedure.[6] To a solution of benzoic acid (1.0 equiv.) in DCM, CDI (1.0 equiv.) was added slowly at 0 °C and left to stir for 0.5–1 h at rt. Following the addition of hydroxylamine hydrochloride (1.3 equiv.), the solution was stirred for 2–3 h at rt. The resulting solution was sequentially washed with cold 1 M HCl and sat. aq. $NaHCO_3$. The organic layer was dried with anhydrous Na_2SO_4 or $MgSO_4$, filtered and concentrated under reduced pressure. The crude residue was then dissolved in Et_2O to which 4 M HCl in 1,4-

dioxane (2.0 equiv.) was added and stirred for 1–2 h at rt. The resulting precipitate was isolated by vacuum filtration and washed with a minimal volume of cold Et_2O to give the desired compounds without further purification.

_N_-Methyl-_O_-benzoylhydroxylamine hydrochloride (3a)

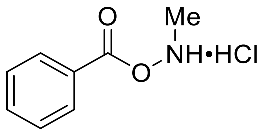

Synthesised according to general procedure A from a solution of benzoic acid (7.78 g, 63.7 mmol), CDI (10.43 g, 64.35 mmol) and *N*-methylhydroxylamine hydrochloride (6.92 g, 82.8 mmol) in DCM (120 mL). Precipitation using Et_2O (85 mL) and 4 M HCl in 1,4-dioxane (32 mL) gave the target product as a white solid in 54% yield (6.48 g, 34.5 mmol).

Characterisation data was consistent with previously reported literature values.[7]

^{1}H NMR (400 MHz, $CDCl_3$) δ_H 11.05 (br s, 2H), 8.03 – 7.96 (m, 2H), 7.71 – 7.63 (m, 1H), 7.52 – 7.43 (m, 2H), 3.32 (s, 3H).

^{13}C{^{1}H} NMR (101 MHz, $CDCl_3$) δ_C 163.0, 135.4, 130.4, 129.1, 124.7, 36.2.

LCMS (ESI$^+$) r.t. = 1.5 min, *m/z* = 152 [M+H]$^+$.

HRMS (ESI$^+$) *m/z*: calcd. for $[C_8H_{10}NO_2]^+$ 152.0712, found 152.0709 [M+H]$^+$.

FTIR (neat) ν_{max} / cm^{-1} 2320 (br), 1768 (m), 1691 (w), 1598 (w), 1451 (w), 1240 (m), 1181 (w), 1157 (w), 1040 (w), 1024 (w).

_O_-Benzoyl-_N_-(_tert_-butyl)hydroxylamine hydrochloride (3b)

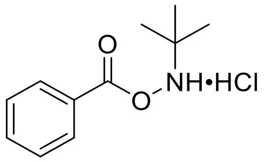

Synthesised according to general procedure A from a solution of benzoic acid (3.74 g, 30.6 mmol), CDI (4.97 g, 30.7 mmol) and *N*-*tert*-butylhydroxylamine hydrochloride (5.00 g, 39.8 mmol) in DCM (70 mL). Precipitation using Et_2O (60 mL) and 4 M HCl in 1,4-dioxane (18 mL) gave the target product as a white solid in 86% yield (6.02 g, 26.2 mmol).

^{1}H NMR (400 MHz, $CDCl_3$) δ_H 10.57 (br s, 2H), 7.89 – 7.82 (m, 2H), 7.63 (tt, J = 7.5, 1.2, 1H), 7.45 – 7.36 (m, 2H), 1.66 (s, 9H).

$^{13}C\{^1H\}$ NMR (101 MHz, $CDCl_3$) δ_C 162.6, 135.0, 130.3, 129.0, 125.2, 62.9, 24.7.

LCMS (ESI^+) r.t. = 2.4 min, *m/z* = 194 $[M+H]^+$.

HRMS (AI^+) *m/z*: calcd. for $[C_{11}H_{16}NO_2]^+$ 194.1181, found 194.1174 $[M+H]^+$.

FTIR (neat) ν_{max} / cm^{-1} 2984 (m), 2658 (m), 2562 (m), 2456 (m), 2321 (br), 2030 (w), 1921 (w), 1762 (vs), 1598 (m), 1455 (m), 1395 (m), 1378 (m), 1241 (s), 1048 (s), 1023 (m).

m.p. 152 – 154 °C.

_O_-Benzoyl-_N_-isopropylhydroxylamine hydrochloride (3c)

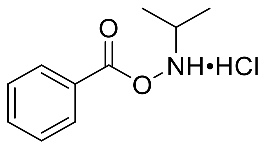

Synthesised according to general procedure A from a solution of benzoic acid (4.29 g, 35.2 mmol), CDI (5.70 g, 35.1 mmol) and *N*-isopropylhydroxylamine hydrochloride (5.09 g, 45.6 mmol) in DCM (70 mL). Precipitation using Et_2O (60 mL) and 4 M HCl in 1,4-dioxane (18 mL) gave the target product as a white solid in 84% yield (6.39 g, 29.6 mmol).

^{1}H NMR (400 MHz, $CDCl_3$) δ_H 9.87 (br s, 2H), 7.98 – 7.90 (m, 2H), 7.65 (tt, J = 7.5, 1.2, 1H), 7.52 – 7.38 (m, 2H), 4.13 (hept, J = 6.5, 1H), 1.59 (d, J = 6.5, 6H).

$^{13}C\{^1H\}$ NMR (101 MHz, $CDCl_3$) δ_C 162.9, 135.2, 130.4, 129.1, 125.0, 54.4, 17.4.

LCMS (ESI^+) r.t. = 2.2 min, *m/z* = 180 $[M+H]^+$.

HRMS (AI^+) *m/z*: calcd. for $[C_{10}H_{14}NO_2]^+$ 180.1025, found 180.1015 $[M+H]^+$.

FTIR (neat) ν_{max} / cm^{-1} 3317 (br), 2578 (m), 2335 (br), 2030 (w), 1763 (s), 1597 (m), 1449 (m), 1397 (m), 1237 (s), 1185 (m), 1039 (vs), 1017 (vs).

m.p. 130 – 133 °C.

***O*-Benzoyl-*N*-benzylhydroxylamine hydrochloride (3d)**

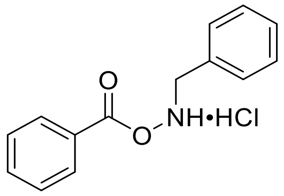

Synthesised according to general procedure A from a solution of benzoic acid (2.73 g, 22.4 mmol), CDI (3.68 g, 22.7 mmol) and *N*-benzylhydroxylamine hydrochloride (4.66 g, 29.2 mmol) in DCM (50 mL). Precipitation using Et_2O (50 mL) and 4 M HCl in 1,4-dioxane (11.5 mL) gave the target product as a white solid in 78% yield (4.60 g, 17.4 mmol).

^{1}H NMR (400 MHz, MeOD) δ_H 8.04 – 7.99 (m, 2H), 7.78 – 7.71 (m, 1H), 7.62 – 7.54 (m, 4H), 7.49 – 7.44 (m, 3H), 4.71 (s, 2H).

^{13}C{^{1}H} NMR (101 MHz, MeOD) δ_C 164.7, 136.3, 131.8, 131.0, 130.9, 130.9, 130.3, 130.2, 126.5, 55.3.

LCMS (ESI$^+$) r.t. = 2.5 min, *m/z* = 228 [M+H]$^+$.

HRMS (AI$^+$) *m/z*: calcd. for $[C_{14}H_{14}NO_2]^+$ 228.1025, found 228.1021 [M+H]$^+$.

FTIR (neat) ν_{max} / cm^{-1} 2315 (br), 1758 (s), 1599 (w), 1453 (m), 1392 (m), 1304 (m), 1239 (m), 1075 (m), 1051 (s), 1026 (m).

m.p. 128 – 132 °C.

Synthesis of benzoyl-protected hydroxamic acids and Weinreb amides (4a–4ab)

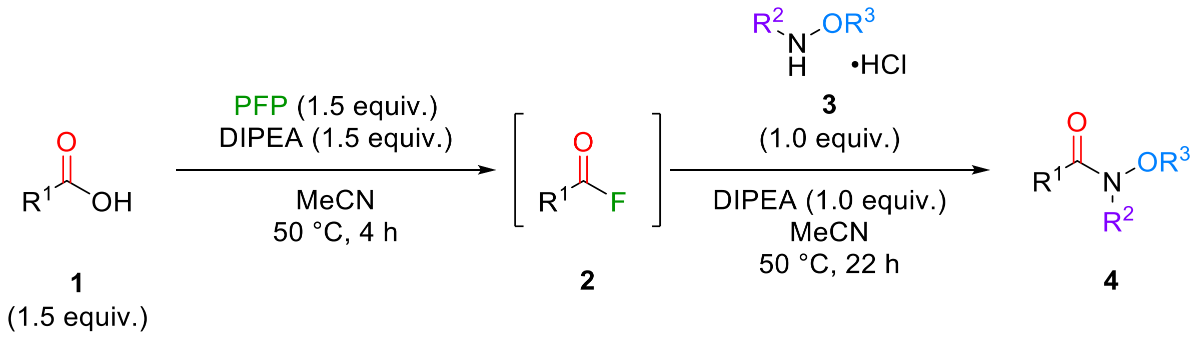

General procedure B:

To an oven-dried sealed vial or round bottom flask, PFP (1.5 equiv.) was added to a solution of carboxylic acid (1.5 equiv.) and DIPEA (1.5 equiv.) in MeCN and stirred at 50 °C for 4 h. To a separate vial or round bottom flask, a solution of hydroxylamine hydrochloride (1.0 equiv.), DIPEA (1.0 equiv.) in MeCN was prepared and subsequently added to the reaction mixture. The resulting solution was left to stir for 22 h at 50 °C. The reaction mixture was then concentrated under reduced pressure, diluted in DCM (15 mL), and washed with cold 0.5 M HCl (20 mL) and sat. aq. $NaHCO_3$ (20 mL). The organic layer was then dried with anhydrous Na_2SO_4, filtered and concentrated under reduced pressure. The crude material was then dissolved in a minimum amount of DCM and purified by flash column chromatography to give the desired compounds.

***N*-(Benzoyloxy)-*N*-methylbenzamide (4a)**

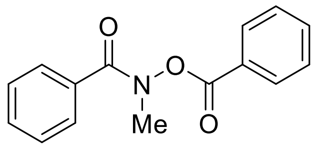

Synthesised according to general procedure B from a solution of PFP (153 µL, 1.46 mmol), benzoic acid (0.18 g, 1.47 mmol) and DIPEA (250 µL, 1.46 mmol) in MeCN (3 mL) and a solution of *N*-methyl-*O*-benzoylhydroxylamine hydrochloride (0.18 g, 0.96 mmol) and DIPEA (170 µL, 0.99 mmol) in MeCN (2 mL). The crude residue was purified by flash column chromatography (1:0 to 4:1, hexane:EtOAc) to give the target product as a clear oil in 90% yield (0.22 g, 0.90 mmol).

Characterisation data was consistent with previously reported literature values.[8]

^{1}H NMR (400 MHz, $CDCl_3$) δ_H 7.94 – 7.87 (m, 2H), 7.69 – 7.51 (m, 3H), 7.46 – 7.28 (m, 5H), 3.52 (s, 3H).

^{13}C{^{1}H} NMR (101 MHz, $CDCl_3$) δ_C 171.4, 164.5, 134.3, 133.4, 131.1, 129.9, 128.8, 128.3, 128.0, 126.9, 37.4.

LCMS (ESI$^+$) r.t = 2.4 min, *m/z* = 256 [M+H]$^+$.

HRMS (ESI$^+$) *m/z*: calcd. for $[C_{15}H_{14}NO_3]^+$ 256.0974, found 256.0974 [M+H]$^+$.

FTIR (neat) ν_{max} / cm^{-1} 1758 (m), 1666 (m), 1600 (w), 1450 (m), 1415 (w), 1354 (m), 1241 m), 1204 (m), 1177 (m), 1061 (w).

***N*-(Benzoyloxy)-*N*-methyl-4-(trifluoromethyl)benzamide (4b)**

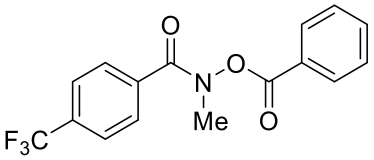

Synthesised according to general procedure B from a solution of PFP (153 µL, 1.46 mmol), 4-(trifluoromethyl)benzoic acid (0.28 g, 1.47 mmol) and DIPEA (250 µL, 1.46 mmol) in MeCN (3 mL) and a solution of *N*-methyl-*O*-benzoylhydroxylamine hydrochloride (0.18 g, 0.96 mmol) and DIPEA (170 µL, 0.99 mmol) in MeCN (2 mL). The crude residue was purified by flash column chromatography (1:0 to 9:1, hexane:EtOAc) to give the target product as a colourless oil in 48% yield (0.15 g, 0.46 mmol).

^{1}H NMR (400 MHz, $CDCl_3$) δ_H 7.89 (app. d, *J* = 7.6, 2H), 7.75 (d, *J* = 8.0, 2H), 7.63 – 7.58 (m, 3H), 7.43 (t, *J* = 7.6, 2H), 3.53 (s, 3H).

^{13}C{^{1}H} NMR (101 MHz, $CDCl_3$) δ_C 170.0, 164.4, 137.0, 134.6, 132.8 (q, *J* = 32.7), 130.0, 128.9, 128.4, 126.5, 125.3 (q, *J* = 3.8), 123.4 (q, *J* = 272.5), 37.1.

^{19}F{^{1}H} NMR (376 MHz, $CDCl_3$) δ_F -63.07.

LCMS (ESI^{+}) r.t = 2.8 min, *m/z* = 324 $[M+H]^{+}$.

HRMS (ESI^{+}) *m/z*: calcd. for $[C_{16}H_{13}NO_3F_3]^{+}$ 324.0848, found 324.0850 $[M+H]^{+}$.

FTIR (neat) ν_{max} / cm^{-1} 1761 (m), 1669 (m), 1601 (w), 1452 (w), 1408 (w), 1370 (m), 1321 (s), 1241 (m), 1208 (m), 1165 (m), 1123 (m), 1067 (s).

***N*-(Benzoyloxy)-*N*-methyl-3-nitrobenzamide (4c)**

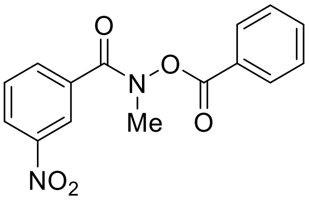

Synthesised according to general procedure B from a solution of PFP (153 μL, 1.46 mmol), 3-nitrobenzoic acid (0.24 g, 1.44 mmol) and DIPEA (250 μL, 1.46 mmol) in MeCN (3 mL) and a solution of *N*-methyl-*O*-benzoylhydroxylamine hydrochloride (0.18 g, 0.96 mmol) and DIPEA (170 μL, 0.99 mmol) in MeCN (2 mL). The crude residue was purified by flash column chromatography (1:0 to 4:1, hexane:EtOAc) to give the target product as a yellow oil in 39% yield (0.11 g, 0.37 mmol).

^{1}H NMR (400 MHz, $CDCl_3$) δ_H 8.57 – 8.52 (m, 1H), 8.26 – 8.19 (m, 1H), 8.01 – 7.97 (m, 1H), 7.93 – 7.88 (m, 2H), 7.64 – 7.58 (m, 1H), 7.53 (t, *J* = 8.1, 1H), 7.46 – 7.39 (m, 2H), 3.56 (s, 3H).

^{13}C{^{1}H} NMR (101 MHz, $CDCl_3$) δ_C 168.8, 164.4, 147.8, 134.9, 134.8, 134.2, 130.0, 129.6, 129.0, 126.2, 125.8, 123.3, 37.0.

LCMS (ESI$^+$) r.t = 2.6 min, *m/z* = 301 [M+H]$^+$.

HRMS (AI$^+$) *m/z*: calcd. for $[C_{15}H_{13}N_2O_5]^+$ 301.0824, found 301.0815 [M+H]$^+$.

FTIR (neat) ν_{max} / cm^{-1} 3091 (w), 1759 (s), 1666 (s), 1530 (s), 1451 (m), 1348 (s), 1242 (s), 1207 (m), 1161 (m), 1098 (w).

***N*-(Benzoyloxy)-*N*-methyl-4-nitrobenzamide (4d)**

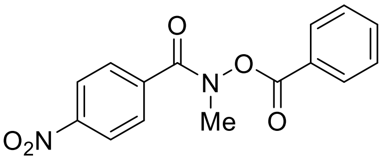

Synthesised according to general procedure B from a solution of PFP (151 μL, 1.44 mmol), 4-nitrobenzoic acid (0.24 g, 1.44 mmol) and DIPEA (250 μL, 1.46 mmol) in MeCN (3 mL) and a solution of *N*-methyl-*O*-benzoylhydroxylamine hydrochloride (0.18 g, 0.96 mmol) and

DIPEA (170 μL, 0.99 mmol) in MeCN (2 mL). The crude residue was purified by flash column chromatography (1:0 to 4:1, hexane:EtOAc) to give the target product as an off-white solid in 39% yield (0.11 g, 0.37 mmol).

^{1}H NMR (400 MHz, $CDCl_3$) δ_H 8.18 (dt, J = 8.8, 2.1, 2H), 7.92 – 7.84 (m, 2H), 7.79 (dt, J = 8.8, 2.1, 2H), 7.66 – 7.57 (m, 1H), 7.48 – 7.39 (m, 2H), 3.54 (s, 3H).

^{13}C{^{1}H} NMR (101 MHz, $CDCl_3$) δ_C 169.4, 164.3, 149.2, 139.4, 134.9, 130.0, 129.1, 129.1, 126.2, 123.5, 36.9.

LCMS (ESI$^+$) r.t = 2.6 min, m/z = 301 [M+H]$^+$.

HRMS (ESI$^+$) m/z: calcd. for $[C_{15}H_{13}N_2O_5]^+$ 301.0824, found 301.0816 [M+H]$^+$.

FTIR (neat) ν_{max} / cm^{-1} 1756 (s), 1657 (s), 1599 (m), 1520 (s), 1450 (m), 1414 (m), 1341 (s), 1251 (m), 1200 (m), 1114 (m), 1065 (m), 1000 (s).

m.p. 60 – 63 °C.

N-(Benzoyloxy)-4-cyano-*N*-methylbenzamide (4e)

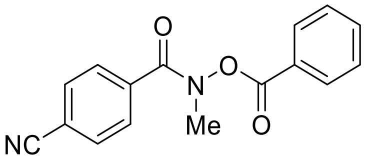

Synthesised according to general procedure B from a solution of PFP (152 μL, 1.45 mmol), 4-cyanobenzoic acid (0.21 g, 1.43 mmol) and DIPEA (250 μL, 1.46 mmol) in MeCN (3 mL) and a solution of *N*-methyl-*O*-benzoylhydroxylamine hydrochloride (0.18 g, 0.96 mmol) and DIPEA (170 μL, 0.99 mmol) in MeCN (2 mL). The crude residue was purified by flash column chromatography (1:0 to 4:1, hexane:EtOAc) to give the target product as a colourless oil in 45% yield (0.12 g, 0.43 mmol).

^{1}H NMR (400 MHz, $CDCl_3$) δ_H 7.91 – 7.84 (m, 2H), 7.75 – 7.69 (m, 2H), 7.65 – 7.59 (m, 3H), 7.47 – 7.41 (m, 2H), 3.52 (s, 3H).

^{13}C{^{1}H} NMR (101 MHz, $CDCl_3$) δ_C 169.6, 164.3, 137.7, 134.8, 132.1, 129.9, 129.0, 128.6, 126.3, 118.0, 114.7, 37.0.

LCMS (ESI^+) r.t = 2.5 min, *m/z* = 280 $[M+H]^+$.

HRMS (ESI^+) *m/z*: calcd. for $[C_{16}H_{13}N_2O_3]^+$ 281.0926, found 281.0916 $[M+H]^+$.

FTIR (neat) ν_{max} / cm^{-1} 2927 (w), 2231 (m), 1759 (s), 1666 (s), 1600 (m), 1451 (m), 1365 (m), 1241 (s), 1206 (m), 1176 (m).

***N*-(Benzoyloxy)-*N*,4-dimethylbenzamide (4f)**

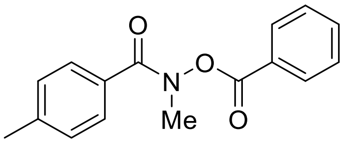

Synthesised according to general procedure B from a solution of PFP (151 µL, 1.44 mmol), *p*-toluic acid (0.20 g, 1.47 mmol) and DIPEA (250 µL, 1.46 mmol) in MeCN (3 mL) and a solution of *N*-methyl-*O*-benzoylhydroxylamine hydrochloride (0.18 g, 0.96 mmol) and DIPEA (170 µL, 0.99 mmol) in MeCN (2 mL). The crude residue was purified by flash column chromatography (1:0 to 4:1, hexane:EtOAc) to give the target product as a colourless oil in 81% yield (0.21 g, 0.78 mmol).

^1H NMR (400 MHz, $CDCl_3$) δ_H 7.97 – 7.90 (m, 2H), 7.62 – 7.57 (m, 1H), 7.55 (dt, *J* = 8.1, 1.8, 2H), 7.48 – 7.39 (m, 2H), 7.17 – 7.09 (m, 2H), 3.51 (s, 3H), 2.31 (s, 3H).

$^{13}C\{^1H\}$ NMR (101 MHz, $CDCl_3$) δ_C 171.6, 164.5, 141.6, 134.3, 130.4, 130.0, 129.0, 128.8, 128.2, 127.1, 37.6, 21.6.

LCMS (ESI^+) r.t = 2.8 min, *m/z* = 270 $[M+H]^+$.

HRMS (ESI^+) *m/z*: calcd. for $[C_{16}H_{16}NO_3]^+$ 270.1130, found 270.1117 $[M+H]^+$.

FTIR (neat) ν_{max} / cm^{-1} 1759 (s), 1655 (vs), 1598 (w), 1448 (m), 1363 (m), 1316 (w), 1244 (s), 1165 (m), 1033 (m), 1021 (m), 1009 (s).

m.p. 72 – 74 °C.

N-(Benzoyloxy)-*N*-methylhexanamide (4g)

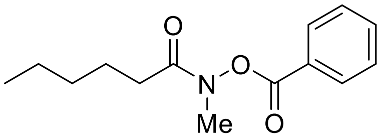

Synthesised according to general procedure B from a solution of PFP (152 µL, 1.45 mmol), hexanoic acid (181 µL, 1.44 mmol) and DIPEA (250 µL, 1.46 mmol) in MeCN (3 mL) and a solution of *N*-methyl-*O*-benzoylhydroxylamine hydrochloride (0.18 g, 0.96 mmol) and DIPEA (170 µL, 0.99 mmol) in MeCN (2 mL). The crude residue was purified by flash column chromatography (1:0 to 4:1, hexane:EtOAc) to give the target product as a clear oil in 88% yield (0.21 g, 0.84 mmol).

^{1}H NMR (400 MHz, $CDCl_3$) δ_H 8.13 – 8.06 (m, 2H), 7.72 – 7.63 (m, 1H), 7.52 (t, J = 7.8, 2H), 3.40 (s, 3H), 2.31 (t, J = 7.5, 2H), 1.64 (p, J = 8.3, 2H), 1.32 – 1.23 (m, 4H), 0.85 (t, J = 6.8, 3H).

$^{13}C\{^1H\}$ NMR (151 MHz, $CDCl_3$) δ_C 174.4, 164.5, 134.6, 130.1, 129.0, 127.0, 35.7, 32.3, 31.5, 24.2, 22.5, 14.0.

LCMS (ESI$^+$) r.t = 2.7 min, *m/z* = 250 [M+H]$^+$.

HRMS (ESI$^+$) *m/z*: calcd. for $[C_{14}H_{20}NO_3]^+$ 250.1443, found 250.1434 [M+H]$^+$.

FTIR (neat) ν_{max} / cm^{-1} 2956 (m), 2932 (m), 2872 (w) 1761 (s), 1677 (s), 1600 (w), 1452 (m), 1417 (m), 1317 (m), 1242 (s), 1173 (m), 1011 (s).

N-(Benzoyloxy)-4-methoxy-*N*-methylbenzamide (4h)

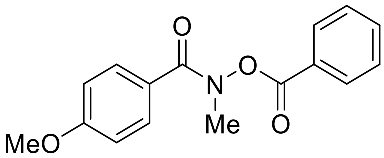

Synthesised according to general procedure B from a solution of PFP (151 µL, 1.44 mmol), 4-methoxybenzoic acid (0.22 g, 1.45 mmol) and DIPEA (250 µL, 1.46 mmol) in MeCN (3 mL) and a solution of *N*-methyl-*O*-benzoylhydroxylamine hydrochloride (0.18 g, 0.96 mmol) and DIPEA (170 µL, 0.99 mmol) in MeCN (2 mL). The crude residue was purified by flash column

chromatography (1:0 to 4:1, hexane:EtOAc) to give the target product as an off-white solid in 84% yield (0.23 g, 0.81 mmol).

^{1}H NMR (400 MHz, $CDCl_3$) δ_H 7.97 – 7.90 (m, 2H), 7.66 – 7.61 (m, 2H), 7.60 – 7.55 (m, 1H), 7.41 (t, *J* = 7.7, 2H), 6.87 – 6.76 (m, 2H), 3.74 (s, 3H), 3.49 (s, 3H).

^{13}C{^{1}H} NMR (101 MHz, $CDCl_3$) δ_C 171.3, 164.5, 161.9, 134.2, 130.3, 129.9, 128.8, 127.0, 125.3, 113.5, 55.3, 37.5.

LCMS (ESI$^+$) r.t = 2.4 min, *m/z* = 286 [M+H]$^+$.

HRMS (ESI$^+$) *m/z*: calcd. for $[C_{16}H_{16}NO_4]^+$ 286.1079, found 286.1076 [M+H]$^+$.

FTIR (neat) ν_{max} / cm^{-1} 2939 (w), 1761 (s), 1654 (vs), 1608 (m), 1510 (m), 1454 (m), 1428 (m), 1365 (m), 1320 (w), 1246 (s), 1078, 1059 (m), 1006 (s).

m.p. 88 – 90 °C.

_N_-(Benzoyloxy)-_N_-methylcyclohexanecarboxamide (4i)

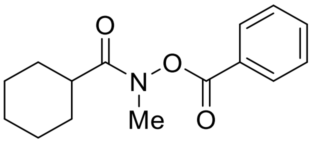

Synthesised according to general procedure B from a solution of PFP (151 µL, 1.44 mmol), cyclohexanecarboxylic acid (0.18 g, 1.40 mmol) and DIPEA (250 µL, 1.46 mmol) in MeCN (3 mL) and a solution of *N*-methyl-*O*-benzoylhydroxylamine hydrochloride (0.18 g, 0.96 mmol) and DIPEA (170 µL, 0.99 mmol) in MeCN (2 mL). The crude residue was purified by flash column chromatography (1:0 to 4:1, hexane:EtOAc) to give the target product as a clear oil in 80% yield (0.20 g, 0.77 mmol).

^{1}H NMR (400 MHz, $CDCl_3$) δ_H 8.12 – 8.04 (m, 2H), 7.71 – 7.62 (m, 1H), 7.56 – 7.48 (m, 2H), 3.39 (s, 3H), 2.40 (tt, *J* = 11.8, 3.3, 1H), 1.86 – 1.70 (m, 4H), 1.66 – 1.58 (m, 1H), 1.57 – 1.40 (m, 2H), 1.30 – 1.08 (m, 3H).

^{13}C{^{1}H} NMR (151 MHz, $CDCl_3$) δ_C 176.8, 164.6, 134.5, 130.0, 129.0, 127.1, 40.8, 36.0, 28.9, 25.8, 25.7.

LCMS (ESI^+) r.t = 2.7 min, *m/z* = 262 $[M+H]^+$.

HRMS (ESI^+) *m/z*: calcd. for $[C_{15}H_{20}NO_3]^+$ 262.1443, found 262.1452 $[M+H]^+$.

FTIR (neat) ν_{max} / cm^{-1} 2932 (m), 2855 (m), 1760 (s), 1670 (s), 1600 (w), 1584 (w), 1451 (m), 1418 (w), 1242 (s), 1171 (m).

_N_-(Benzoyloxy)-4-ethynyl-_N_-methylbenzamide (4j)

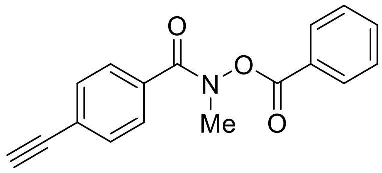

Synthesised according to general procedure B from a solution of PFP (1.48 mL, 14.1 mmol), 4-ethynylbenzoic acid (2.07 g, 14.2 mmol) and DIPEA (2.42 mL, 14.1 mmol) in MeCN (20 mL) and a solution of *N*-methyl-*O*-benzoylhydroxylamine hydrochloride (1.77 g, 9.43 mmol) and DIPEA (1.62 mL, 9.45 mmol) in MeCN (10 mL). The crude residue was purified by flash column chromatography (1:0 to 4:1, hexane:EtOAc) to give the target product as a cream solid in 72% yield (1.90 g, 6.80 mmol).

^1H NMR (400 MHz, $CDCl_3$) δ_H 7.92 – 7.88 (m, 2H), 7.63 – 7.58 (m, 3H), 7.47 – 7.40 (m, 4H), 3.51 (s, 3H), 3.13 (s, 1H).

$^{13}C\{^1H\}$ NMR (101 MHz, $CDCl_3$) δ_C 170.7, 164.5, 134.5, 133.5, 132.0, 130.0, 128.9, 128.1, 126.7, 125.0, 82.9, 79.3, 37.3.

LCMS (ESI^+) r.t = 2.7 min, *m/z* = 280 $[M+H]^+$.

HRMS (ESI^+) *m/z*: calcd. for $[C_{17}H_{14}NO_3]^+$ 280.0974, found 280.0977 $[M+H]^+$.

FTIR (neat) ν_{max} / cm^{-1} 3278 (m), 1760 (vs), 1657 (vs), 1599 (m), 1475 (m), 1449 (m), 1364 (m), 1244 (s), 1165 (m).

m.p. 108 – 110 °C.

***N*-(Benzoyloxy)-*N*-methylpicolinamide (4k)**

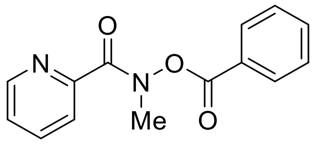

Synthesised according to general procedure B from a solution of PFP (152 μL, 1.45 mmol), 2-picolinic acid (0.18 g, 1.46 mmol) and DIPEA (250 μL, 1.46 mmol) in MeCN (3 mL) and a solution of *N*-methyl-*O*-benzoylhydroxylamine hydrochloride (0.18 g, 0.96 mmol) and DIPEA (170 μL, 0.99 mmol) in MeCN (2 mL). The crude residue was purified by flash column chromatography (1:0 to 4:1, hexane:EtOAc) to give the target product as a yellow oil in 53% yield (0.13 g, 0.51 mmol).

^{1}H NMR (400 MHz, $CDCl_3$) δ_H 8.45 (app. br s, 1H), 8.03 – 7.92 (m, 2H), 7.88 – 7.81 (m, 1H), 7.77 (td, *J* = 7.7, 1.6, 1H), 7.60 (tt, *J* = 7.5, 1.3, 1H), 7.43 (app. t, *J* = 7.8, 2H), 7.31 (app. t, *J* = 6.3, 1H), 3.67 (s, 3H).

$^{13}C\{^1H\}$ NMR (126 MHz, DMSO-d_6) δ_C 165.7, 163.5, 151.0, 148.0, 136.8, 133.8, 129.0, 128.5, 126.6, 125.2, 123.2, 37.6 – variable temperature NMR run at 80.0 °C.

LCMS (ESI$^+$) r.t = 1.9 min, *m/z* = 257 [M+H]$^+$.

HRMS (ESI$^+$) *m/z*: calcd. for $[C_{14}H_{13}N_2O_3]^+$ 257.0926, found 257.0926 [M+H]$^+$.

FTIR (neat) ν_{max} / cm^{-1} 1761 (m), 1661 (m), 1600 (w), 1584 (w), 1569 (w), 1451 (w), 1416 (w), 1373 (w), 1241 (m), 1171 (m).

***N*-(Benzoyloxy)-2-(4-isobutylphenyl)-*N*-methylpropanamide (4l)**

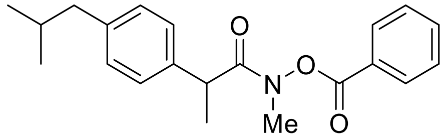

Synthesised according to general procedure B from a solution of PFP (152 μL, 1.45 mmol), 2-(4-isobutylphenyl)propanoic acid (0.30 g, 1.45 mmol) and DIPEA (250 μL, 1.46 mmol) in MeCN (3 mL) and a solution of *N*-methyl-*O*-benzoylhydroxylamine hydrochloride (0.18 g, 0.96 mmol) and DIPEA (170 μL, 0.99 mmol) in MeCN (2 mL). The crude residue was purified

by flash column chromatography (1:0 to 4:1, hexane:EtOAc) to give the target product as a clear oil in 83% yield (0.27 g, 0.80 mmol).

^{1}H NMR (400 MHz, CDCl$_3$) δ_H 7.91 (d, *J* = 7.7, 2H), 7.67 – 7.62 (m, 1H), 7.46 (t, *J* = 7.8, 2H), 7.07 – 7.02 (m, 2H), 7.02 – 6.98 (m, 2H), 3.80 (q, *J* = 7.0, 1H), 3.37 (s, 3H), 2.42 (d, *J* = 7.2, 2H), 1.82 (app. hept, *J* = 6.8, 1H), 1.44 (d, *J* = 7.0, 3H), 0.89 (dd, *J* = 6.6, 2.3, 6H).

^{13}C{^{1}H} NMR (151 MHz, CDCl$_3$) δ_C 174.9, 163.9, 140.4, 138.1, 134.4, 130.1, 129.5, 128.8, 127.2, 127.0, 45.1, 42.9, 36.1, 30.3, 22.5, 22.5, 19.7.

LCMS (ESI$^+$) r.t = 3.4 min, *m/z* = 340 [M+H]$^+$.

HRMS (ESI$^+$) *m/z*: calcd. for [$C_{21}H_{26}NO_3$]$^+$ 340.1913, found 340.1913 [M+H]$^+$.

FTIR (neat) ν_{max} / cm^{-1} 2954 (m), 2869 (w), 1763 (s), 1673 (s), 1600 (m), 1584 (w), 1511 (w), 1451 (m), 1376 (m), 1241 (s), 1168 (m).

_N_-(Benzoyloxy)-2-(6-methoxynaphthalen-2-yl)-_N_-methylpropanamide (4m)

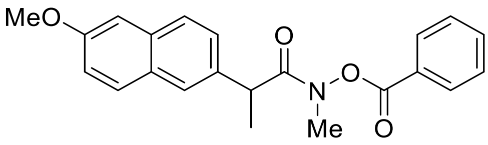

Synthesised according to general procedure B from a solution of PFP (152 µL, 1.45 mmol), 2-(6-methoxy-2-naphthyl)propionic acid (0.33 g, 1.43 mmol) and DIPEA (250 µL, 1.46 mmol) in MeCN (3 mL) and a solution of *N*-methyl-*O*-benzoylhydroxylamine hydrochloride (0.18 g, 0.96 mmol) and DIPEA (170 µL, 0.99 mmol) in MeCN (2 mL). The crude residue was purified by flash column chromatography (1:0 to 4:1, hexane:EtOAc) to give the target product as a pale-yellow solid in 77% yield (0.27 g, 0.74 mmol).

^{1}H NMR (400 MHz, CDCl$_3$) δ_H 7.87 (app. br s, 2H), 7.72 – 7.60 (m, 2H), 7.52 – 7.39 (m, 4H), 7.31 (d, *J* = 8.5, 1H), 7.15 – 7.04 (m, 2H), 3.95 (q, *J* = 6.9, 1H), 3.90 (s, 3H), 3.39 (s, 3H), 1.53 (d, *J* = 6.9, 3H).

^{13}C{^{1}H} NMR (101 MHz, CDCl$_3$) δ_C 174.6, 164.0, 157.6, 135.8, 134.4, 133.6, 130.1, 129.1, 128.9, 128.8, 127.4, 126.8, 126.3, 126.0, 119.0, 105.6, 55.3, 43.2, 36.0, 19.6.

LCMS (ESI$^+$) r.t = 2.9 min, *m/z* = 364 [M+H]$^+$.

HRMS (ESI$^+$) *m/z*: calcd. for $[C_{22}H_{22}NO_4]^+$ 364.1549, found 364.1550 $[M+H]^+$.

FTIR (neat) ν_{max} / cm^{-1} 2982 (w), 1762 (s), 1672 (s), 1605 (m), 1506 (w), 1374 (m), 1242 (m), 1229 (m), 1177 (m).

m.p. 102 – 104 °C.

_N_-(Benzoyloxy)-_N_-isopropyl-4-methylbenzamide (4n)

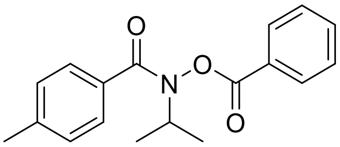

Synthesised according to general procedure B from a solution of PFP (152 μL, 1.45 mmol), *p*-toluic acid (0.20 g, 1.47 mmol) and DIPEA (250 μL, 1.46 mmol) in MeCN (3 mL) and a solution of *O*-benzoyl-*N*-isopropylhydroxylamine hydrochloride (0.21 g, 0.97 mmol) and DIPEA (170 μL, 0.99 mmol) in MeCN (2 mL). The crude residue was purified by flash column chromatography (1:0 to 4:1, hexane:EtOAc) to give the target product as a pale-yellow oil in 52% yield (0.15 g, 0.50 mmol).

^{1}H NMR (400 MHz, $CDCl_3$) δ_H 8.06 – 7.99 (m, 2H), 7.64 – 7.57 (m, 1H), 7.54 (dt, *J* = 8.1, 1.7, 2H), 7.49 – 7.42 (m, 2H), 7.16 (d, *J* = 7.8, 2H), 4.69 (hept, *J* = 6.6, 1H), 2.32 (s, 3H), 1.30 (d, *J* = 6.6, 6H).

$^{13}C\{^1H\}$ NMR (101 MHz, $CDCl_3$) δ_C 170.1, 164.6, 141.2, 134.0, 131.5, 130.0, 129.1, 128.7, 127.7, 127.3, 52.6, 21.5, 19.8.

LCMS (ESI$^+$) r.t = 2.9 min, *m/z* = 298 $[M+H]^+$.

HRMS (ESI$^+$) *m/z*: calcd. for $[C_{18}H_{20}NO_3]^+$ 298.1443, found 298.1433 $[M+H]^+$.

FTIR (neat) ν_{max} / cm^{-1} 2980 (m), 1762 (s), 1654 (s), 1611 (m)., 1451 (m), 1390 (m), 1368 9m), 1314 (m), 1236 (s), 1176 (m).

***N*-(Benzoyloxy)-*N*-benzyl-4-methoxybenzamide (4o)**

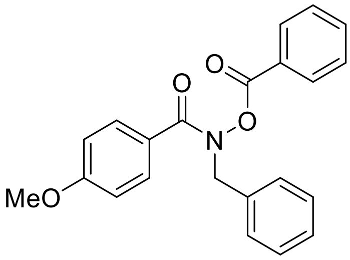

Synthesised according to general procedure B from a solution of PFP (153 µL, 1.46 mmol), 4-methoxybenzoic acid (0.22 g, 1.45 mmol) and DIPEA (250 µL, 1.46 mmol) in MeCN (3 mL) and a solution of *O*-benzoyl-*N*-benzylhydroxylamine hydrochloride (0.25 g, 0.95 mmol) and DIPEA (170 µL, 0.99 mmol) in MeCN (2 mL). The crude residue was purified by flash column chromatography (1:0 to 4:1, hexane:EtOAc) to give the target product as a pale-yellow oil in 46% yield (0.16 g, 0.44 mmol).

^{1}H NMR (700 MHz, $CDCl_3$) δ_H 7.87 (d, *J* = 7.9, 2H), 7.70 (d, *J* = 8.5, 2H), 7.55 (td, *J* = 7.5, 1.5, 1H), 7.42 (d, *J* = 7.6, 2H), 7.40 – 7.34 (m, 4H), 7.33 – 7.29 (m, 1H), 6.84 (d, *J* = 8.5, 2H), 5.11 (s, 2H), 3.75 (s, 3H).

^{13}C{^{1}H} NMR (176 MHz, $CDCl_3$) δ_C 170.7, 164.4, 162.0, 135.5, 134.1, 130.3, 129.9, 128.7, 128.7, 128.4, 127.9, 127.0, 125.3, 113.6, 55.3, 53.7.

LCMS (ESI$^+$) r.t = 3.0 min, *m/z* = 362 [M+H]$^+$.

HRMS (ESI$^+$) *m/z*: calcd. for $[C_{22}H_{20}NO_4]^+$ 362.1392, found 362.1379 [M+H]$^+$.

FTIR (neat) ν_{max} / cm^{-1} 2935 (w), 2840 (w), 1757 (s), 1660 (m), 1603 (s), 1511 (m)., 1452 (m), 1378 (m), 1343 (m), 1305 (m), 1252 (s), 1230 (vs), 1171 (s).

***N*-(Benzoyloxy)-*N*-benzyl-4-cyanobenzamide (4p)**

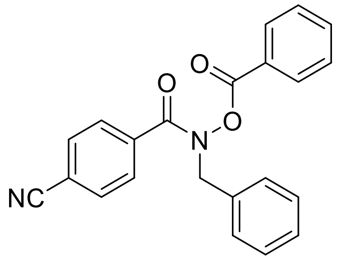

Synthesised according to general procedure B from a solution of PFP (153 µL, 1.46 mmol), 4-cyanobenzoic acid (0.22 g, 1.50 mmol) and DIPEA (250 µL, 1.46 mmol) in MeCN (3 mL) and a solution of *O*-benzoyl-*N*-benzylhydroxylamine hydrochloride (0.25 g, 0.95 mmol) and DIPEA (170 µL, 0.99 mmol) in MeCN (2 mL). The crude residue was purified by flash column chromatography (1:0 to 4:1, hexane:EtOAc) to give the target product as an off-white solid in 25% yield (0.084 g, 0.24 mmol).

^{1}H NMR (400 MHz, $CDCl_3$) δ_H 7.82 – 7.71 (m, 4H), 7.65 – 7.55 (m, 3H), 7.43 – 7.31 (m, 7H), 5.09 (s, 2H).

^{13}C{^{1}H} NMR (176 MHz, $CDCl_3$) δ_C 169.1, 164.3, 137.7, 134.7, 134.7, 132.1, 129.9, 128.9, 128.9, 128.7, 128.6, 128.3, 126.2, 118.0, 114.7, 53.1.

LCMS (ESI$^+$) r.t = 2.9 min, *m/z* = 357 [M+H]$^+$.

HRMS (ESI$^+$) *m/z*: calcd. for $[C_{22}H_{17}N_2O_3]^+$ 357.1239, found 357.1232 [M+H]$^+$.

FTIR (neat) ν_{max} / cm^{-1} 2233 (m), 1761 (vs), 1669 (s), 1600 (m)., 1497 (m), 1452 (m), 1403 (m), 1346 9m), 1230 (vs), 1178 (m), 1013 (s).

m.p. 98 – 100 °C.

***N*-(Benzoyloxy)-4-cyano-*N*-isopropylbenzamide (4r)**

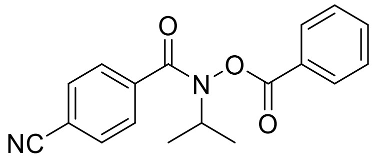

To an oven-dried Ace pressure tube, PFP (161 μL, 1.53 mmol) was added to a solution of 4-cyanobenzoic acid (0.23 g, 1.56 mmol) and DIPEA (250 μL, 1.46 mmol) in MeCN and stirred at 120 °C for 4 h. The reaction mixture was allowed to cool before adding a solution of *O*-benzoyl-*N*-isopropylhydroxylamine hydrochloride (0.20 g, 0.93 mmol), DIPEA (170 μL, 0.99 mmol) in MeCN (2 mL). The resulting solution was left to stir for 22 h at 50 °C. The reaction mixture was then concentrated under reduced pressure, diluted in DCM (15 mL), and washed with cold 0.5 M HCl (20 mL) and sat. aq. $NaHCO_3$ (20 mL). The organic layer was then dried with anhydrous $MgSO_4$, filtered and concentrated under reduced pressure. Purification by flash column chromatography (1:0 to 4:1, hexane:EtOAc) gave the target product as a white solid in 56% yield (0.16 g, 0.52 mmol).

^{1}H NMR (400 MHz, $CDCl_3$) δ_H 7.93 (dd, *J* = 7.2, 1.3, 2H), 7.75 – 7.66 (m, 2H), 7.66 – 7.58 (m, 3H), 7.51 – 7.39 (m, 2H), 4.77 (hept, *J* = 6.1, 1H), 1.32 (d, *J* = 6.1, 6H).

^{13}C{^{1}H} NMR (101 MHz, $CDCl_3$) δ_C 168.0, 164.7, 138.8, 134.6, 132.2, 129.9, 129.0, 128.2, 126.4, 118.1, 114.3, 51.8, 19.7.

LCMS (ESI$^+$) r.t = 2.7 min, *m/z* = 309 [M+H]$^+$.

HRMS (ESI$^+$) *m/z*: calcd. for $[C_{18}H_{17}N_2O_3]^+$ 309.1234, found 309.1235 [M+H]$^+$.

FTIR (neat) ν_{max} / cm^{-1} 2980 (w), 2935 (w), 2232 (m), 1765 (vs), 1667 (s), 1600 (m), 1452 (m), 1393 (m), 1316 (m), 1238 (vs), 1179 (m), 1052 (m), 1012 (s).

m.p. 112 – 115 °C.

***N*-methoxy-*N*-methyl-2-(4-(trifluoromethyl)phenyl)acetamide (4s)**

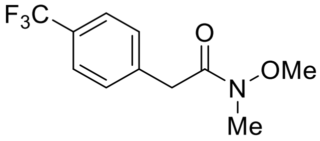

Synthesised according to general procedure B from a solution of PFP (153 µL, 1.46 mmol), 4-(trifluoromethyl)phenylacetic acid (0.30 g, 1.47 mmol) and DIPEA (250 µL, 1.46 mmol) in MeCN (3 mL) and a solution of *N*,*O*-dimethylhydroxylamine hydrochloride (0.096 g, 0.98 mmol) and DIPEA (170 mL, 0.99 mmol) in MeCN (2 mL). The crude residue was purified by flash column chromatography (1:0 to 4:1, hexane:EtOAc) to give the target product as a pale-yellow solid in 35% yield (0.083 g, 0.34 mmol).

Characterisation data was consistent with previously reported literature values.[9]

^{1}H NMR (400 MHz, $CDCl_3$) δ_H 7.57 (d, *J* = 8.0, 2H), 7.40 (d, *J* = 8.0, 2H), 3.82 (s, 2H), 3.65 (s, 3H), 3.19 (s, 3H).

^{13}C{^{1}H} NMR (101 MHz, $CDCl_3$) δ_C 171.5, 139.1, 129.9, 129.24 (q, *J* = 32.4), 125.48 (q, *J* = 3.8), 124.33 (q, *J* = 271.9), 61.5, 39.1, 32.4.

^{19}F{^{1}H} NMR (376 MHz, $CDCl_3$) δ_F -62.51.

LCMS (ESI$^+$) r.t = 2.3 min, *m/z* = 248 [M+H]$^+$.

HRMS (ESI$^+$) *m/z*: calcd. for $[C_{11}H_{13}F_3NO_2]^+$ 248.08929, found 248.09029 [M+H]$^+$.

***N*-Methoxy-*N*-methylhexanamide (4t)**

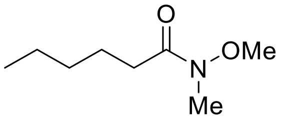

Synthesised according to general procedure B from a solution of PFP (153 µL, 1.46 mmol), hexanoic acid (182 µL, 1.45 mmol) and DIPEA (250 µL, 1.46 mmol) in MeCN (3 mL) and a solution of *N*,*O*-dimethylhydroxylamine hydrochloride (0.097 g, 0.99 mmol) and DIPEA (170 µL, 0.99 mmol) in MeCN (2 mL). The crude residue was purified by flash column

chromatography (1:0 to 4:1, hexane:EtOAc) to give the target product as a colourless oil in 70% yield (0.11 g, 0.69 mmol).

Characterisation data was consistent with previously reported literature values.[10]

^{1}H NMR (400 MHz, $CDCl_3$) δ_H 3.67 (s, 3H), 3.16 (s, 3H), 2.39 (t, J = 7.7, 2H), 1.62 (p, J = 7.5, 2H), 1.31 (h, J = 3.2, 4H), 0.92 – 0.86 (m, 3H).

^{13}C{^{1}H} NMR (101 MHz, $CDCl_3$) δ_C 175.0, 61.3, 32.3, 32.0, 31.7, 24.5, 22.6, 14.1.

LCMS (ESI$^+$) r.t = 2.0 min, *m/z* = 160 [M+H]$^+$.

HRMS (ESI$^+$) *m/z*: calcd. for $[C_8H_{18}NO_2]^+$ 160.133205, found 160.13328 [M+H]$^+$.

_N_-Methoxy-2-(6-methoxynaphthalen-2-yl)-_N_-methylpropanamide (4u)

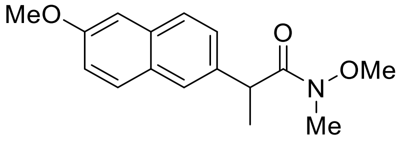

Synthesised according to general procedure B from a solution of PFP (152 µL, 1.45 mmol), 2-(6-methoxy-2-naphthyl)propionic acid (0.33 g, 1.43 mmol) and DIPEA (250 µL, 1.46 mmol) in MeCN (3 mL) and a solution of *N*,*O*-dimethylhydroxylamine hydrochloride (0.094 g, 0.96 mmol) and DIPEA (170 µL, 0.99 mmol) in MeCN (2 mL). The crude residue was purified by flash column chromatography (1:0 to 4:1, hexane:EtOAc) to give the target product as an off-white solid in 80% yield (0.21 g, 0.77 mmol).

Characterisation data was consistent with previously reported literature values.[11]

^{1}H NMR (400 MHz, $CDCl_3$) δ_H 7.70 (d, J = 8.5, 2H), 7.67 (d, J = 1.8, 1H), 7.42 (dd, J = 8.4, 1.8, 1H), 7.13 (dd, J = 8.8, 2.5, 1H), 7.10 (d, J = 2.5, 1H), 4.32 – 4.21 (m, 1H) , 3.91 (s, 3H), 3.40 (s, 3H), 3.17 (s, 3H), 1.51 (d, J = 7.1, 3H).

^{13}C{^{1}H} NMR (101 MHz, $CDCl_3$) δ_C 175.5, 157.6, 137.2, 133.6, 129.4, 129.1, 127.3, 126.7, 126.0, 119.0, 105.7, 61.3, 55.4, 42.0, 32.5, 19.8.

LCMS (ESI$^+$) r.t = 2.6 min, *m/z* = 274 [M+H]$^+$.

HRMS (ESI$^+$) *m/z*: calcd. for $[C_{16}H_{20}NO_3]^+$ 274.1443, found 274.1447 [M+H]$^+$.

***N*-Methoxy-*N*-methylbenzamide (4v)**

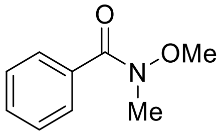

Synthesised according to general procedure B from a solution of PFP (153 μL, 1.46 mmol), benzoic acid (0.18 g, 1.47 mmol) and DIPEA (250 μL, 1.46 mmol) in MeCN (3 mL) and a solution of *N*,*O*-dimethylhydroxylamine hydrochloride (0.094 g, 0.96 mmol) and DIPEA (170 μL, 0.99 mmol) in MeCN (2 mL). The crude residue was purified by flash column chromatography (1:0 to 4:1, hexane:EtOAc) to give the target product as a colourless oil in 70% yield (0.11 g, 0.67 mmol).

Characterisation data was consistent with previously reported literature values.[12]

^{1}H NMR (400 MHz, $CDCl_3$) δ_H 7.68 – 7.64 (m, 2H), 7.48 – 7.36 (m, 3H), 3.55 (s, 3H), 3.35 (s, 3H).

$^{13}C\{^1H\}$ NMR (101 MHz, $CDCl_3$) δ_C 170.1, 134.2, 130.7, 128.3, 128.1, 61.1, 33.9.

LCMS (ESI$^+$) r.t = 1.5 min, *m/z* = 166 $[M+H]^+$.

HRMS (AI$^+$) *m/z*: calcd. for $[C_9H_{12}NO_2]^+$ 166.0868, found 166.0867 $[M+H]^+$.

4-Fluoro-*N*-methoxy-*N*-methylbenzamide (4w)

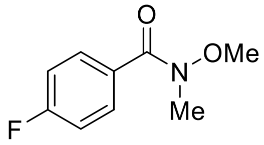

Synthesised according to general procedure B from a solution of PFP (153 μL, 1.46 mmol), 4-fluorobenzoic acid (0.21 g, 1.50 mmol) and DIPEA (250 μL, 1.46 mmol) in MeCN (3 mL) and a solution of *N*,*O*-dimethylhydroxylamine hydrochloride (0.096 g, 0.98 mmol) and DIPEA (170 μL, 0.99 mmol) in MeCN (2 mL). The crude residue was purified by flash column chromatography (1:0 to 4:1, hexane:EtOAc) to give the target product as a pale-yellow oil in 72% yield (0.13 g, 0.71 mmol).

Characterisation data was consistent with previously reported literature values.[10]

^{1}H NMR (400 MHz, $CDCl_3$) δ_H 7.78 – 7.68 (m, 2H), 7.13 – 7.02 (m, 2H), 3.53 (s, 3H), 3.35 (s, 3H).

$^{13}C\{^{1}H\}$ NMR (101 MHz, $CDCl_3$) δ_C 168.8, 165.4, 162.9, 131.0, 130.9, 130.1, 130.0, 115.3, 115.1, 61.1, 33.7.

$^{19}F\{^{1}H\}$ NMR (376 MHz, $CDCl_3$) δ_F -109.05.

LCMS (ESI^+) r.t = 1.7 min, *m/z* = 184 $[M+H]^+$.

HRMS (ESI^+) *m/z*: calcd. for $[C_9H_{11}FNO_2]^+$ 184.0768, found 184.0768 $[M+H]^+$.

***N*,4-Dimethoxy-*N*-methylbenzamide (4x)**

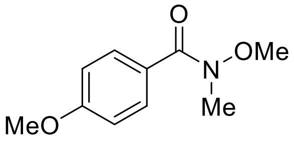

Synthesised according to general procedure B from a solution of PFP (152 µL, 1.45 mmol), 4-methoxybenzoic acid (0.22 g, 1.45 mmol) and DIPEA (250 µL, 1.46 mmol) in MeCN (3 mL) and a solution of *N*,*O*-dimethylhydroxylamine hydrochloride (0.095 g, 0.97 mmol) and DIPEA (170 µL, 0.99 mmol) in MeCN (2 mL). The crude residue was purified by flash column chromatography (1:0 to 4:1, hexane:EtOAc) to give the target product as a colourless oil in 85% yield (0.16 g, 0.82 mmol).

Characterisation data was consistent with previously reported literature values. [10]

^{1}H NMR (400 MHz, $CDCl_3$) δ_H 7.76 – 7.67 (m, 2H), 6.93 – 6.85 (m, 2H), 3.84 (s, 3H), 3.55 (s, 3H), 3.34 (s, 3H).

$^{13}C\{^{1}H\}$ NMR (101 MHz, $CDCl_3$) δ_C 169.5, 161.6, 130.7, 126.1, 113.3, 61.0, 55.4, 34.0.

LCMS (ESI^+) r.t = 1.6 min, *m/z* = 196 $[M+H]^+$.

HRMS (ESI^+) *m/z*: calcd. for $[C_{10}H_{14}NO_3]^+$ 196.0968, found 196.0968 $[M+H]^+$.

2-Iodo-*N*-methoxy-*N*-methylbenzamide (4y)

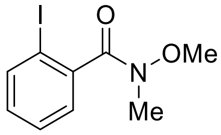

Synthesised according to general procedure B from a solution of PFP (153 µL, 1.46 mmol), 2-iodobenzoic acid (0.36 g, 1.45 mmol) and DIPEA (250 µL, 1.46 mmol) in MeCN (3 mL) and a solution of *N*,*O*-dimethylhydroxylamine hydrochloride (0.095 g, 0.97 mmol) and DIPEA (170 µL, 0.99 mmol) in MeCN (2 mL). The crude residue was purified by flash column chromatography (1:0 to 4:1, hexane:EtOAc) to give the target product as a white solid in 57% yield (0.16 g, 0.55 mmol).

Characterisation data was consistent with previously reported literature values of two rotamers in a 75:25 ratio at rt.[13]

^{1}H NMR (400 MHz, $CDCl_3$, mixture of two rotamers in an approximate 75:25 ratio) δ_H 7.83 (dd, *J* = 7.9, 1.1, 1H), 7.38 (td, *J* = 7.5, 1.1, 1H), 7.27 (dd, *J* = 7.7, 1.6, 1H), 7.09 (td, *J* = 7.7, 1.7, 1H), 3.92 (br s, 0.7H), 3.46 (s, 2.3H), 3.39 (s, 2.2H), 3.12 (br s, 0.8H).

^{13}C{^{1}H} NMR (101 MHz, $CDCl_3$) δ_C ^{13}C NMR (101 MHz, $CDCl_3$) δ_C 170.8, 141.7, 139.0, 130.40, 127.7, 127.2, 92.6, 61.5, 32.7.

LCMS (ESI$^+$) r.t. = 1.9 min, *m/z* = 292 [M+H]$^+$.

HRMS (ESI$^+$) *m/z*: calcd. for $[C_9H_{11}NO_2I]^+$ 291.9835, found 291.9849 [M+H]$^+$.

***N*-methoxy-*N*,4-dimethylbenzamide (4z)**

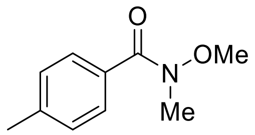

Synthesised according to general procedure B from a solution of PFP (154 µL, 1.47 mmol), *p*-toluic acid (0.20 g, 1.47 mmol) and DIPEA (250 µL, 1.46 mmol) in MeCN (3 mL) and a solution of *N*,*O*-dimethylhydroxylamine hydrochloride (0.095 g, 0.97 mmol) and DIPEA (170 µL, 0.99 mmol) in MeCN (2 mL). The crude residue was purified by flash column

chromatography (1:0 to 4:1, hexane:EtOAc) to give the target product as a colourless oil in 87% yield (0.15 g, 0.84 mmol).

Characterisation data was consistent with previously reported literature values.[12]

^{1}H NMR (400 MHz, $CDCl_3$) δ_H 7.59 (d, J = 7.8, 2H), 7.20 (d, J = 7.8, 2H), 3.56 (s, 3H), 3.35 (s, 3H), 2.38 (s, 3H).

^{13}C{^{1}H} NMR (101 MHz, $CDCl_3$) δ_C 170.1, 141.0, 131.3, 128.8, 128.5, 61.1, 34.0, 21.6.

LCMS (ESI$^+$) r.t = 1.90 min, m/z = 180 [M+H]$^+$.

HRMS (ESI$^+$) m/z: calcd. for $[C_{10}H_{14}NO_2]^+$ 180.1025, found 180.1044 [M+H]$^+$.

_N_-Methoxy-_N_-methylcyclohexanecarboxamide (4aa)

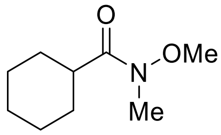

Synthesised according to general procedure B from a solution of PFP (156 µL, 1.48 mmol), cyclohexanecarboxylic acid (0.19 g, 1.48 mmol) and DIPEA (250 µL, 1.46 mmol) in MeCN (3 mL) and a solution of *N*,*O*-dimethylhydroxylamine hydrochloride (0.098 g, 1.00 mmol) and DIPEA (170 µL, 0.99 mmol) in MeCN (2 mL). The crude residue was purified by flash column chromatography (1:0 to 4:1, hexane:EtOAc) to give the target product as a colourless oil in 62% yield (0.11 g, 0.62 mmol).

Characterisation data was consistent with previously reported literature values. [14]

^{1}H NMR (400 MHz, $CDCl_3$) δ_H 3.67 (s, 3H), 3.15 (s, 3H), 2.66 (t, J = 11.0, 1H), 1.84 – 1.61 (m, 5H), 1.54 – 1.38 (m, 2H), 1.35 – 1.17 (m, 3H).

^{13}C{^{1}H} NMR (101 MHz, $CDCl_3$) δ_C 177.6, 61.6, 40.1, 32.4, 29.1, 25.9, 25.9.

LCMS (ESI$^+$) r.t = 1.9 min, m/z = 172 [M+H]$^+$.

HRMS (AI$^+$) m/z: calcd. for $[C_9H_{18}NO_2]^+$ 172.1338, found 172.1339 [M+H]$^+$.

Synthesis of hydroxamic acids (5a-l)

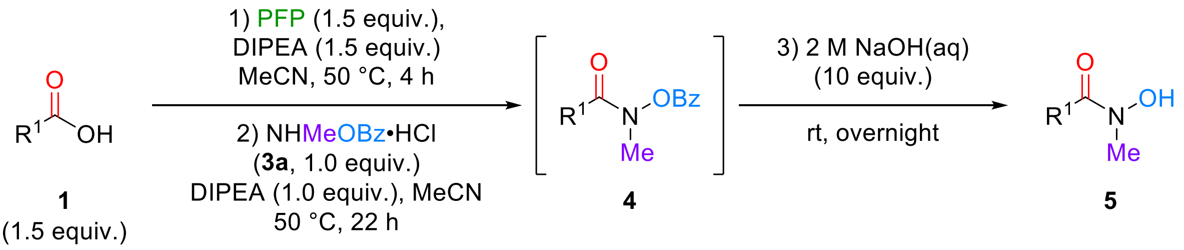

General procedure C:

To an oven-dried sealed vial or round bottom flask, PFP (1.5 equiv.) was added to a solution of carboxylic acid (1.5 equiv.) and DIPEA (1.5 equiv.) in MeCN and stirred at 50 °C for 4 h. To a separate vial or round bottom flask, a solution of hydroxylamine hydrochloride (1.0 equiv.), DIPEA (1.0 equiv.) in MeCN was prepared and subsequently added to the reaction mixture. The resulting solution was left to stir for 22 h at 50 °C. The solution was allowed to cool before adding 2 M aq. NaOH (10 equiv.) and then stirred overnight at rt. The reaction mixture was then washed with DCM (20 mL) before the addition of 0.5 M HCl (25 mL). The product was then extracted with EtOAc (3 × 20 mL) and then washed with half sat. aq. $NaHCO_3$ (2 × 20 mL). The organic layer was then dried with anhydrous Na_2SO_4 or $MgSO_4$, filtered and concentrated under reduced pressure to give the desired compounds without further purification.

General procedure D (deprotection of benzoyl-protected hydroxamic acids):

To a round bottom flask, 2 M aq. NaOH (10 equiv.) was added to a solution of a benzoyl-protected hydroxamic acid (1.0 equiv.) in THF and left to stir overnight or until complete by TLC. The resulting solution was acidified to pH ~1 with 1 M aq. HCl and extracted with EtOAc (3 × 20 mL). The combined organic layers were washed sat. aq. $NaHCO_3$ (2 × 30 mL), dried with anhydrous Na_2SO_4 or $MgSO_4$, filtered and concentrated under reduced pressure to give the desired compounds.

General procedure E (deprotection of benzoyl-protected hydroxamic acids):

To a round bottom flask, 2 M NH_3 in MeOH (10–15 equiv.) was added to a benzoyl-protected hydroxamic acid (1.0 equiv.) and left to stir overnight or until complete by TLC. The resulting solution was concentrated under reduced pressure and purified by flash column to give the desired compounds.

N-Hydroxy-N-methylbenzamide (5a)

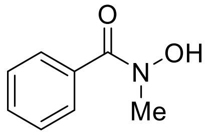

Using general procedure B: From a solution of PFP (153 μL, 1.46 mmol), benzoic acid (0.18 g, 1.47 mmol) and DIPEA (250 μL, 1.46 mmol) in MeCN (3 mL) and a solution of *N*-methylhydroxylamine hydrochloride (0.081 g, 0.97 mmol) and DIPEA (170 μL, 0.99 mmol) in MeCN (2 mL). The crude residue was purified by flash column chromatography (1:0 to 19:1, hexane:EtOAc) to give the target product as a clear oil in in 20% yield (0.028 g, 0.19 mmol).

Compound **4a** was also isolated as a side-product in 30% yield (0.074 g, 0.29 mmol).

From general procedure C: From a solution of PFP (154 μL, 1.47 mmol), benzoic acid (0.18 g, 1.47 mmol) and DIPEA (250 μL, 1.46 mmol) in MeCN (3 mL) and a solution of *N*-methyl-*O*-benzoylhydroxylamine hydrochloride (0.18 g, 0.96 mmol) and DIPEA (170 μL, 0.99 mmol) in MeCN (2 mL). Subsequent reaction with 2 M aq. NaOH (4.80 mL, 9.60 mmol) gave the target product as a brown-orange oil in 76% yield (0.11 g, 0.73 mmol).

Characterisation data was consistent with previously reported literature values.[15]

^{1}H NMR (400 MHz, $CDCl_3$) δ_H 7.57 – 7.38 (m, 5H), 3.38 (s, 3H).

^{13}C{^{1}H} NMR (101 MHz, $CDCl_3$) δ_C 167.0, 132.3, 131.1, 128.7, 128.1, 38.5.

LCMS (ESI$^+$) r.t = 1.1 min, *m/z* = 152 [M+H]$^+$.

HRMS (AI$^+$) *m/z*: calcd. for $[C_8H_{10}NO_2]^+$ 152.0712, found 152.0696 [M+H]$^+$.

N-Hydroxy-N,3,5-trimethylbenzamide (5b)

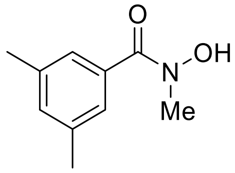

Synthesised according to general procedure C from a solution of PFP (152 μL, 1.45 mmol), 3,5-dimethylbenzoic acid (0.22 g, 1.47 mmol) and DIPEA (250 μL, 1.46 mmol) in MeCN (3 mL) and a solution of *N*-methyl-*O*-benzoylhydroxylamine hydrochloride (0.18 g, 0.96 mmol)

and DIPEA (170 µL, 0.99 mmol) in MeCN (2 mL). Subsequent reaction with 2 M aq. NaOH (4.80 mL, 9.60 mmol) gave the target product as a brown-orange oil in 93% yield (0.16 g, 0.89 mmol).

^{1}H NMR (400 MHz, $CDCl_3$) δ_H 7.11 (s, 2H), 7.09 (s, 1H), 3.38 (s, 3H), 2.33 (s, 6H).

^{13}C{^{1}H} NMR (101 MHz, $CDCl_3$) δ_C 167.3, 138.4, 132.6, 132.2, 125.7, 38.6, 21.3.

LCMS (ESI$^+$) r.t. = 1.7 min, *m/z* = 180 $[M+H]^+$.

HRMS (AI$^+$) *m/z*: calcd. for $[C_{10}H_{14}NO_2]^+$ 180.1025, found 180.1009 $[M+H]^+$.

FTIR (neat) ν_{max} / cm^{-1} 3177 (br), 2920 (br), 1586 (vs), 1482 (m), 1433 (s), 1387 (s), 1306 (m), 1201 (s), 1163 (m), 1111 (m), 1040 (w).

_N_-Hydroxy-4-methoxy-_N_-methylbenzamide (5c)

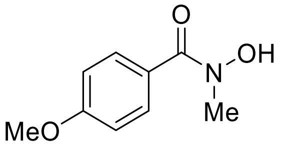

Using general procedure C: From a solution of PFP (154 µL, 1.47 mmol), 4-methoxybenzoic acid (0.22 g, 1.45 mmol) and DIPEA (250 µL, 1.46 mmol) in MeCN (3 mL) and a solution of *N*-methyl-*O*-benzoylhydroxylamine hydrochloride (0.18 g, 0.96 mmol) and DIPEA (170 µL, 0.99 mmol) in MeCN (2 mL). Subsequent reaction with 2 M aq. NaOH (4.80 mL, 9.60 mmol) gave the target product as a brown-orange oil in 57% yield (0.10 g, 0.55 mmol).

Using general procedure D: *N*-(benzoyloxy)-4-methoxy-*N*-methylbenzamide (0.11 g, 0.39 mmol, **4h**) in THF (2 mL) and 2 M aq. NaOH (1.9 mL, 3.8 mmol) gave the target product as a clear oil in 87% yield (0.061 g, 0.34 mmol).

Using general procedure E: *N*-(benzoyloxy)-4-methoxy-*N*-methylbenzamide (0.10 g, 0.35 mmol, **4h**) and 2 M NH_3 in MeOH (2.74 mL, 5.48 mmol). Purification by flash column chromatography (1:0 to 1:1, hexane:EtOAc) gave the target product as a clear oil in 97% yield (0.061 g, 0.34 mmol).

Characterisation data was consistent with previously reported literature values.[15]

^{1}H NMR (400 MHz, $CDCl_3$) δ_H 7.48 (d, J = 8.3, 2H), 6.90 (d, J = 8.3, 2H), 3.83 (s, 3H), 3.40 (s, 3H).

$^{13}C\{^1H\}$ NMR (101 MHz, $CDCl_3$) δ_C 167.3, 161.8, 130.1, 124.2, 113.9, 55.5, 39.1.

LCMS (ESI^+) r.t. = 1.2 min, m/z = 182 $[M+H]^+$.

HRMS (ESI^+) m/z: calcd. for $[C_9H_{12}NO_3]^+$ 182.0817, found 182.0821 $[M+H]^+$.

2-Chloro-*N*-hydroxy-*N*-methylbenzamide (5d)

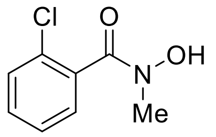

Synthesised according to general procedure C from a solution of PFP (154 µL, 1.47 mmol), 2-chlorobenzoic acid (0.23 g, 1.47 mmol) and DIPEA (250 µL, 1.46 mmol) in MeCN (3 mL) and a solution of *N*-methyl-*O*-benzoylhydroxylamine hydrochloride (0.18 g, 0.96 mmol) and DIPEA (170 µL, 0.99 mmol) in MeCN (2 mL). Subsequent reaction with 2 M aq. NaOH (4.80 mL, 9.60 mmol) gave the target product as an off-white solid in 53% yield (0.094 g, 0.51 mmol).

^{1}H NMR (400 MHz, $CDCl_3$) δ_H 8.51 (app. br s, 1H), 7.42 – 7.26 (m, 4H), 3.18 (s, 3H).

$^{13}C\{^1H\}$ NMR (101 MHz, $CDCl_3$) δ_C 163.2, 133.0, 131.4, 131.2, 129.9, 128.9, 127.3, 37.9.

LCMS (ESI^+) r.t. = 1.2 min, m/z = 186 $[M+H]^+$.

HRMS (AI^+) m/z: calcd. for $[C_8H_9NO_2Cl]^+$ 186.0322, found 186.0320 $[M+H]^+$.

FTIR (neat) ν_{max} / cm^{-1} 3065 (br), 2843 (br), 1592 (s), 1482 (s), 1432 (vs), 1385 (s), 1298 (m), 1269 (m), 1216 (m), 1190 (m), 1114 (m), 1086 (m), 1047 (m).

m.p. 74 – 80 °C.

***N*-Hydroxy-*N*,4-dimethylbenzamide (5e)**

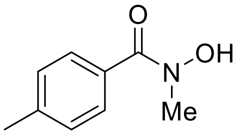

Synthesised according to general procedure C from a solution of PFP (155 μL, 1.48 mmol), *p*-toluic acid (0.20 g, 1.47 mmol) and DIPEA (250 μL, 1.46 mmol) in MeCN (3 mL) and a solution of *N*-methyl-*O*-benzoylhydroxylamine hydrochloride (0.18 g, 0.96 mmol) and DIPEA (170 μL, 0.99 mmol) in MeCN (2 mL). Subsequent reaction with 2 M aq. NaOH (4.80 mL, 9.60 mmol) gave the target product as a brown-orange oil 70% yield (0.11 g, 0.67 mmol).

Characterisation data was consistent with previously reported literature values.[15]

^{1}H NMR (400 MHz, $CDCl_3$) δ_H 7.39 (d, *J* = 7.3, 2H), 7.19 (d, *J* = 7.3, 2H), 3.36 (s, 3H), 2.37 (s, 3H).

^{13}C{^{1}H} NMR (101 MHz, $CDCl_3$) δ_C 167.4, 141.4, 129.4, 129.2, 128.1, 38.9, 21.6.

LCMS (ESI$^+$) r.t. = 1.4 min, *m/z* = 166 [M+H]$^+$.

HRMS (AI$^+$) *m/z*: calcd. for $[C_9H_{12}NO_2]^+$ 166.0868, found 166.0860 [M+H]$^+$.

***N*-Hydroxy-*N*-methylcyclohexanecarboxamide (5f)**

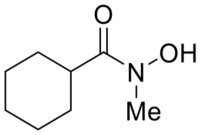

Synthesised according to general procedure C from a solution of PFP (155 μL, 1.48 mmol), cyclohexanecarboxylic acid (0.19 g, 1.48 mmol) and DIPEA (250 μL, 1.46 mmol) in MeCN (3 mL) and a solution of *N*-methyl-*O*-benzoylhydroxylamine hydrochloride (0.18 g, 0.96 mmol) and DIPEA (170 μL, 0.99 mmol) in MeCN (2 mL). Subsequent reaction with 2 M aq. NaOH (4.80 mL, 9.60 mmol) gave the target product as an off-white solid in 73% yield (0.11 g, 0.70 mmol).

^{1}H NMR (700 MHz, MeOD) δ_H 3.18 (s, 3H), 2.93 (t, *J* = 11.3, 1H), 1.82 – 1.73 (m, 4H), 1.73 – 1.66 (m, 1H), 1.44 – 1.19 (m, 5H).

^{13}C{^{1}H} NMR (176 MHz, MeOD) δ_C 178.6, 40.8, 36.4, 29.8, 27.0, 26.8.

LCMS (ESI^+) r.t. = 1.5 min, *m/z* = 158 $[M+H]^+$.

HRMS (AI^+) *m/z*: calcd. for $[C_8H_{16}NO_2]^+$ 158.1181, found 158.1186 $[M+H]^+$.

FTIR (neat) ν_{max} / cm^{-1} 2933 (m), 2855 (m), 2762 (br), 1571 (s), 1448 (vs), 1389 (m), 1297 (m), 1193 (s), 1096 (m).

m.p. 84 – 88 °C.

_N_-Hydroxy-_N_-methylcinnamamide (5g)

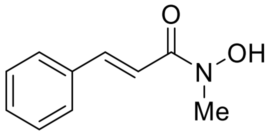

Synthesised according to general procedure C from a solution of PFP (152 μL, 1.45 mmol), cinnamic acid (0.22 g, 1.48 mmol) and DIPEA (250 μL, 1.46 mmol) in MeCN (3 mL) and a solution of *N*-methyl-*O*-benzoylhydroxylamine hydrochloride (0.18 g, 0.96 mmol) and DIPEA (170 μL, 0.99 mmol) in MeCN (2 mL). Subsequent reaction with 2 M aq. NaOH (4.80 mL, 9.60 mmol) gave the target product as an off-white solid in 76% yield (0.13 g, 0.73 mmol).

^{1}H NMR (700 MHz, MeOD) δ_H 7.59 – 7.54 (m, 3H), 7.39 – 7.33 (m, 3H), 7.33 – 7.28 (m, 1H), 3.32 (s, 3H).

$^{13}C\{^1H\}$ NMR (101 MHz, MeOD) δ_C 168.4, 143.5, 136.4, 130.9, 129.9, 128.9, 117.3, 36.6.

LCMS (ESI^+) r.t. = 1.4 min, *m/z* = 166 $[M+H]^+$.

HRMS (AI^+) *m/z*: calcd. for $[C_{10}H_{12}NO_2]^+$ 178.0868, found 178.0854 $[M+H]^+$.

FTIR (neat) ν_{max} / cm^{-1} 2806 (br), 2255 (br), 1636 (s), 1568 (s), 1485 (m), 1450 (m), 1424 (m), 1391 (s), 1296 (m), 1192 (m), 1108 (m), 1074 (m).

m.p. 114 – 117 °C.

4-Ethynyl-*N*-hydroxy-*N*-methylbenzamide (5h)

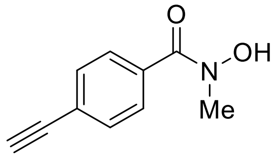

Synthesised according to general procedure D from a solution of *N*-(benzoyloxy)-4-ethynyl-*N*-methylbenzamide (0.21 g, 0.75 mmol, **4j**) and 2 M aq. NaOH (3.70 mL, 7.40 mmol) in THF (15 mL), stirred for 47 h which gave the target product as an off-white solid in 84% yield (0.11 g, 0.63 mmol).

^{1}H NMR (400 MHz, MeOD) δ_H 7.62 (d, J = 8.2, 2H), 7.51 (d, J = 8.2, 2H), 3.62 (s, 1H), 3.36 (s, 3H).

^{13}C{^{1}H} NMR (176 MHz, MeOD) δ_C 170.7, 135.8, 132.5, 129.5, 125.9, 83.7, 80.5, 37.5.

LCMS (ESI$^+$) r.t. = 1.4 min, *m/z* = 176 [M+H]$^+$.

HRMS (AI$^+$) *m/z*: calcd. for $[C_{10}H_{10}NO_2]^+$ 176.0712, found 176.0706 [M+H]$^+$.

FTIR (neat) ν_{max} / cm^{-1} 3283 (m), 2808 (br), 1578 (s), 1542 (s), 1467 (s), 1431 (s), 1389 (s), 1212 (s), 1182 (m), 1072 (m), 1019 (m).

m.p. 108 – 110 °C.

Synthesis of other compounds (6-10)

Benzyl azide (6)

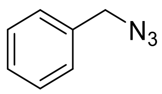

Synthesised according to a modified literature procedure.[16] To a solution of benzyl bromide (0.55 mL, 4.62 mmol) in acetone/H_2O (130 mL, 3/1 v/v) was added sodium azide (0.45 g, 6.92 mmol) and left to stir for 29 h at rt. Et_2O (30 mL) was added and the layers were separated. The aqueous layer was further extracted with Et_2O (3 × 30 mL) to which the combined organic layers were washed with brine (3 × 100 mL). The organic phase was dried with anhydrous

$MgSO_4$, filtered and concentrated under reduced pressure to give the target product as a yellow oil in 89% yield (0.55 g, 4.13 mmol).

Characterisation data was consistent with previously reported literature values.[17]

^{1}H NMR (400 MHz, $CDCl_3$) δ_H 7.43 – 7.31 (m, 5H), 4.35 (s, 2H).

^{13}C{^{1}H} NMR (101 MHz, $CDCl_3$) δ_C 135.5, 129.0, 128.4, 128.4, 54.9.

GCMS (EI$^+$) r.t. = 3.3 min, *m/z* = 133 [M+H]$^+$.

N-(Benzoyloxy)-4-(1-benzyl-1*H*-1,2,3-triazol-4-yl)-*N*-methylbenzamide (7)

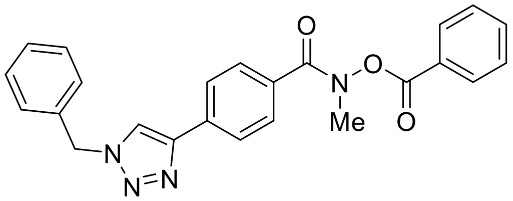

A solution of *N*-(benzoyloxy)-4-ethynyl-*N*-methylbenzamide (0.21 g, 0.75 mmol, **4j**), benzyl azide (0.10 g, 0.75 mmol, **6**), copper(II) sulfate pentahydrate (0.031 g, 0.12 mmol) and sodium L-ascorbate (0.038 g, 0.19 mmol) in MeOH (25 mL) was left to stir at rt for 20 h. The solution was then concentrated under reduced pressure and diluted in H_2O (30 mL) and EtOAc (30 mL). The layers were separated and further extract with EtOAc (3 × 30 mL). The combined organic layer was dried with anhydrous $MgSO_4$, filtered and concentrated under reduced pressure. Purification by flash column chromatography (1:0 to 7:3, hexane:EtOAc) gave the target product as an off-white solid in 71% yield (0.22 g, 0.53 mmol).

^{1}H NMR (400 MHz, $CDCl_3$) δ_H 7.91 (d, *J* = 7.3, 2H), 7.77 (d, *J* = 8.0, 2H), 7.69 (d, *J* = 8.0, 2H), 7.66 (s, 1H), 7.58 (t, *J* = 7.5, 1H), 7.41 (app. t, *J* = 7.8, 2H), 7.38 – 7.35 (m, 3H), 7.31 – 7.27 (m, 2H), 5.55 (s, 2H), 3.52 (s, 3H).

^{13}C{^{1}H} NMR (176 MHz, $CDCl_3$) δ_C 171.1, 164.5, 147.3, 134.5, 134.4, 133.2, 132.8, 130.0, 129.3, 129.0, 128.9, 128.8, 128.2, 126.8, 125.4, 120.3, 54.5, 37.4.

LCMS (ESI$^+$) r.t. = 2.7 min, *m/z* = 413 [M+H]$^+$.

HRMS (ESI$^+$) *m/z*: calcd. for $[C_{24}H_{21}N_4O_3]^+$ 413.1614, found 413.1601 [M+H]$^+$.

FTIR (neat) ν_{max} / cm^{-1} 1764 (s), 1656 (s), 1616 (w), 1452 (m), 1437 (m), 1350 (m), 1255 (m), 1220 (m), 1156 (m), 1065 (m), 1047 (m), 1034 (m), 1012 (s).

m.p. 156 – 157 °C.

4-(1-Benzyl-1*H*-1,2,3-triazol-4-yl)-*N*-hydroxy-*N*-methylbenzamide (8)

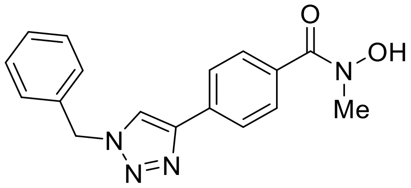

Synthesised according to general procedure D from a solution of *N*-(benzoyloxy)-4-(1-benzyl-1*H*-1,2,3-triazol-4-yl)-*N*-methylbenzamide (0.089 g, 0.22 mmol, **7**) and 2 M aq. NaOH (1.10 mL, 2.20 mmol) in THF (5 mL), stirred for 47 h which gave the target product as an light-brown solid in 73% yield (0.048 g, 0.16 mmol).

^{1}H NMR (700 MHz, MeOD) δ_H 8.38 (s, 1H), 7.86 (d, *J* = 7.7, 2H), 7.70 (app. br s, 2H), 7.40 – 7.32 (m, 5H), 5.63 (s, 2H), 3.37 (s, 3H).

^{13}C{^{1}H} NMR (101 MHz, MeOD) δ_C 170.9, 148.3, 136.7, 135.2, 133.7, 130.1, 130.1, 129.6, 129.1, 126.1, 122.9, 55.1, 38.0.

LCMS (ESI^{+}) r.t. = 1.8 min, *m/z* = 309 [M+H]$^{+}$.

HRMS (ESI^{+}) *m/z*: calcd. for $[C_{17}H_{17}N_4O_2]^+$ 309.1352, found 309.1364 [M+H]$^{+}$.

FTIR (neat) ν_{max} / cm^{-1} 2319 (br), 1970 (w), 1758 (vs), 1655 (m), 1599 (m), 1451 (m), 1390 (m), 1304 (m), 1240 (s), 1051 (m).

m.p. 140 – 144 °C.

Oxonane-2,9-dione (9)

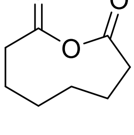

Synthesised according to a literature procedure.[18] Suberic acid (2.49 g, 14.3 mmol) was refluxed in acetic anhydride (5.00 mL) for 2 h. The resulting mixture was concentrated under reduced pressure using toluene to from a lower boiling point azeotrope. The residue was then

purified by recrystallisation in MeCN to give the target product as a cream solid in 54% yield (1.21 g, 7.75 mmol).

Characterisation data was consistent with previously reported literature values.[19]

^{1}H NMR (400 MHz, $CDCl_3$) δ_H 2.44 (t, J = 7.4, 4H), 1.72 – 1.61 (m, 4H), 1.43 – 1.33 (m, 4H).

$^{13}C\{^1H\}$ NMR (101 MHz, $CDCl_3$) δ_C 169.5, 35.2, 28.5, 24.0.

LCMS (ESI$^+$) r.t. = 2.7 min, *m/z* = 157 [M+H]$^+$.

HRMS (ESI$^+$) *m/z*: calcd. for $[C_8H_{13}O_3]^+$ 157.0865, found 157.0858 [M+H]$^+$.

N^1-(Benzoyloxy)-N^1-methyl-N^8-phenyloctanediamide (10)

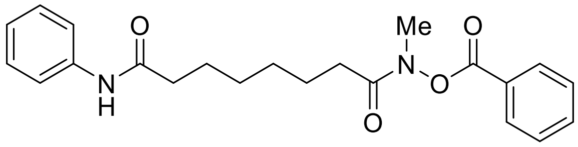

In an oven-dried sealed vial, a solution of oxonane-2,9-dione (0.15 g, 0.96 mmol, **9**) and aniline (85 µl, 0.93 mmol) in DMF (2 mL) were stirred at 40 °C for 2 h. Subsequently, pentafluoropyridine (98 µL, 0.93 mmol) and *N*,*N*-diisopropylethylamine (160 µl, 0.93 mmol) were added and then stirred for a further 4 h at 50 °C. Lastly, a solution of *N*-methyl-*O*-benzoylhydroxylamine hydrochloride (0.12 g, 0.64 mmol) and *N*,*N*-diisopropylethylamine (110 µl, 0.64 mmol) in DMF (1 mL) was added and left to stir for 22 h at 50 °C. The reaction mixture was then concentrated under reduced pressure, diluted in DCM (15 mL), and sequentially washed with cold 0.5 M HCl (20 mL) and sat. aq. $NaHCO_3$ (20 mL). The organic layer was then dried with anhydrous $MgSO_4$, filtered and concentrated under reduced pressure. The residue was then purified by flash column chromatography (1:0 to 1:1, hexane:EtOAc) to give the target product as a pale-orange oil in 39% yield (0.095 g, 0.25 mmol).

^{1}H NMR (400 MHz, $CDCl_3$) δ_H 8.08 (d, J = 7.7, 2H), 7.67 (t, J = 7.5, 1H), 7.63 – 7.56 (m, 1H), 7.55 – 7.47 (m, 4H), 7.28 (t, J = 8.0, 2H), 7.07 (t, J = 7.4, 1H), 3.40 (s, 3H), 2.38 – 2.25 (m, 4H), 1.70 (t, J = 7.1, 2H), 1.65 (t, J = 6.9, 2H), 1.44 – 1.29 (m, 4H).

$^{13}C\{^1H\}$ NMR (176 MHz, $CDCl_3$) δ_C 174.2, 171.7, 164.5, 138.3, 134.6, 130.1, 129.0, 129.0, 126.8, 124.1, 119.9, 37.5, 35.6, 32.1, 28.8, 28.7, 25.4, 24.1.

LCMS (ESI$^+$) r.t. = 2.6 min, *m/z* = 383 [M+H]$^+$.

HRMS (ESI$^+$) *m/z*: calcd. for $[C_{22}H_{27}N_2O_4]^+$ 383.1965, found 383.1975 $[M+H]^+$.

FTIR (neat) ν_{max} / cm^{-1} 3322 (br), 2930 (m), 2855 (w), 2342 (w), 1761 (s), 1659 (s), 1599 (s), 1541 (s), 1498 (m), 1441 (s), 1385 (m), 1309 (m), 1242 (s), 1175 (m), 1036 (m), 1009 (s).

NMR data for synthesised compounds

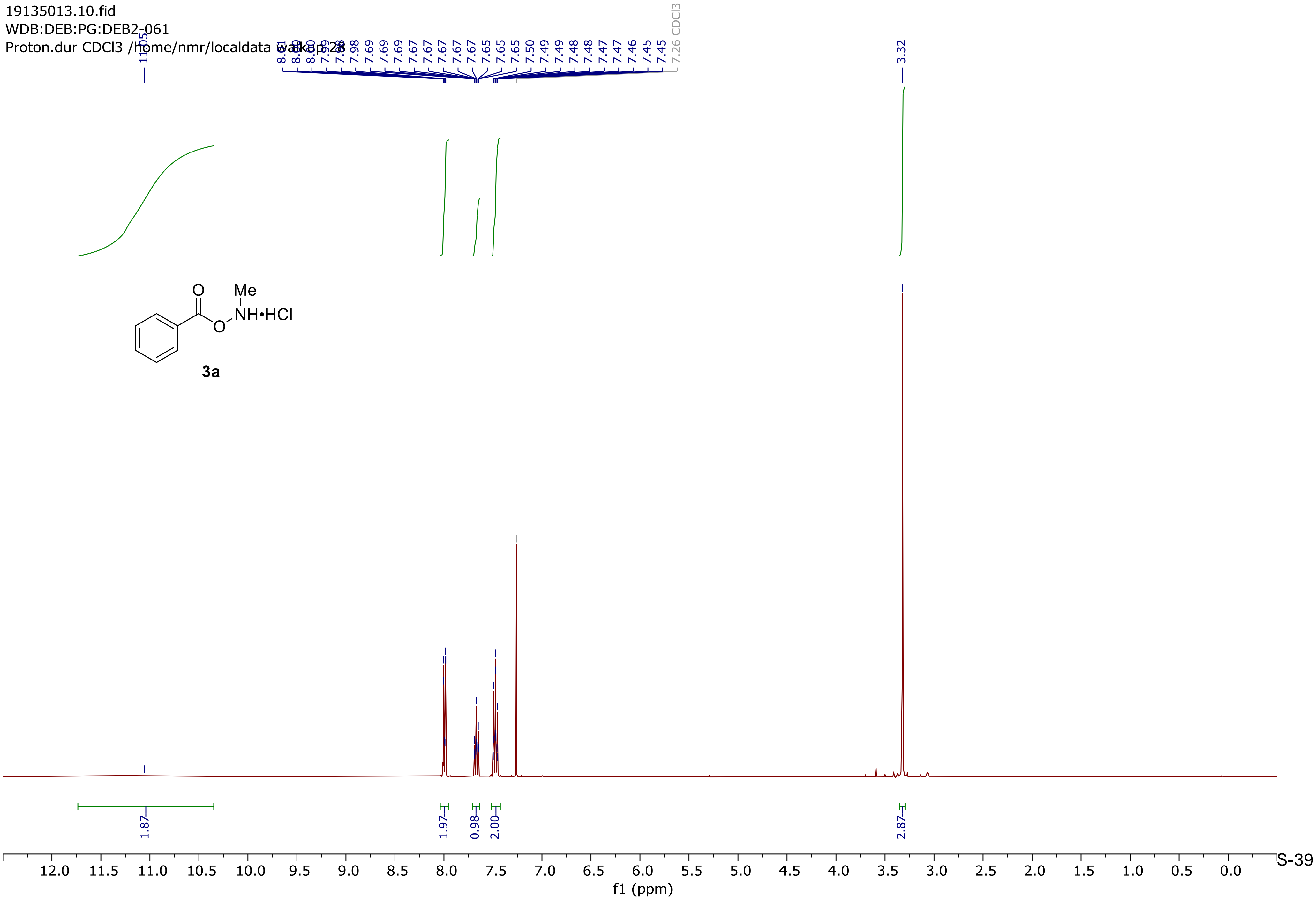

Figure S1; ^{1}H NMR (400 MHz, $CDCl_3$) for compound **3a**.

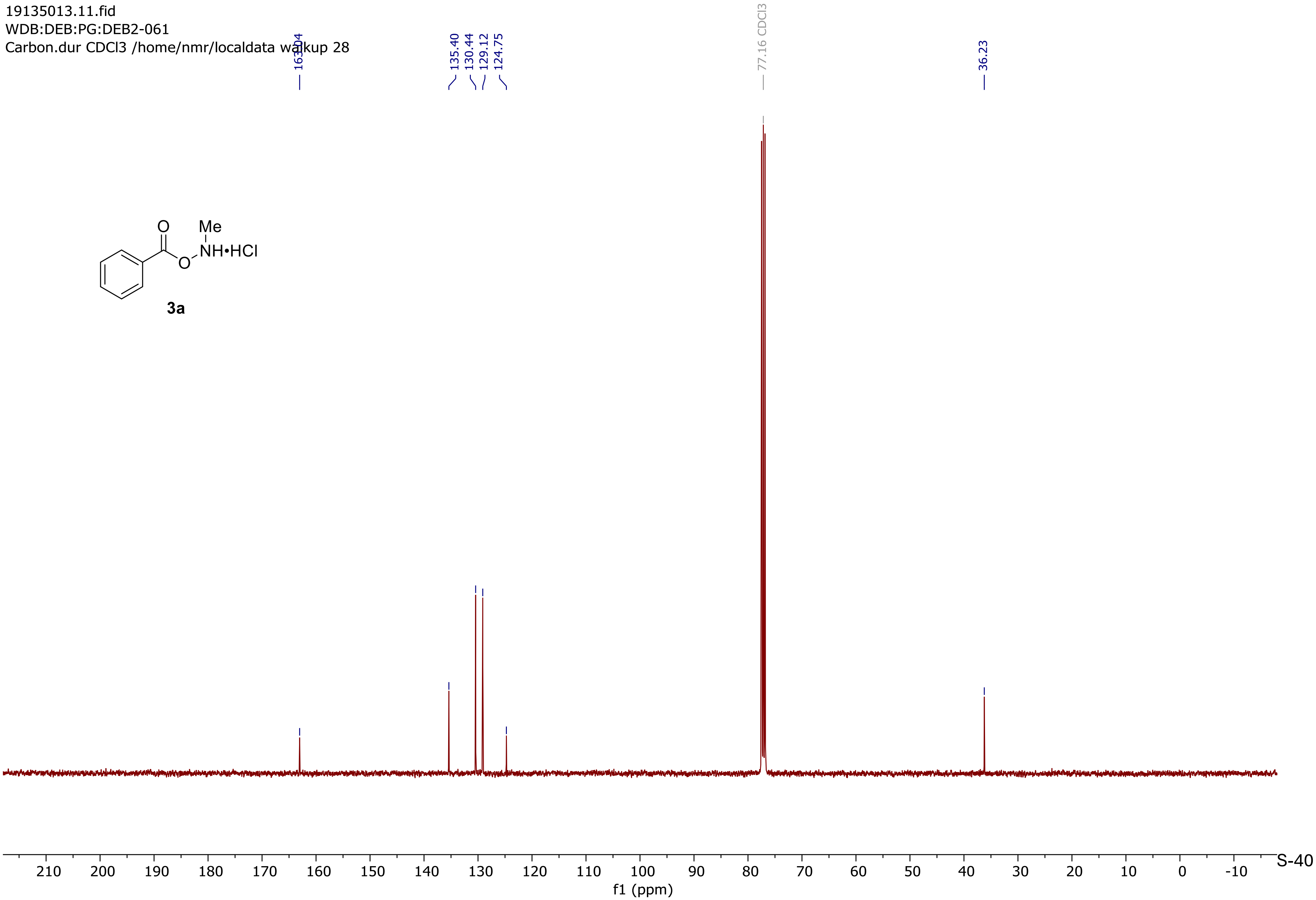

Figure S2; $^{13}C\{^{1}H\}$ NMR (101 MHz, $CDCl_3$) for compound **3a**.

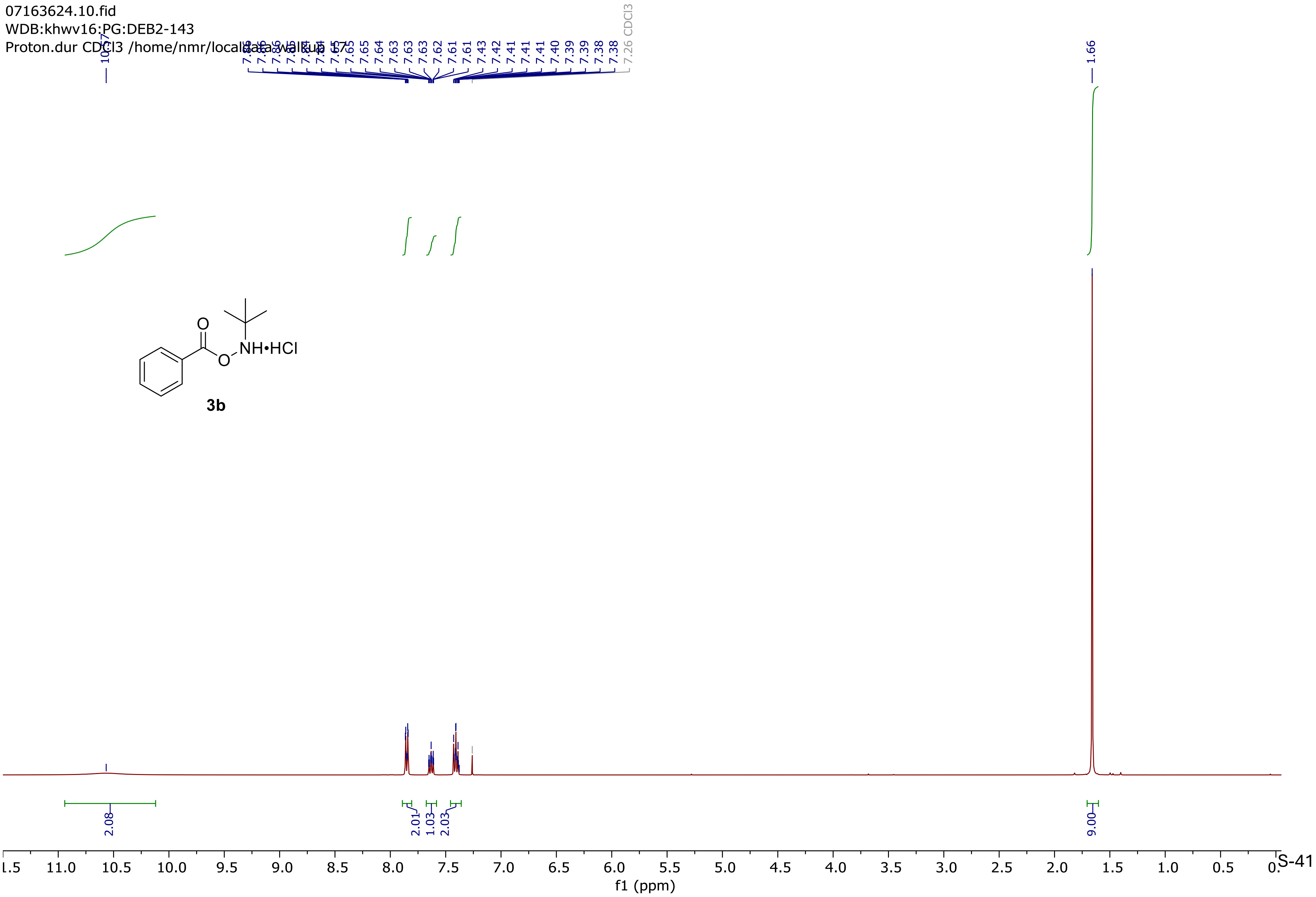

Figure S3; ^{1}H NMR (400 MHz, $CDCl_3$) for compound **3b**.

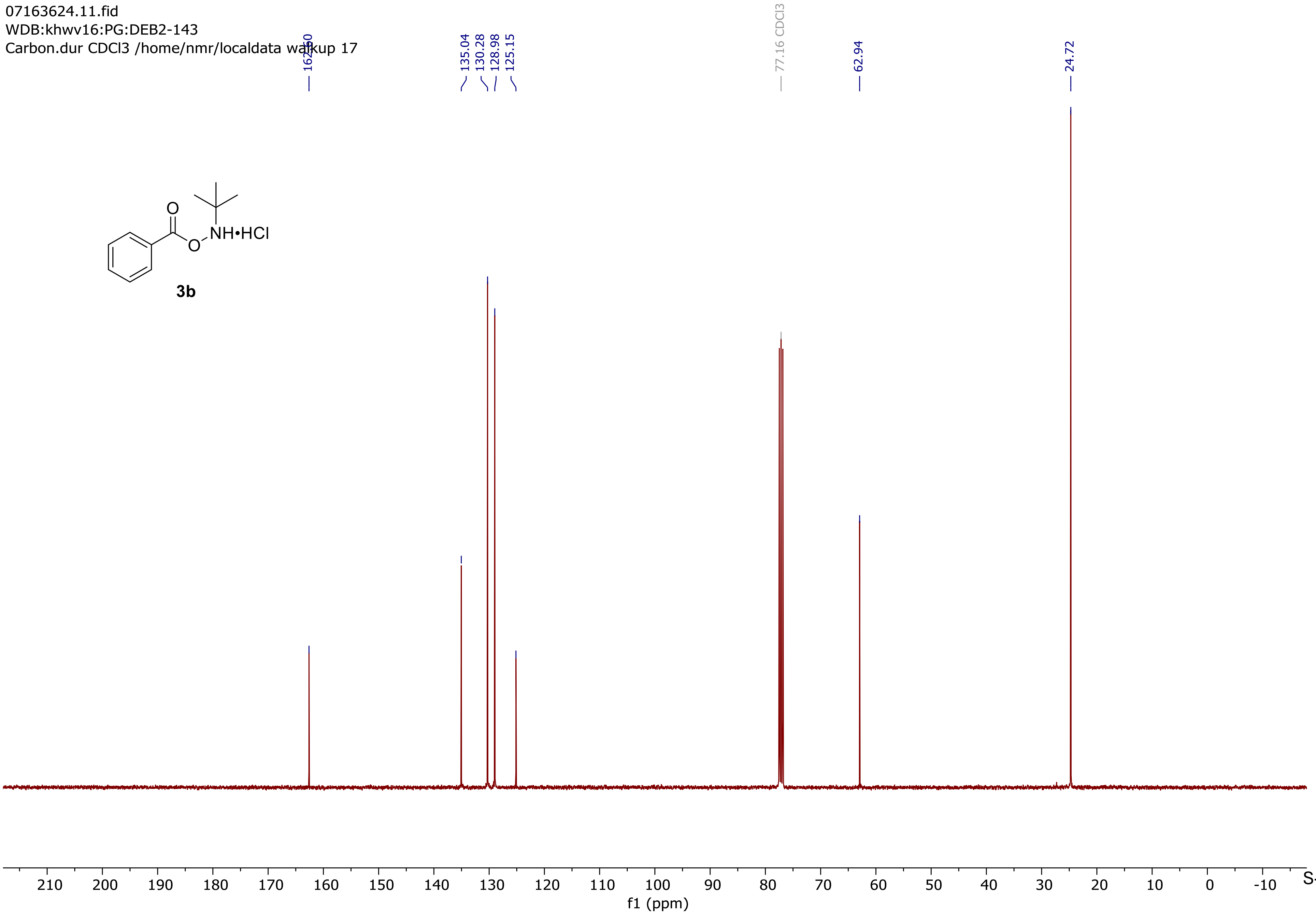

Figure S4; $^{13}C\{^{1}H\}$ NMR (101 MHz, $CDCl_3$) for compound **3b**.

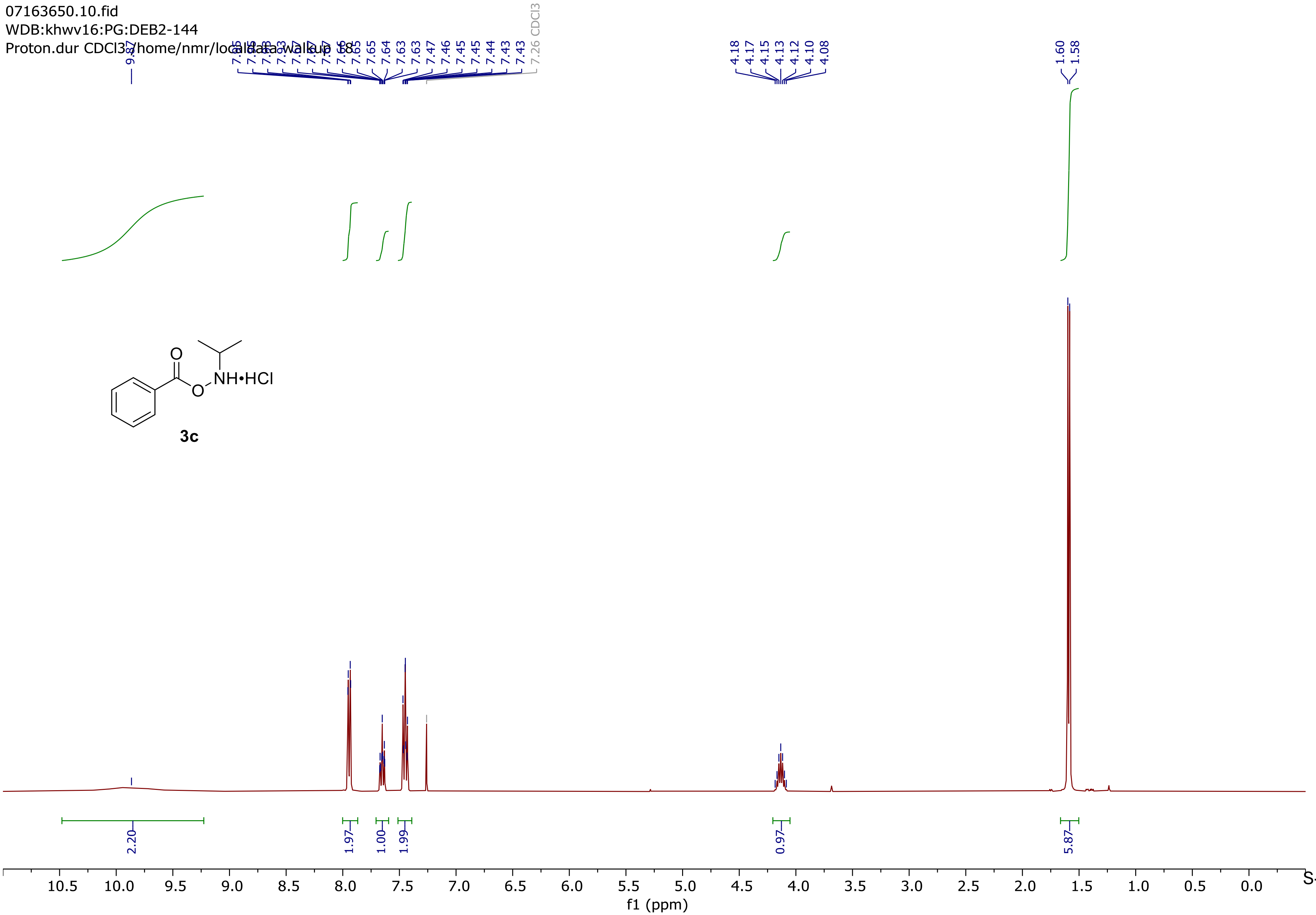

Figure S5; ^{1}H NMR (400 MHz, $CDCl_3$) for compound **3c**.

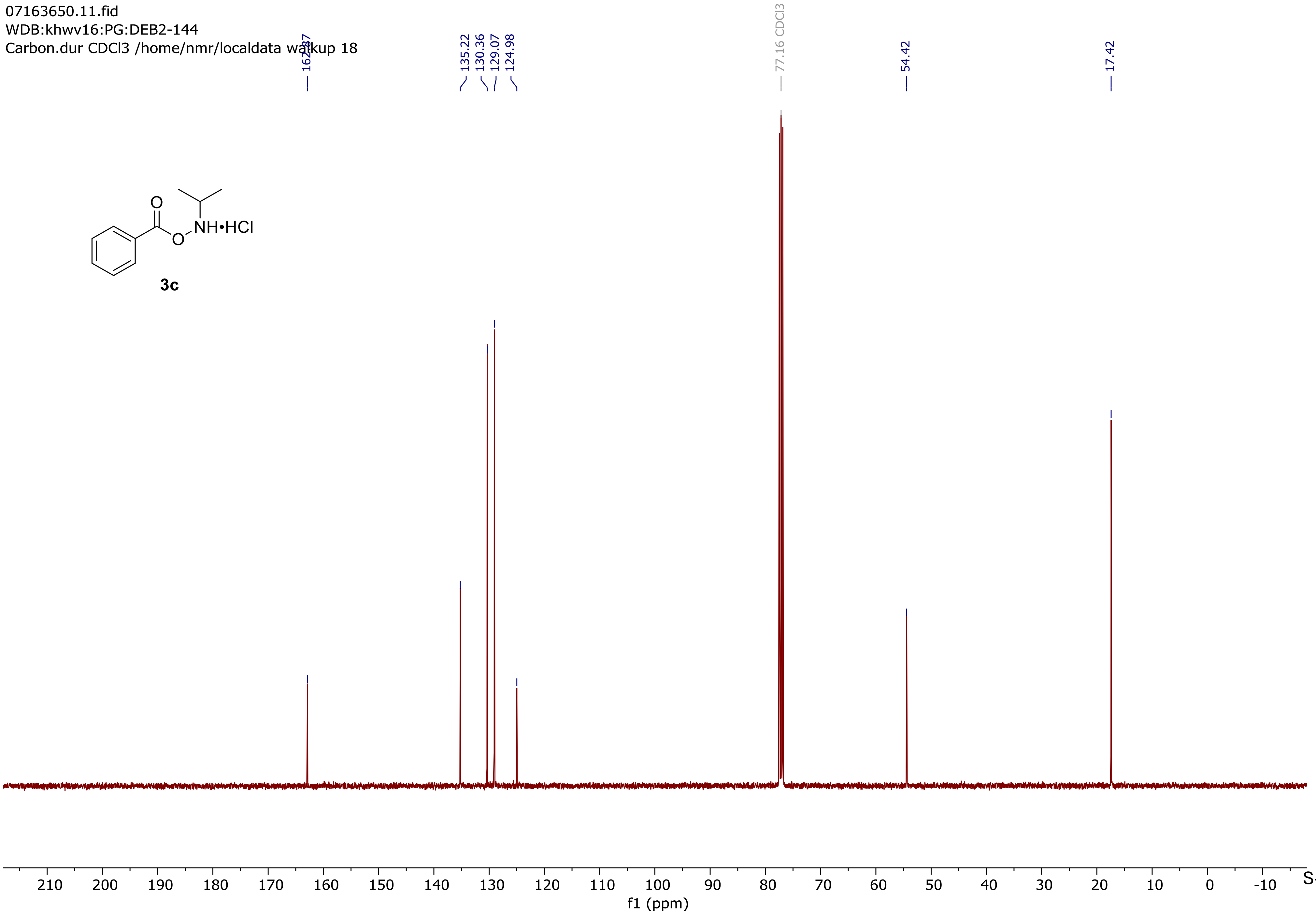

Figure S6; $^{13}C\{^{1}H\}$ NMR (101 MHz, $CDCl_3$) for compound **3c**.

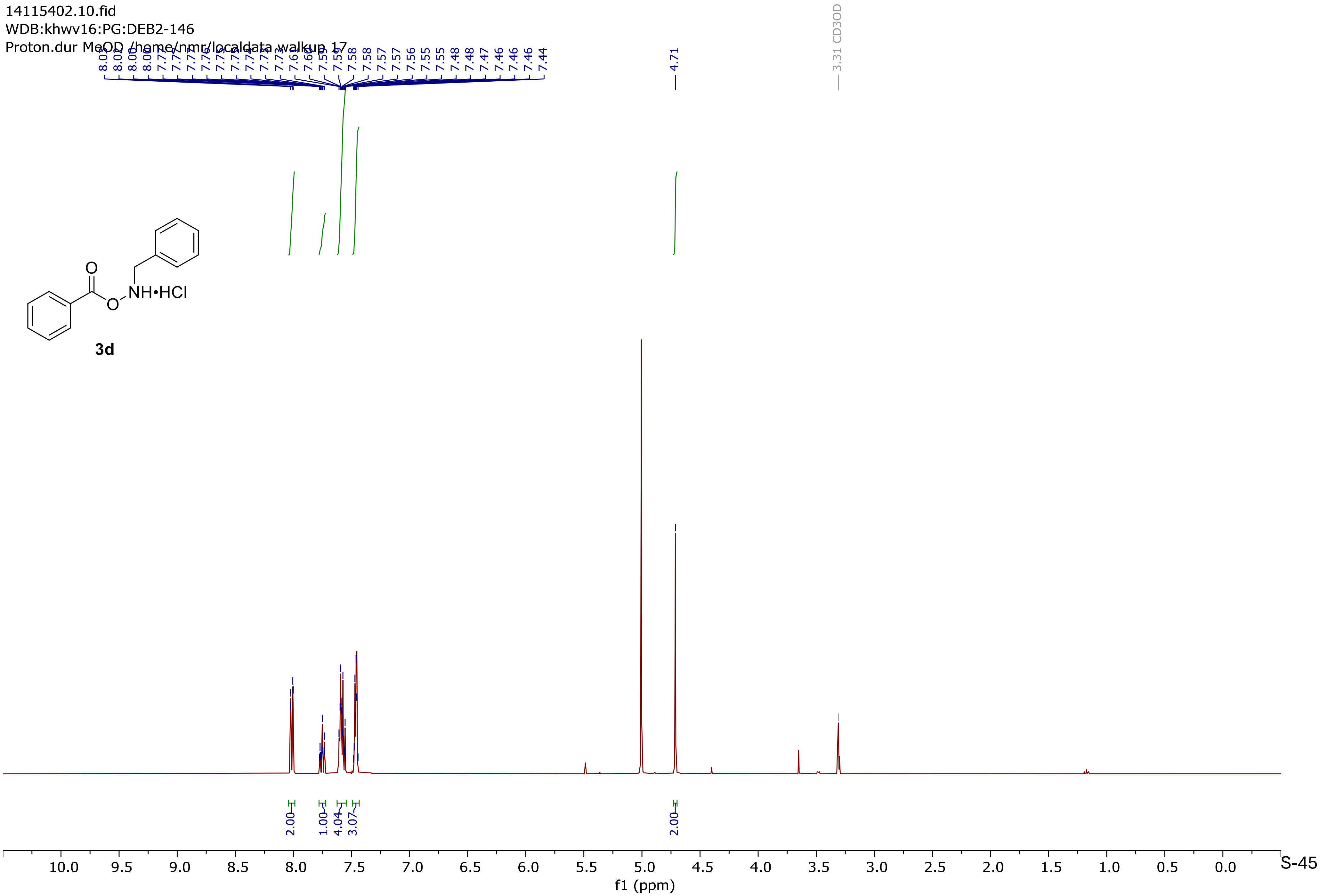

Figure S7; ^{1}H NMR (400 MHz, MeOD) for compound **3d**.

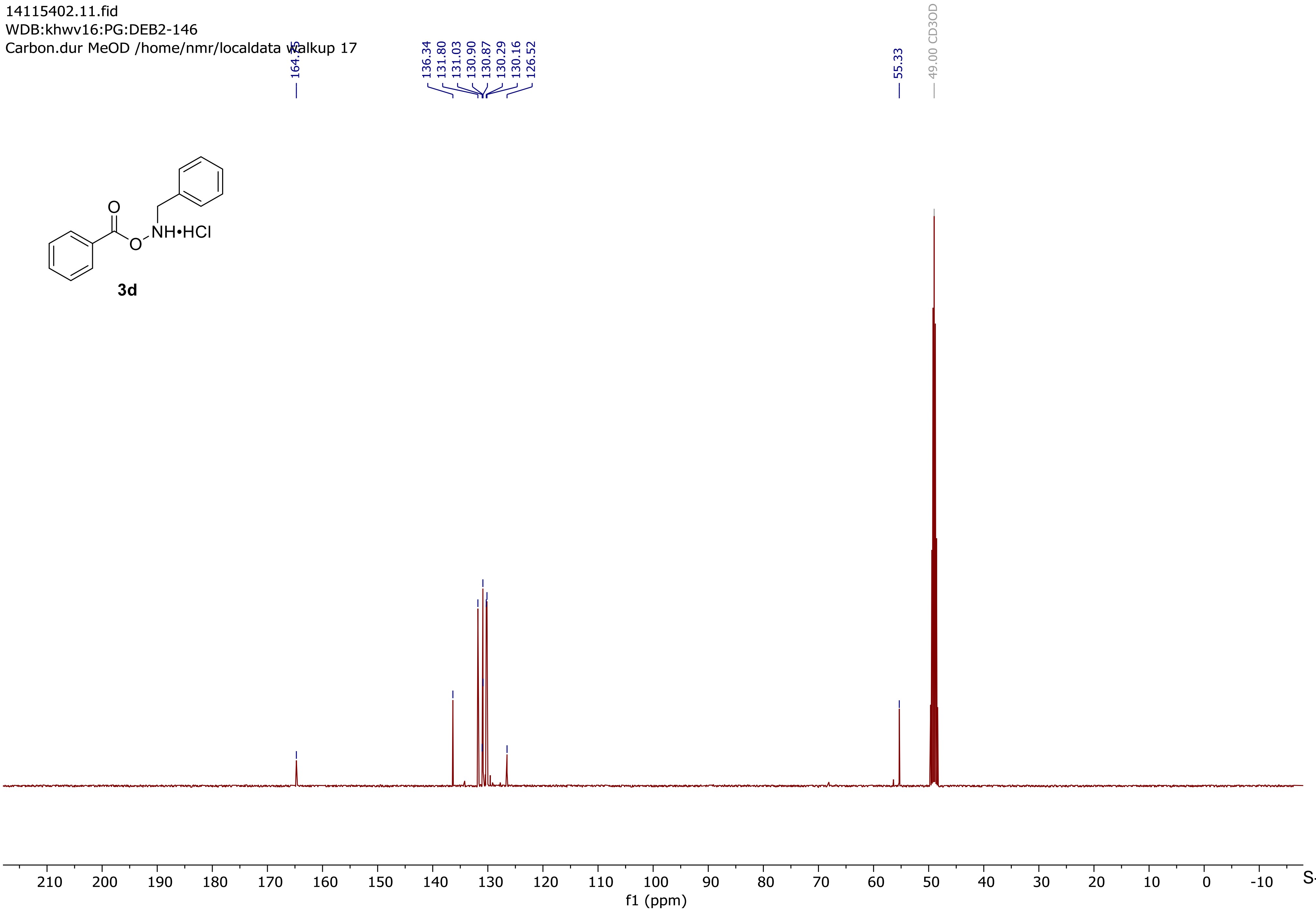

Figure S8; $^{13}C\{^{1}H\}$ NMR (101 MHz, MeOD) for compound **3d**.

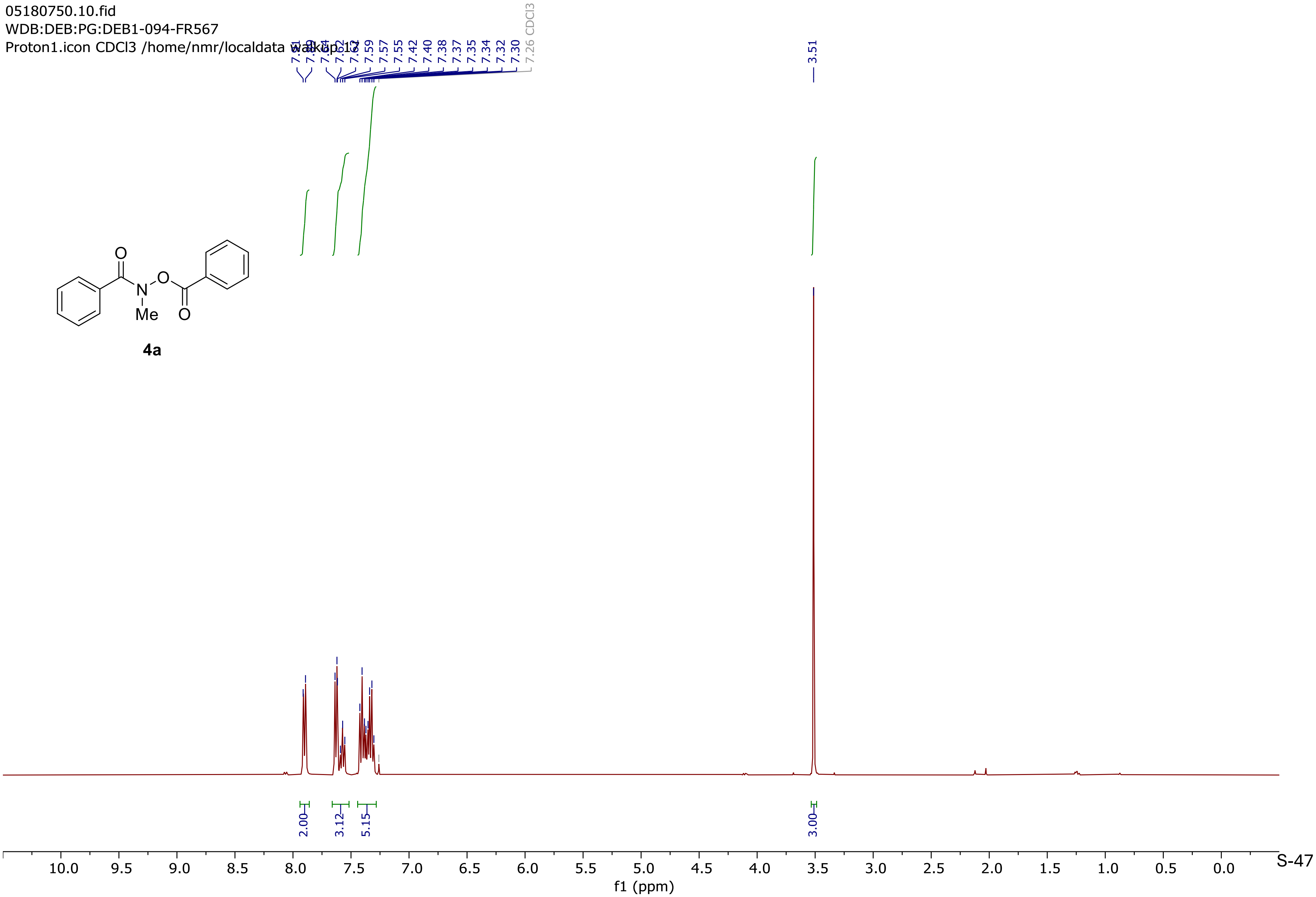

Figure S9; ^{1}H NMR (400 MHz, $CDCl_3$) for compound **4a**.

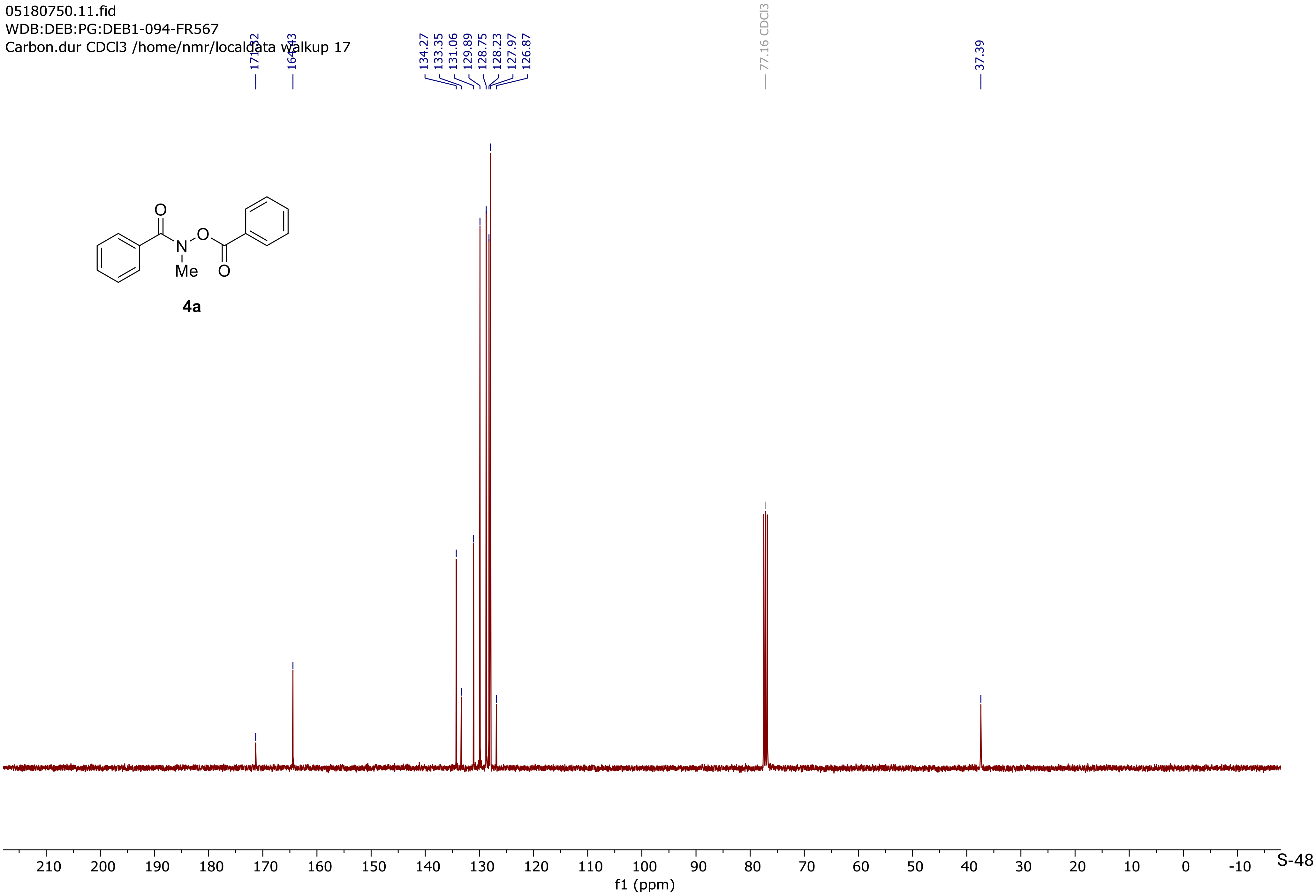

Figure S10; $^{13}C\{^{1}H\}$ NMR (101 MHz, $CDCl_3$) for compound **4a**.

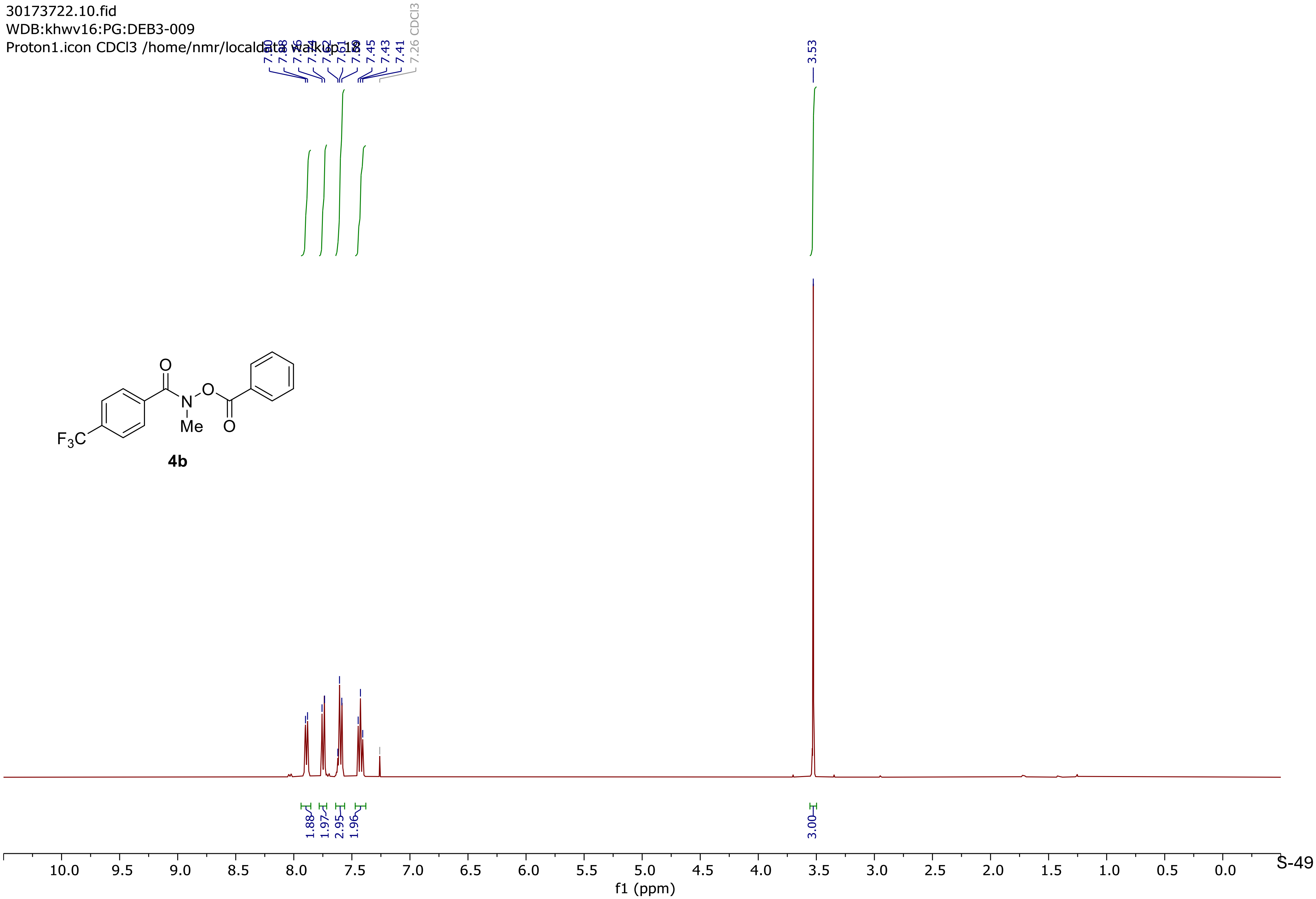

4b

Figure S11; ^{1}H NMR (400 MHz, $CDCl_3$) for compound **4b**.

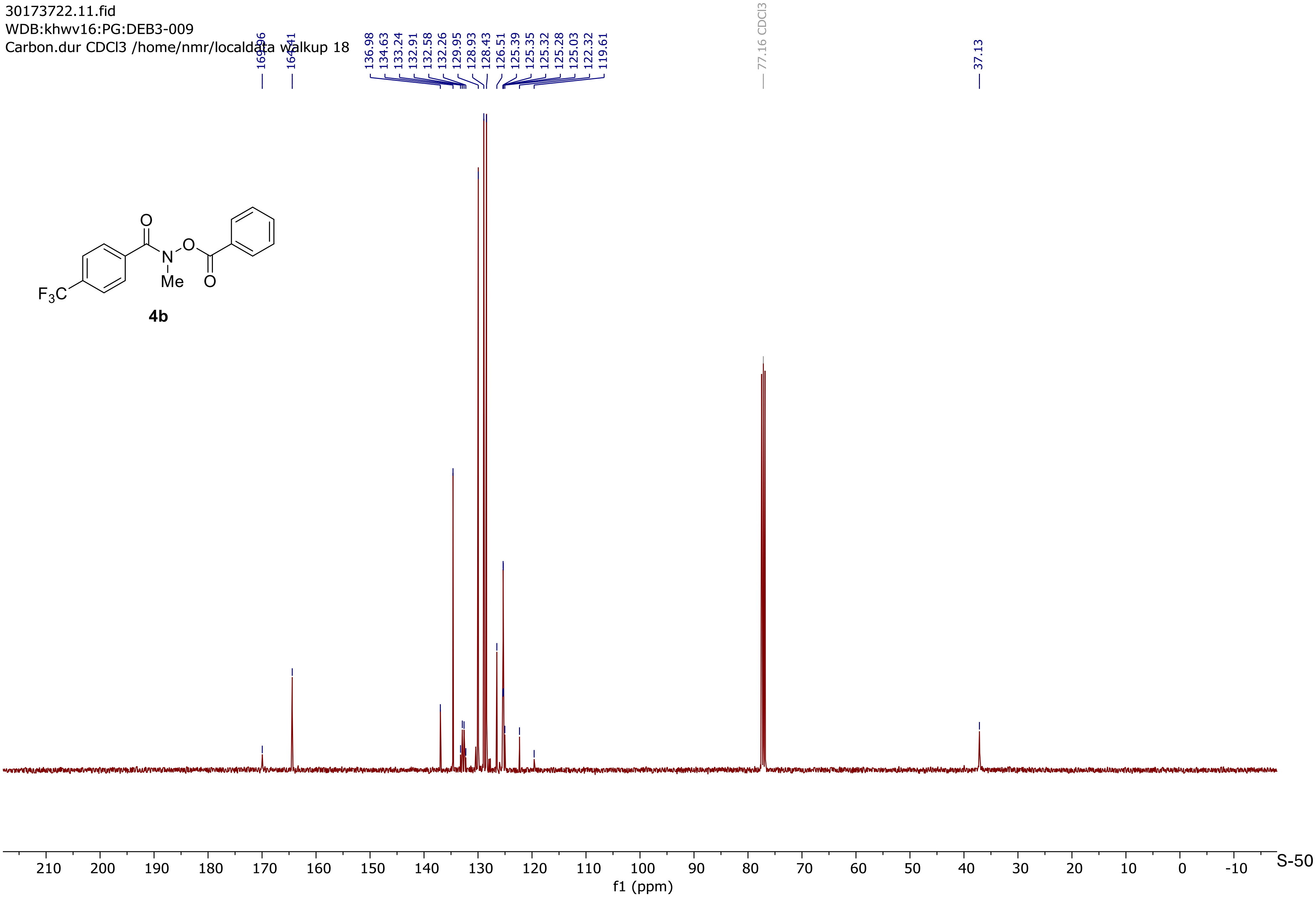

Figure S12; $^{13}C\{^{1}H\}$ NMR (101 MHz, $CDCl_3$) for compound **4b**.

17112904.13.fid
WDB:DEB:PG:DEB1-110-FR10-19
F19_limits_dec.dur CDCl3 /home/nmr/localdata walkup 7

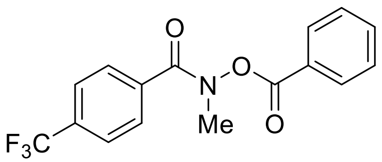

4b

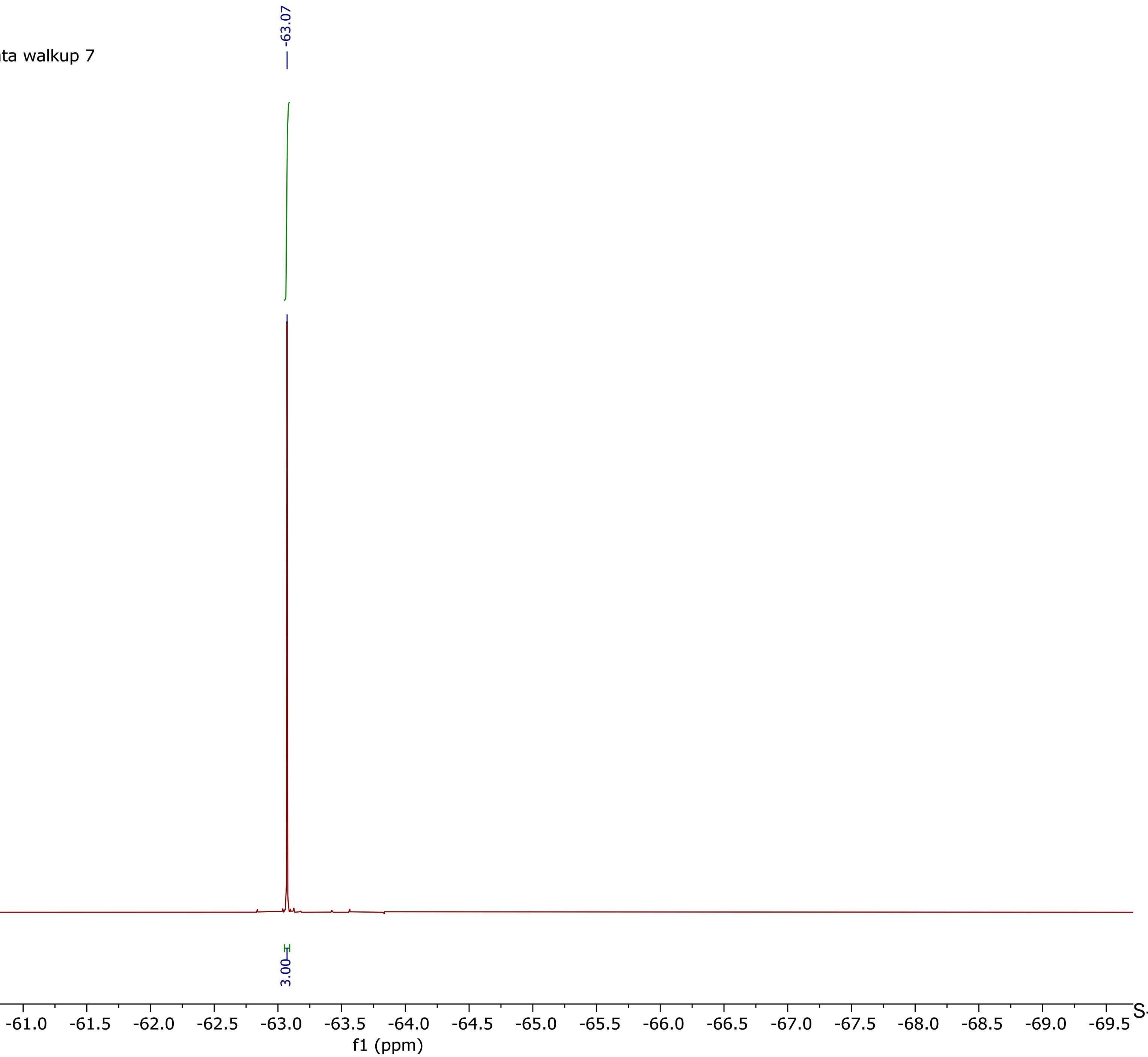

Figure S13; $^{19}F\{^1H\}$ NMR (376 MHz, $CDCl_3$) for compound **4b**.

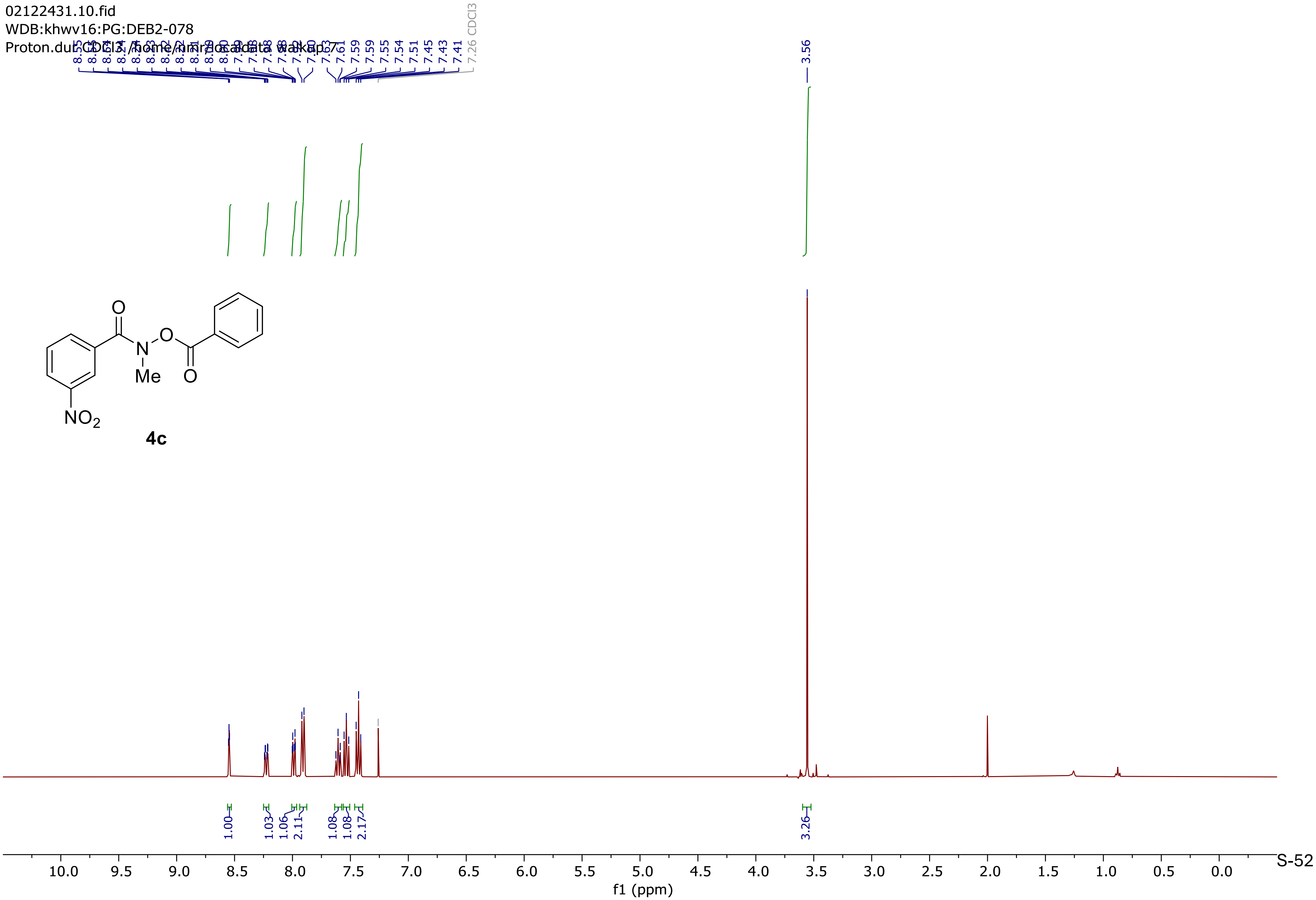

4c

Figure S14; ^{1}H NMR (400 MHz, $CDCl_3$) for compound **4c**.

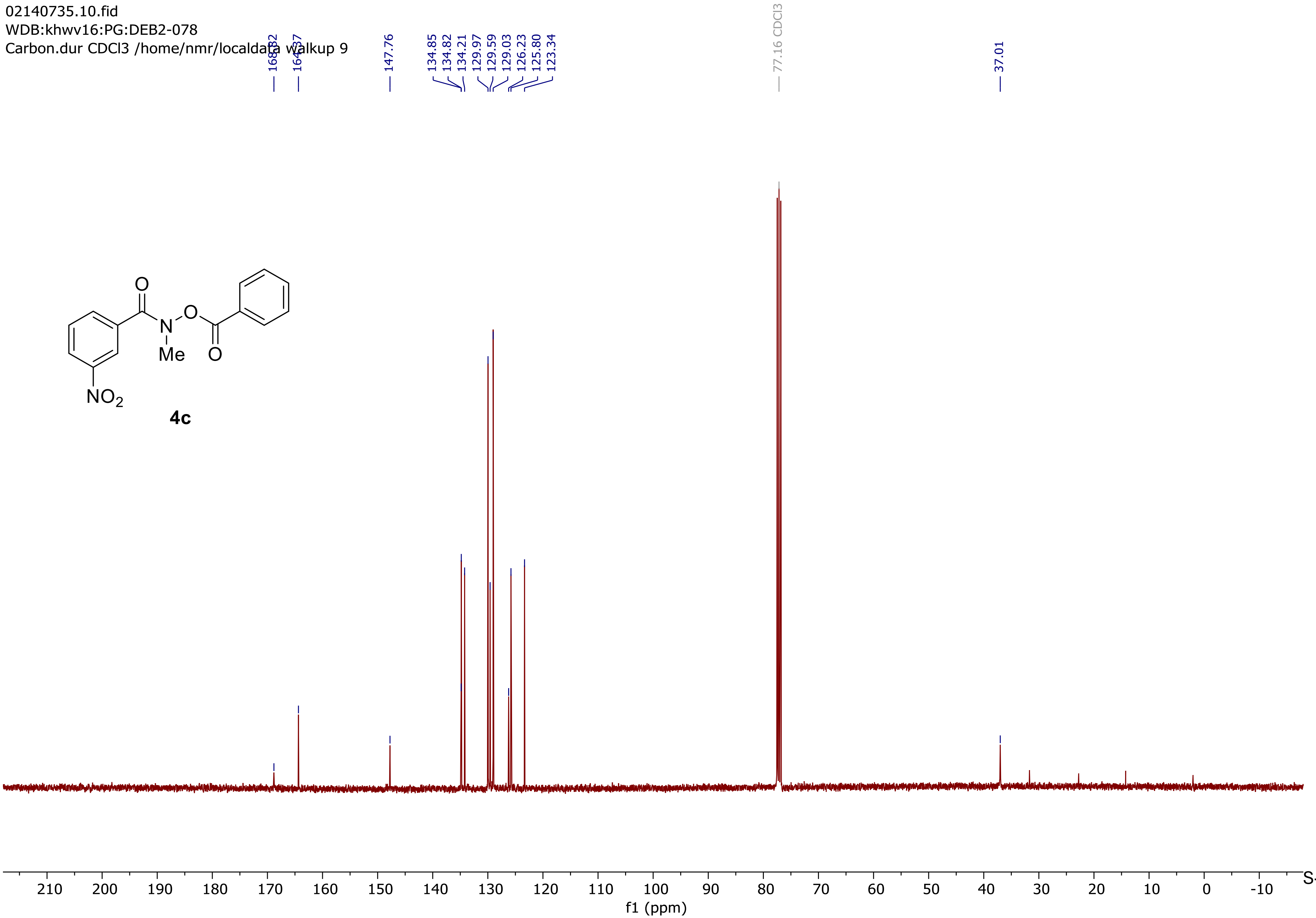

Figure S15; $^{13}C\{^{1}H\}$ NMR (101 MHz, $CDCl_3$) for compound **4c**.

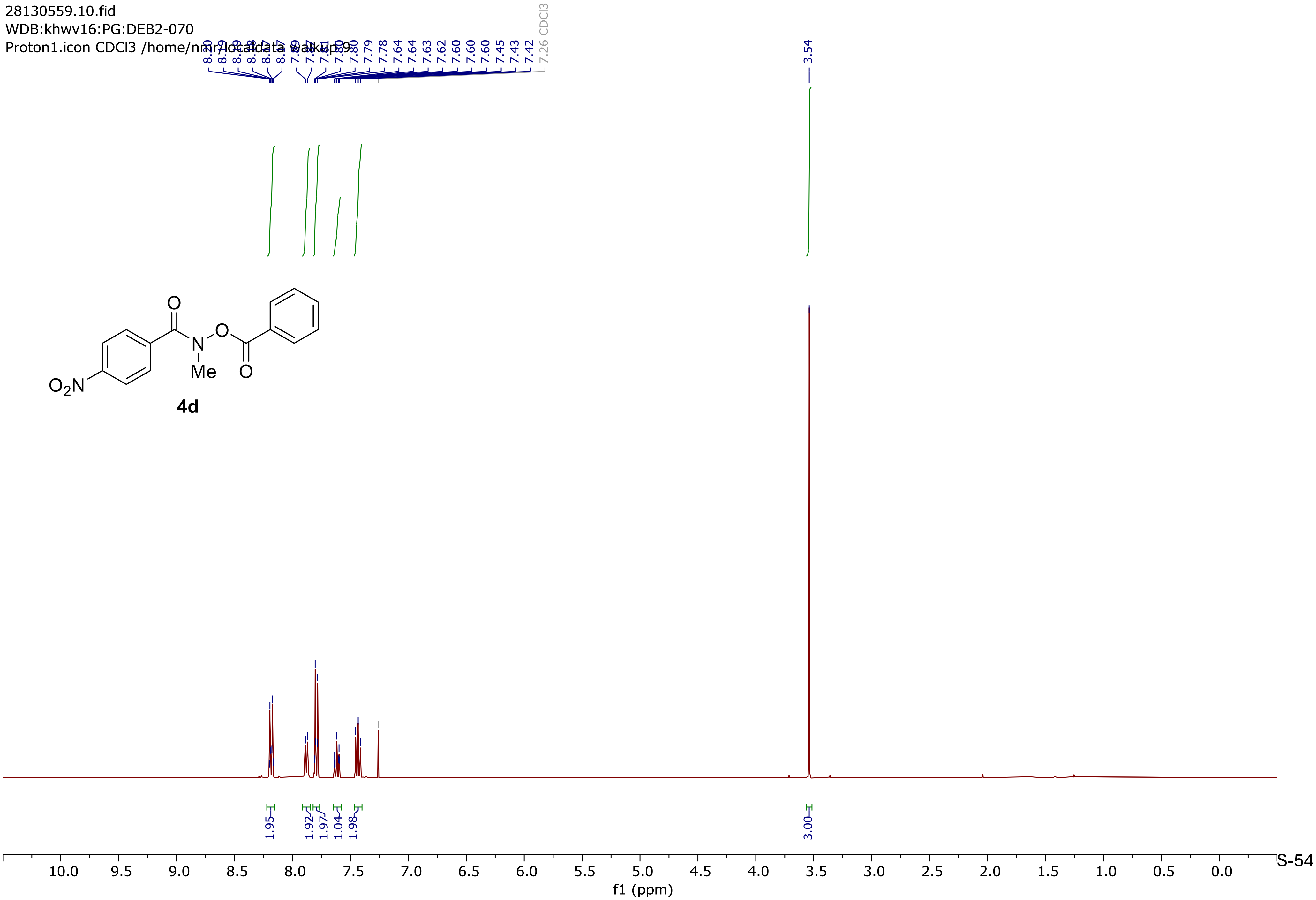

Figure S16; ^{1}H NMR (400 MHz, CDCl$_3$) for compound **4d**.

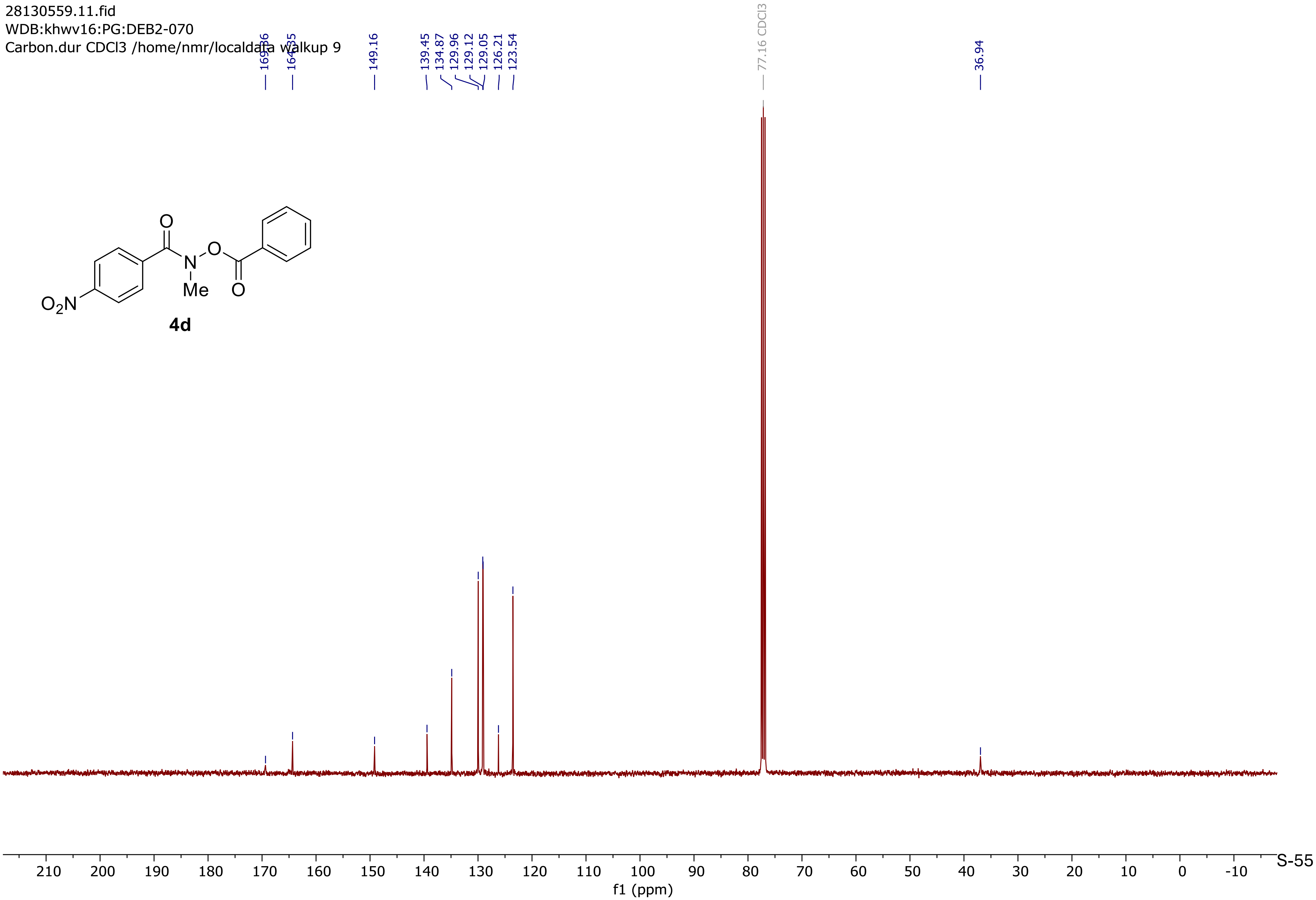

4d

Figure S17; ${}^{13}C\{{}^{1}H\}$ NMR (101 MHz, $CDCl_3$) for compound **4d**.

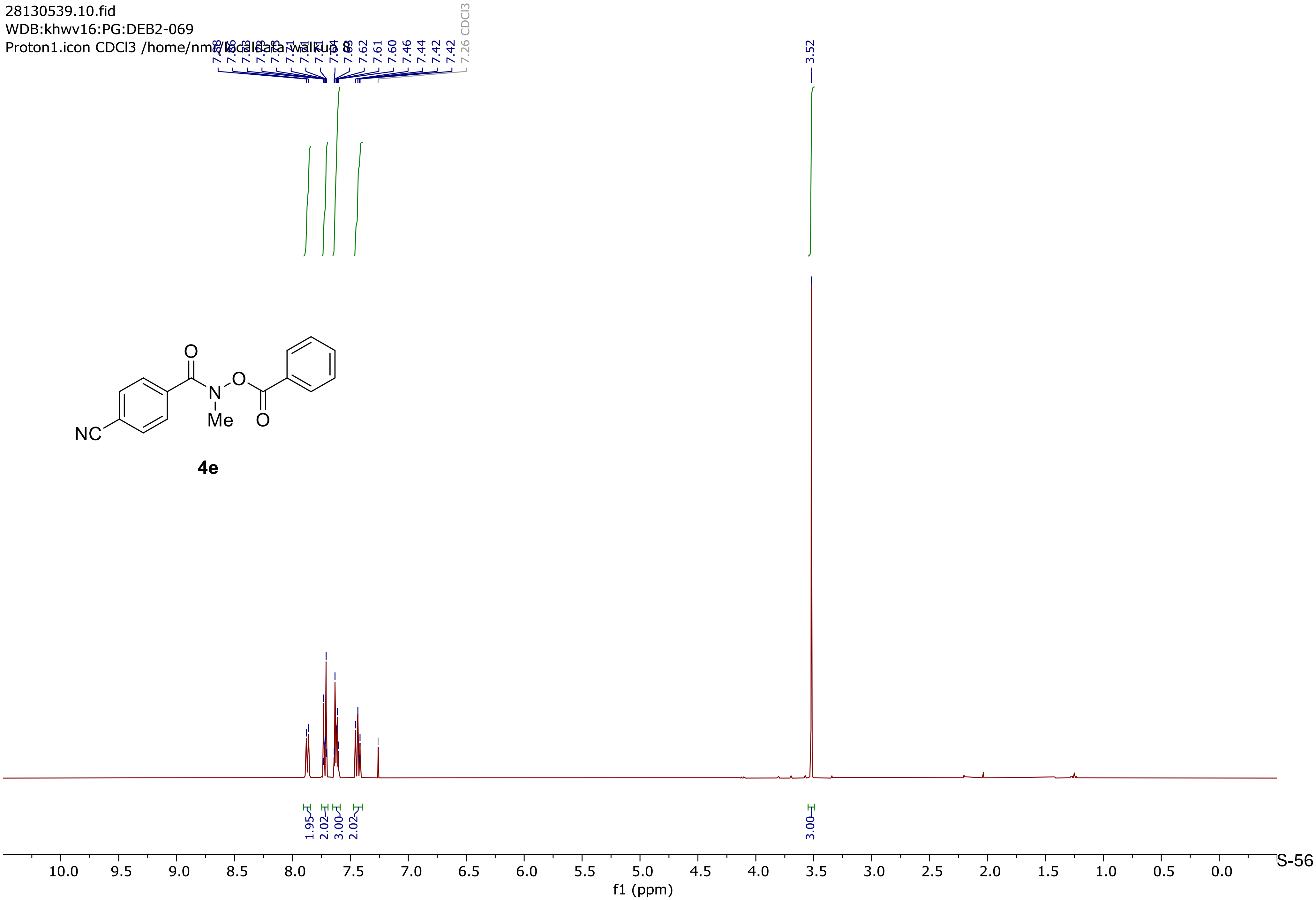

Figure S18; ^{1}H NMR (400 MHz, $CDCl_3$) for compound **4e**.

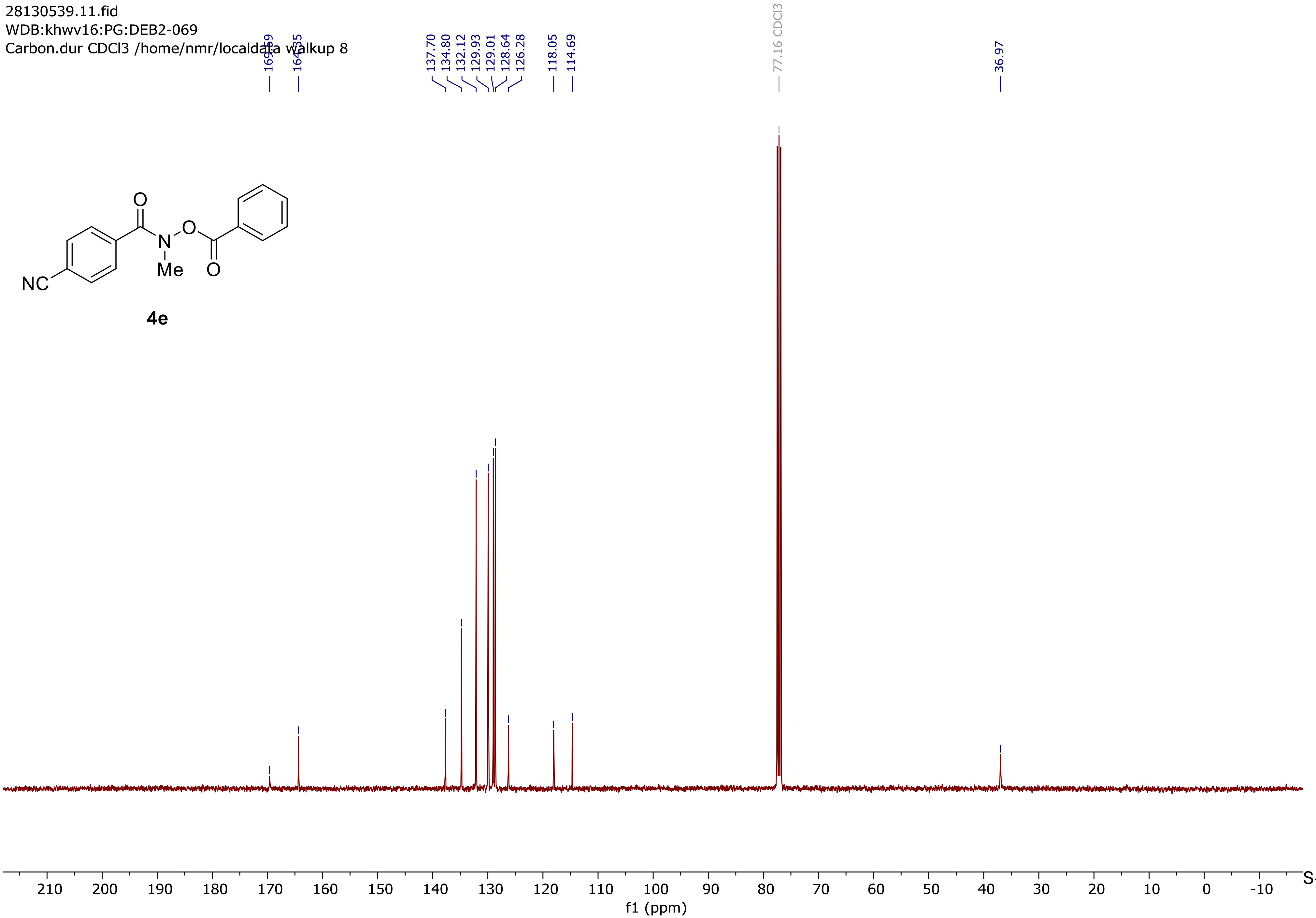

Figure S19; $^{13}C\{^{1}H\}$ NMR (101 MHz, $CDCl_3$) for compound **4e**.

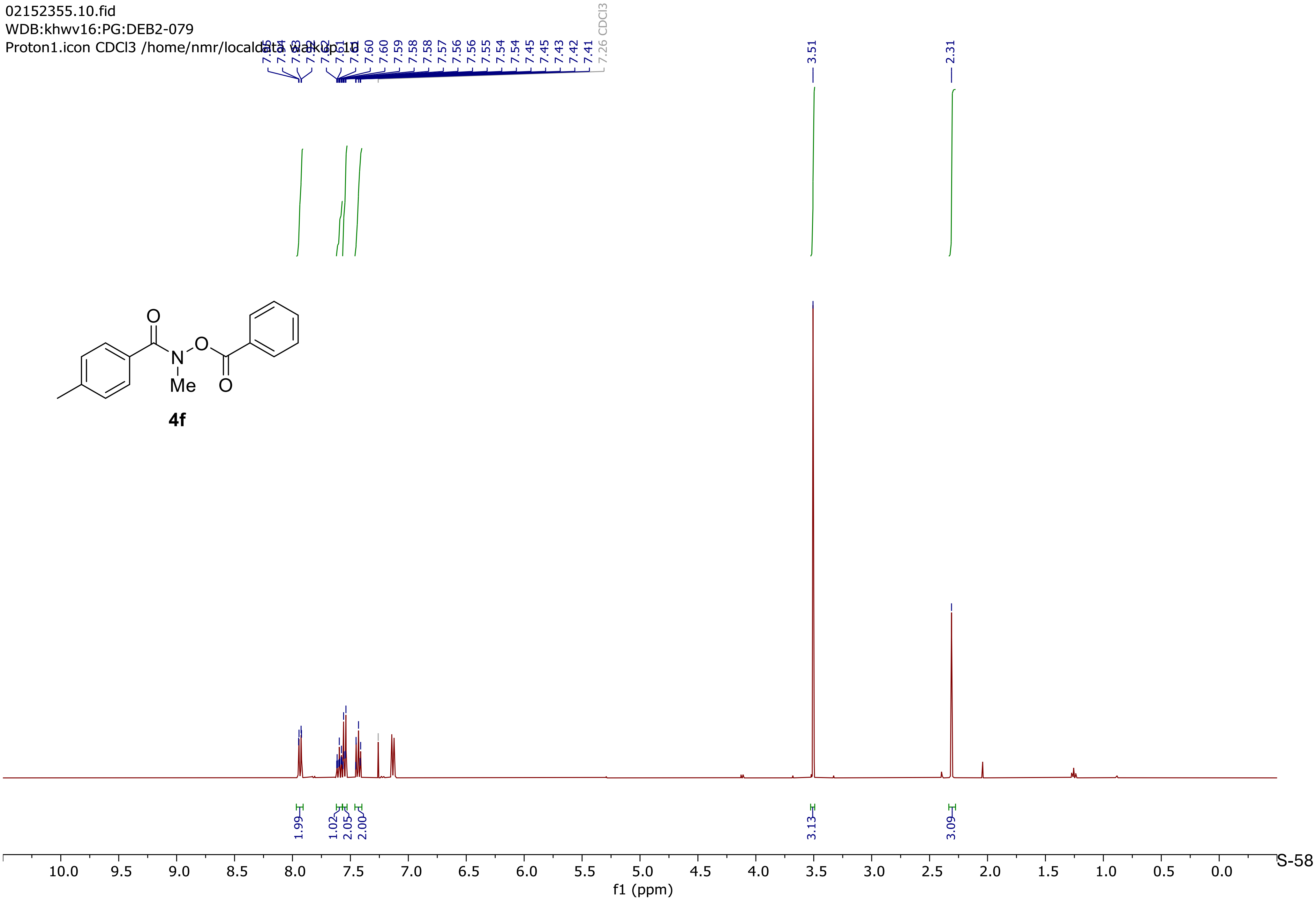

Figure S20; ^{1}H NMR (400 MHz, $CDCl_3$) for compound **4f**.

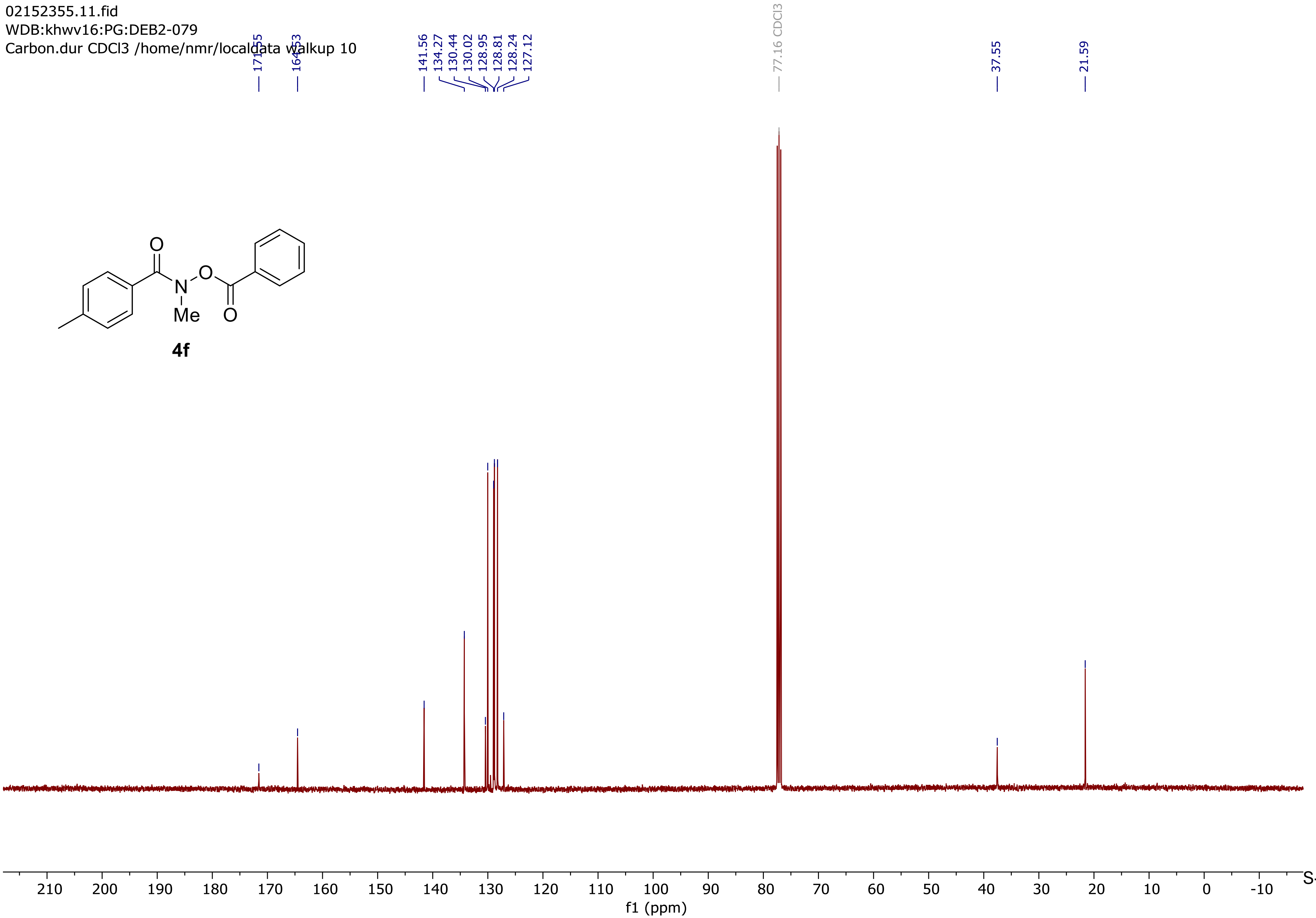

Figure S21; $^{13}C\{^{1}H\}$ NMR (101 MHz, $CDCl_3$) for compound **4f**.

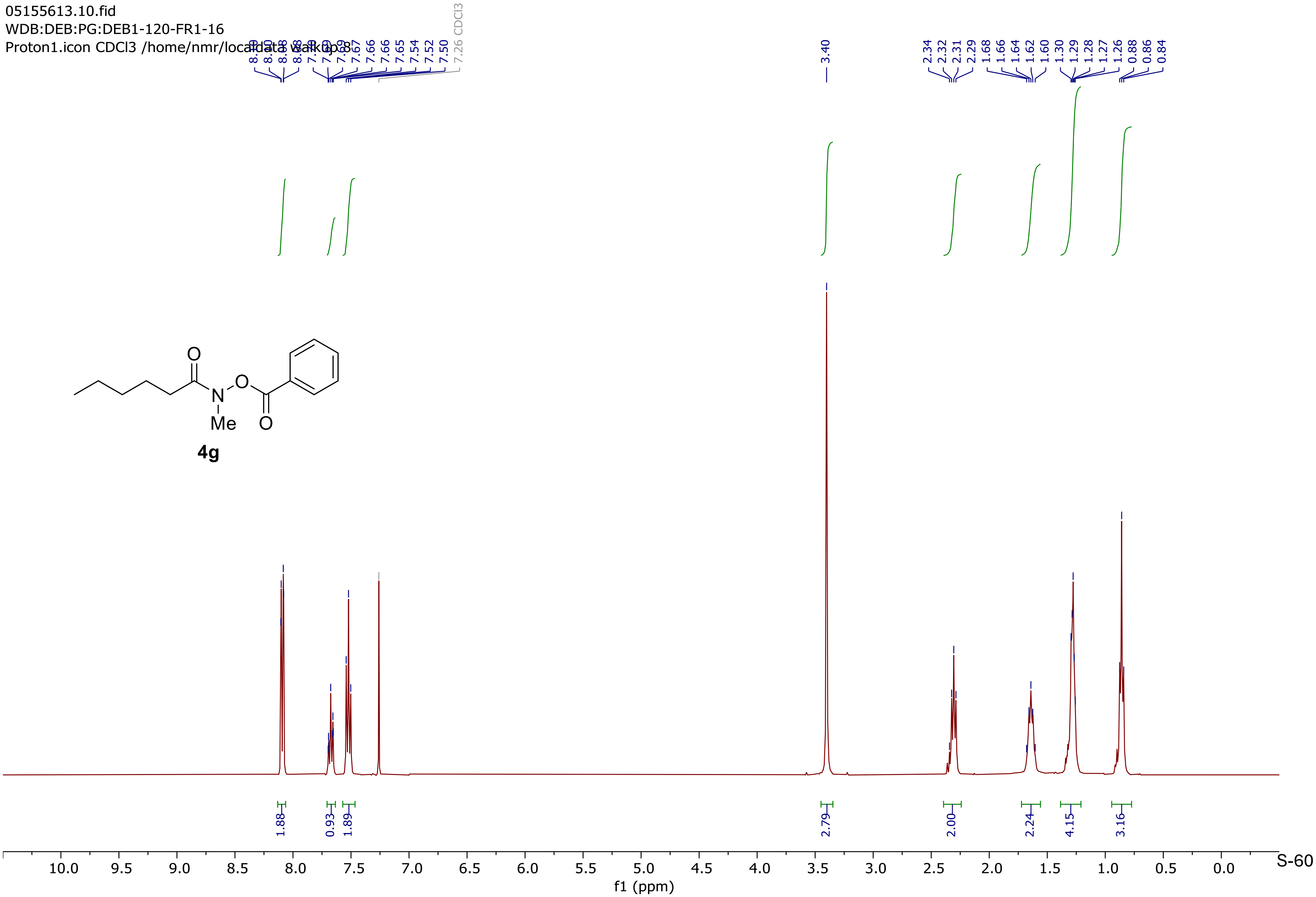

4g

Figure S22; ^{1}H NMR (400 MHz, CDCl$_3$) for compound **4g**.

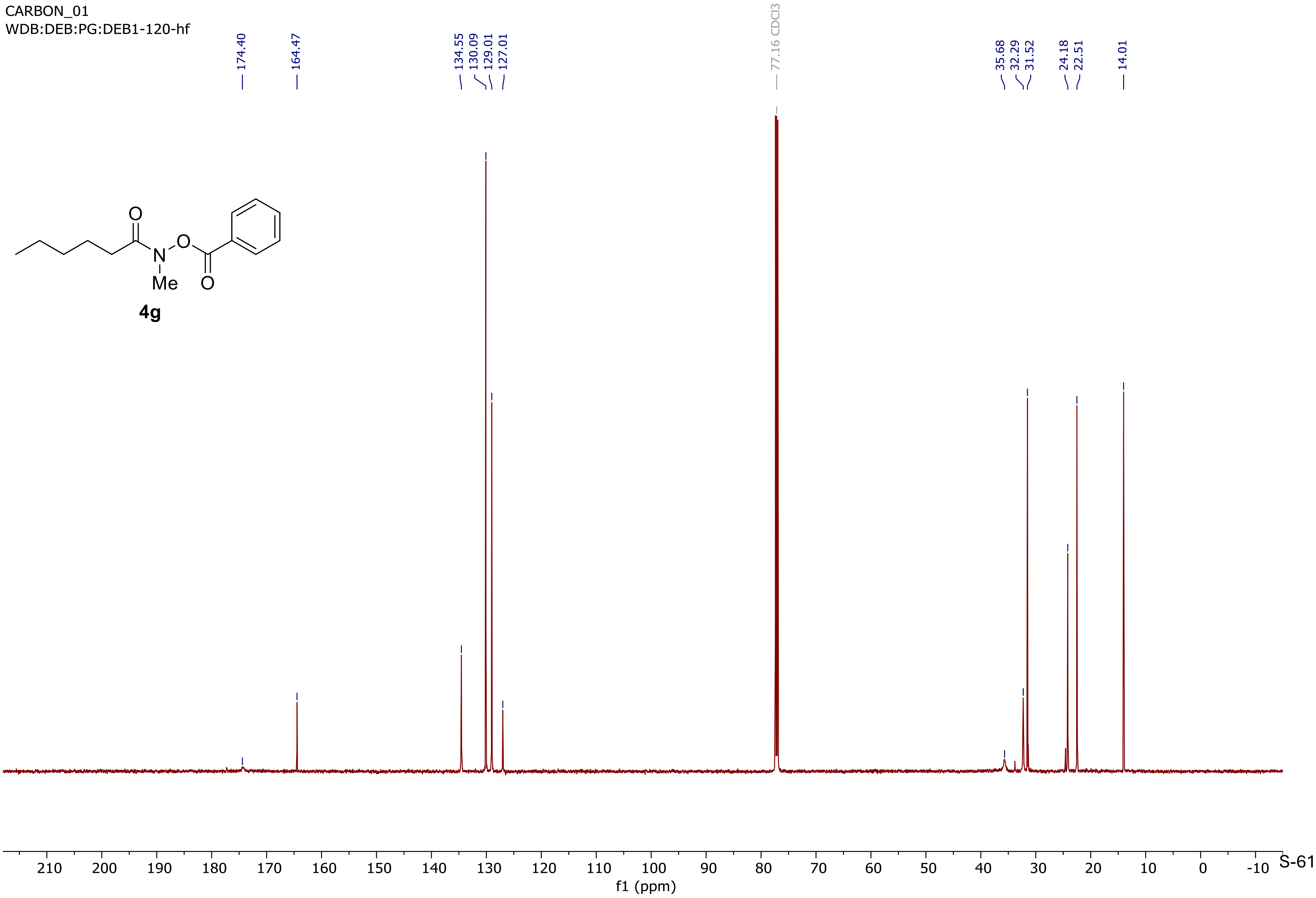

Figure S23; $^{13}C\{^{1}H\}$ NMR (151 MHz, $CDCl_3$) for compound **4g**.

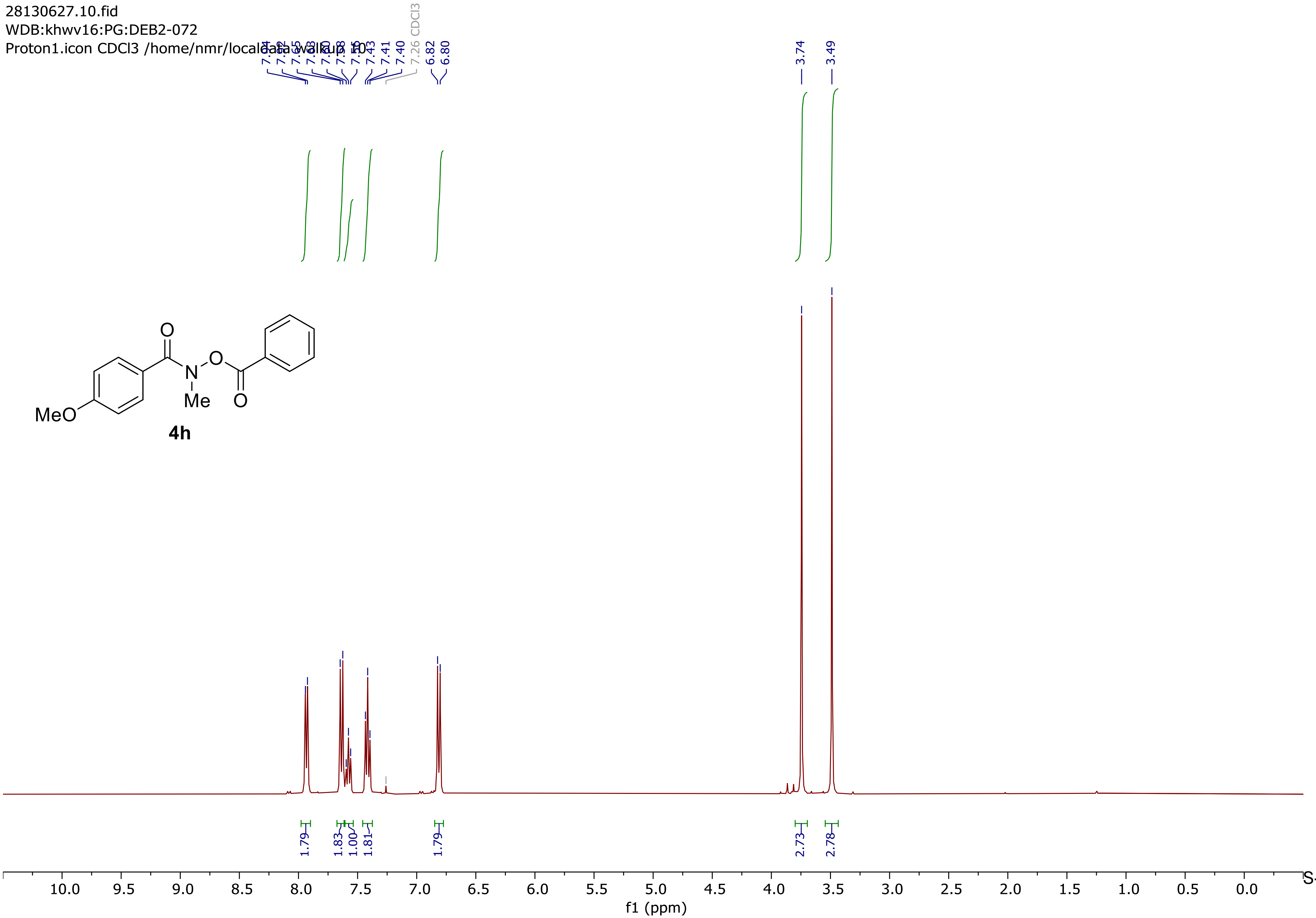

Figure S24; ^{1}H NMR (400 MHz, $CDCl_3$) for compound **4h**.

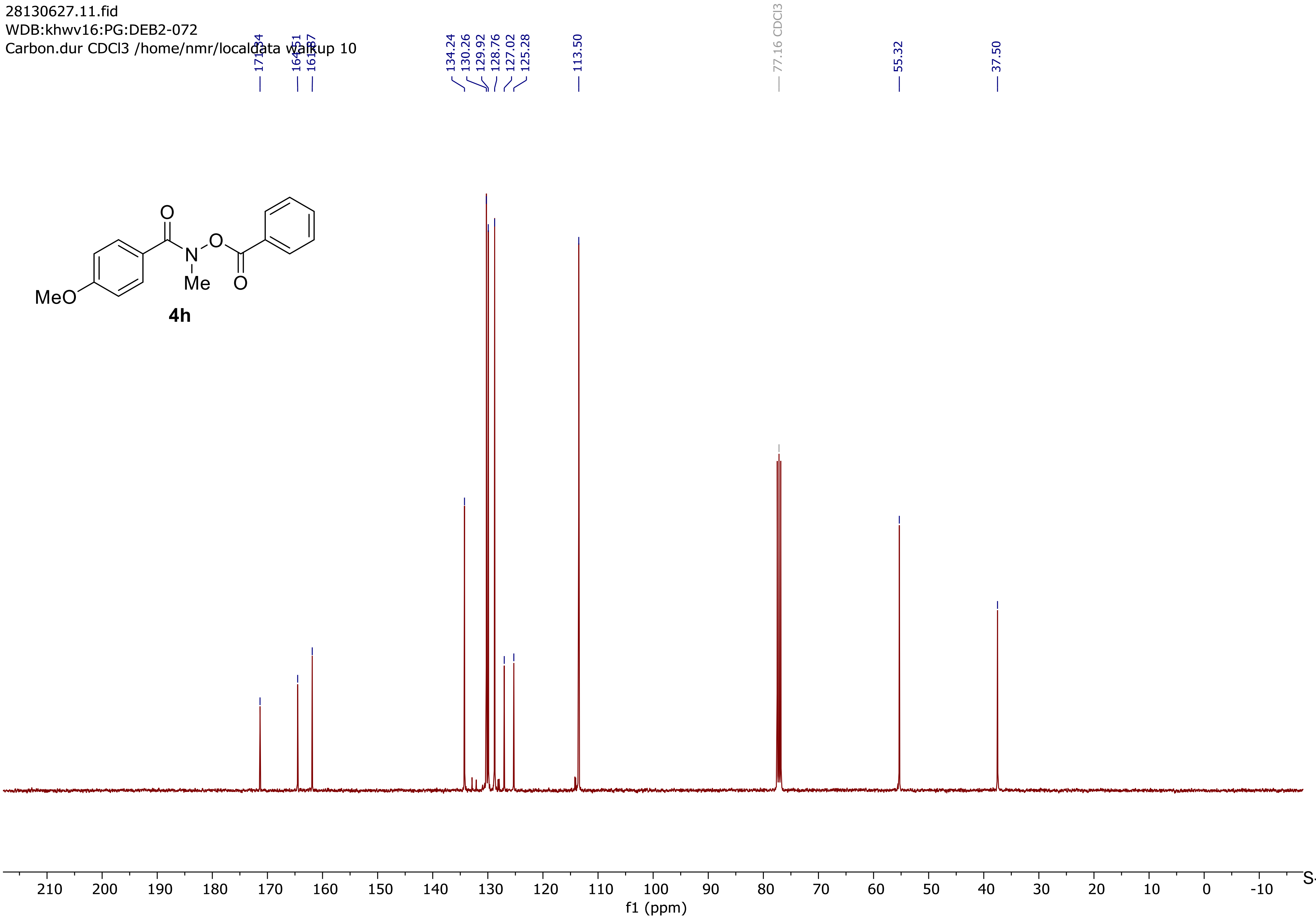

Figure S25; $^{13}C\{^{1}H\}$ NMR (101 MHz, $CDCl_3$) for compound **4h**.

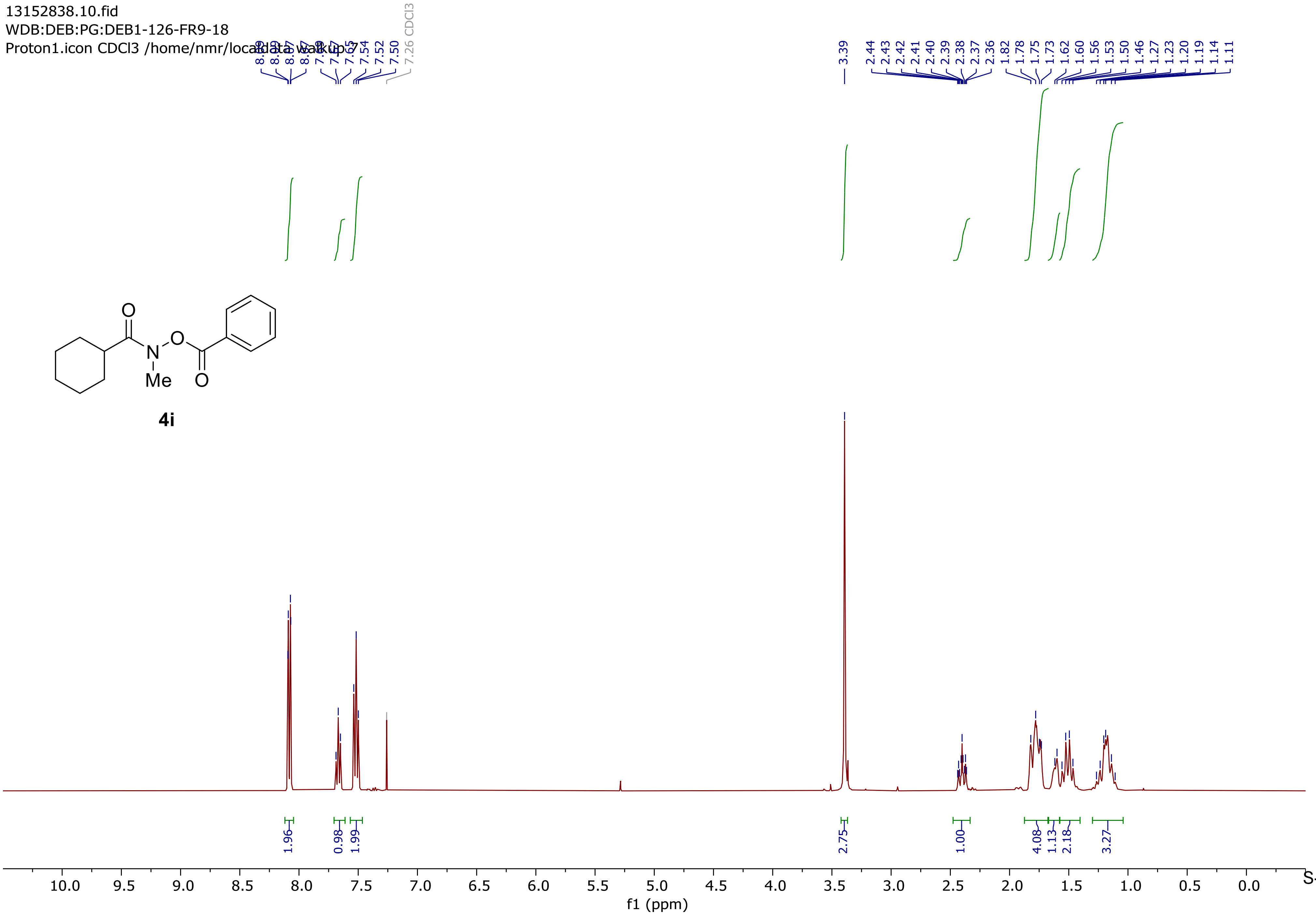

Figure S26; ^{1}H NMR (400 MHz, $CDCl_3$) for compound **4i**.

CARBON_01
WDB:DEB:PG:DEB1-126-hf

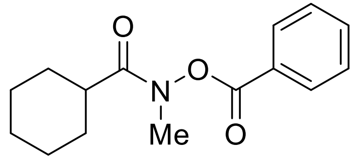

4i

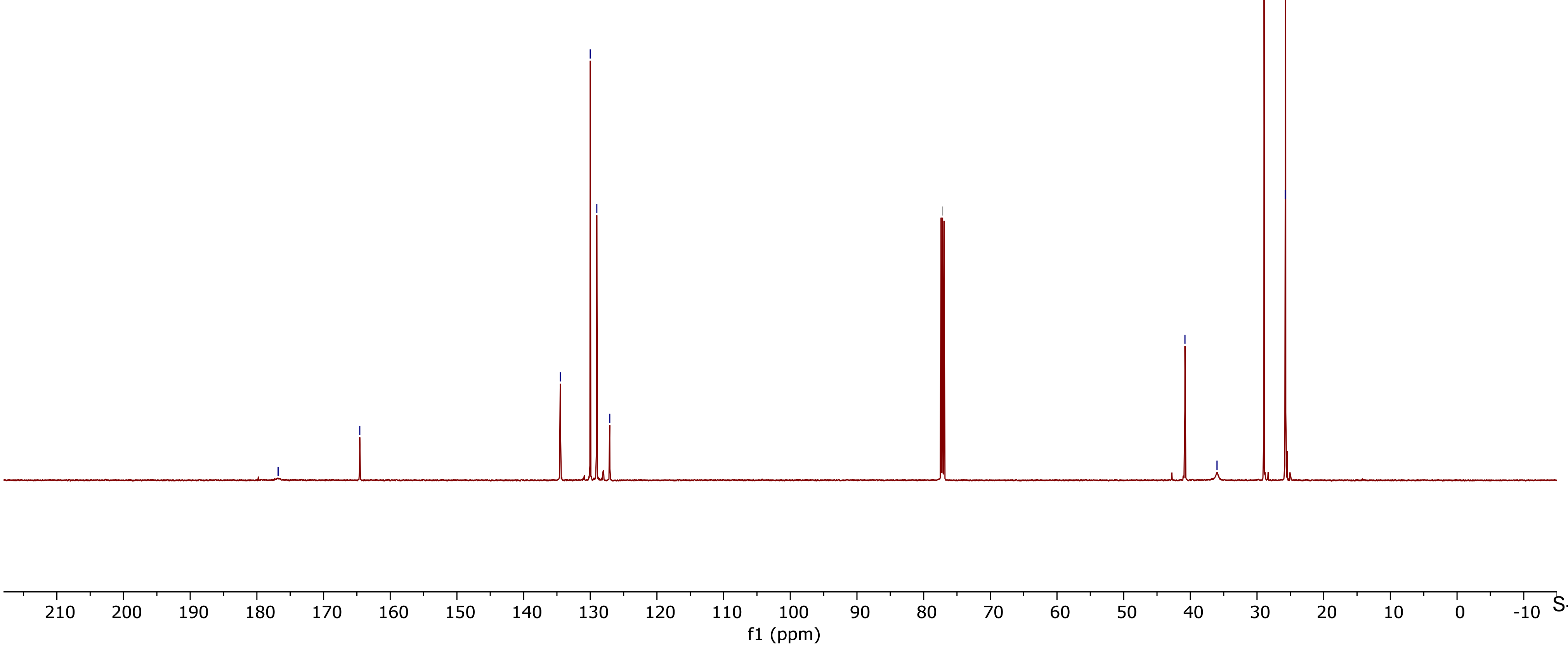

Figure S27; $^{13}C\{^1H\}$ NMR (151 MHz, $CDCl_3$) for compound **4i**.

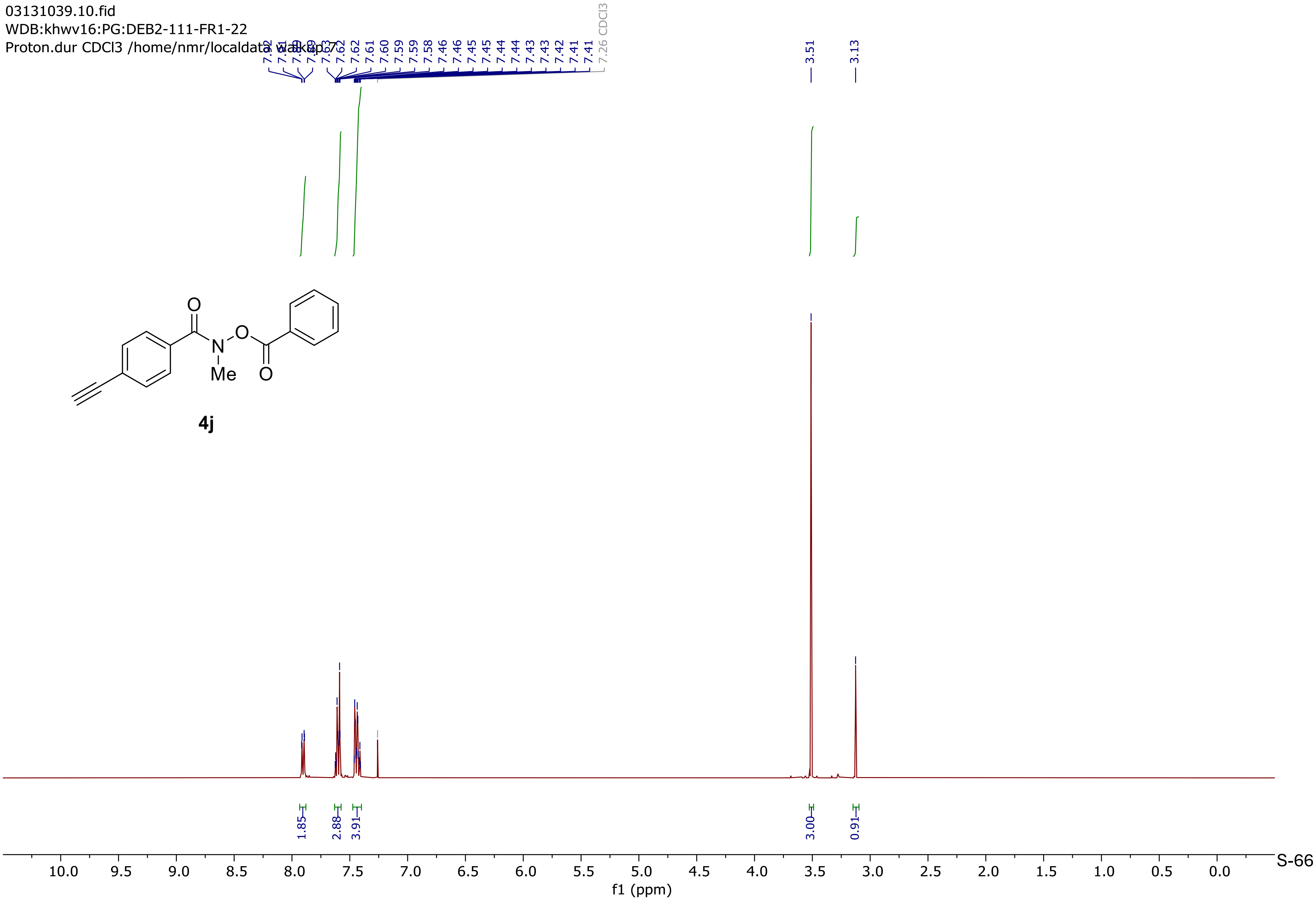

Figure S28; ^{1}H NMR (400 MHz, $CDCl_3$) for compound **4j**.

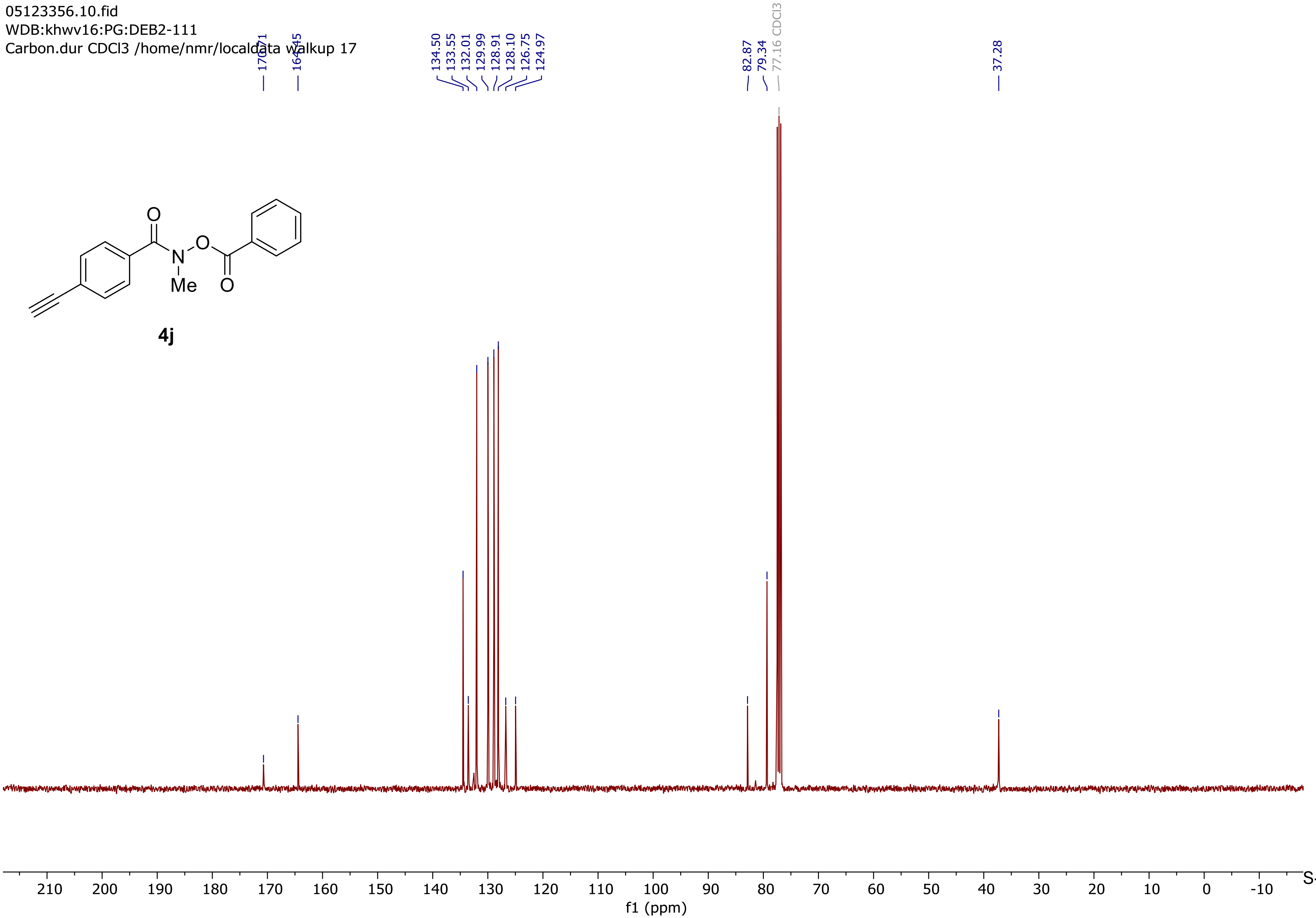

Figure S29; $^{13}C\{^{1}H\}$ NMR (101 MHz, $CDCl_3$) for compound **4j**.

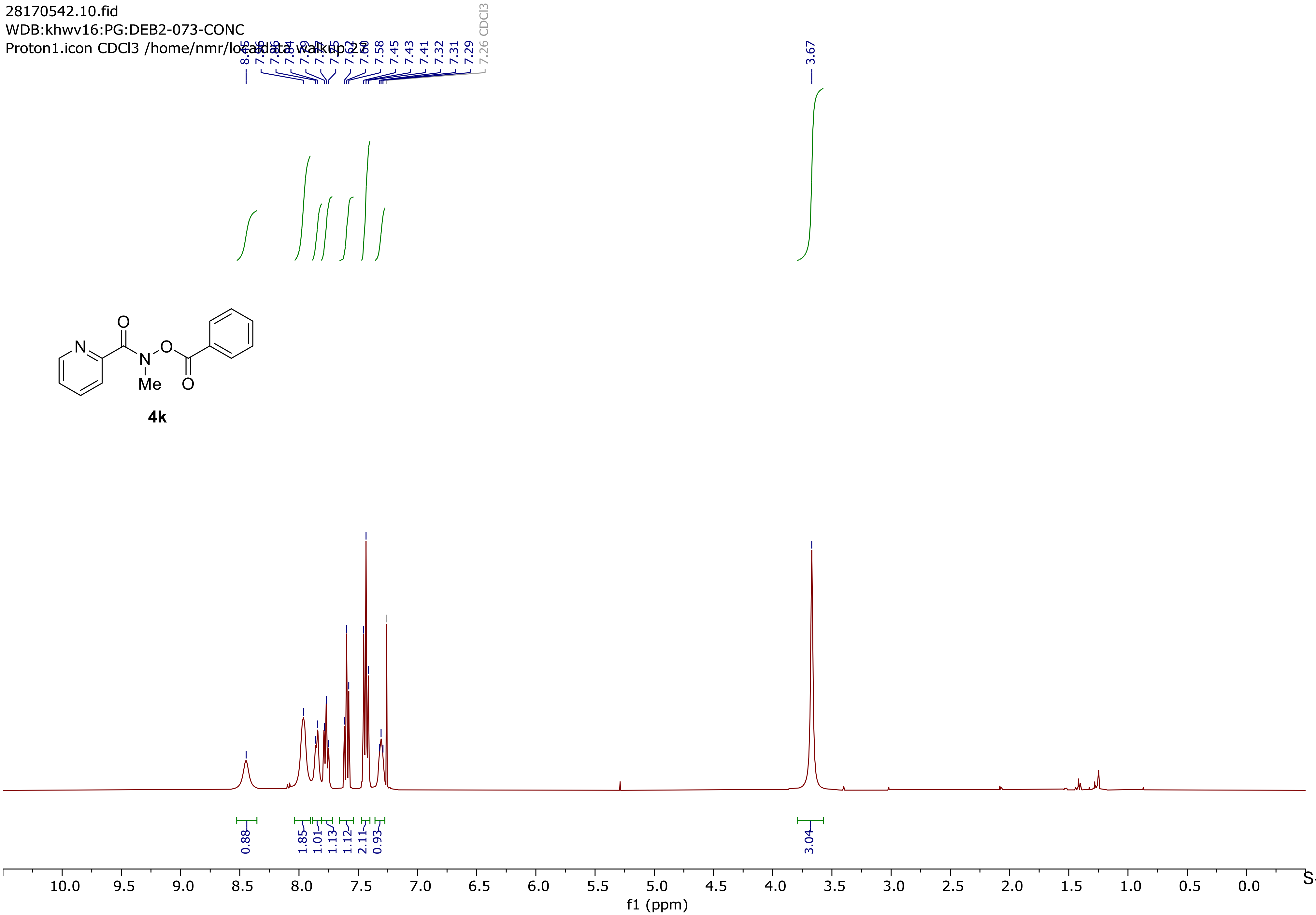

Figure S30; ^{1}H NMR (400 MHz, $CDCl_3$) for compound **4k**.

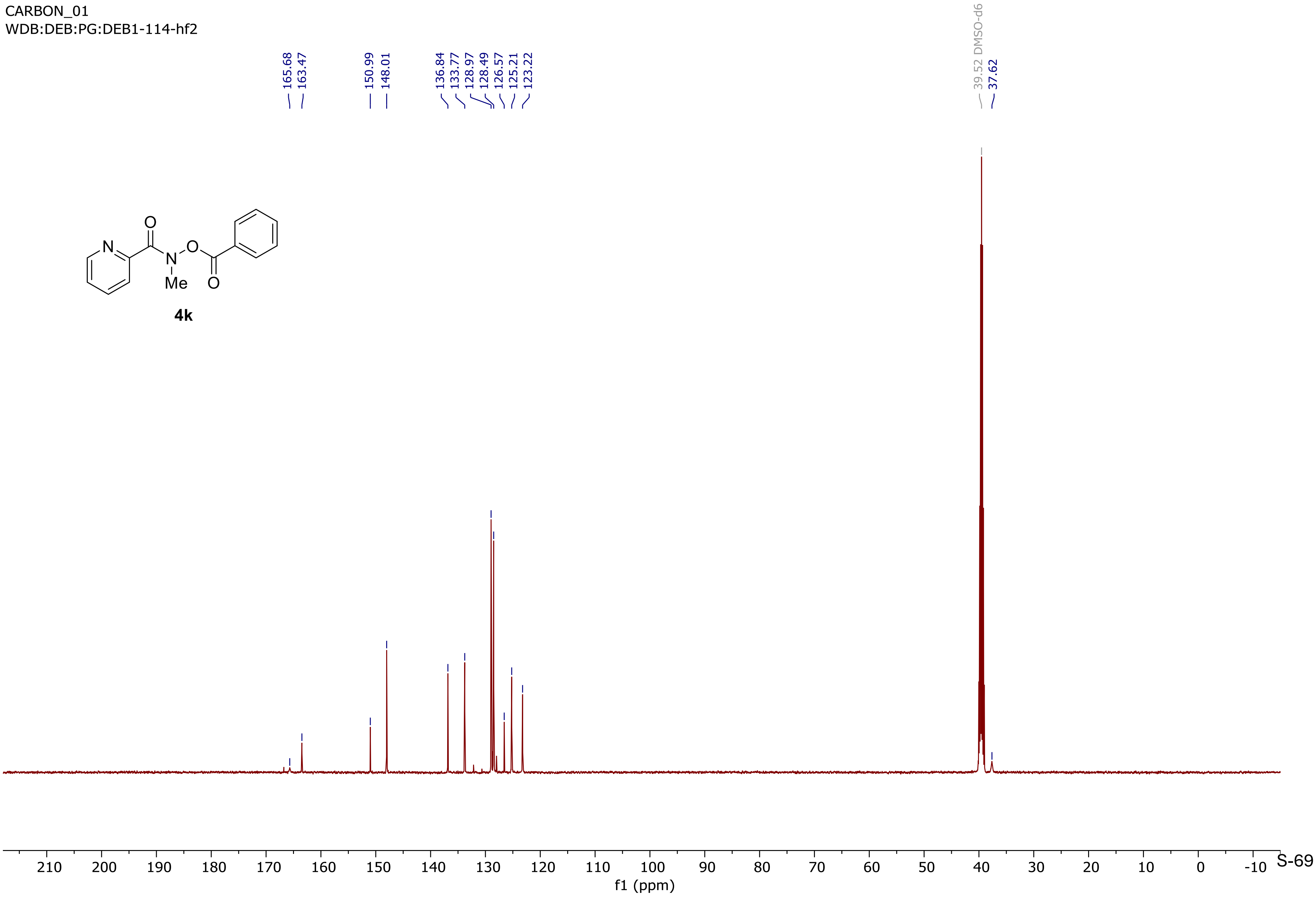

Figure S31; $^{13}C\{^{1}H\}$ NMR (126 MHz, DMSO-d_6) for compound **4k**.

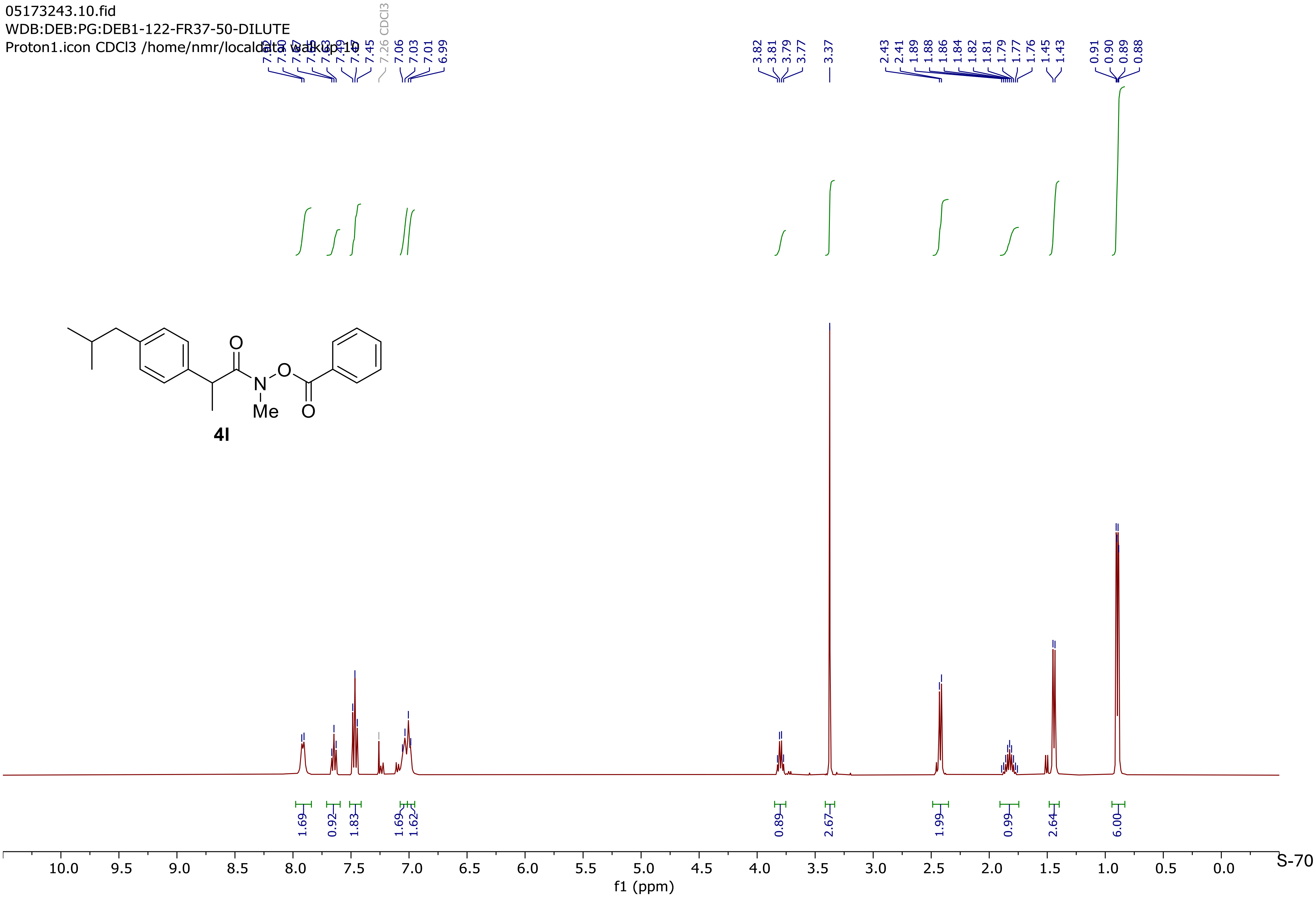

Figure S32; ^{1}H NMR (400 MHz, $CDCl_3$) for compound **4l**.

CARBON_01
WDB:DEB:PG:DEB1-122-hf

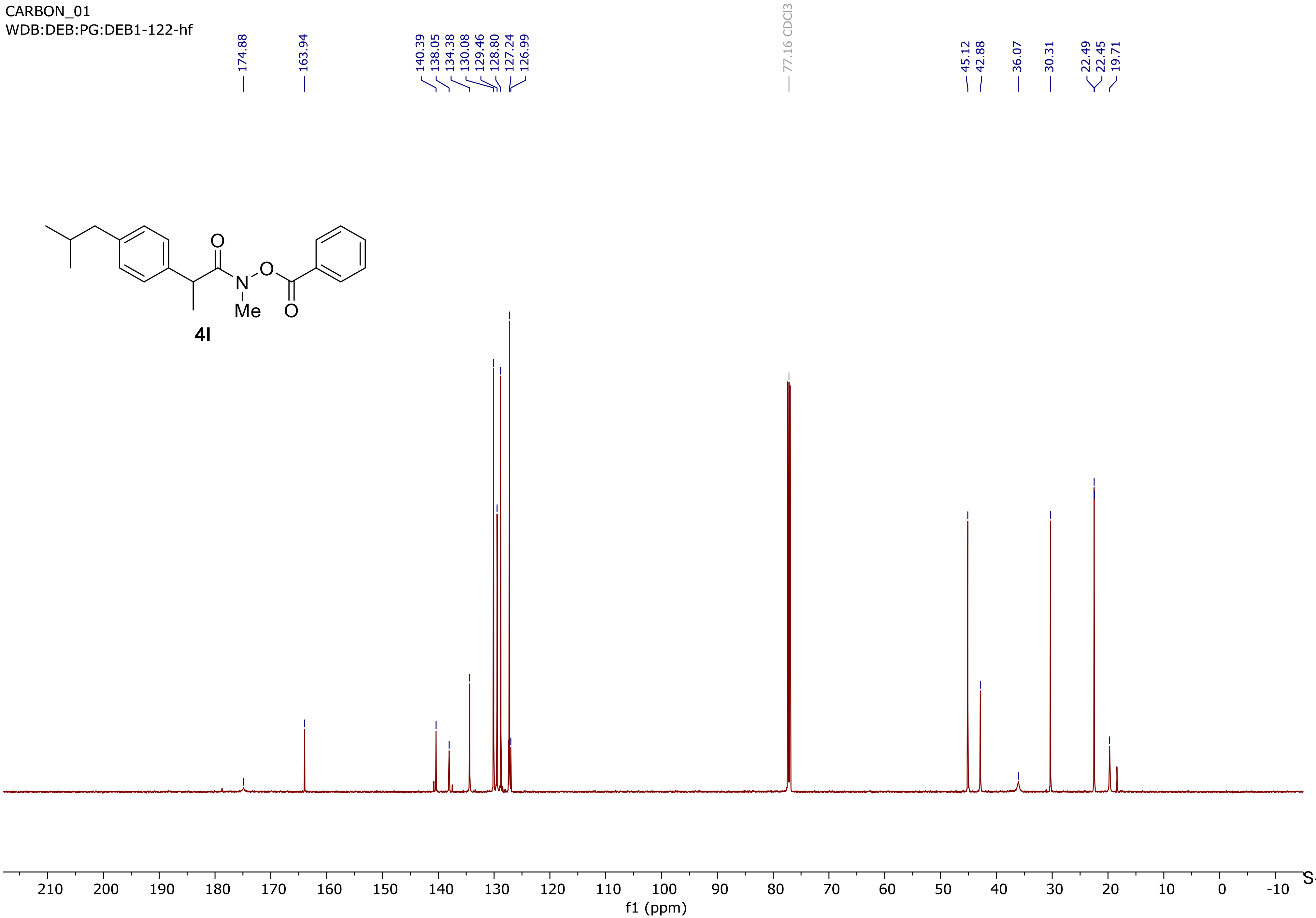

Figure S33; $^{13}C\{^{1}H\}$ NMR (151 MHz, $CDCl_3$) for compound **4l**.

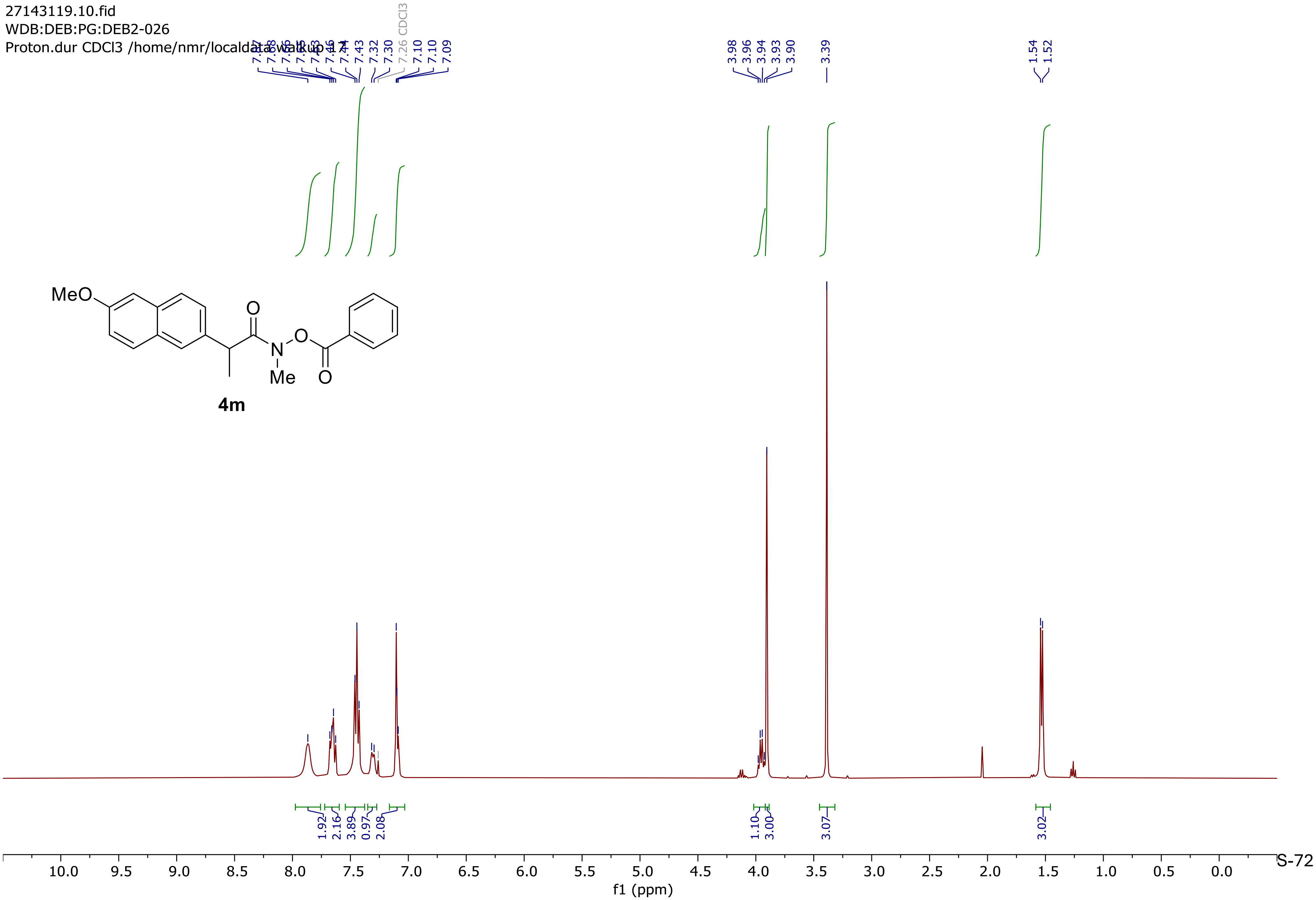

Figure S34; ^{1}H NMR (400 MHz, $CDCl_3$) for compound **4m**.

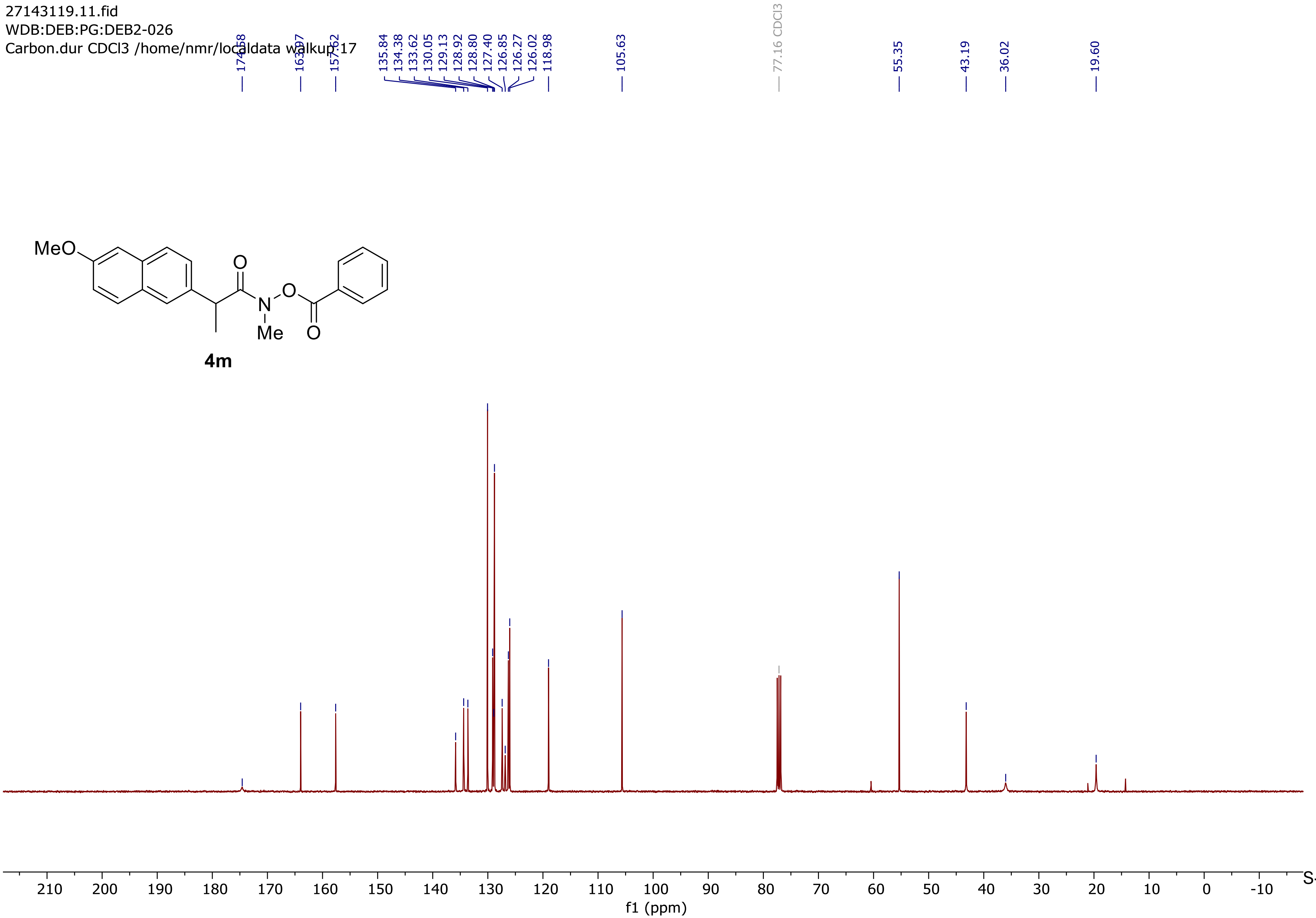

Figure S35; $^{13}C\{^{1}H\}$ NMR (101 MHz, $CDCl_3$) for compound **4m**.

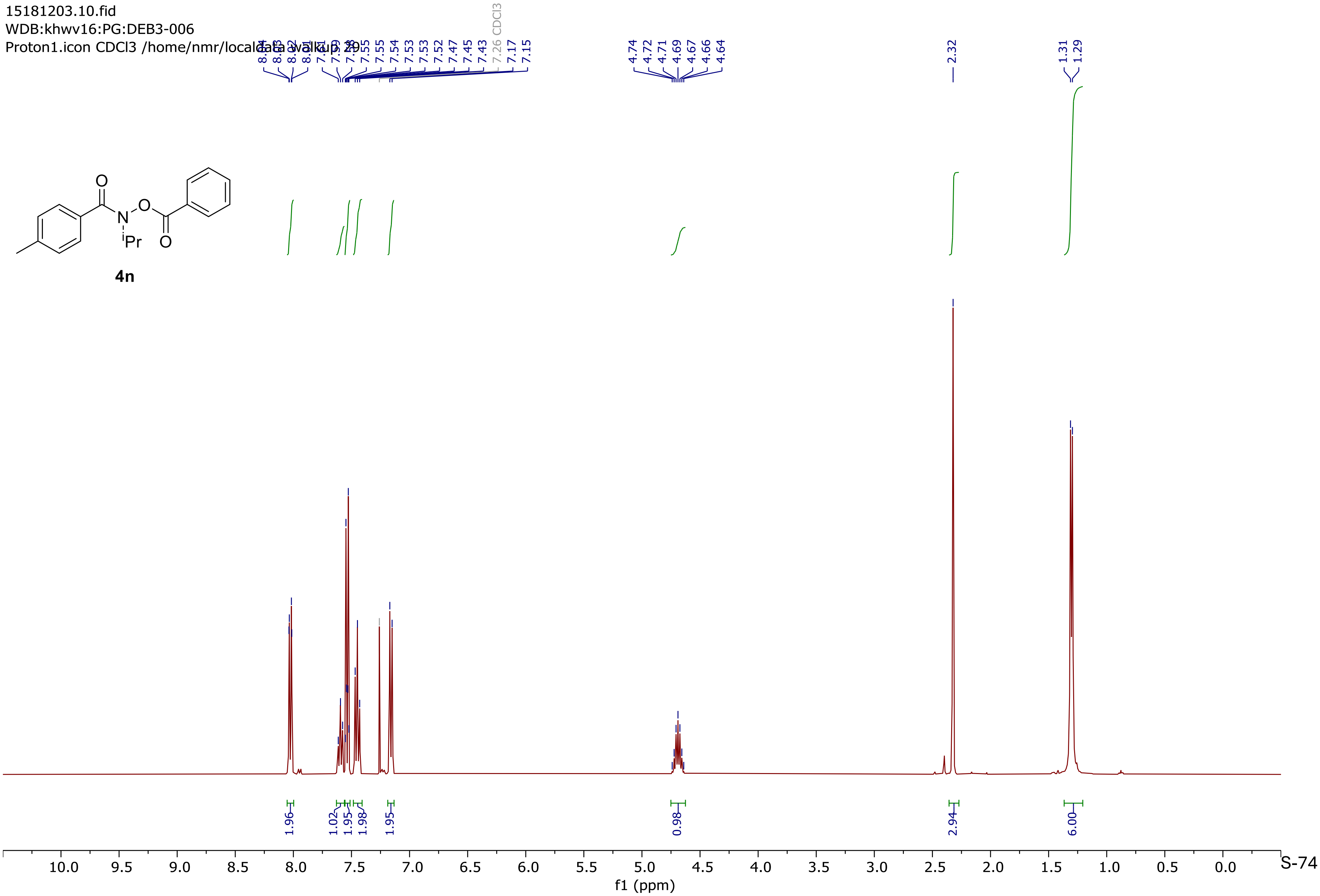

Figure S36; ^{1}H NMR (400 MHz, $CDCl_3$) for compound **4n**.

15181203.11.fid
WDB:khwv16:PG:DEB3-006
Carbon.dur CDCl3 /home/nmr/localdata walkup 29

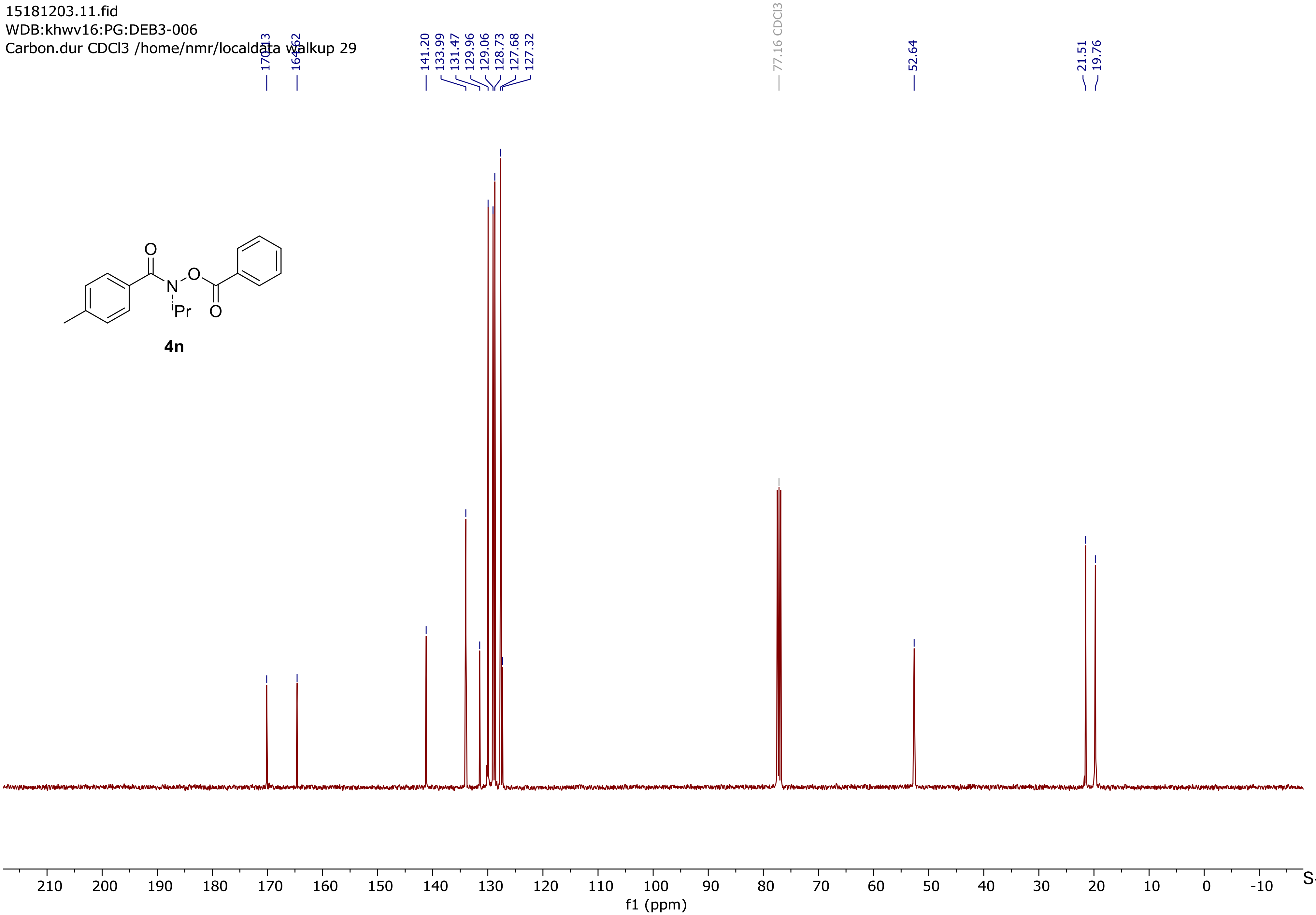

Figure S37; $^{13}C\{^{1}H\}$ NMR (101 MHz, $CDCl_3$) for compound **4n**.

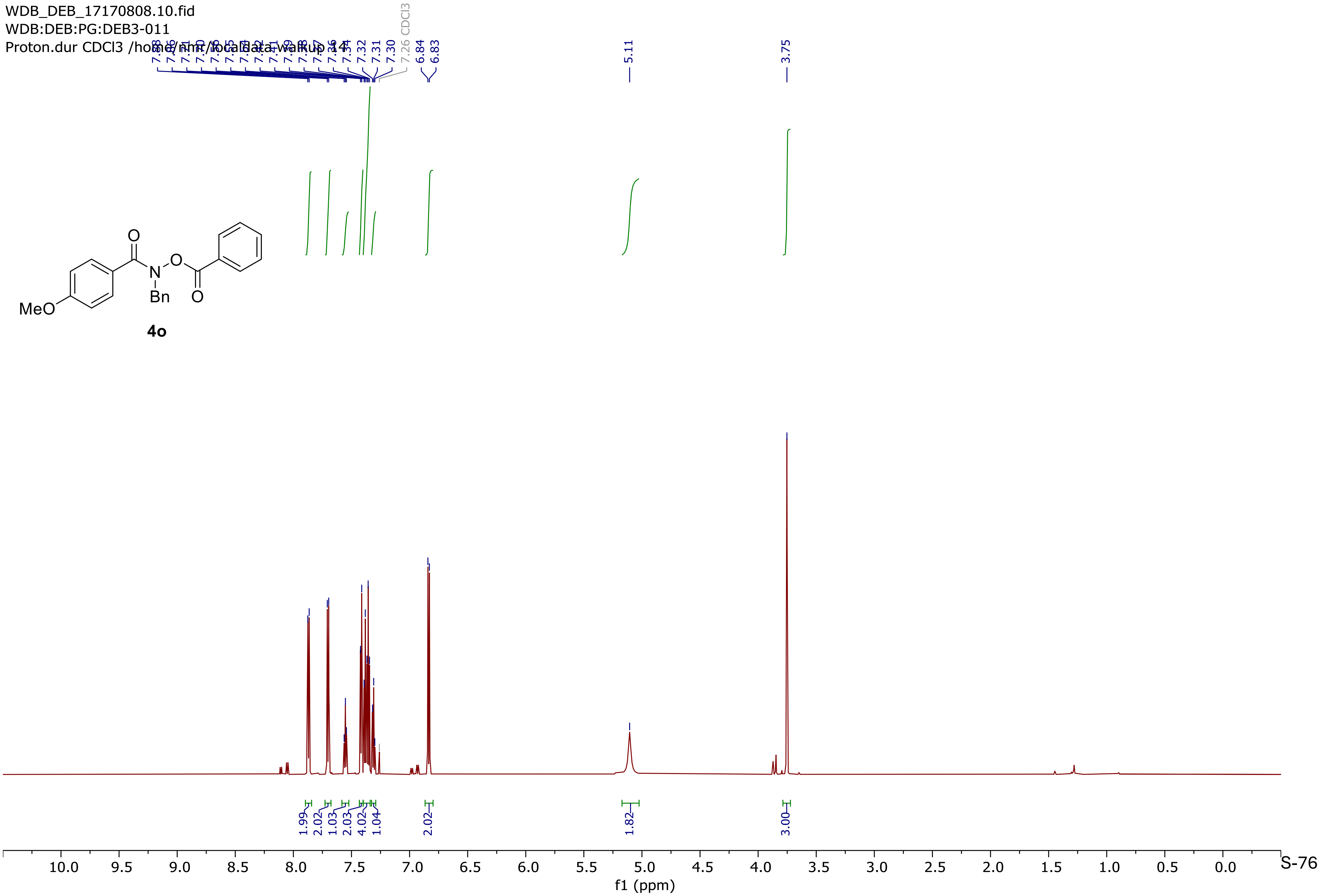

Figure S38; ^{1}H NMR (700 MHz, $CDCl_3$) for compound **4o**.

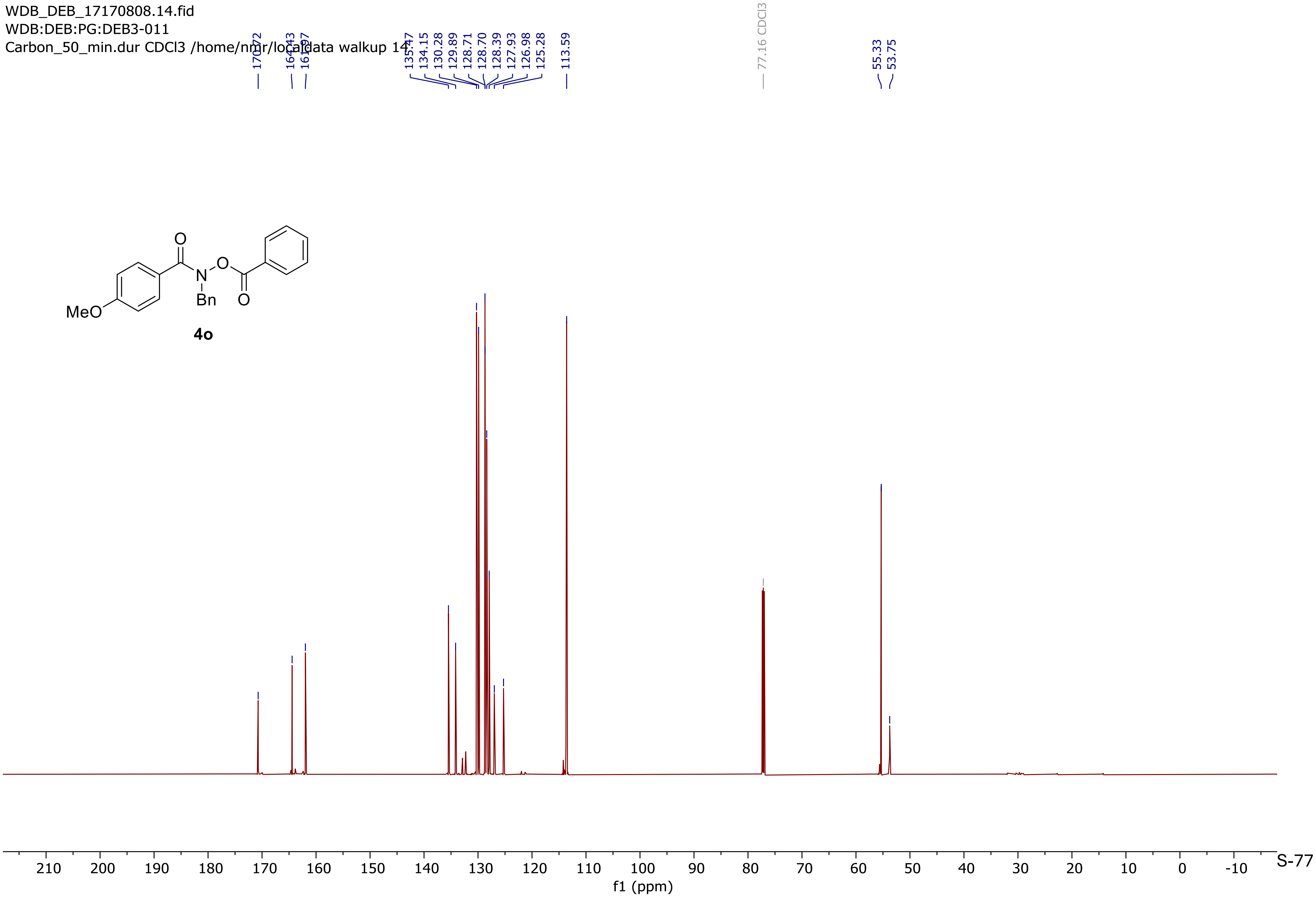

Figure S39; $^{13}C\{^{1}H\}$ NMR (176 MHz, $CDCl_3$) for compound **4o**.

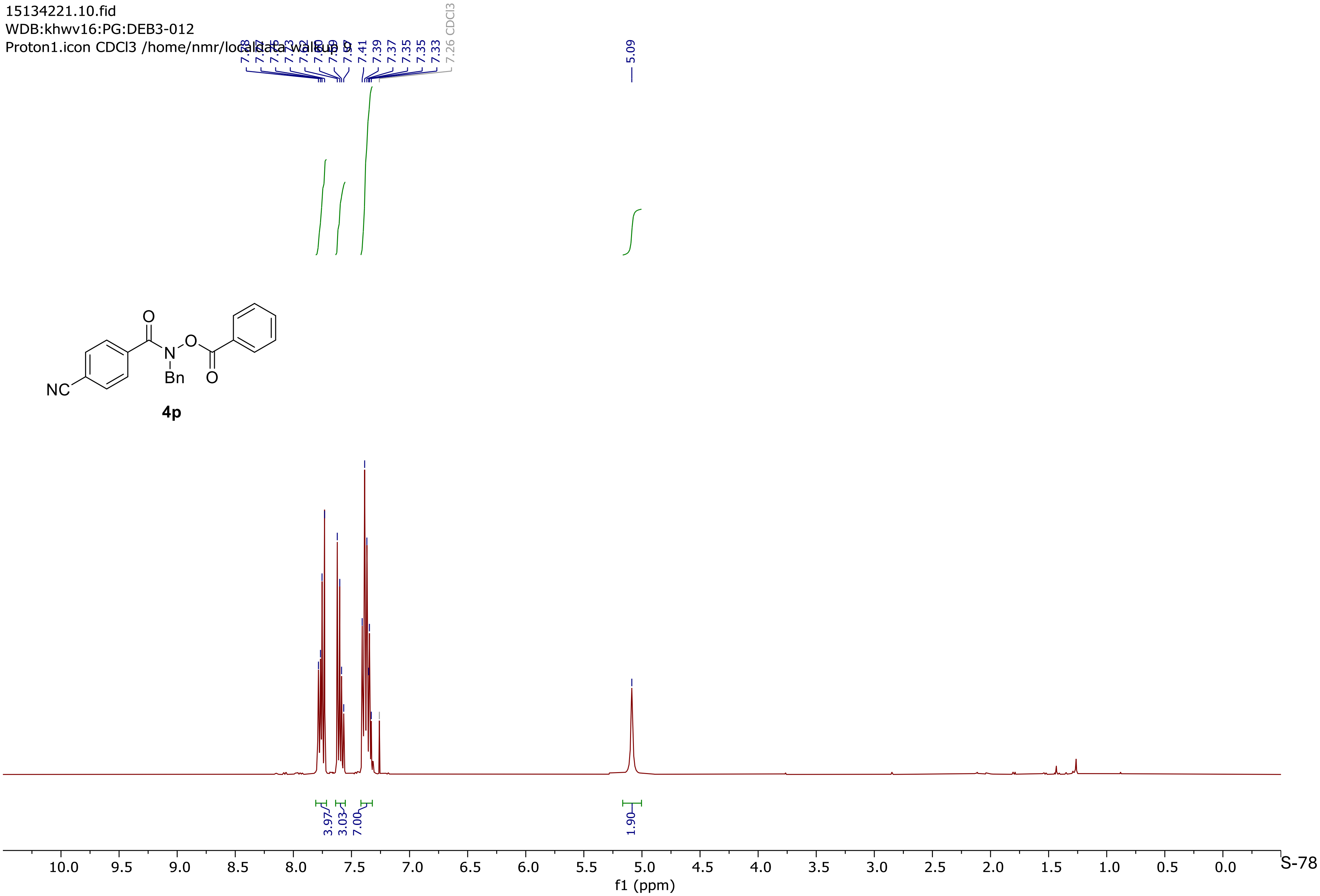

Figure S40; ¹H NMR (400 MHz, $CDCl_3$) for compound **4p**.

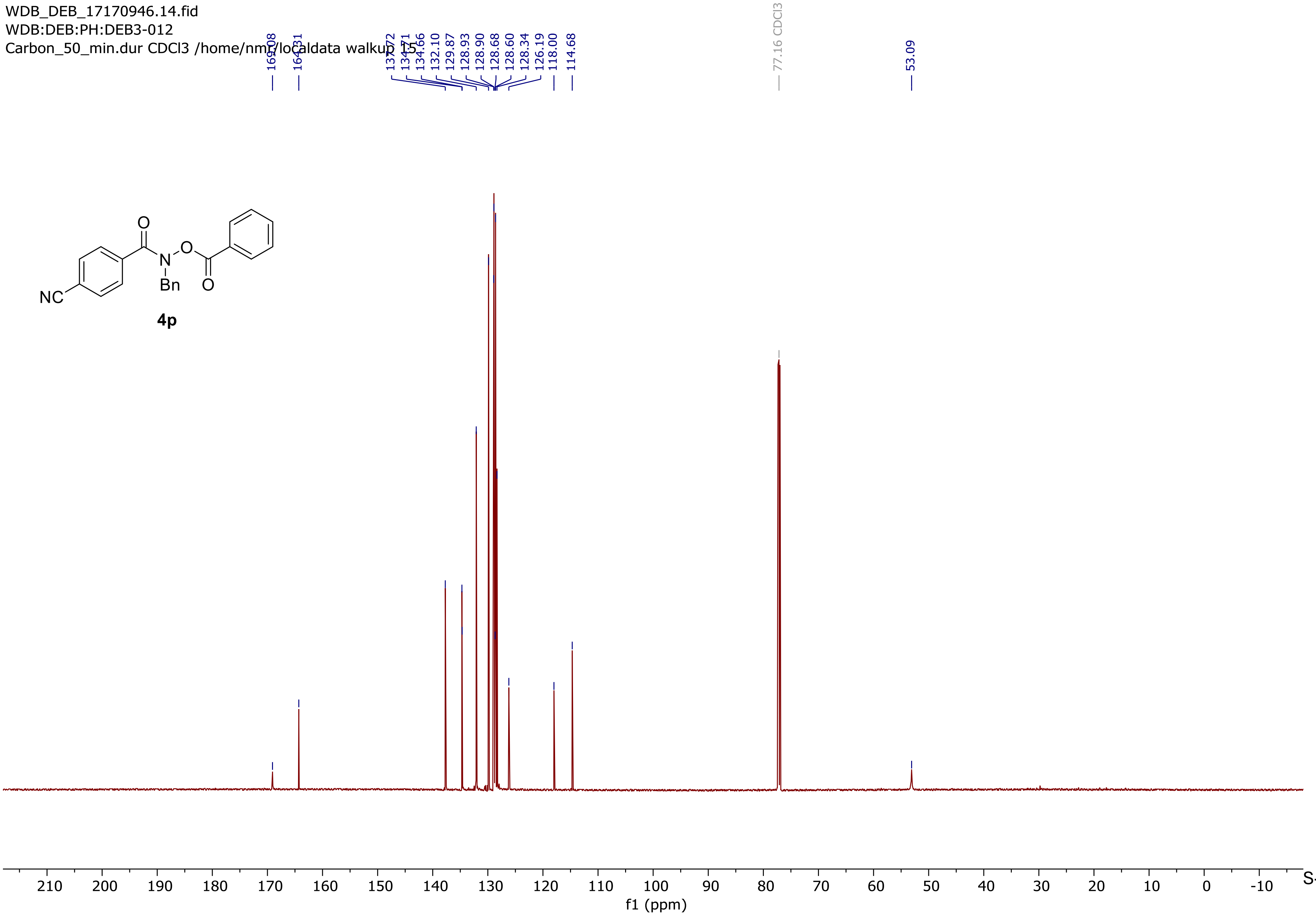

Figure S41; $^{13}C\{^{1}H\}$ NMR (176 MHz, $CDCl_3$) for compound **4p**.

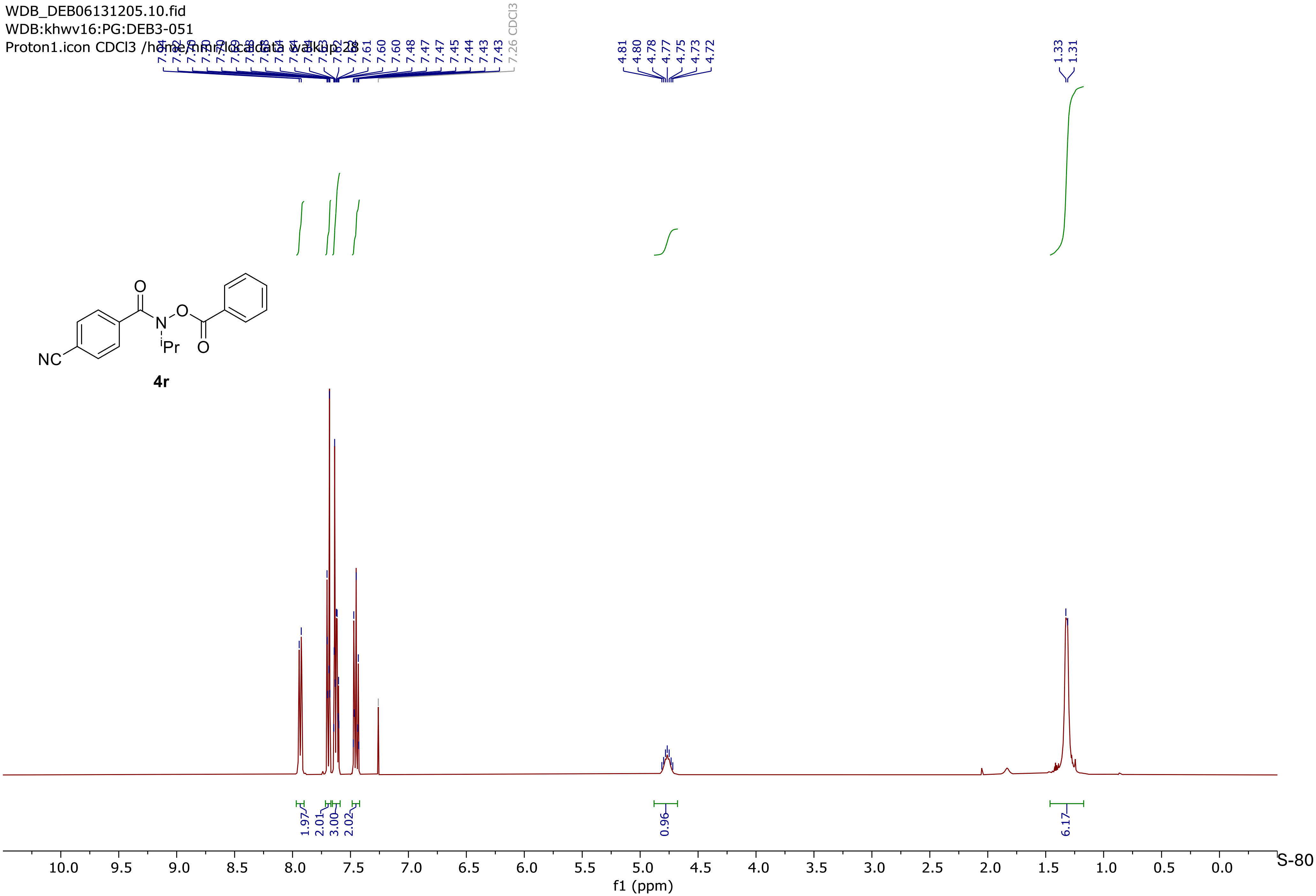

Figure S42; ^{1}H NMR (400 MHz, $CDCl_3$) for compound **4r**.

WDB_DEB06131205.11.fid
WDB:khwv16:PG:DEB3-051
Carbon.dur CDCl3 /home/nmr/localdata walkup 28

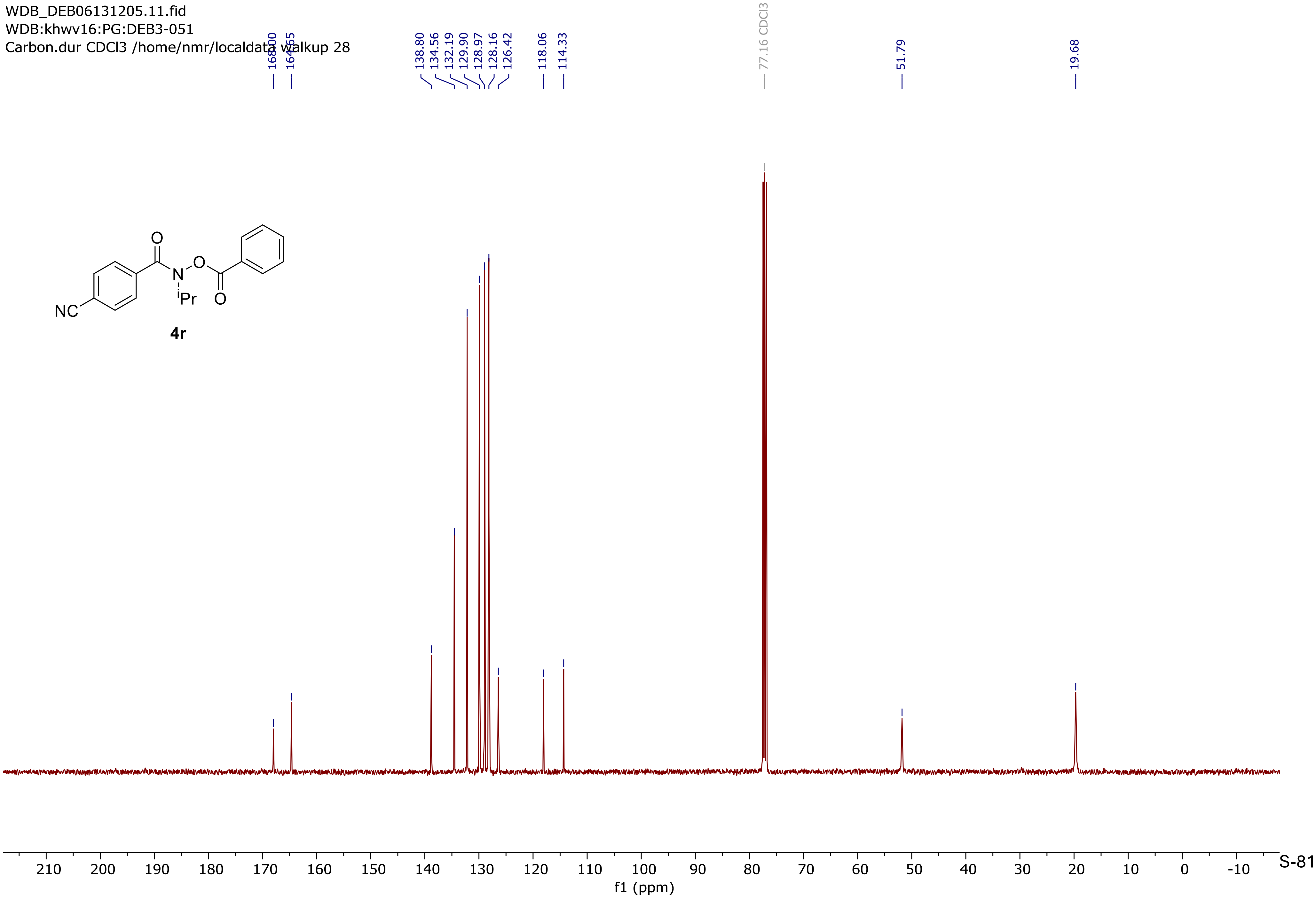

Figure S43; $^{13}C\{^1H\}$ NMR (101 MHz, $CDCl_3$) for compound **4r**.

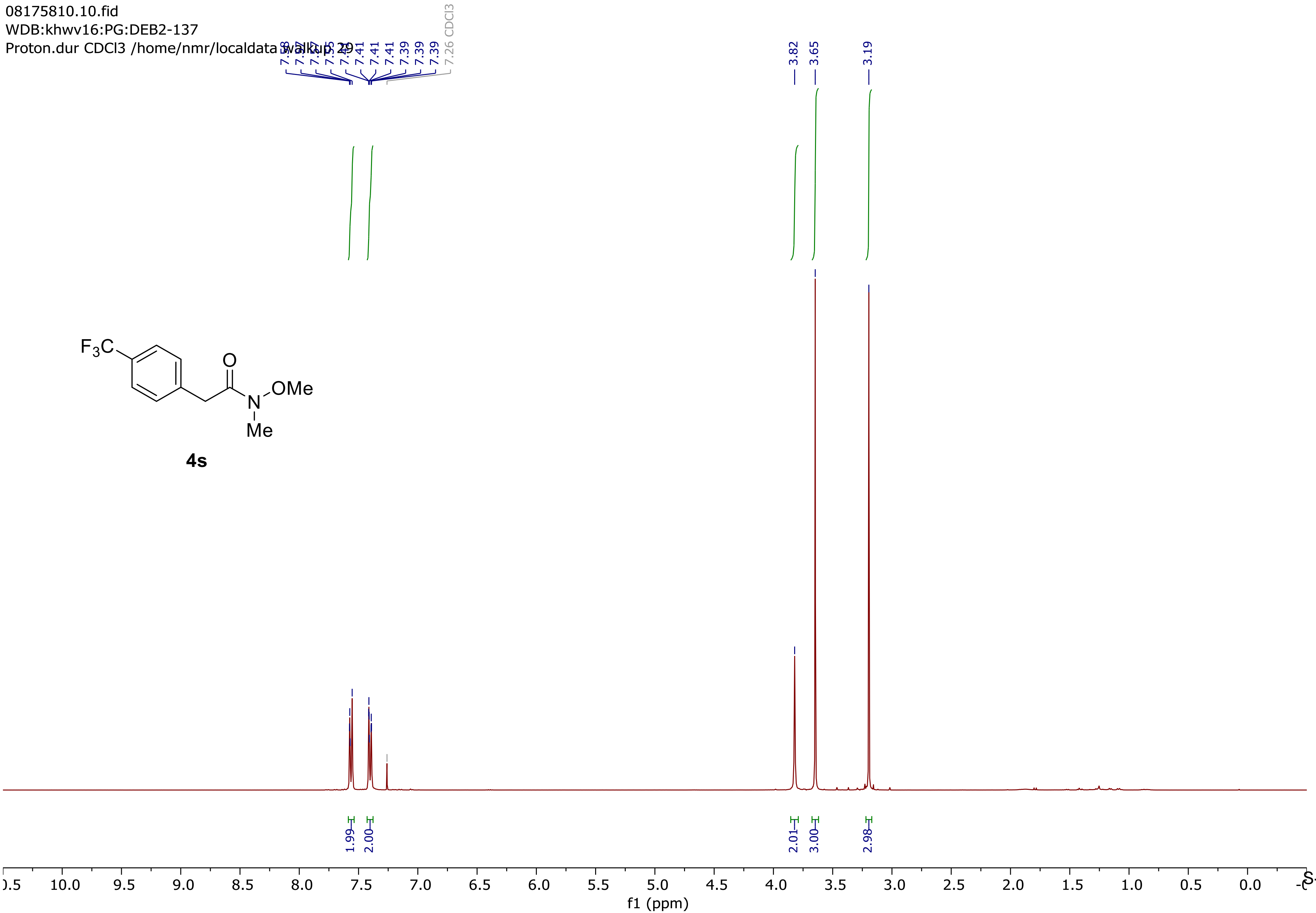

Figure S44; ^{1}H NMR (400 MHz, $CDCl_3$) for compound **4s**.

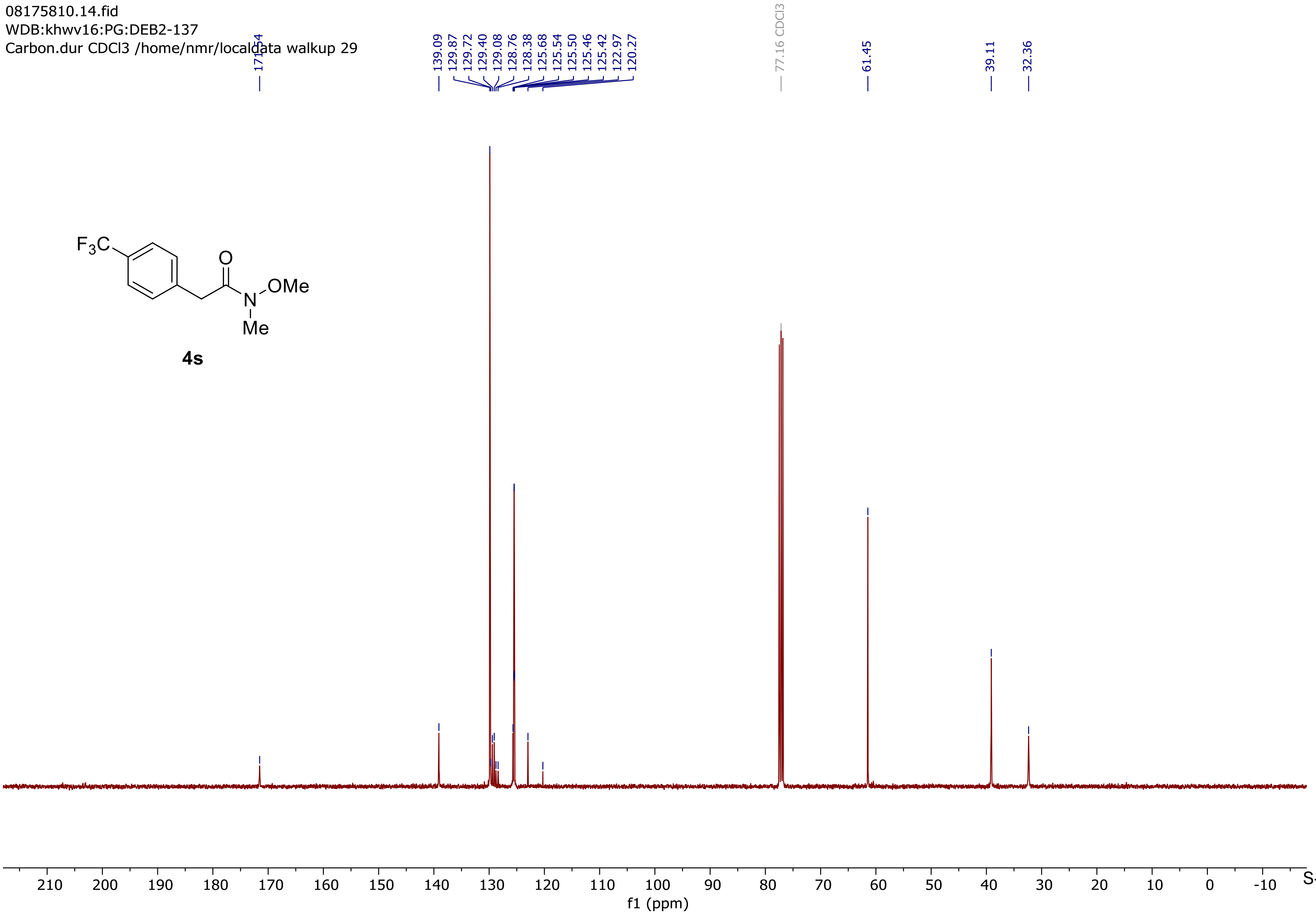

Figure S45; $^{13}C\{^1H\}$ NMR (101 MHz, $CDCl_3$) for compound **4s**.

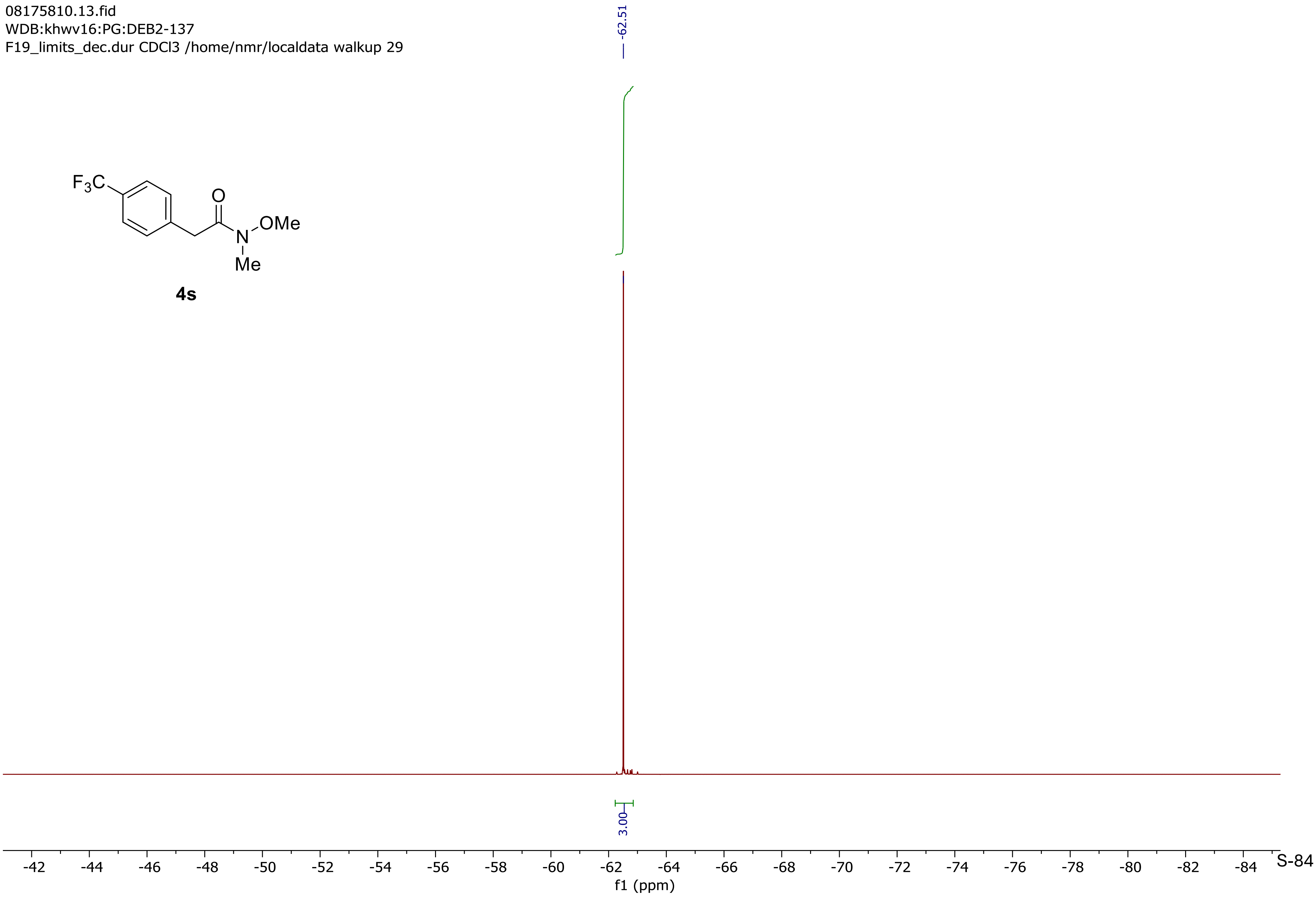

Figure S46; $^{19}F\{^1H\}$ NMR (376 MHz, $CDCl_3$) for compound **4s**.

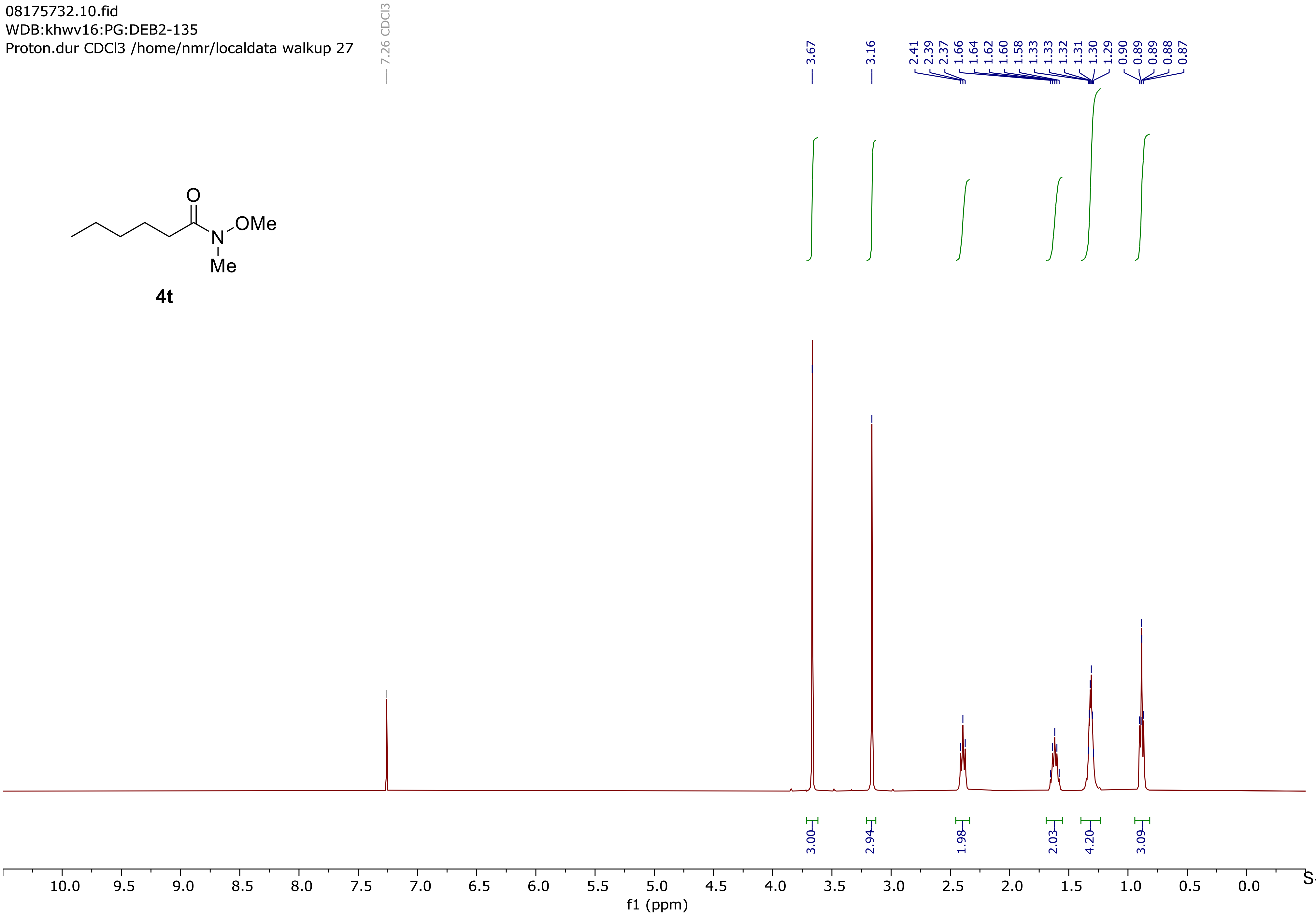

Figure S47; ^{1}H NMR (400 MHz, $CDCl_3$) for compound **4t**.

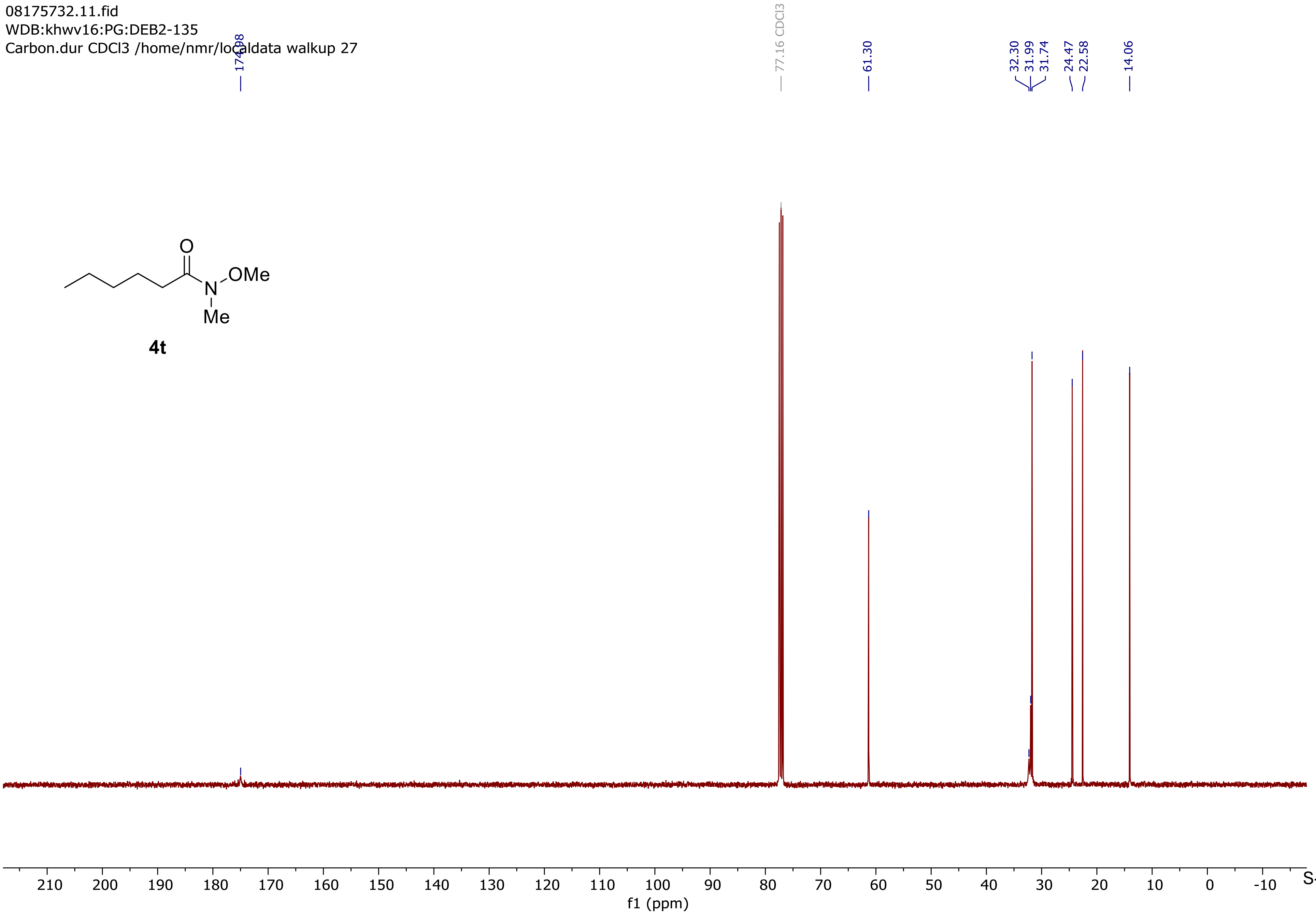

Figure S48; $^{13}C\{^1H\}$ NMR (101 MHz, $CDCl_3$) for compound **4t**.

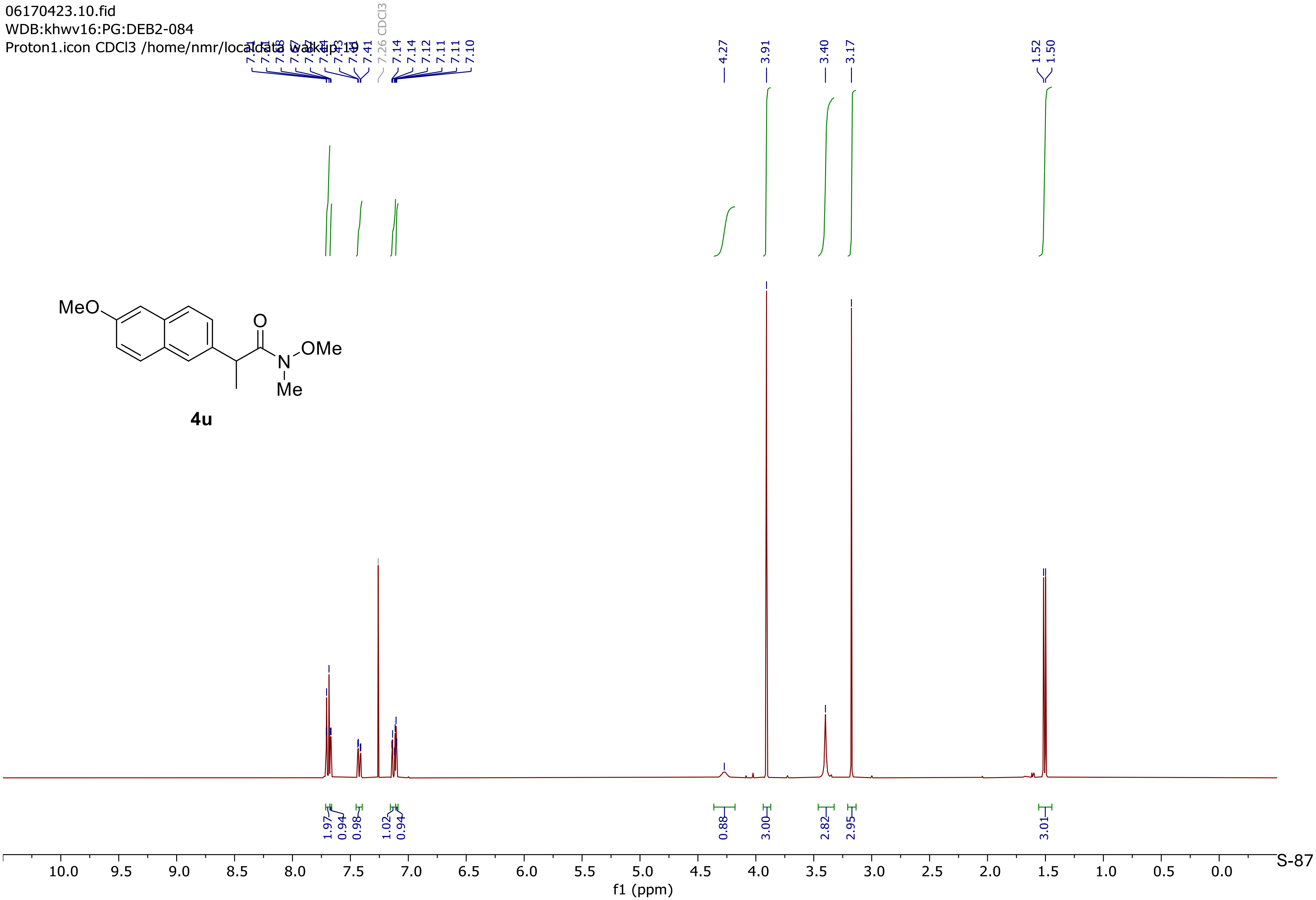

Figure S49; ^{1}H NMR (400 MHz, $CDCl_3$) for compound **4u**.

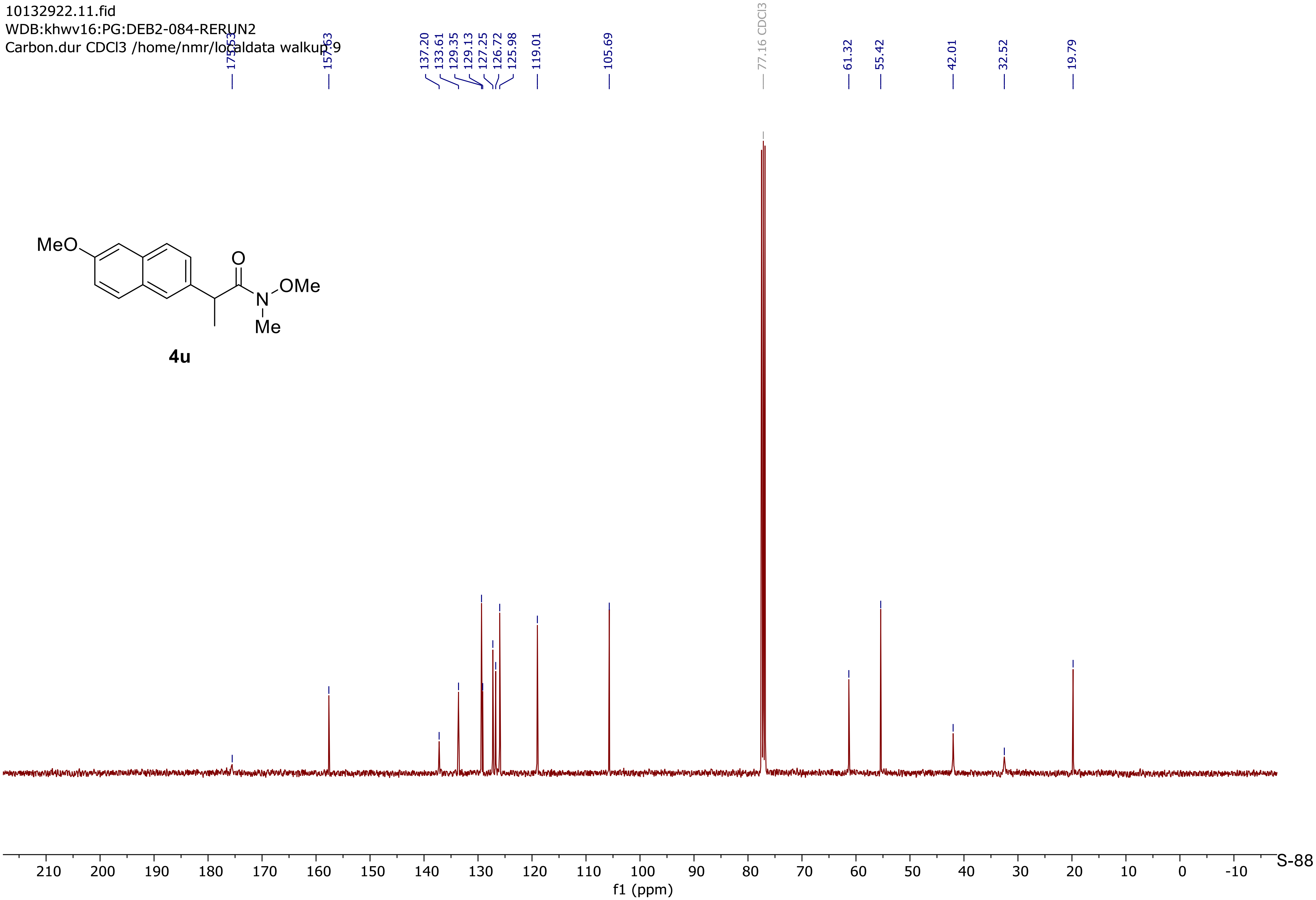

Figure S50; $^{13}C\{^{1}H\}$ NMR (101 MHz, $CDCl_3$) for compound **4u**.

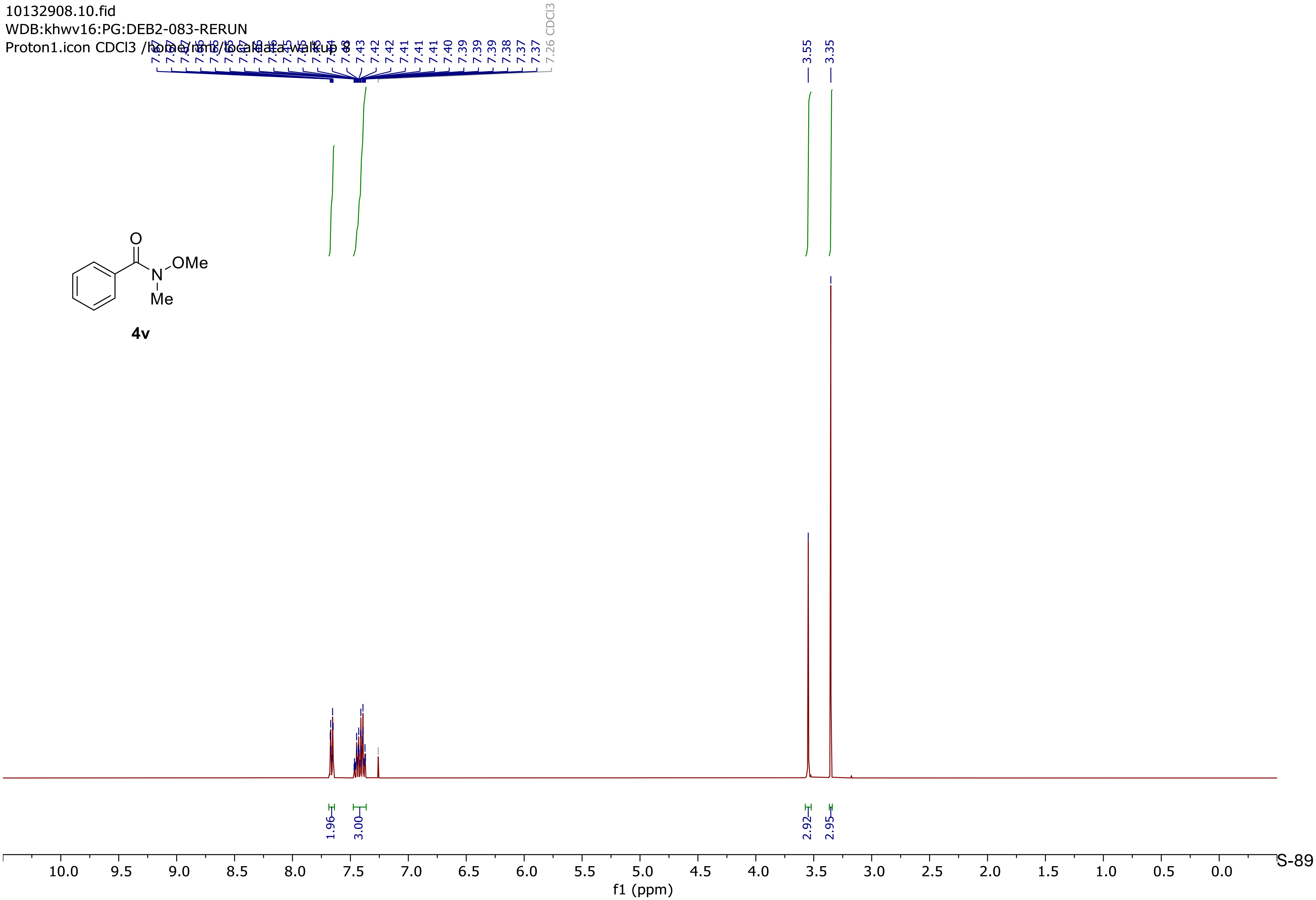

Figure S51; ^{1}H NMR (400 MHz, $CDCl_3$) for compound **4v**.

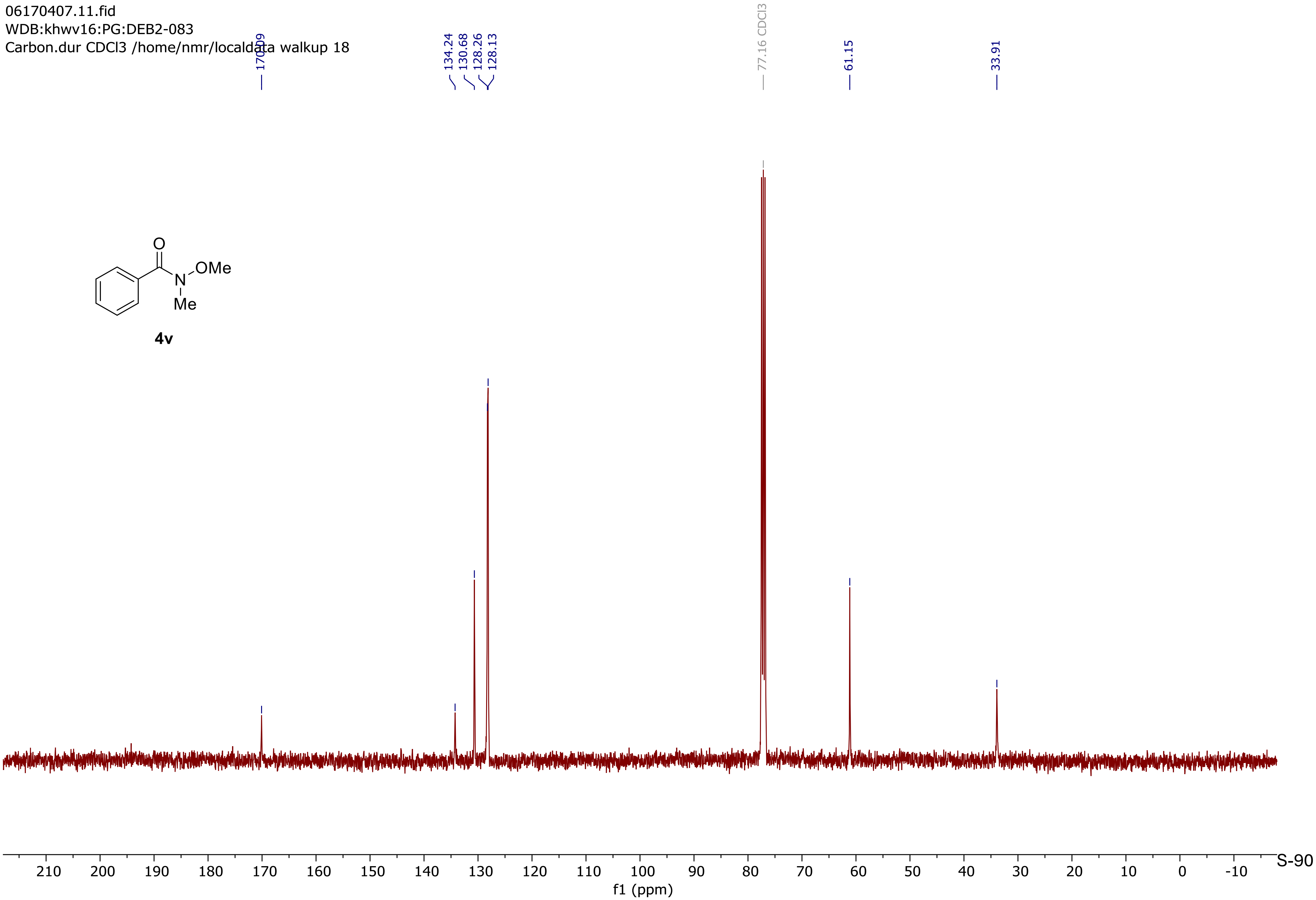

Figure S52; $^{13}C\{^{1}H\}$ NMR (101 MHz, $CDCl_3$) for compound **4v**.

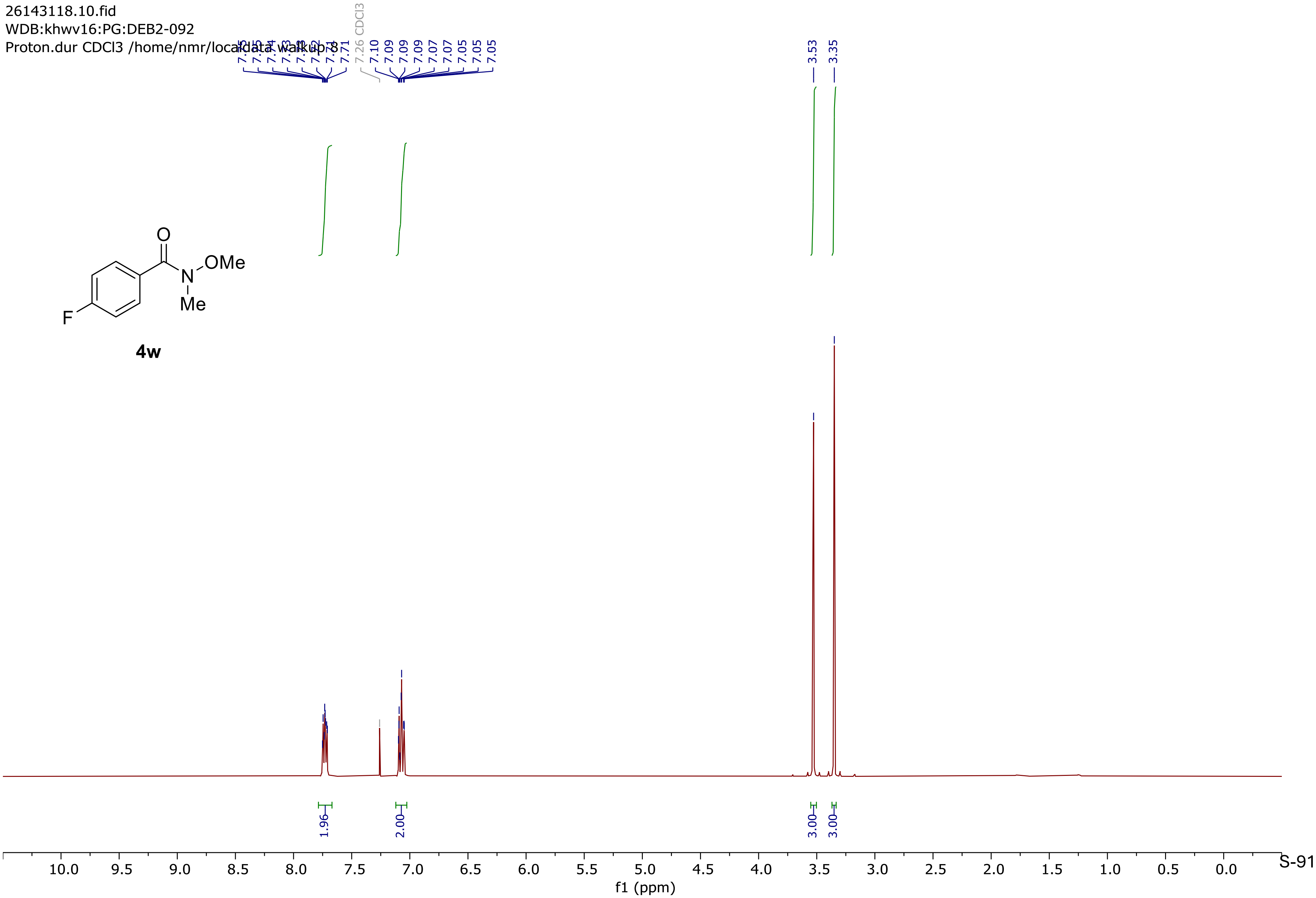

Figure S53; ^{1}H NMR (400 MHz, $CDCl_3$) for compound **4w**.

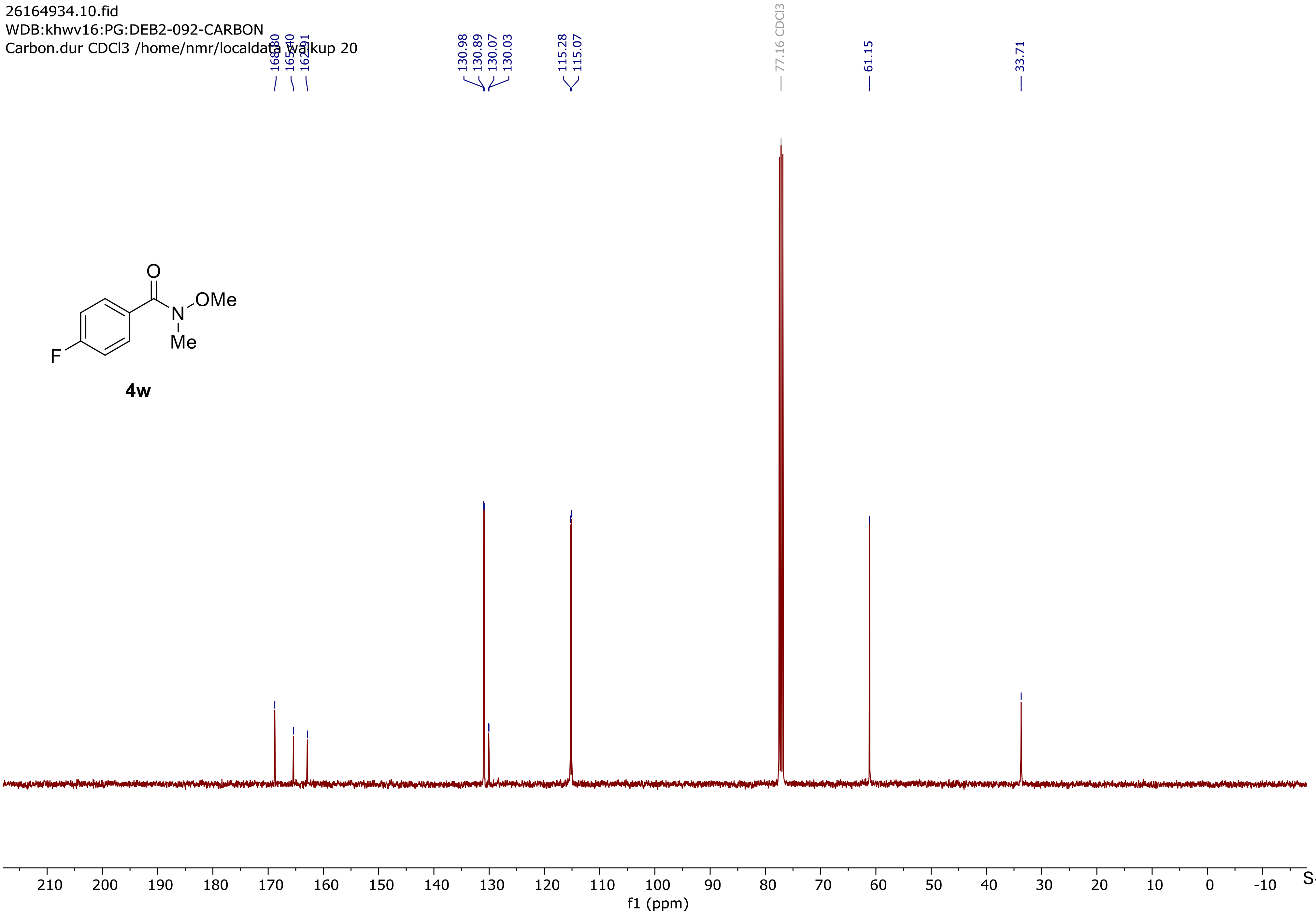

Figure S54; $^{13}C\{^{1}H\}$ NMR (101 MHz, $CDCl_3$) for compound **4w**.

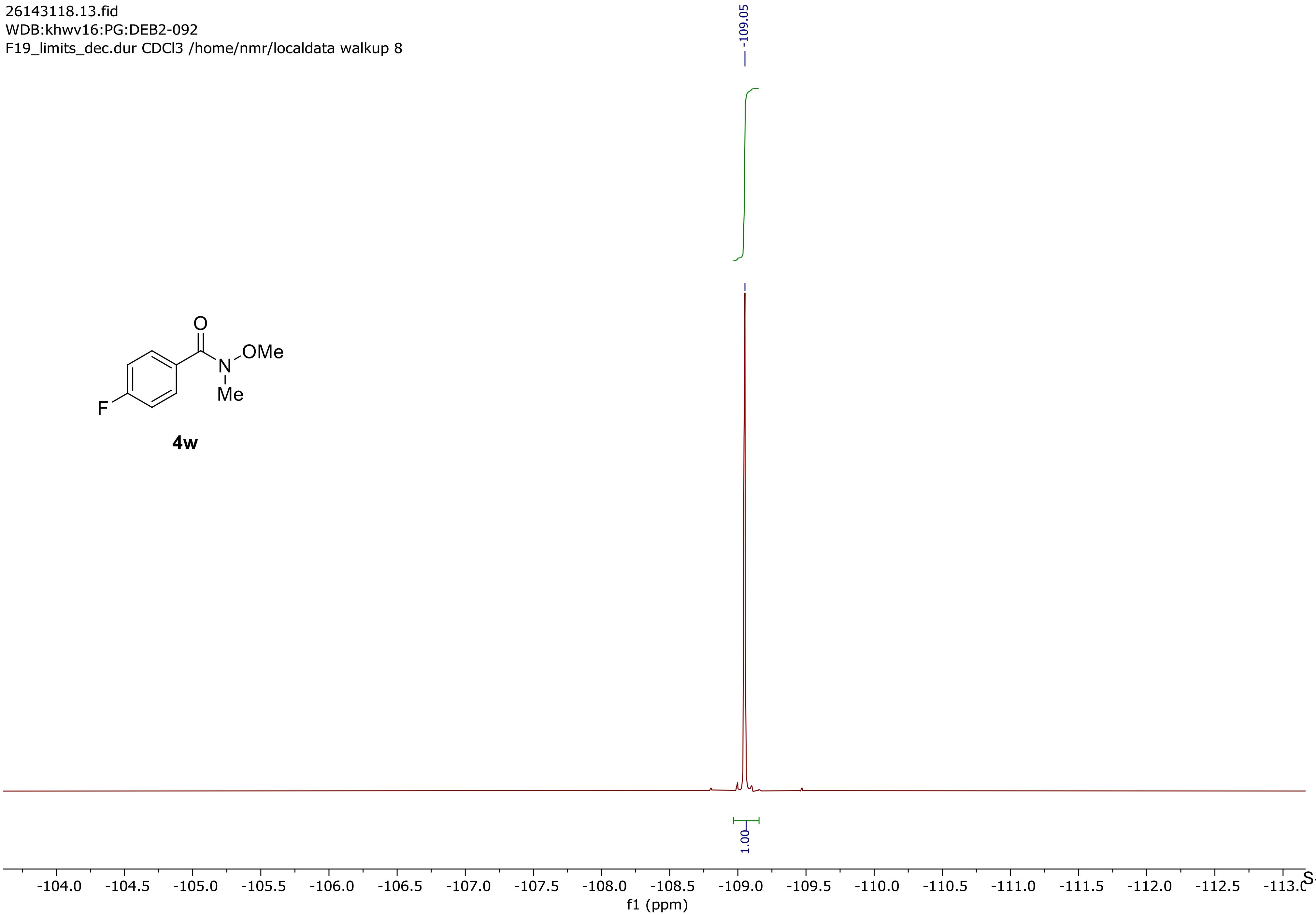

4w

Figure S55; $^{19}F\{^{1}H\}$ NMR (376 MHz, $CDCl_3$) for compound **4w**.

26124409.10.fid
WDB:khwv16:PG:DEB2-093
Proton1.icon CDCl3 /home/nmr/localdata walkup 7

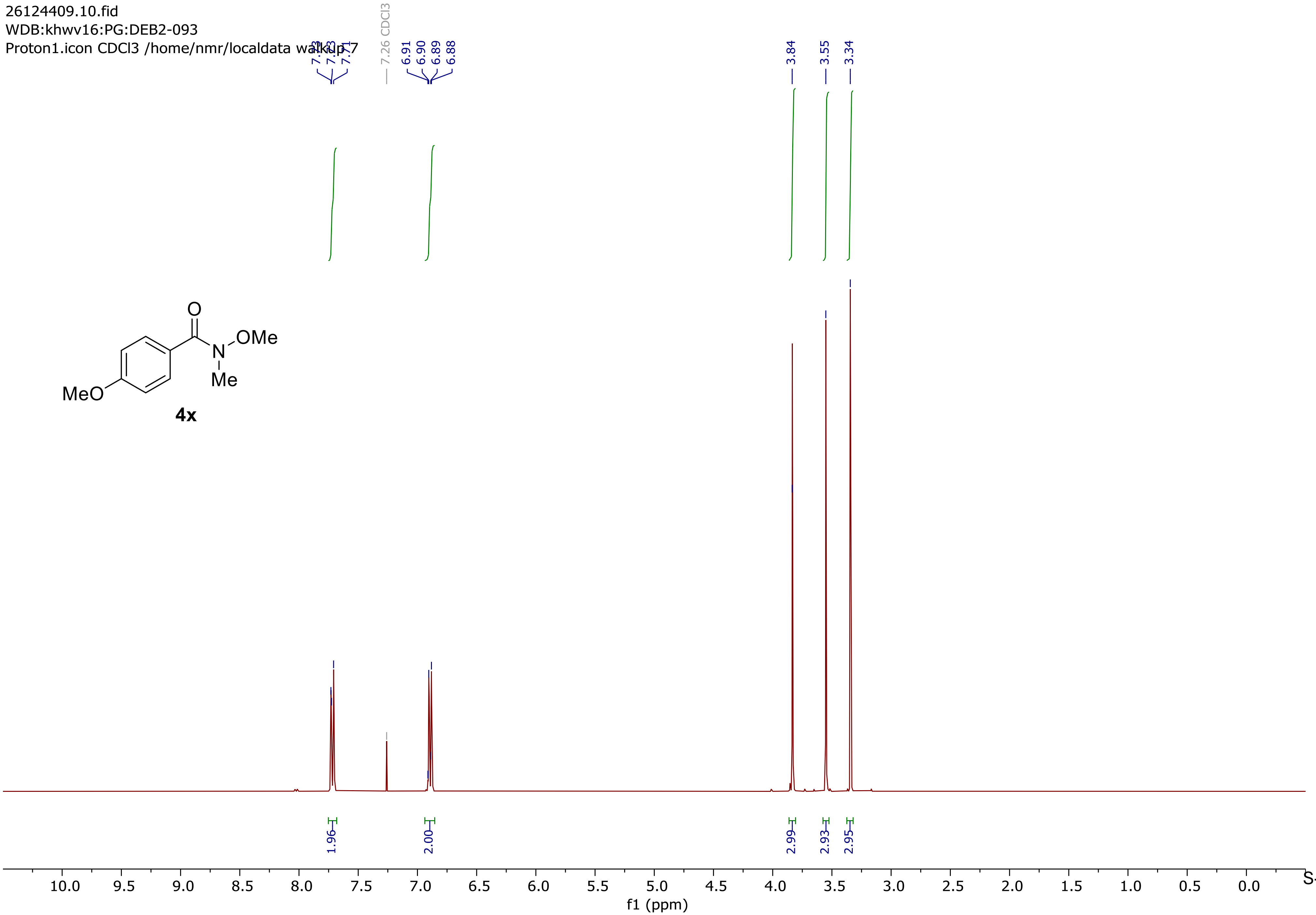

Figure S56; ^{1}H NMR (400 MHz, $CDCl_3$) for compound **4x**.

26124409.11.fid
WDB:khwv16:PG:DEB2-093
Carbon.dur CDCl3 /home/nmr/localdata walkup 7

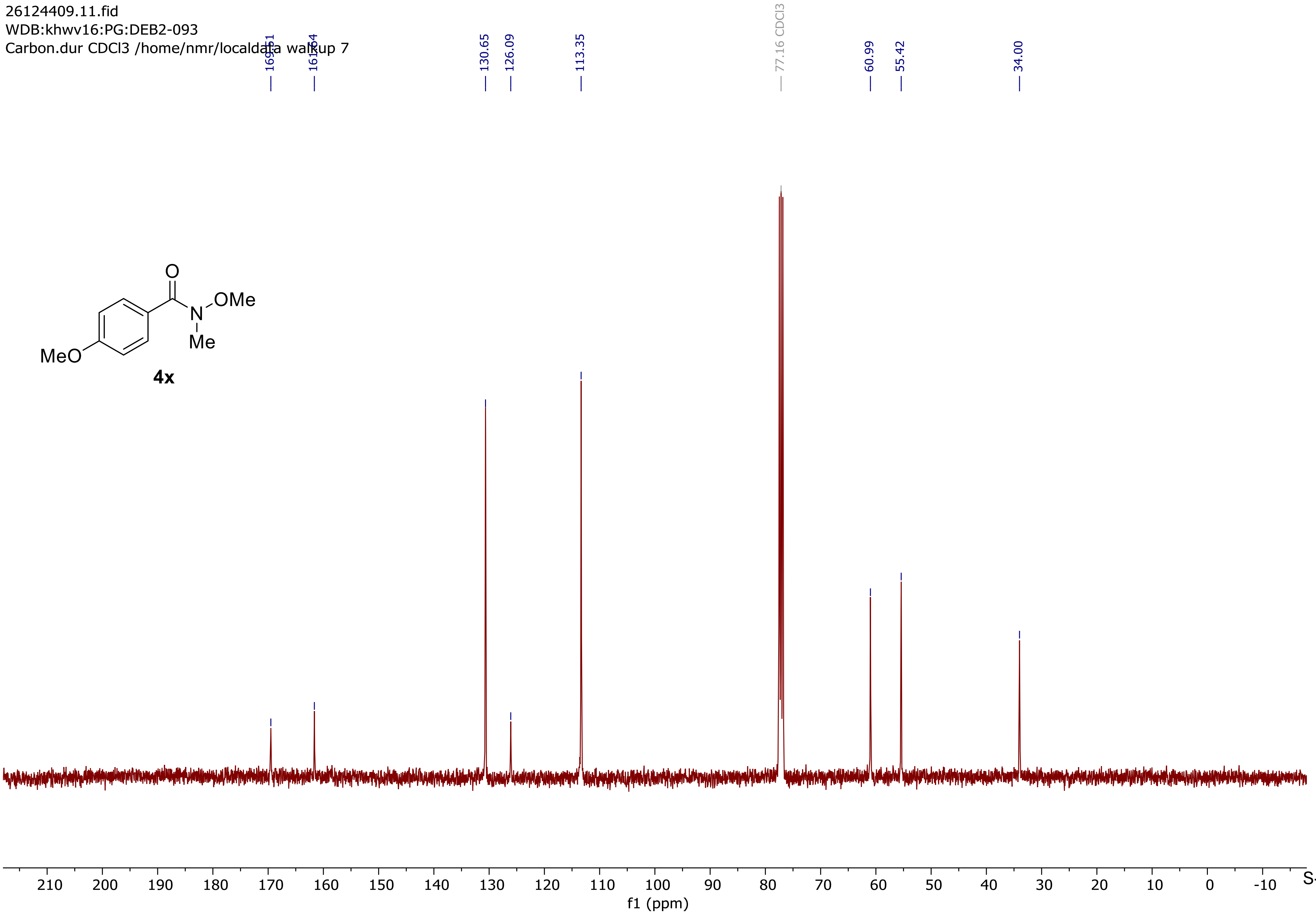

Figure S57; $^{13}C\{^1H\}$ NMR (101 MHz, $CDCl_3$) for compound **4x**.

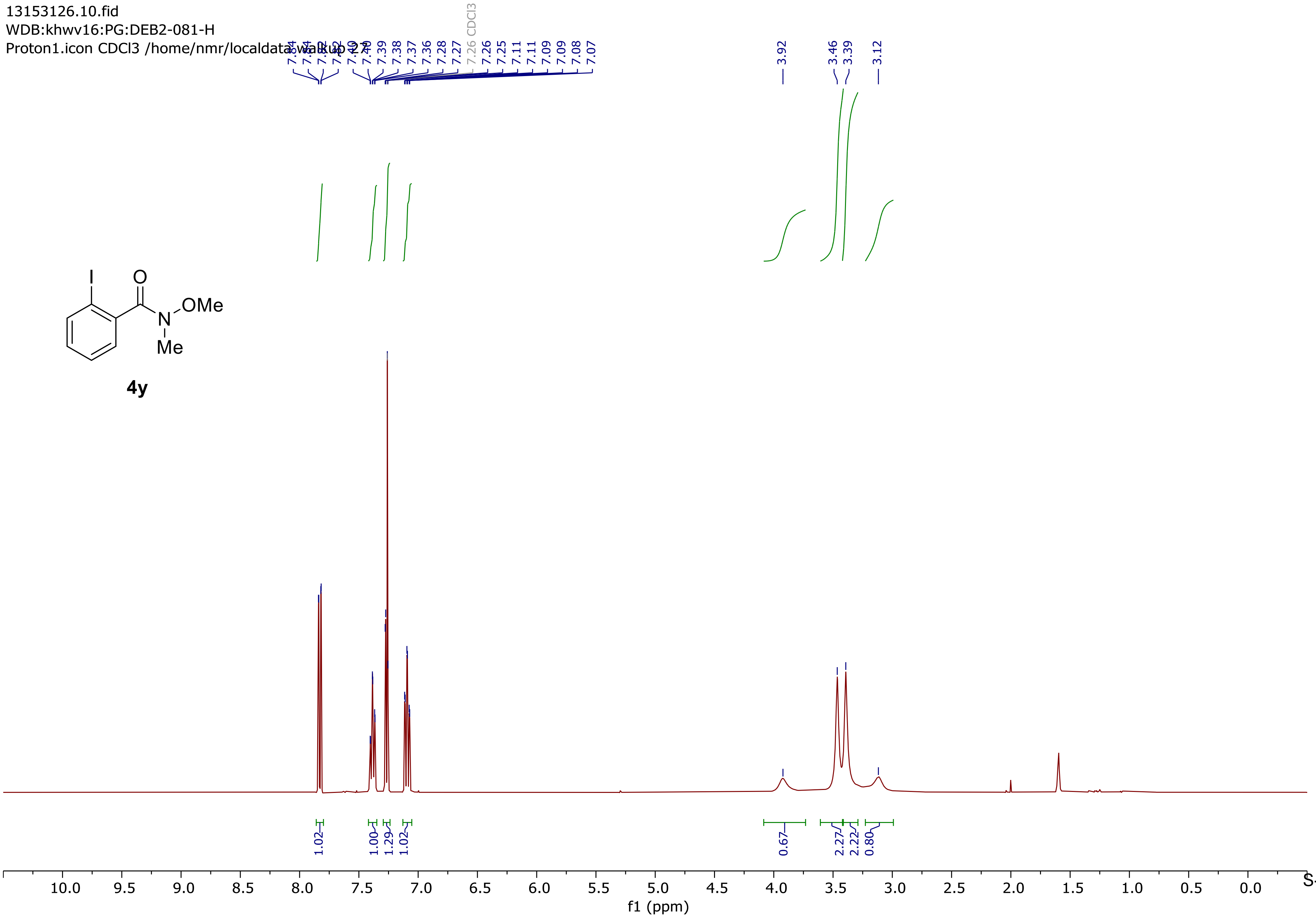

Figure S58; ^{1}H NMR (400 MHz, $CDCl_3$) for compound **4y**.

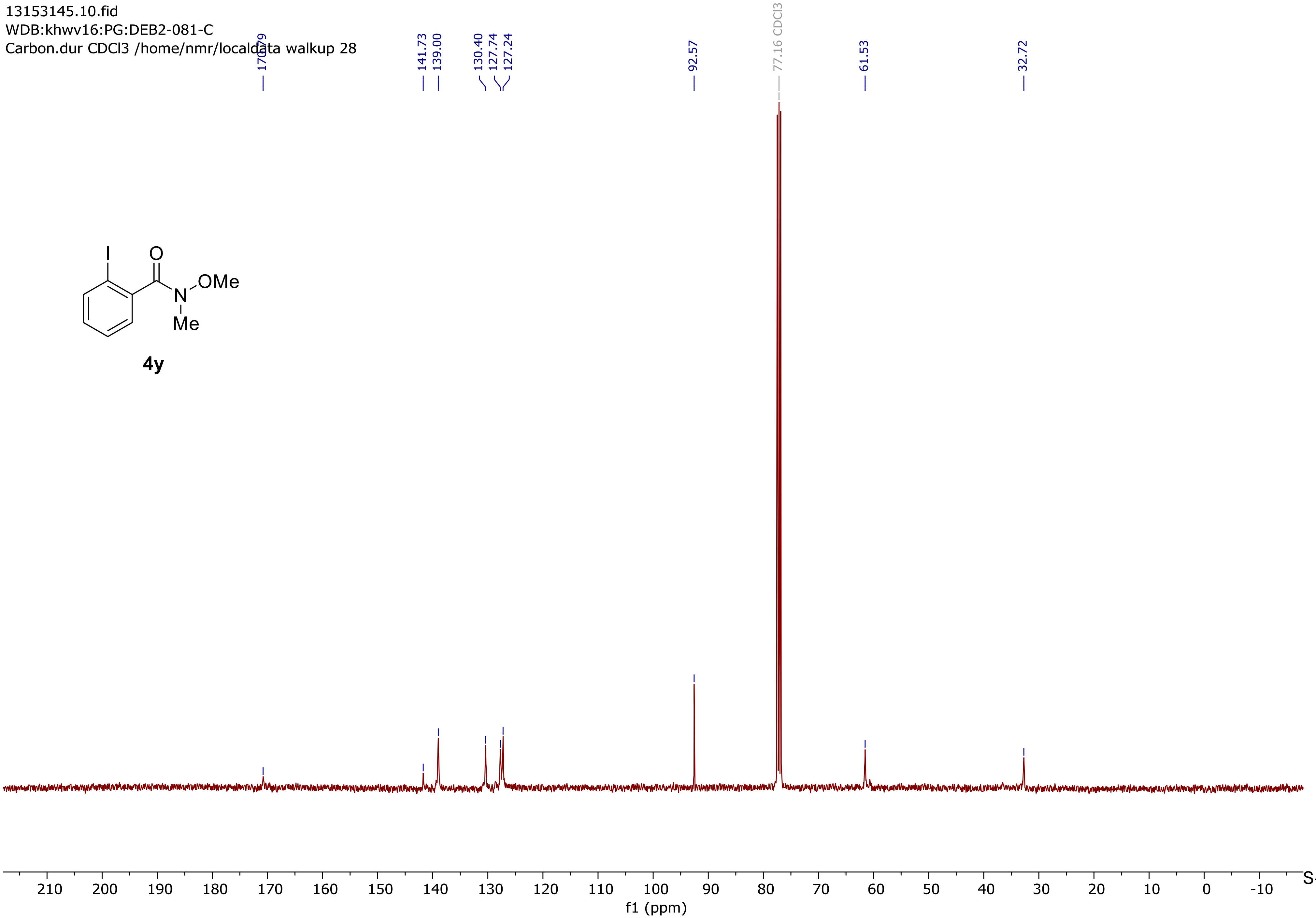

Figure S59; $^{13}C\{^{1}H\}$ NMR (101 MHz, $CDCl_3$) for compound **4y**.

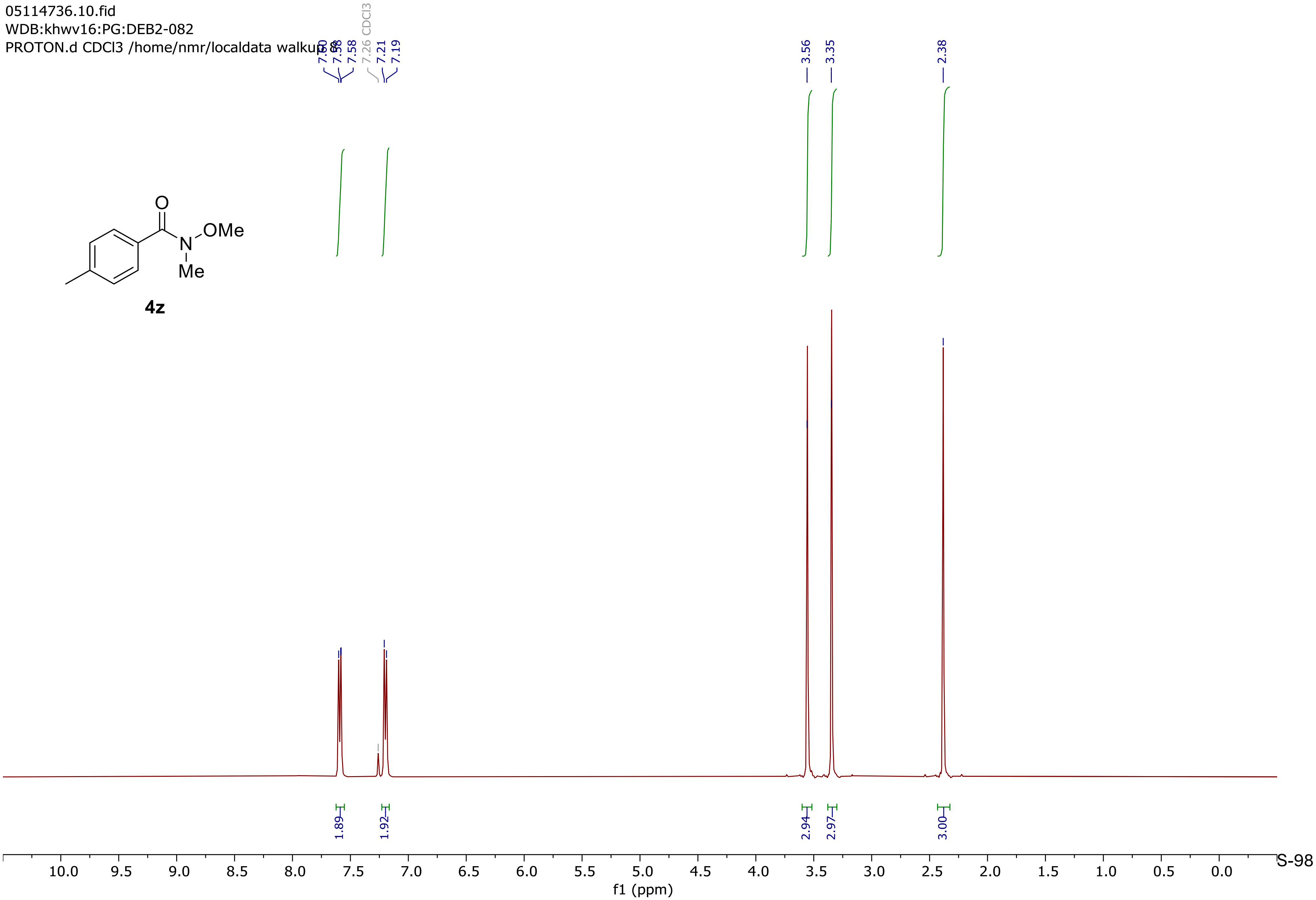

Figure S60; ^{1}H NMR (400 MHz, $CDCl_3$) for compound **4z**.

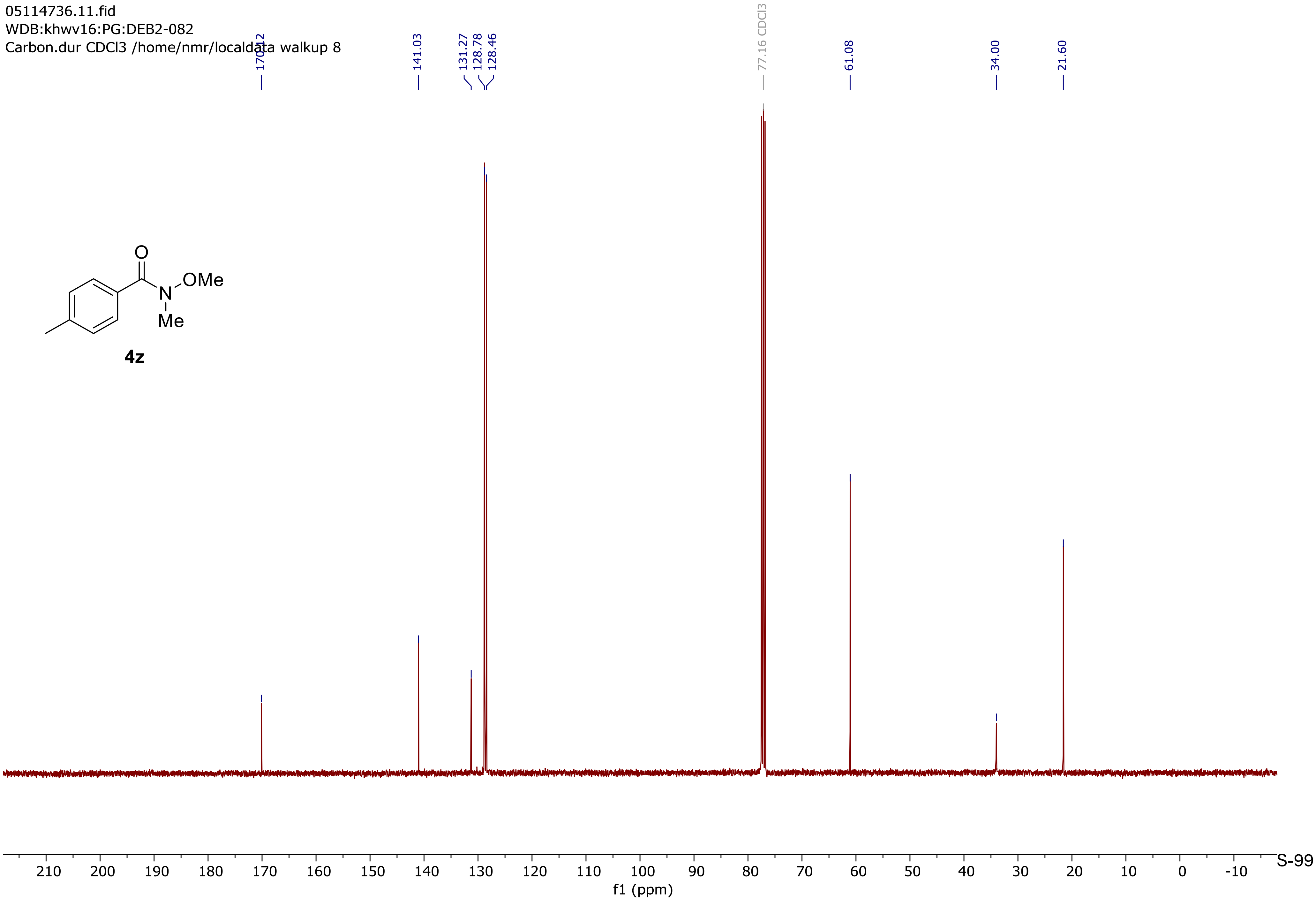

Figure S61; $^{13}C\{^{1}H\}$ NMR (101 MHz, $CDCl_3$) for compound **4z**.

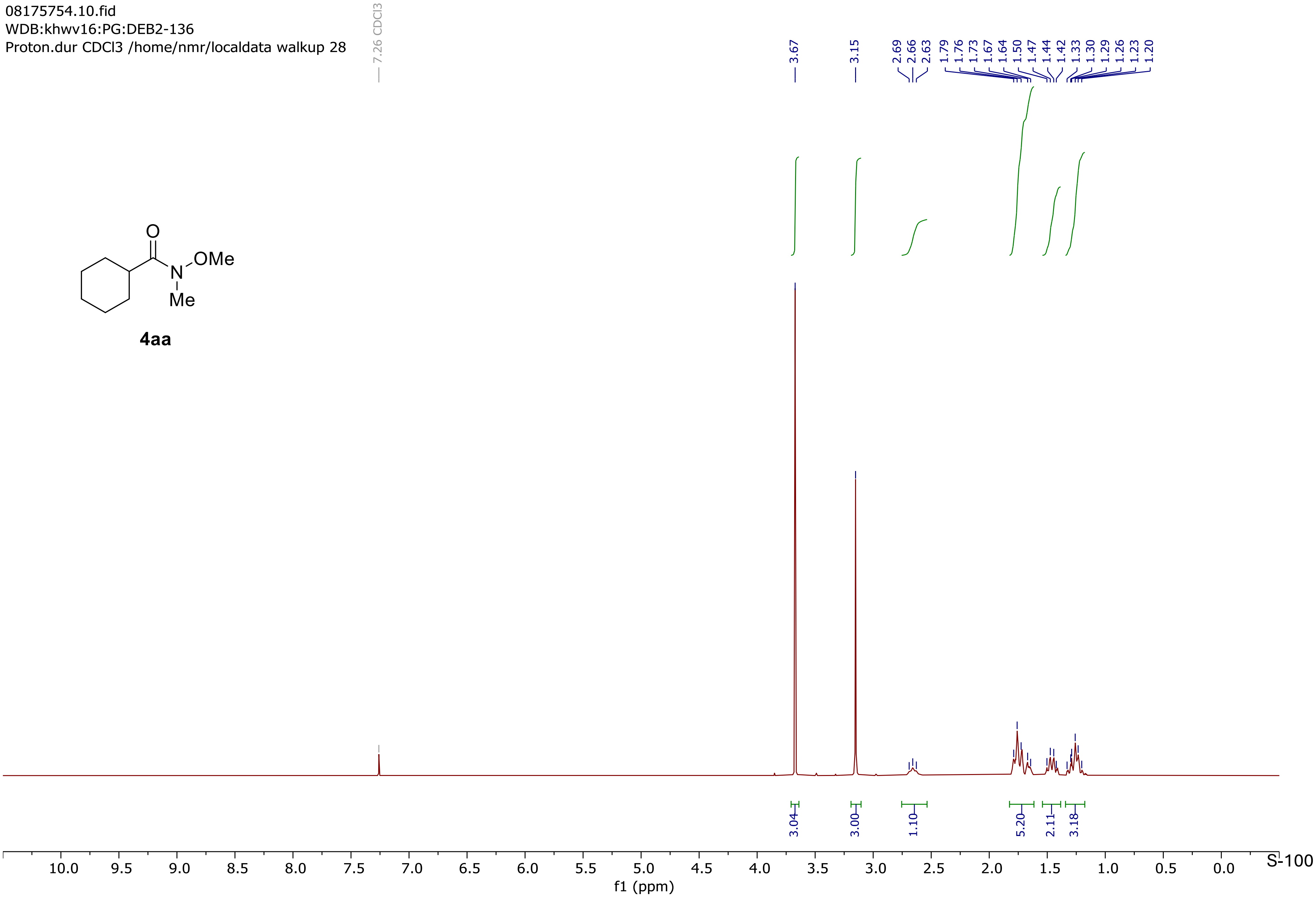

4aa

Figure S62; ^{1}H NMR (400 MHz, $CDCl_3$) for compound **4aa**.

08175754.11.fid
WDB:khwv16:PG:DEB2-136
Carbon.dur CDCl3 /home/nmr/localdata walkup 28

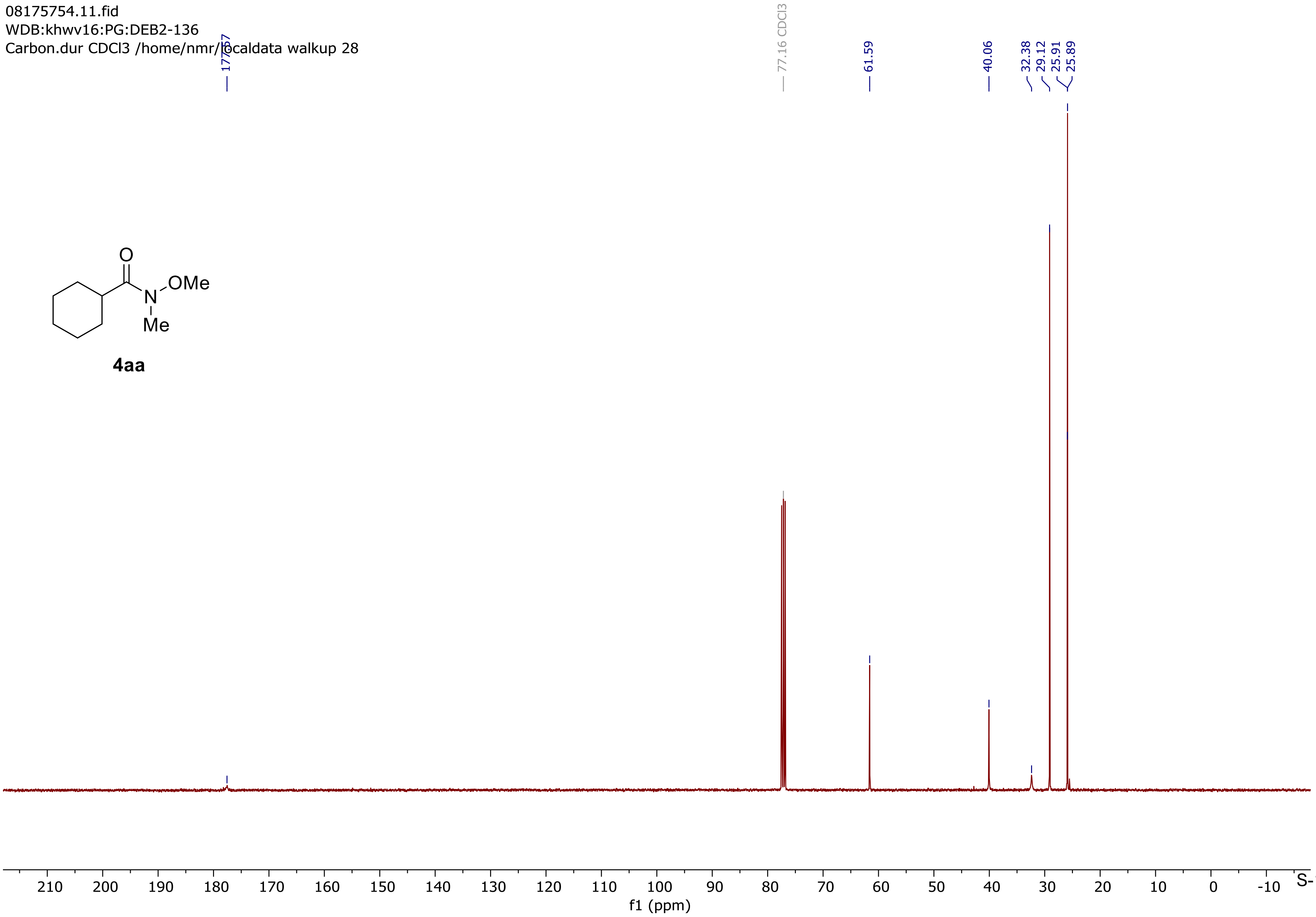

4aa

Figure S63; $^{13}C\{^{1}H\}$ NMR (101 MHz, $CDCl_3$) for compound **4aa**.

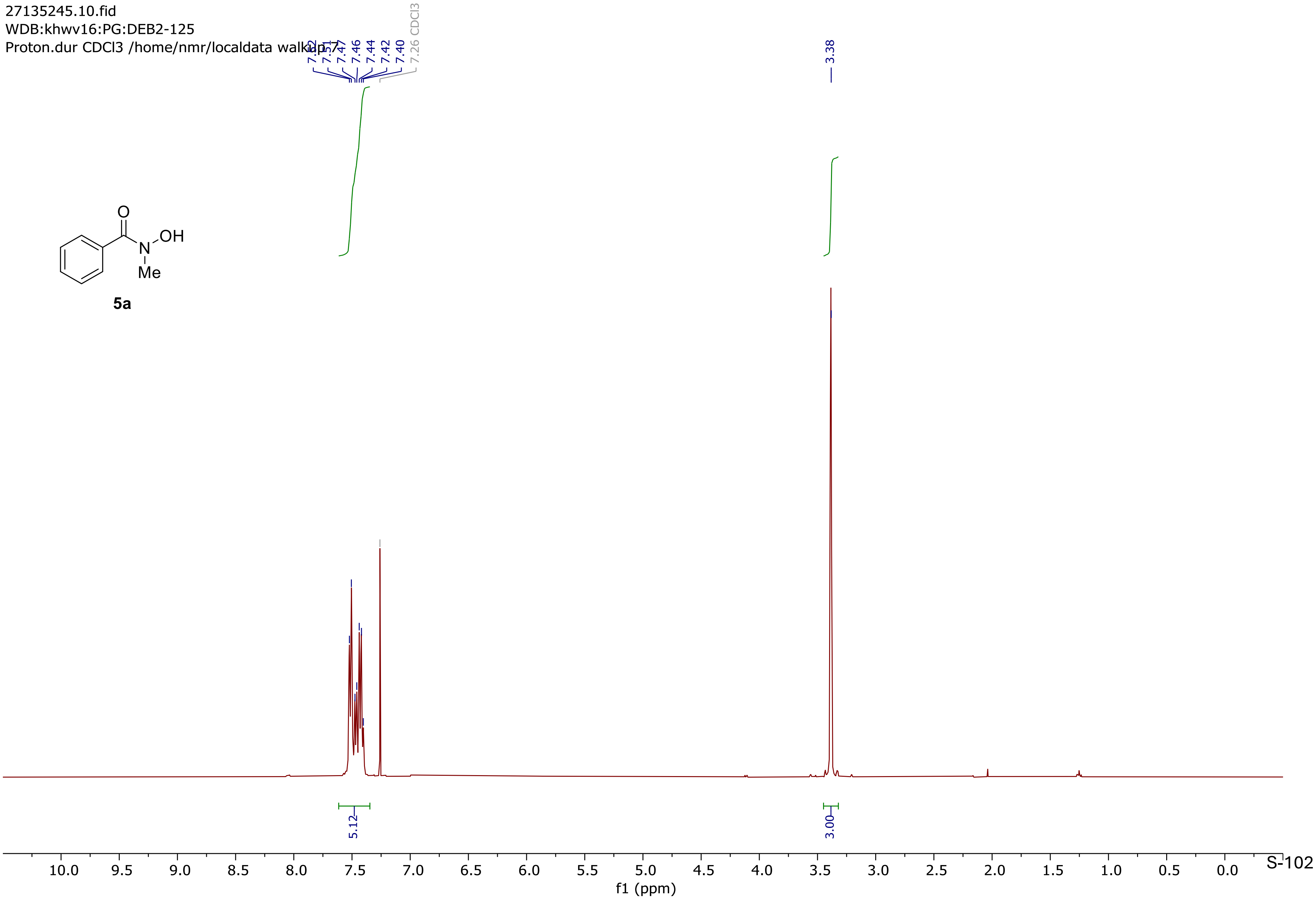

Figure S64; ^{1}H NMR (400 MHz, $CDCl_3$) for compound **5a**.

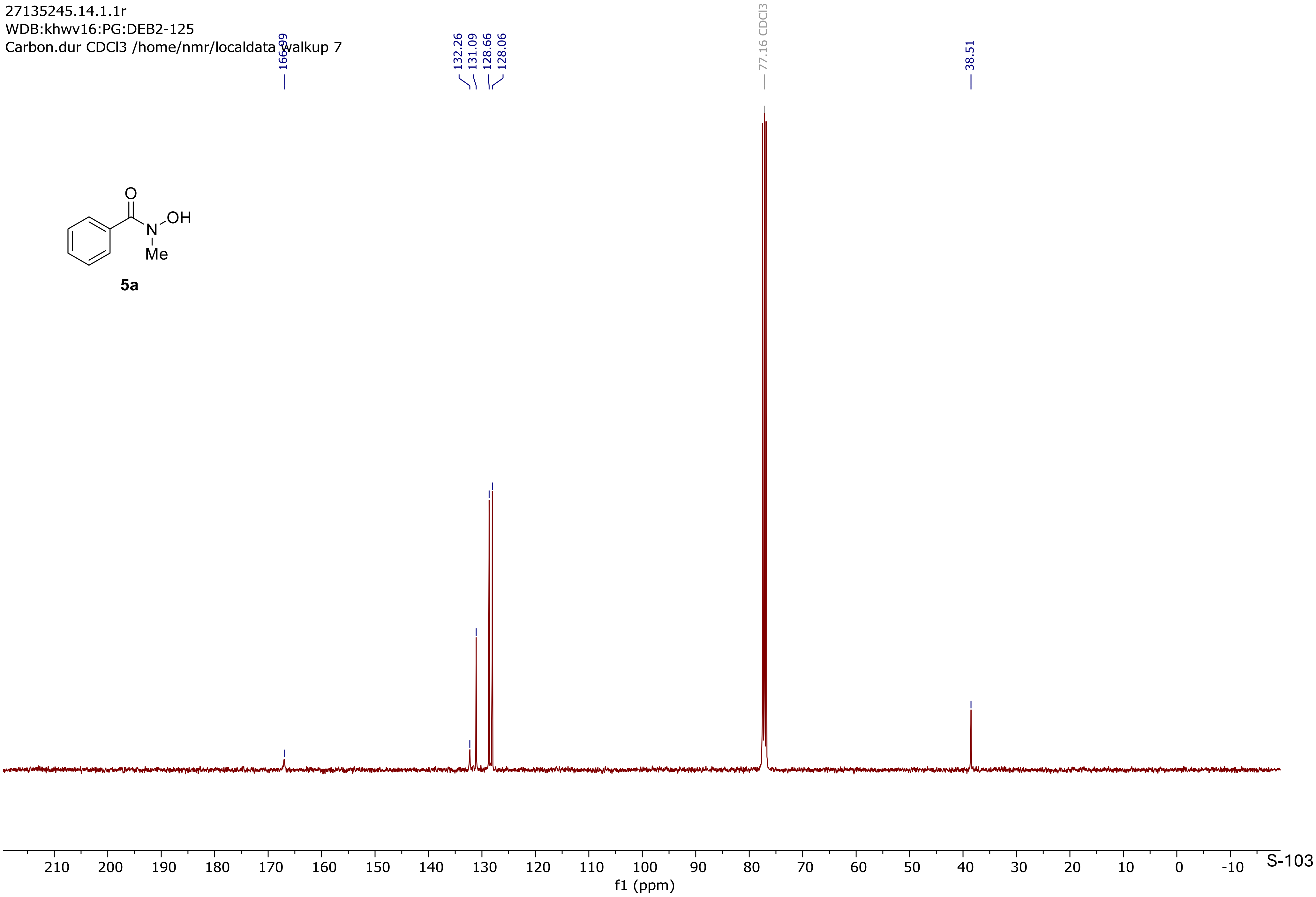

Figure S65; $^{13}C\{^1H\}$ NMR (101 MHz, $CDCl_3$) for compound **5a**.

07163727.10.fid
WDB:khwv16:PG:DEB2-138-DILUTE
Proton.dur CDCl3 /home/nmr/localdata walkup 20

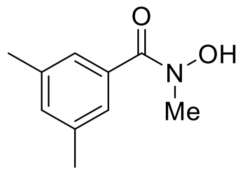

5b

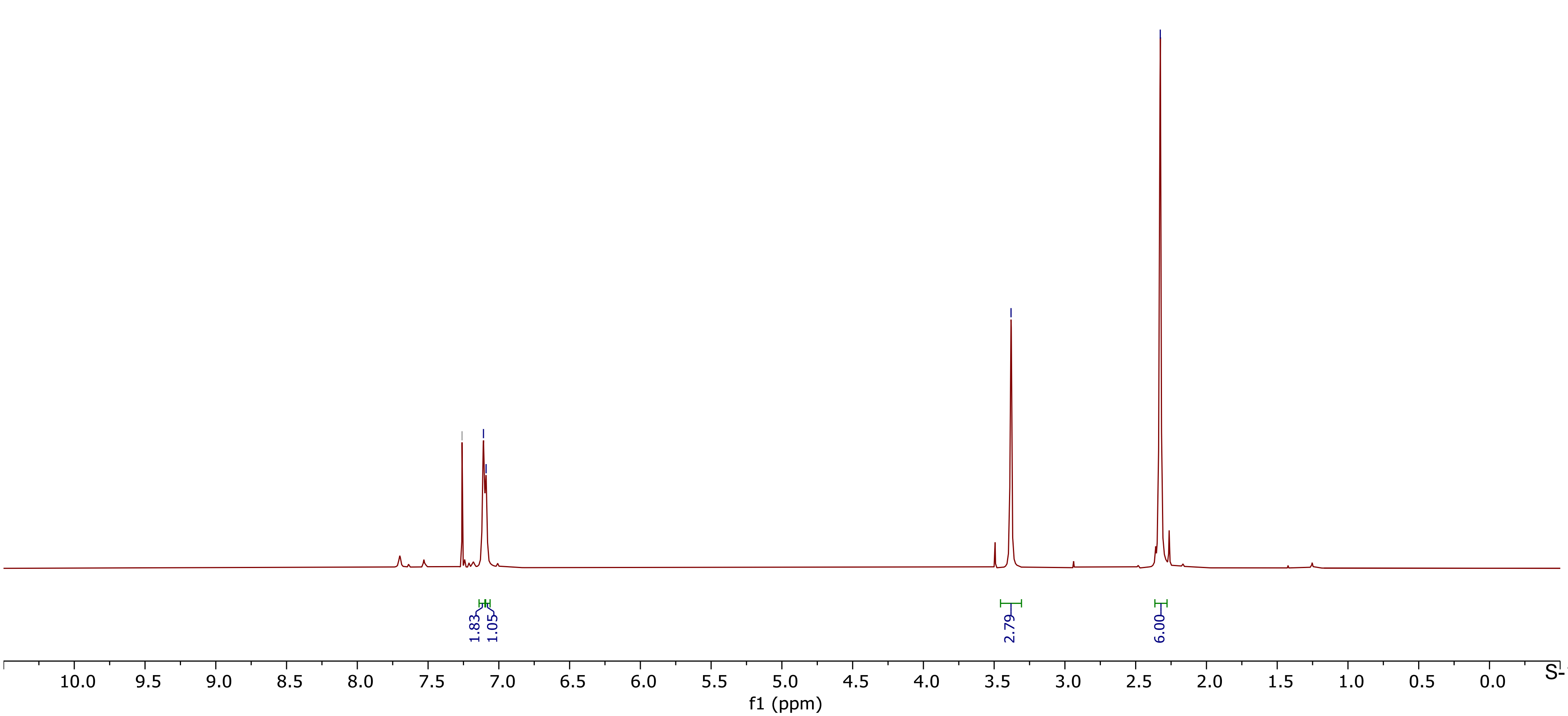

Figure S66; ^{1}H NMR (400 MHz, $CDCl_3$) for compound **5b**.

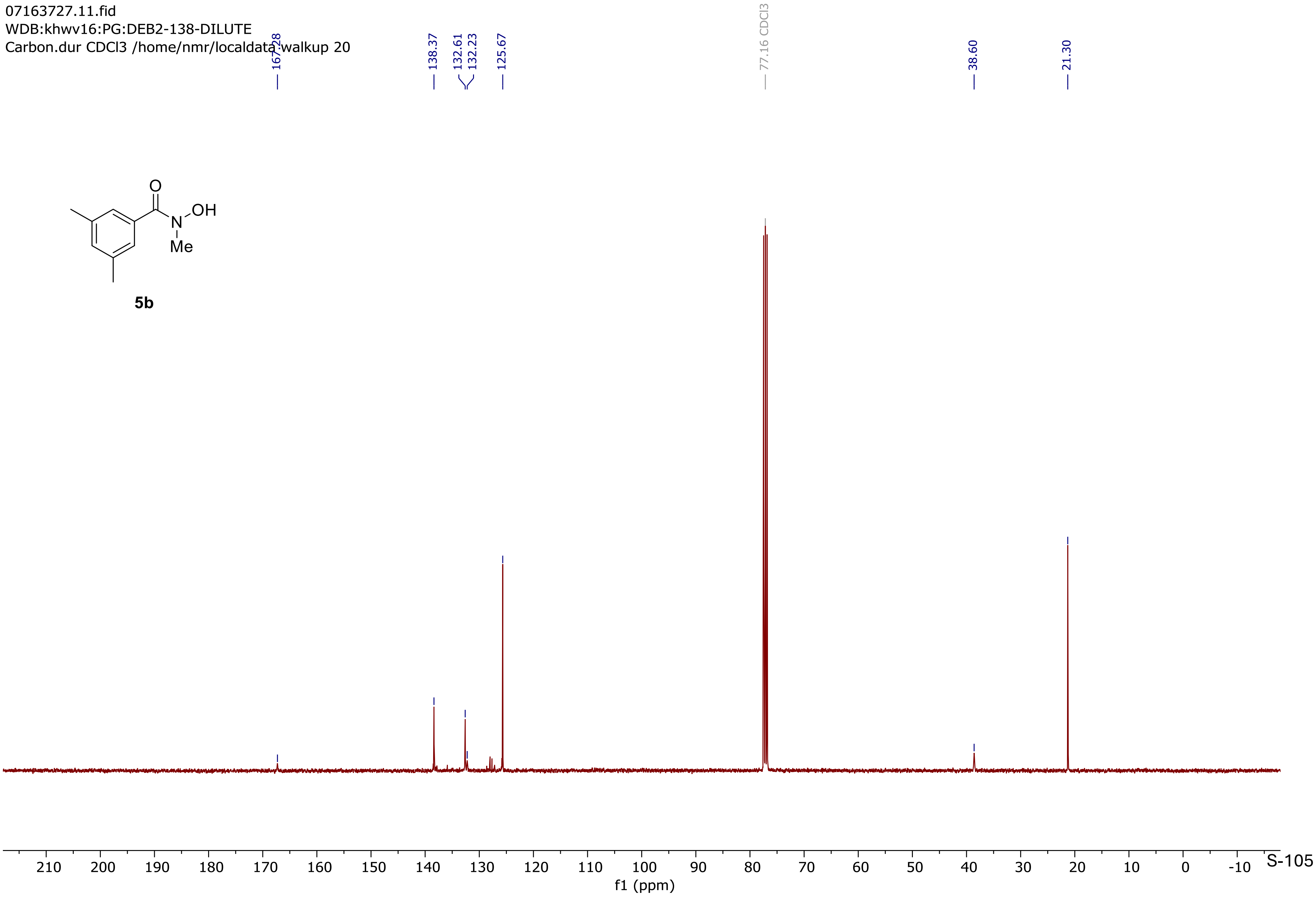

5b

Figure S67; $^{13}C\{^{1}H\}$ NMR (101 MHz, $CDCl_3$) for compound **5b**.

27135311.10.fid
WDB:khwv16:PG:DEB2-126
Proton.dur CDCl3 /home/nmr/localdata walkup 8

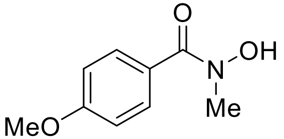

5c

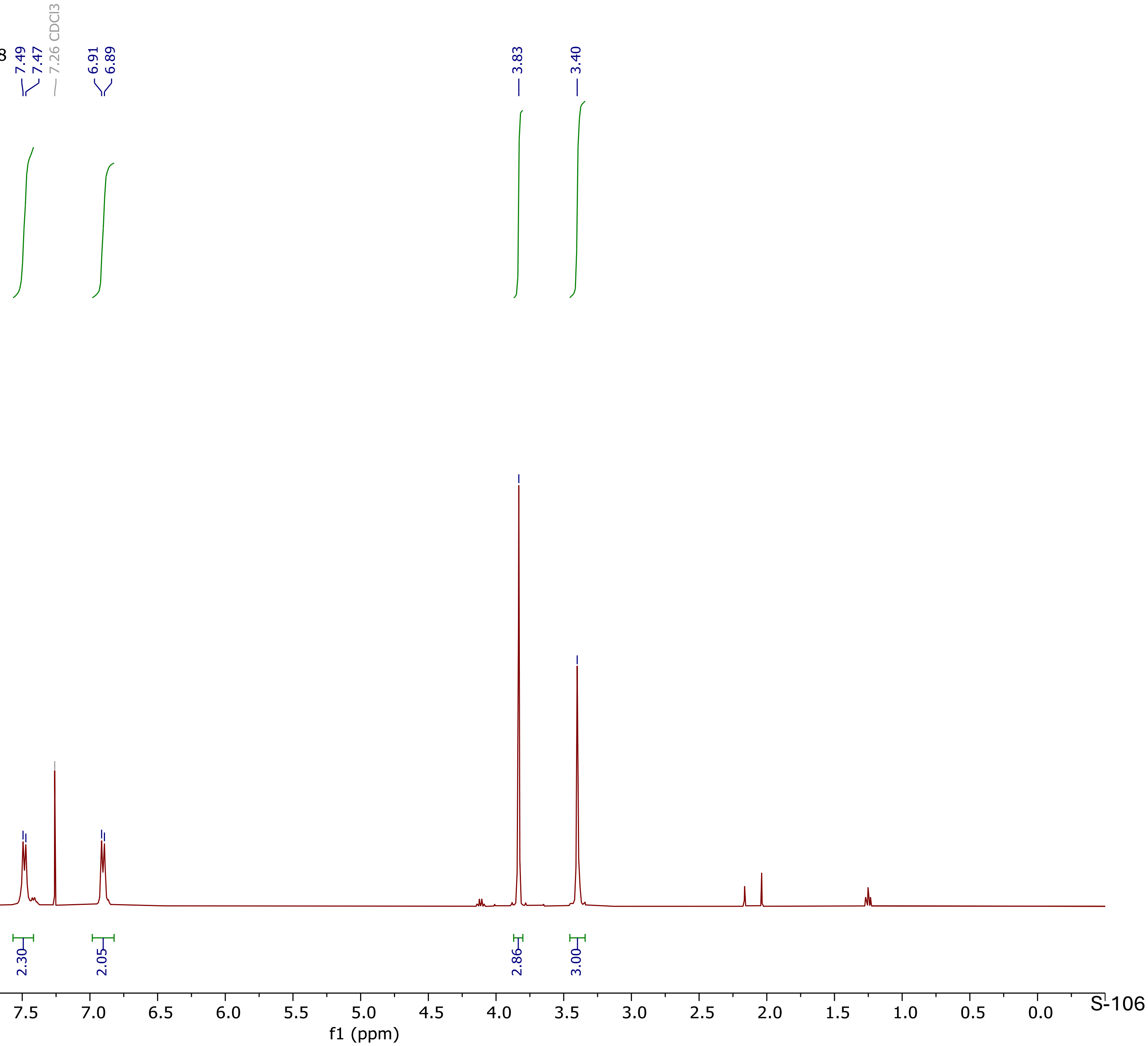

Figure S68; ^{1}H NMR (400 MHz, $CDCl_3$) for compound **5c**.

27135311.14.1.1r
WDB:khwv16:PG:DEB2-126
Carbon.dur CDCl3 /home/nmr/localdata:walkup 8

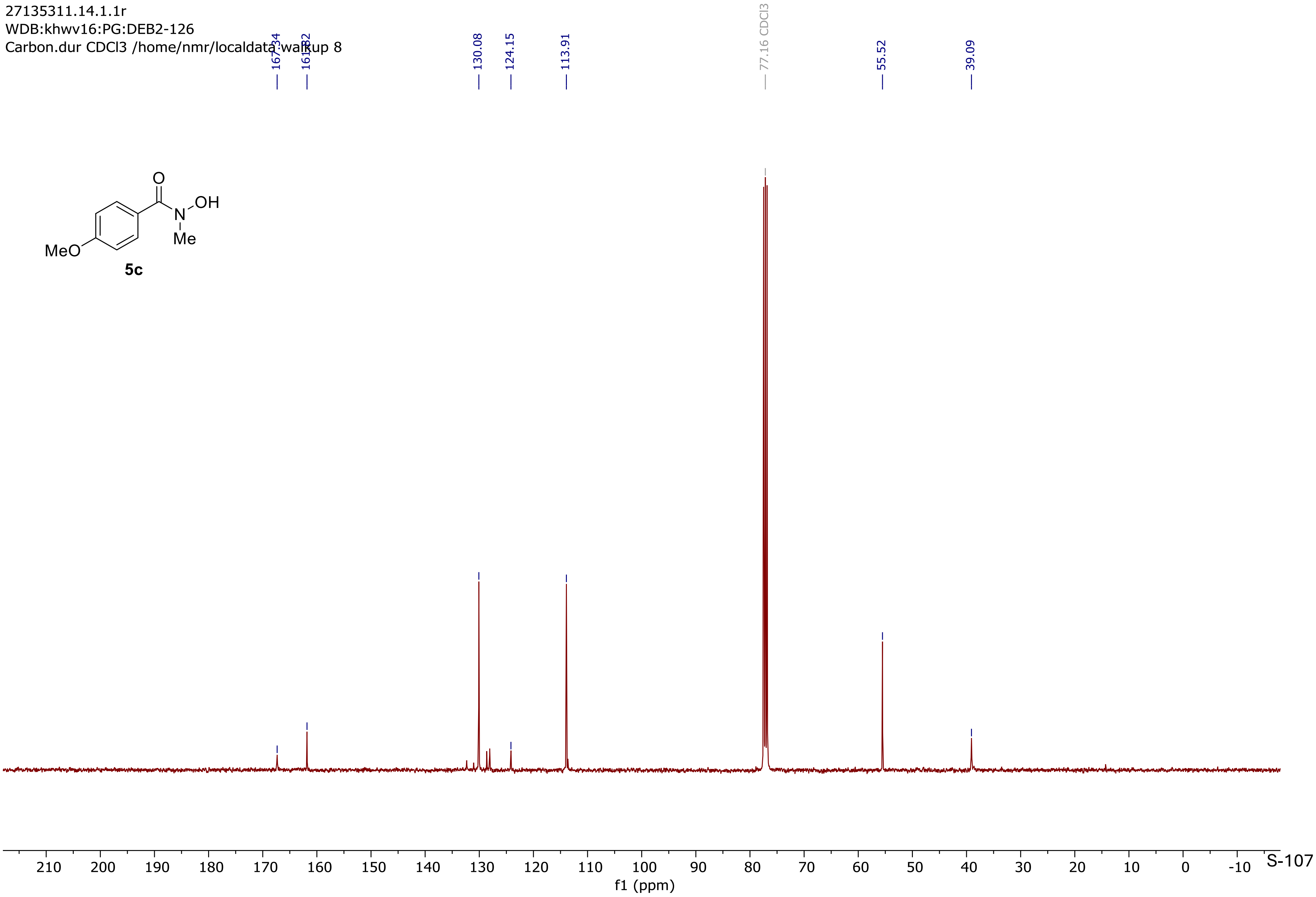

5c

Figure S69; $^{13}C\{^{1}H\}$ NMR (101 MHz, $CDCl_3$) for compound **5c**.

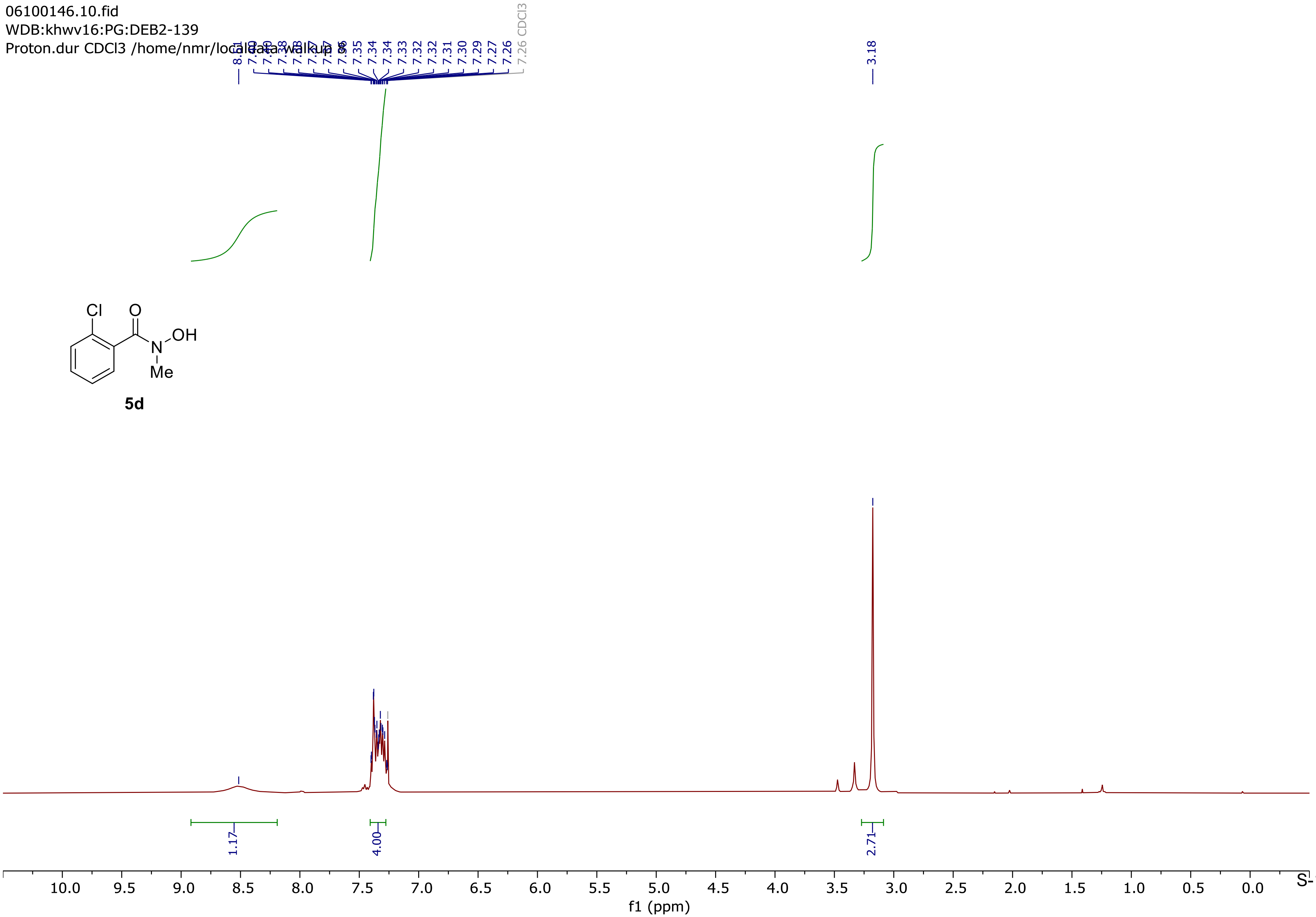

Figure S70; ^{1}H NMR (400 MHz, $CDCl_3$) for compound **5d**.

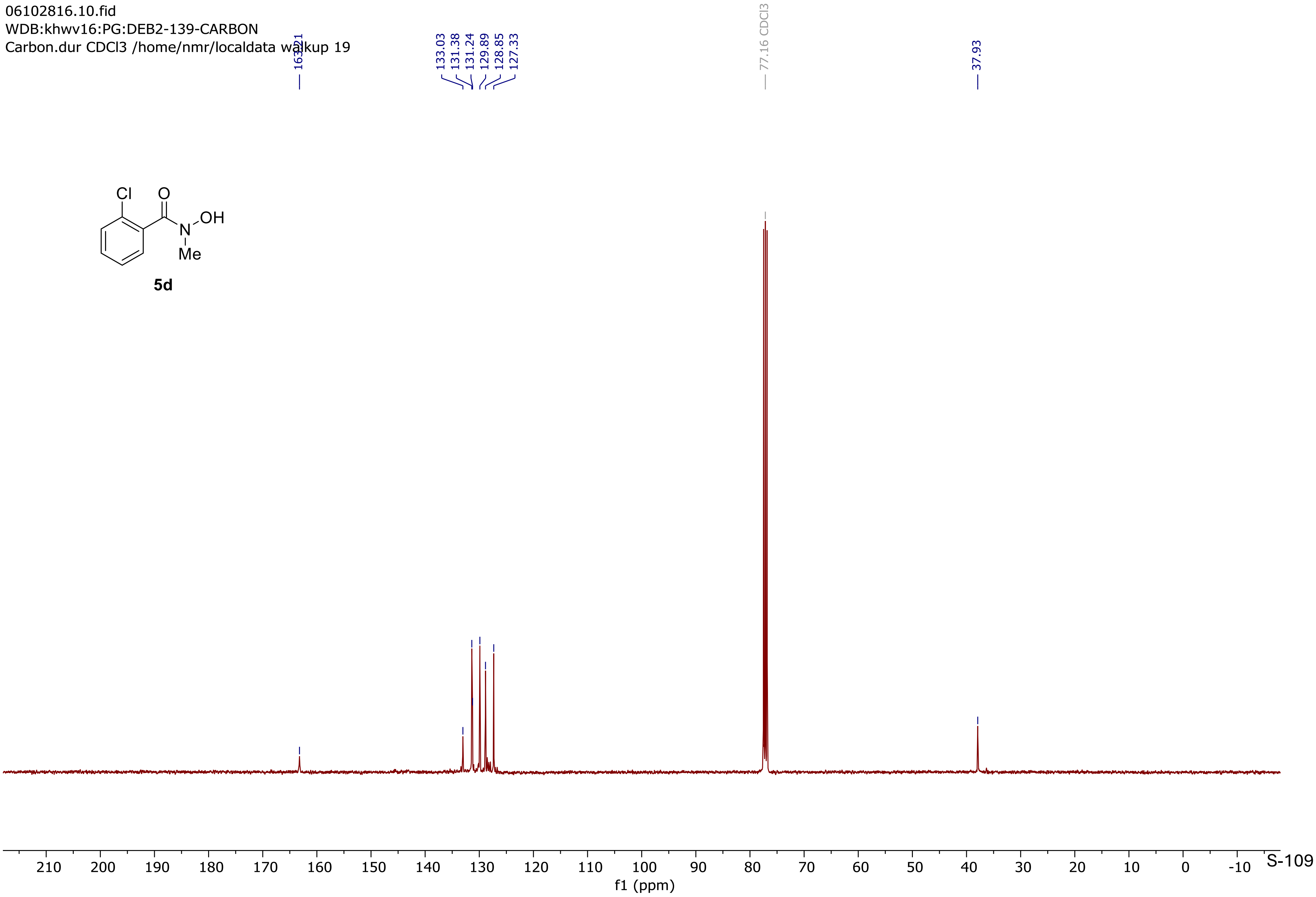

Figure S71; $^{13}C\{^{1}H\}$ NMR (101 MHz, $CDCl_3$) for compound **5d**.

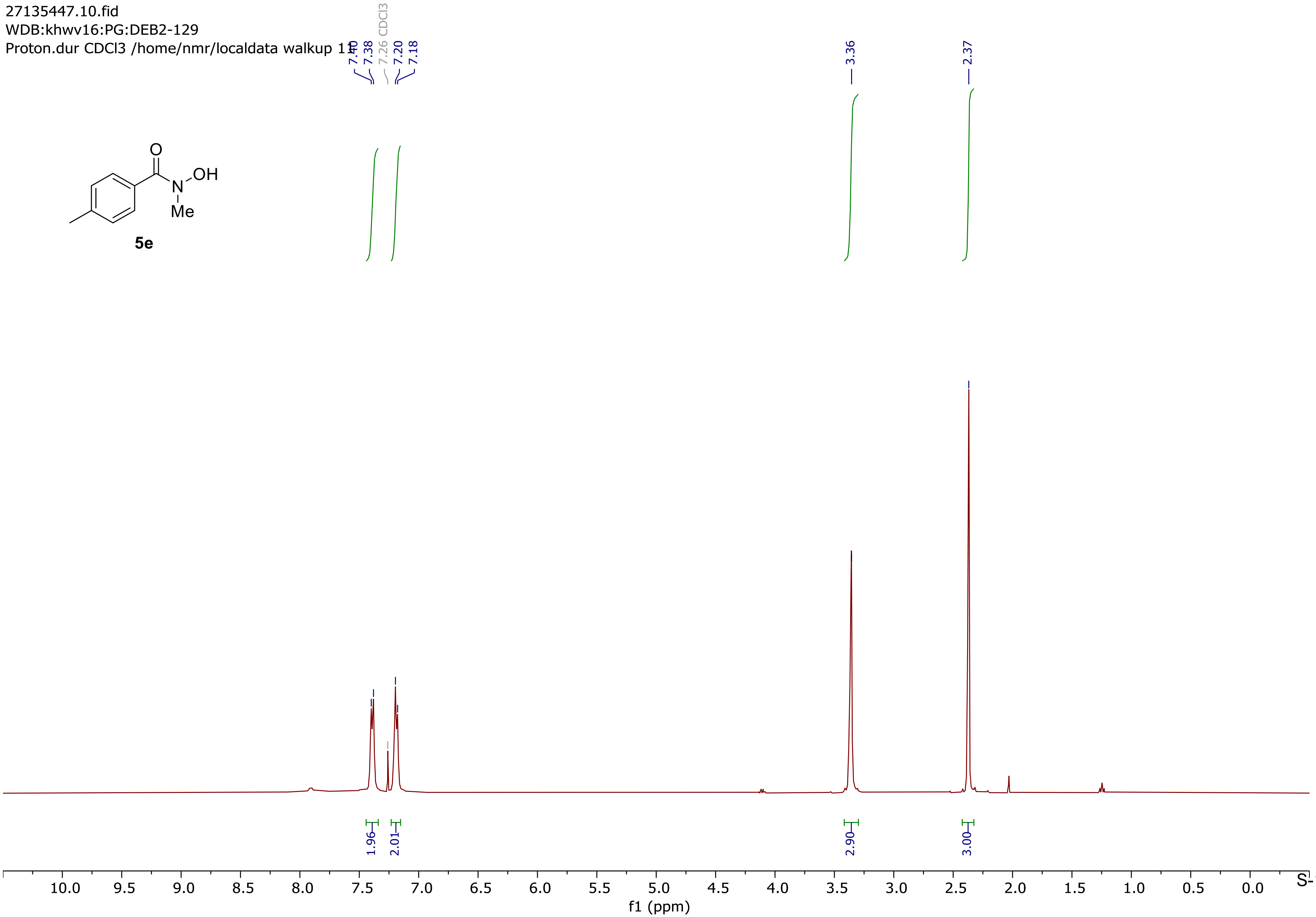

Figure S72; ^{1}H NMR (400 MHz, $CDCl_3$) for compound **5e**.

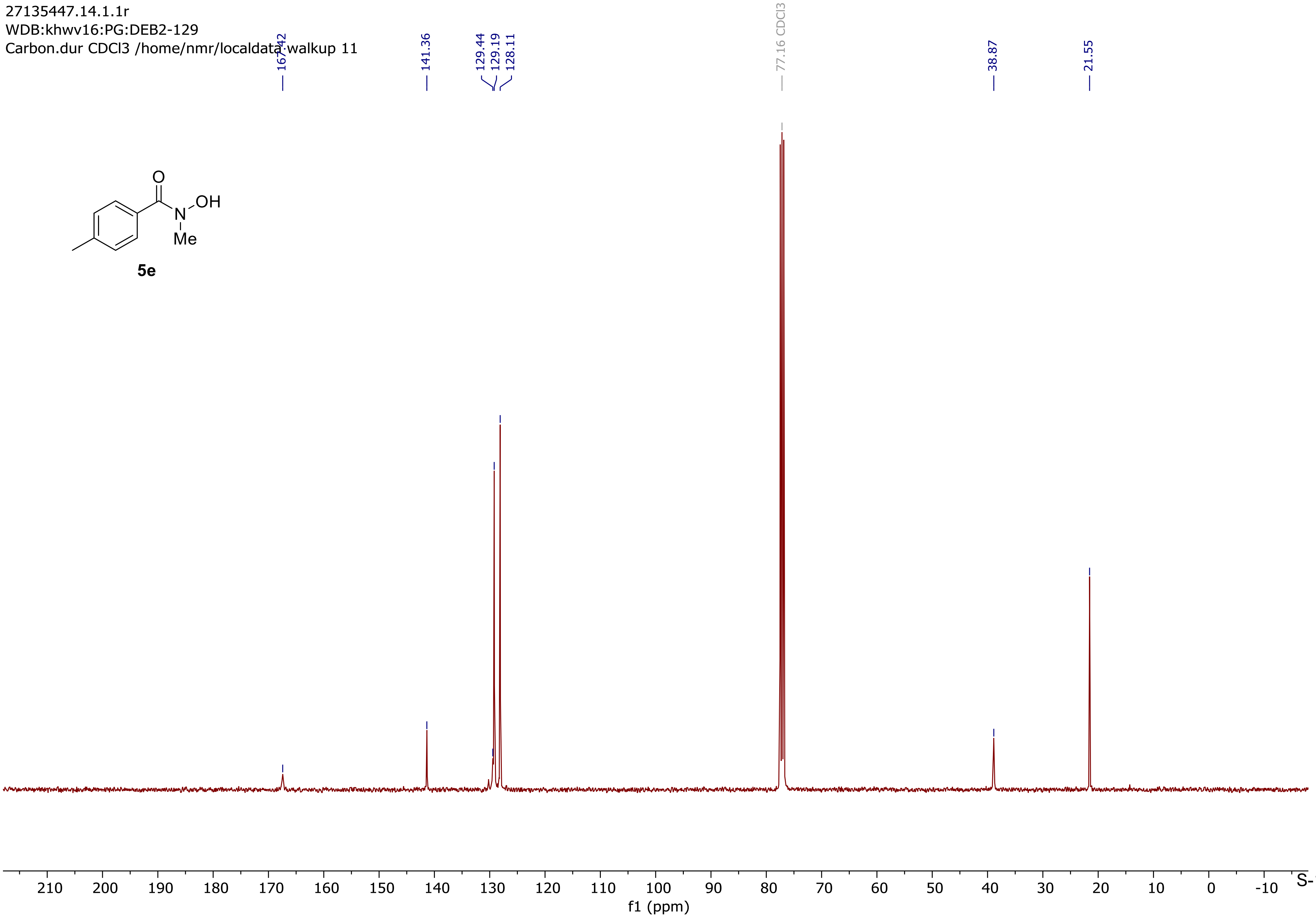

Figure S73; $^{13}C\{^{1}H\}$ NMR (101 MHz, $CDCl_3$) for compound **5e**.

10162523.17.fid
WDB:DEB:PG:DEB2-140
Proton.dur MeOD /home/nmr/localdata walkup 15

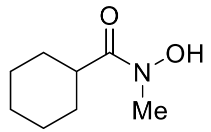

5f

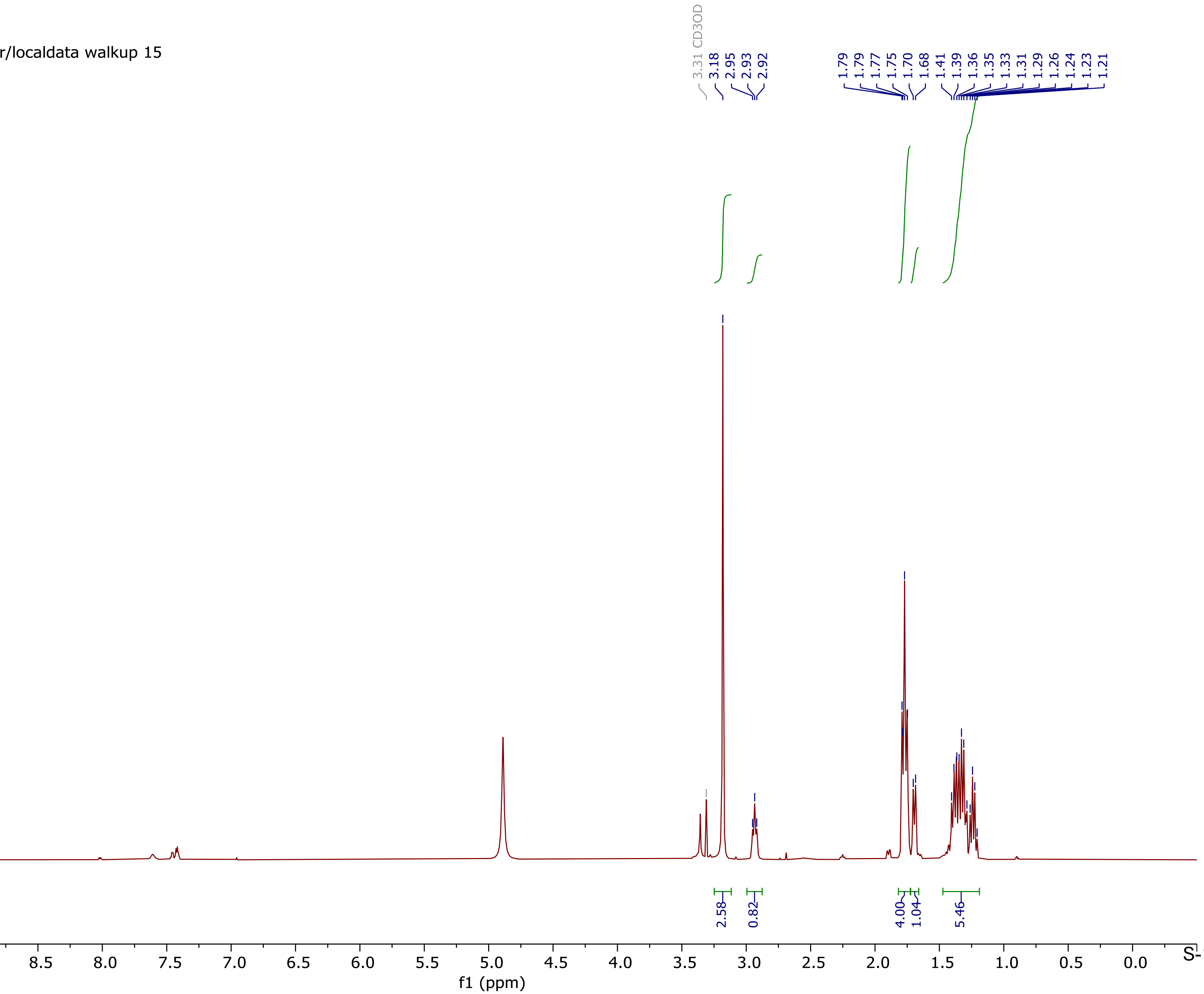

Figure S74; ^{1}H NMR (700 MHz, MeOD) for compound **5f**.

10162523.14.fid
WDB:DEB:PG:DEB2-140
Carbon_50_min.dur MeOD /home/nmr/localdata walkup 15

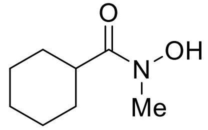

5f

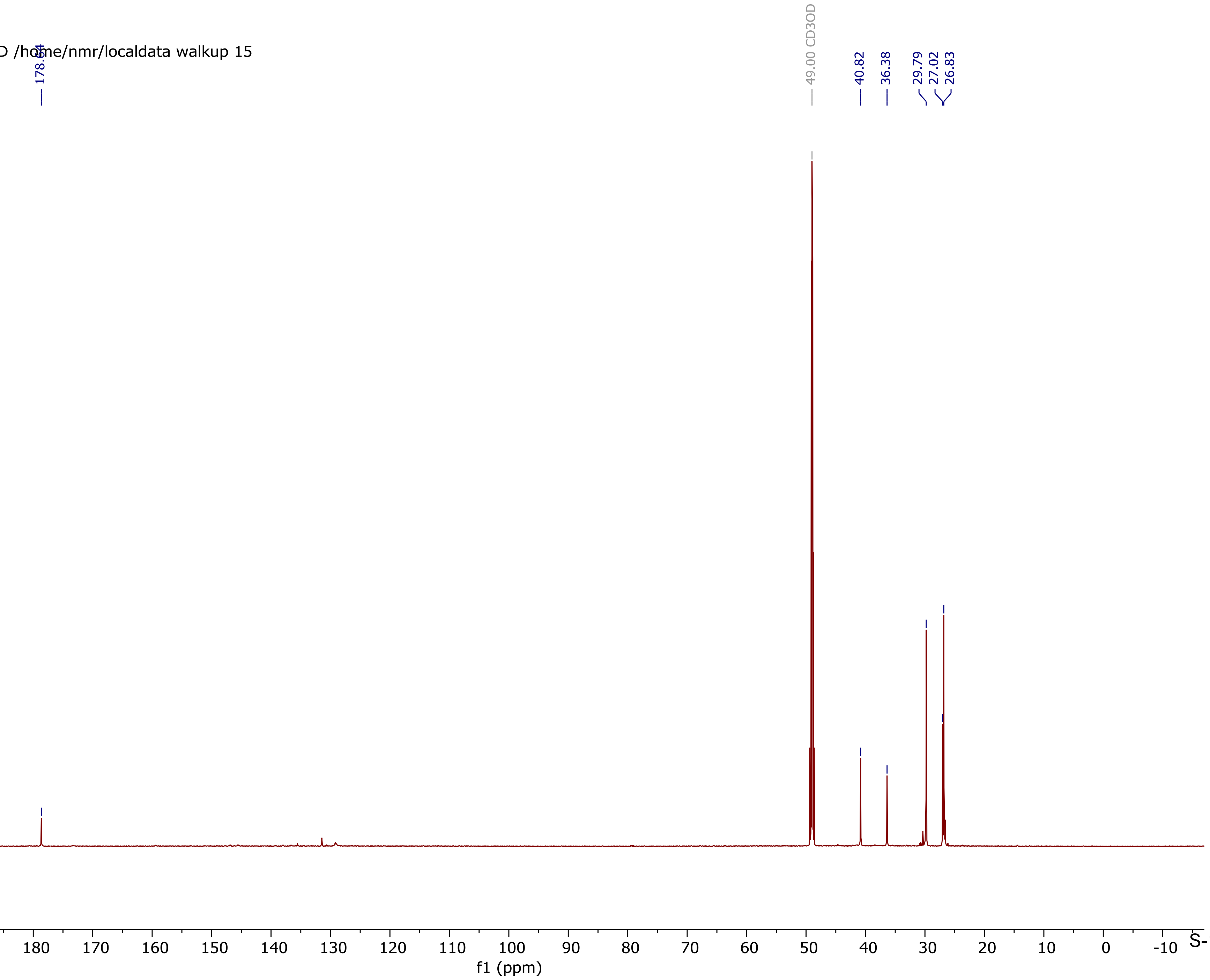

Figure S75; $^{13}C\{^{1}H\}$ NMR (176 MHz, MeOD) for compound **5f**.

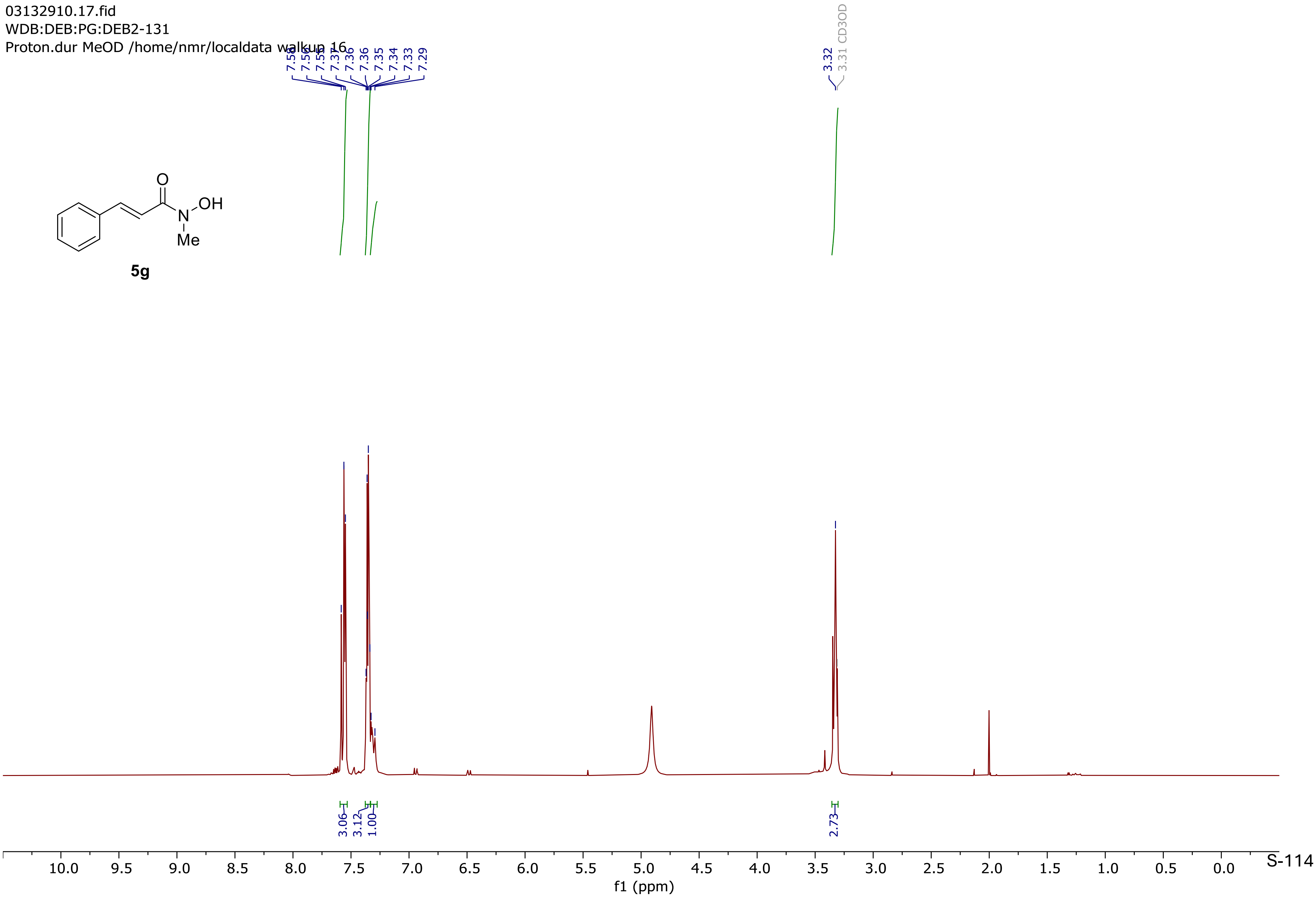

Figure S76; ^{1}H NMR (700 MHz, MeOD) for compound **5g**.

30174936.11.fid
WDB:khwv16:PG:DEB2-131-MEOH
Carbon.dur MeOD /home/nmr/localdata walkup 19

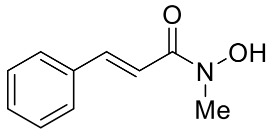

5g

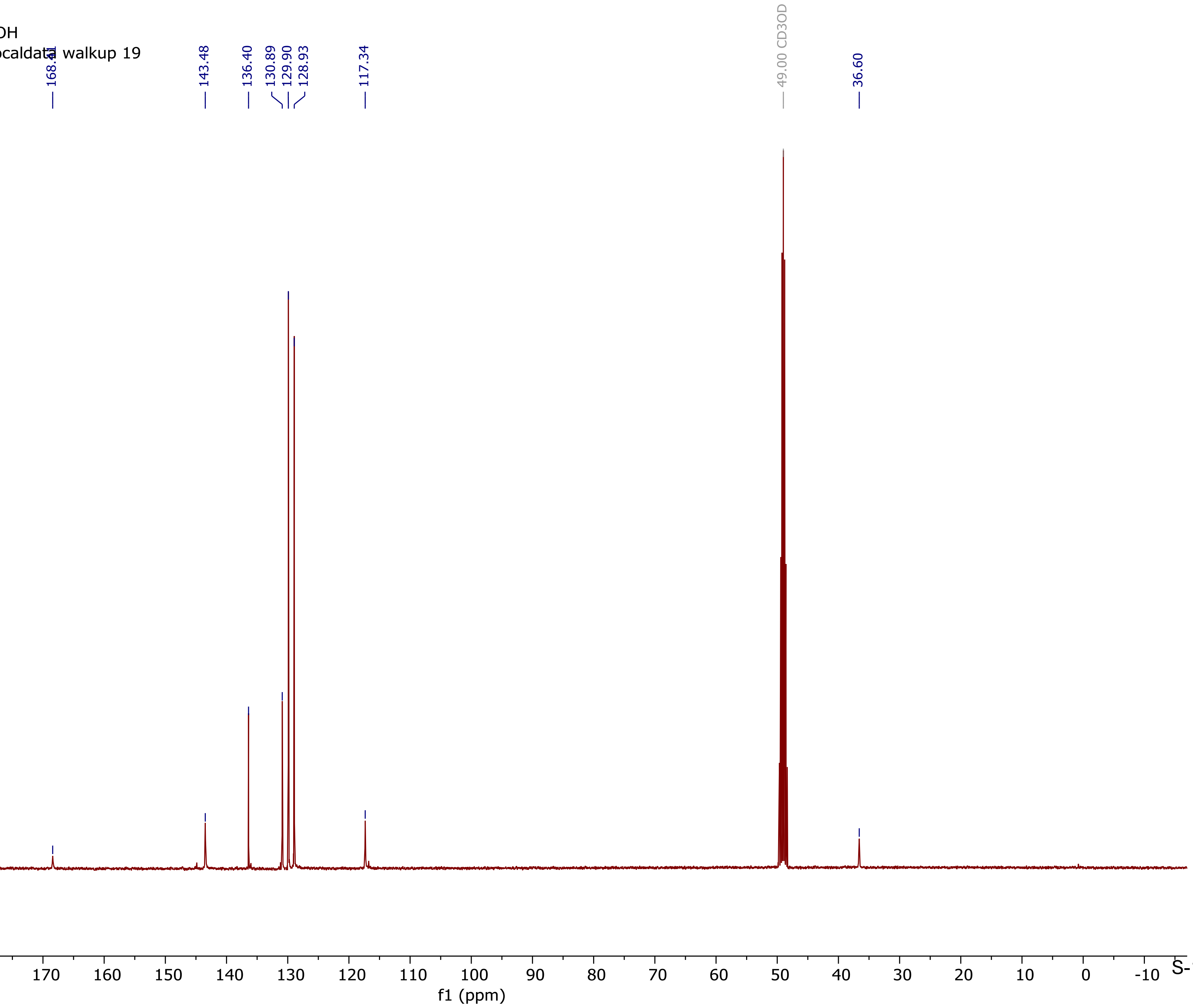

Figure S77; $^{13}C\{^{1}H\}$ NMR (101 MHz, MeOD) for compound **5g**.

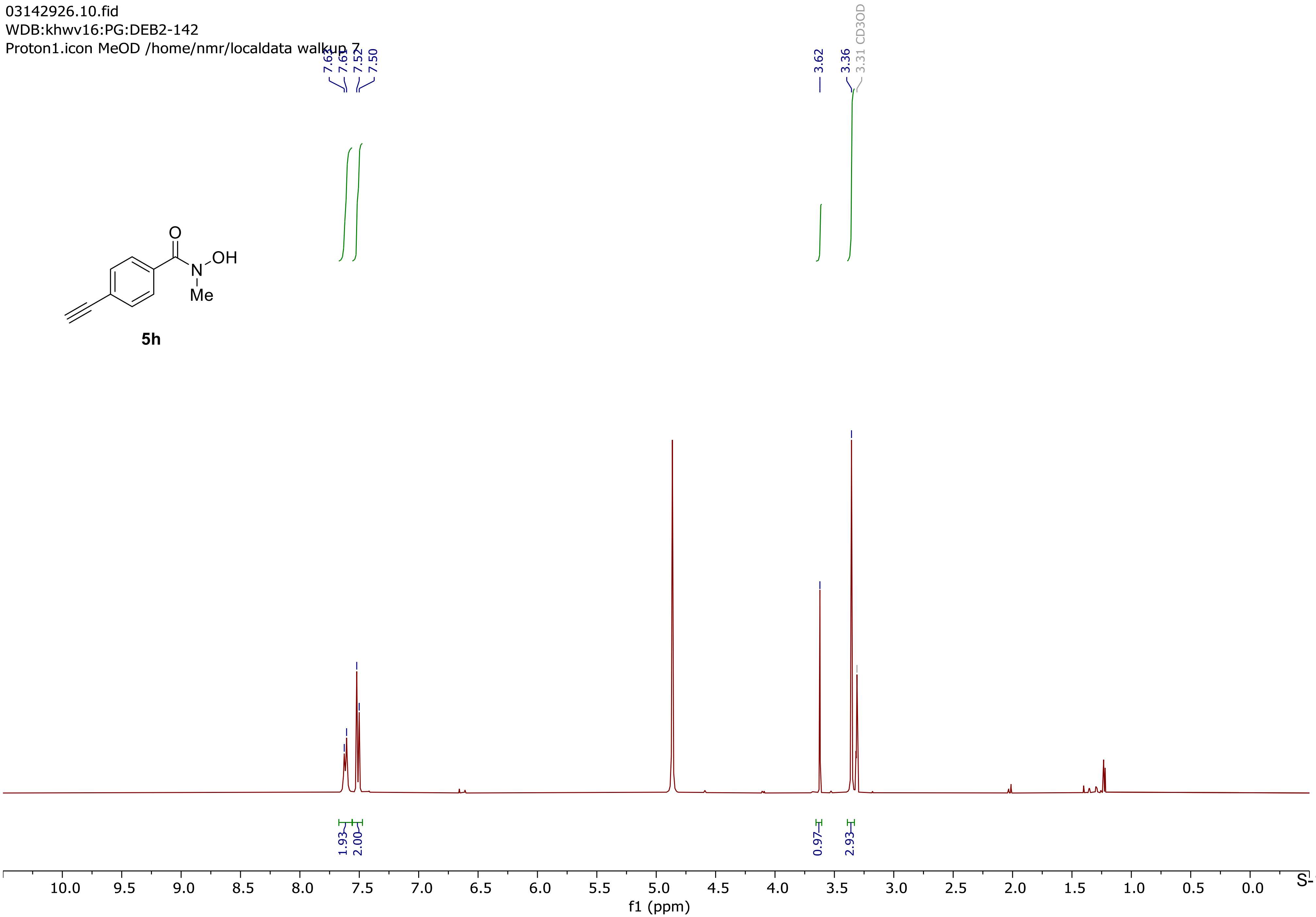

5h

Figure S78; ^{1}H NMR (400 MHz, MeOD) for compound **5h**.

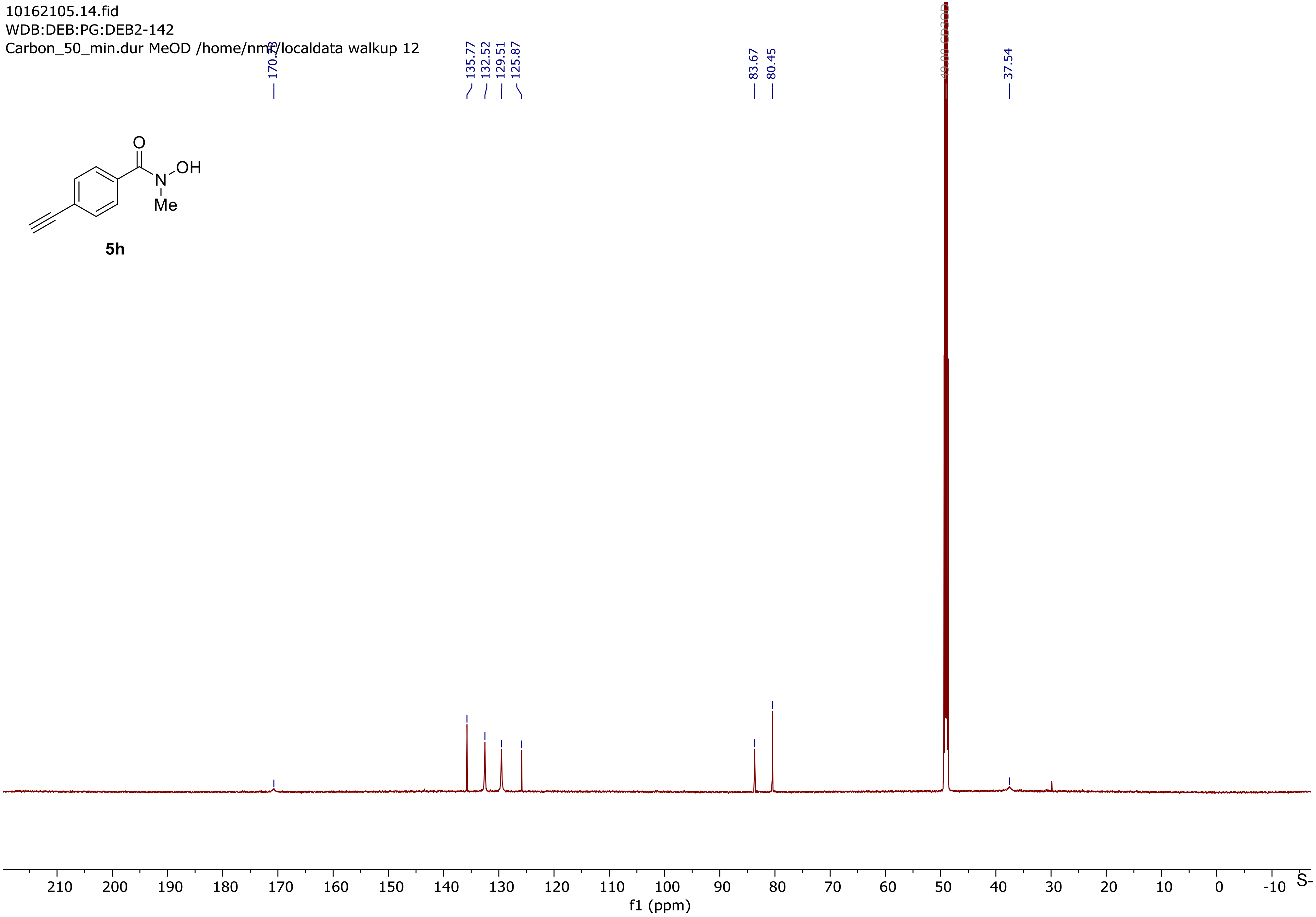

Figure S79; $^{13}C\{^{1}H\}$ NMR (176 MHz, MeOD) for compound **5h**.

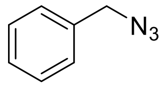

6

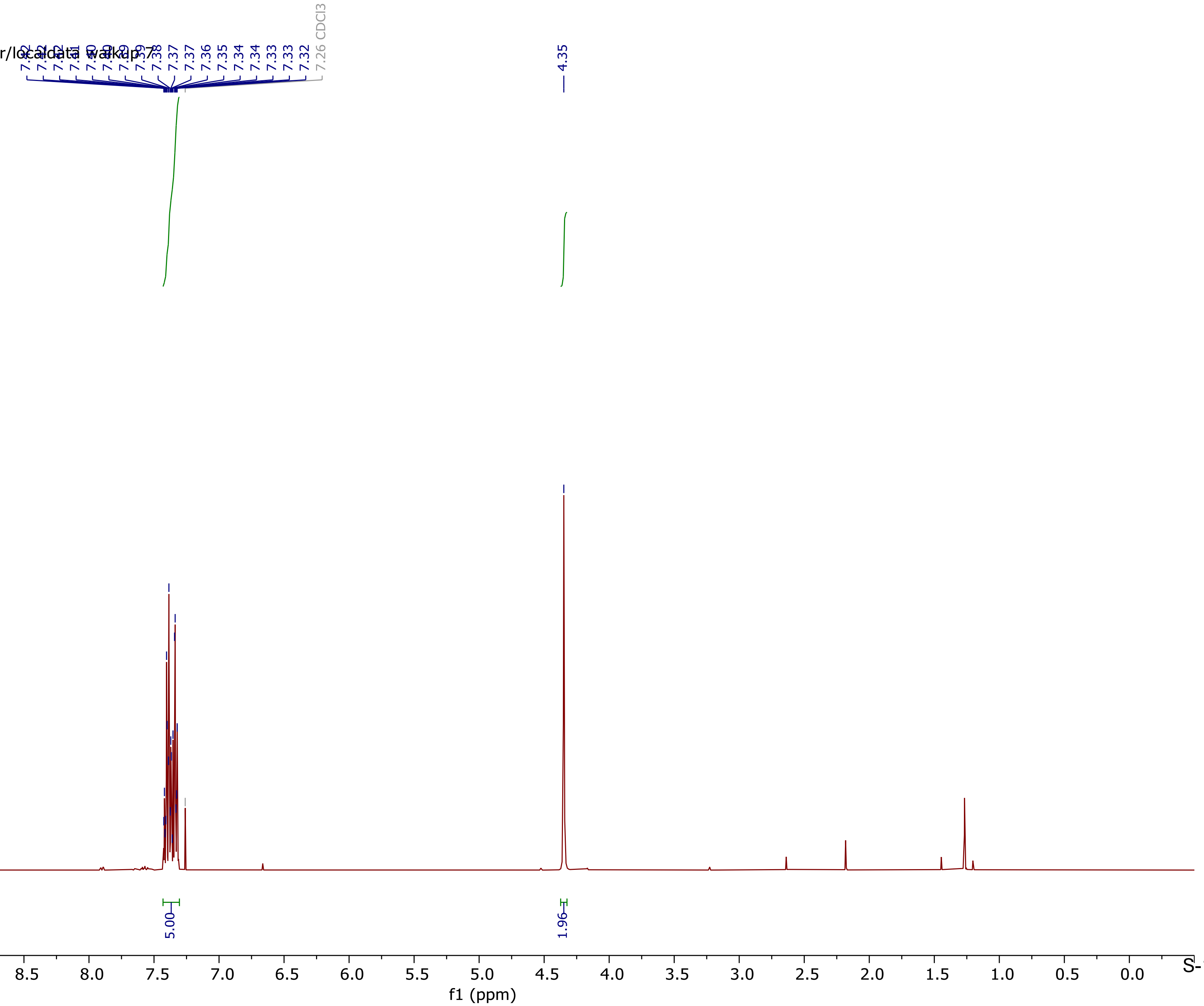

Figure S80; ^{1}H NMR (400 MHz, $CDCl_3$) for compound **6**.

10132841.11.fid
WDB:khwv16:PG:DEB2-115
Carbon.dur CDCl3 /home/nmr/localdata walkup 7

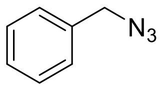

6

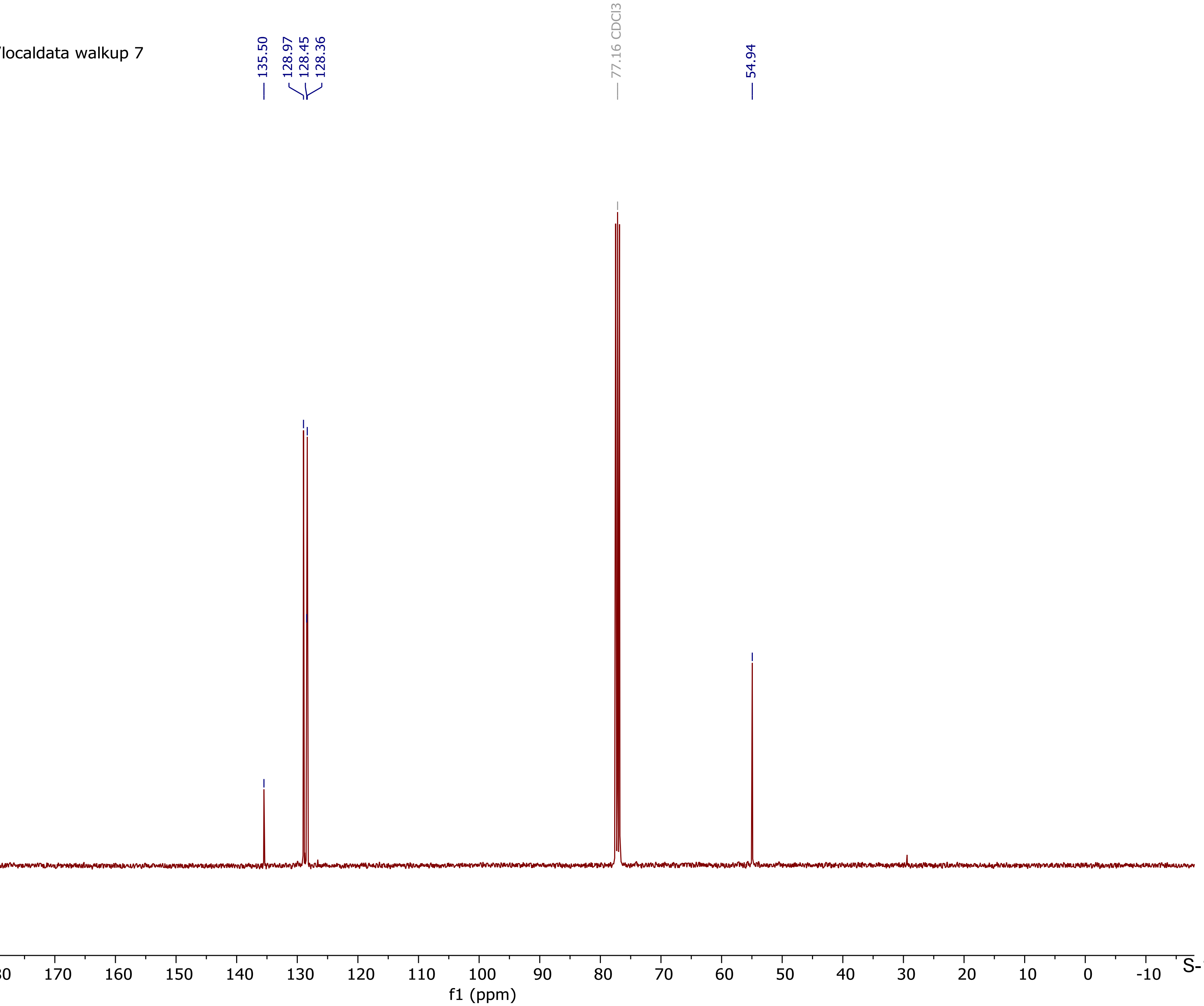

Figure S81; $^{13}C\{^{1}H\}$ NMR (101 MHz, $CDCl_3$) for compound **6**.

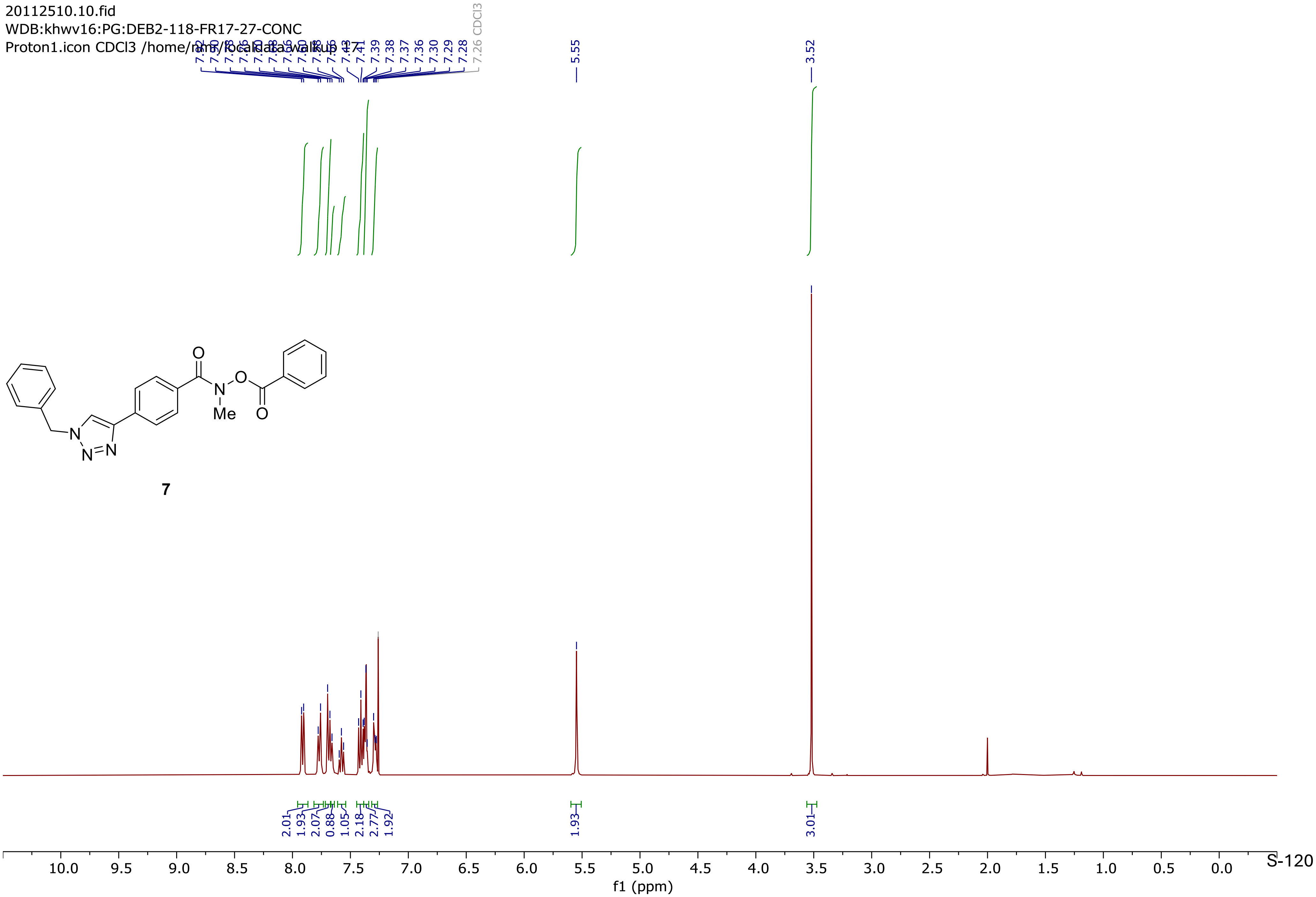

Figure S82; ^{1}H NMR (400 MHz, $CDCl_3$) for compound **7**.

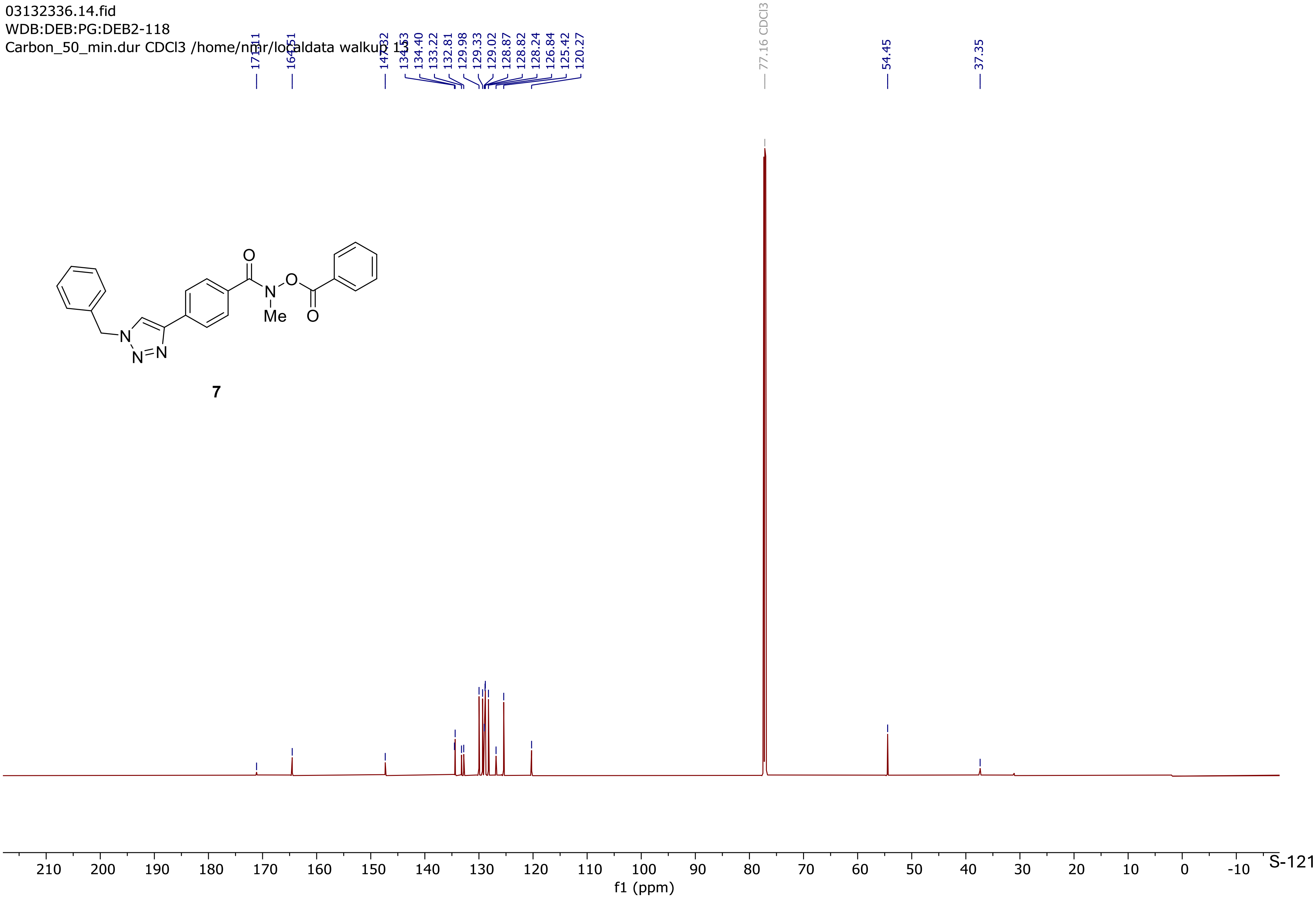

Figure S83; $^{13}C\{^{1}H\}$ NMR (176 MHz, $CDCl_3$) for compound **7**.

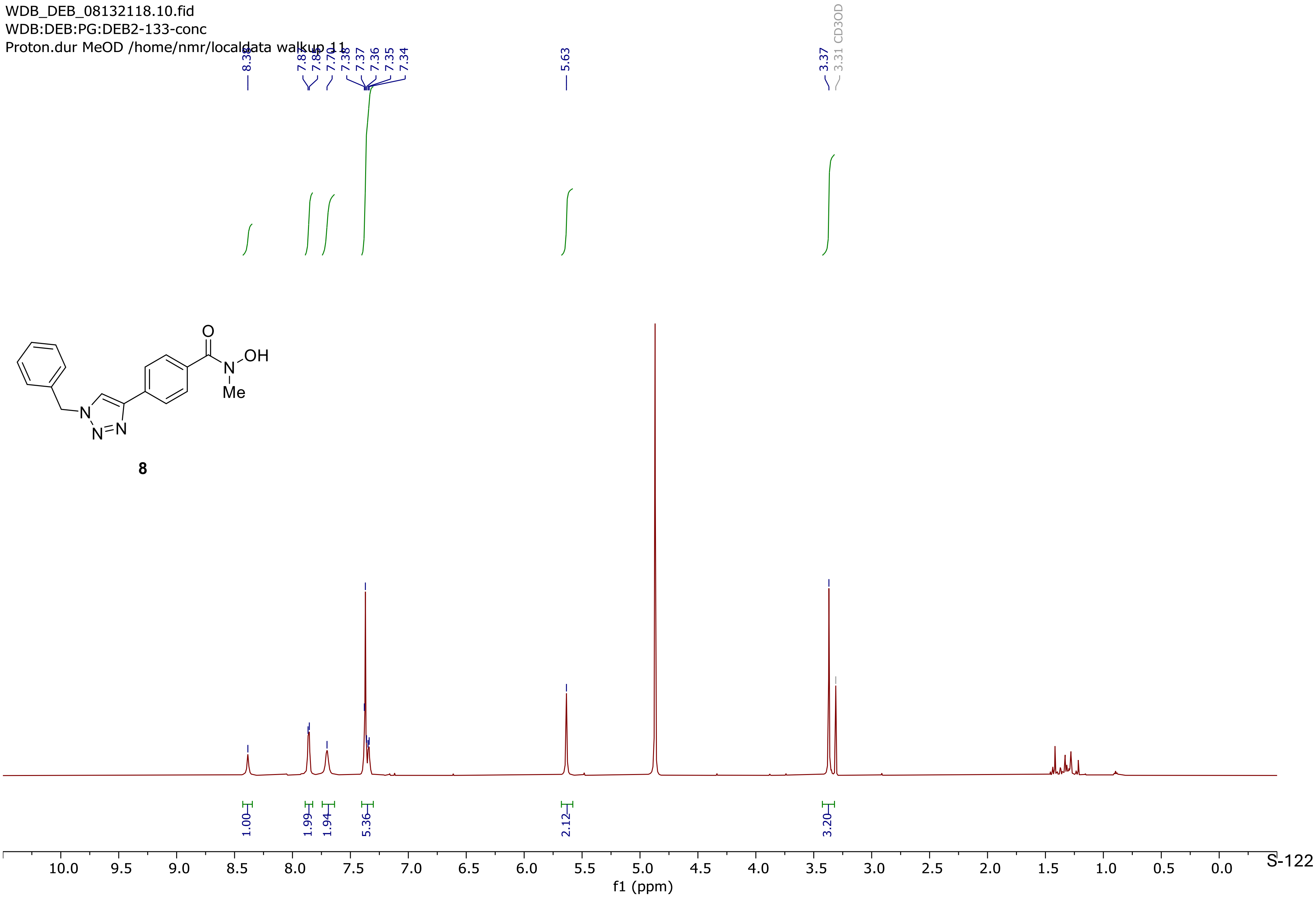

8

Figure S84; ^{1}H NMR (700 MHz, MeOD) for compound **8**.

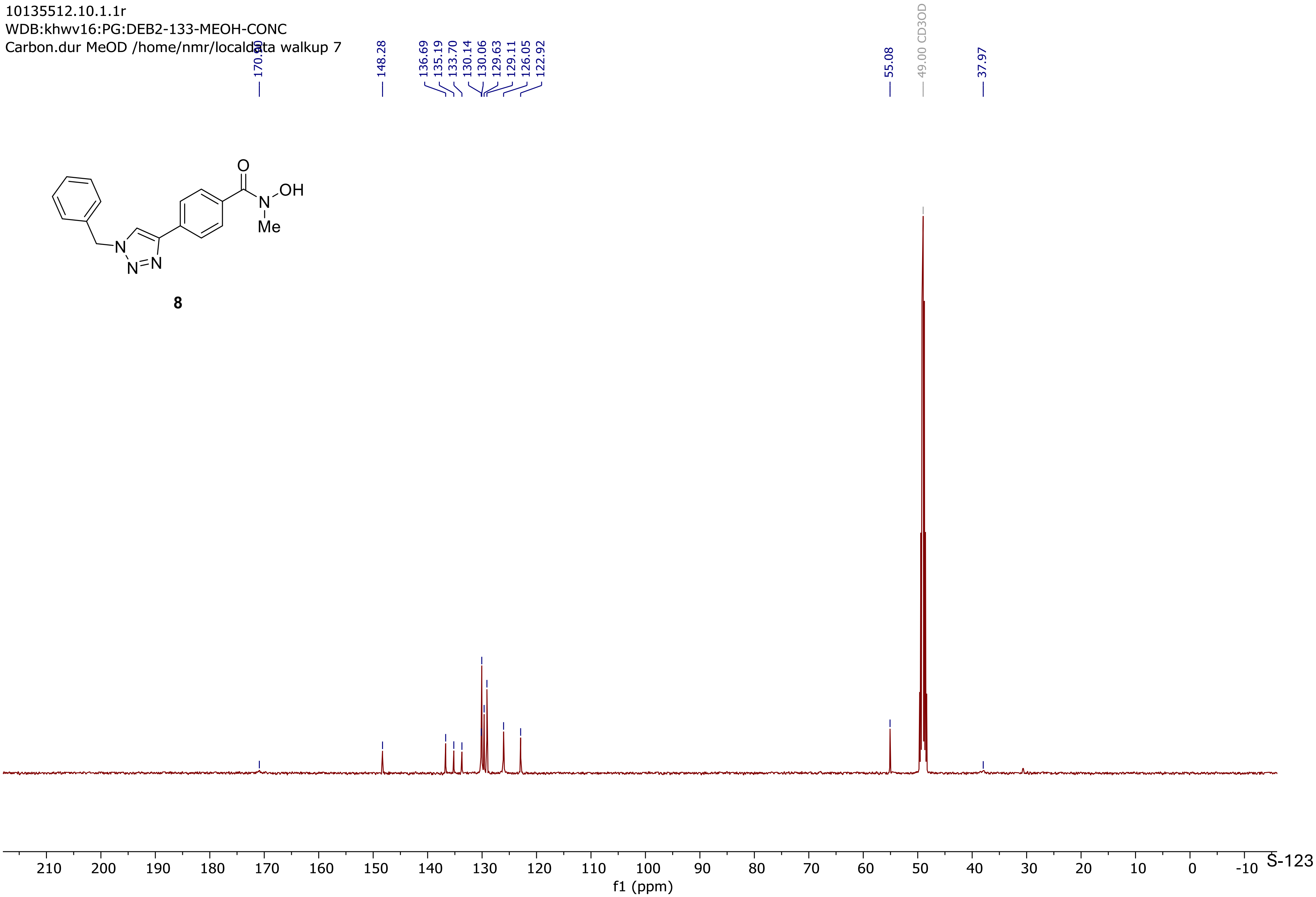

Figure S85; $^{13}C\{^{1}H\}$ NMR (101 MHz, MeOD) for compound **8**.

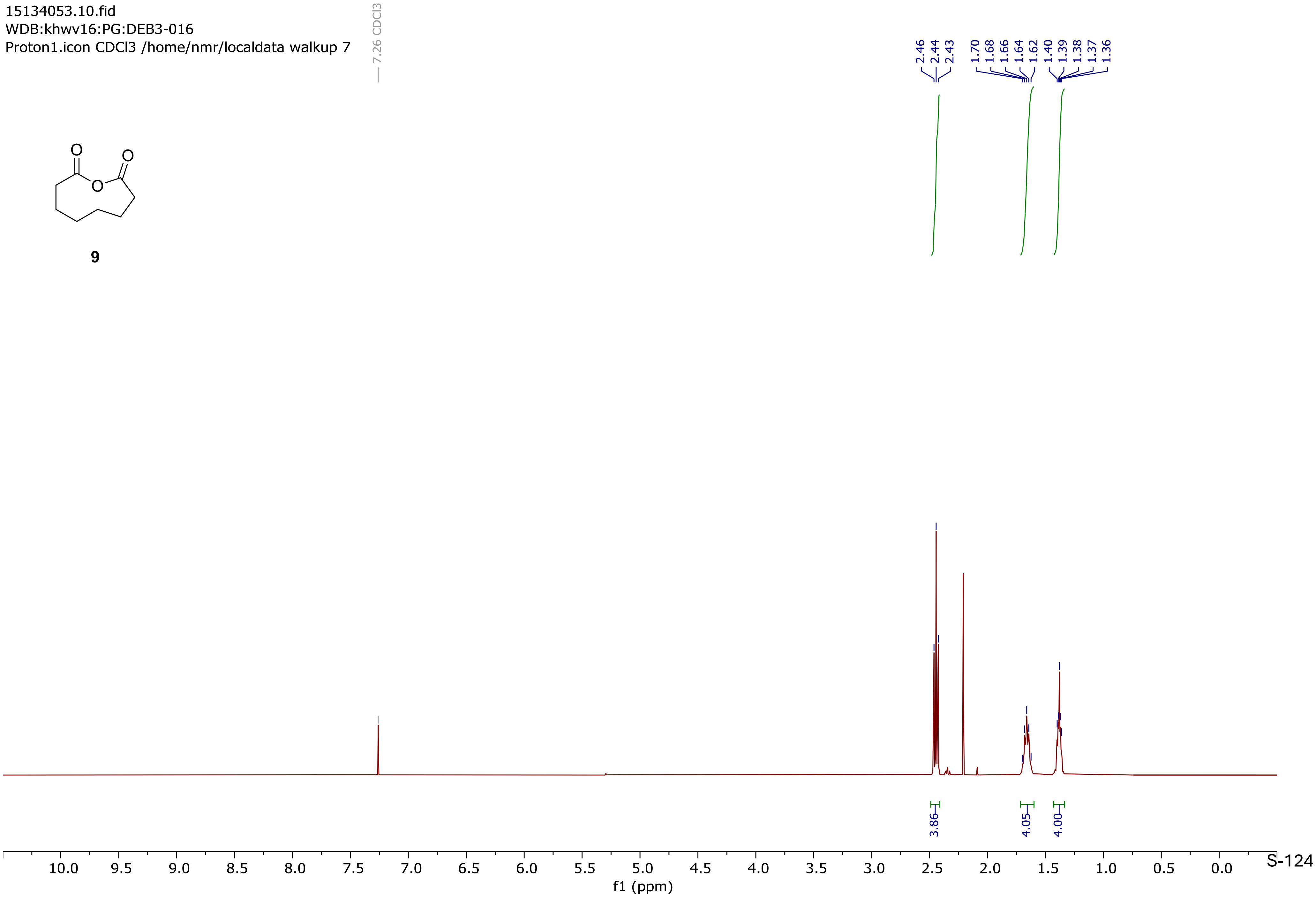

Figure S86; ^{1}H NMR (400 MHz, $CDCl_3$) for compound **9**.

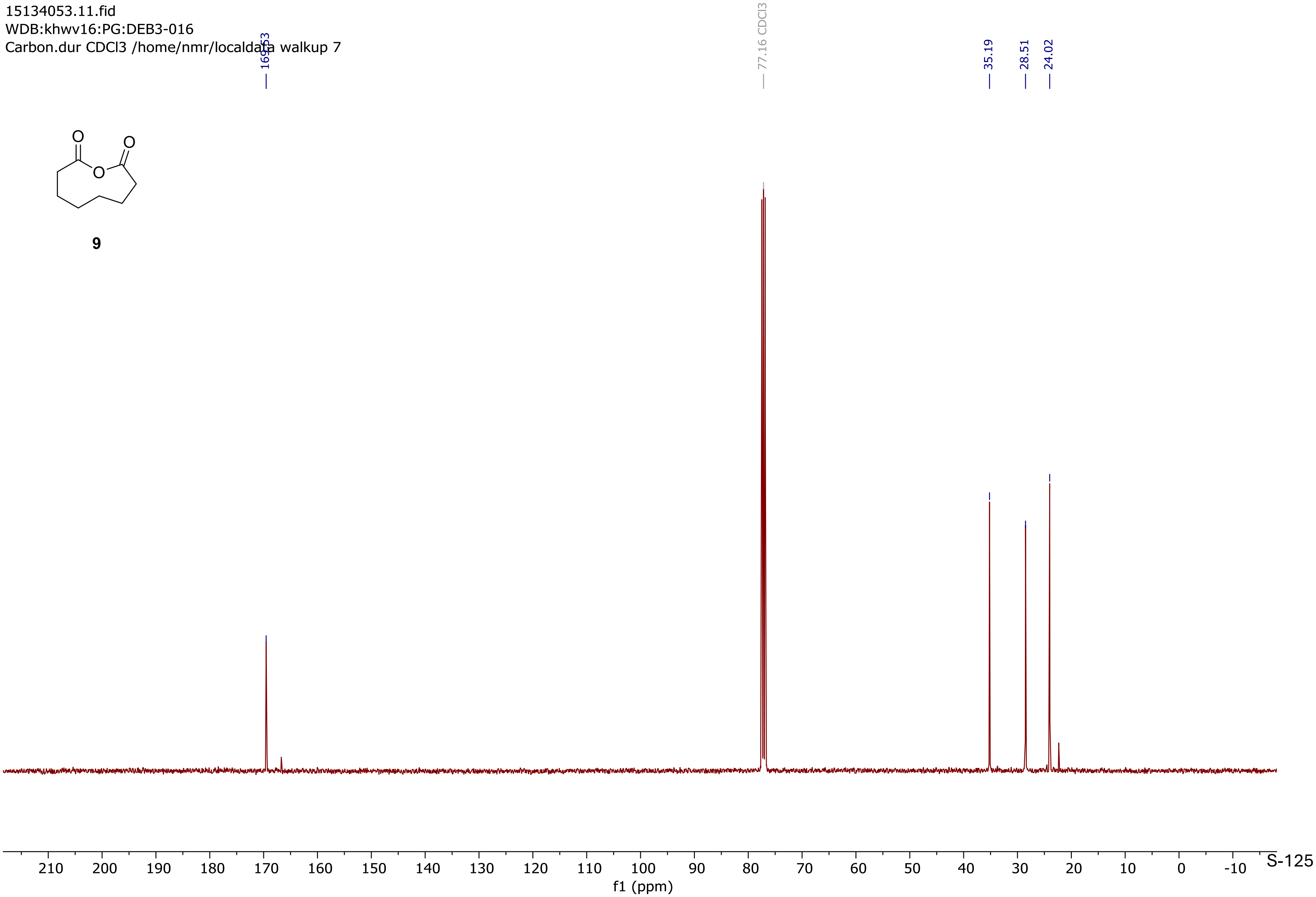

Figure S87; $^{13}C\{^{1}H\}$ NMR (101 MHz, $CDCl_3$) for compound **9**.

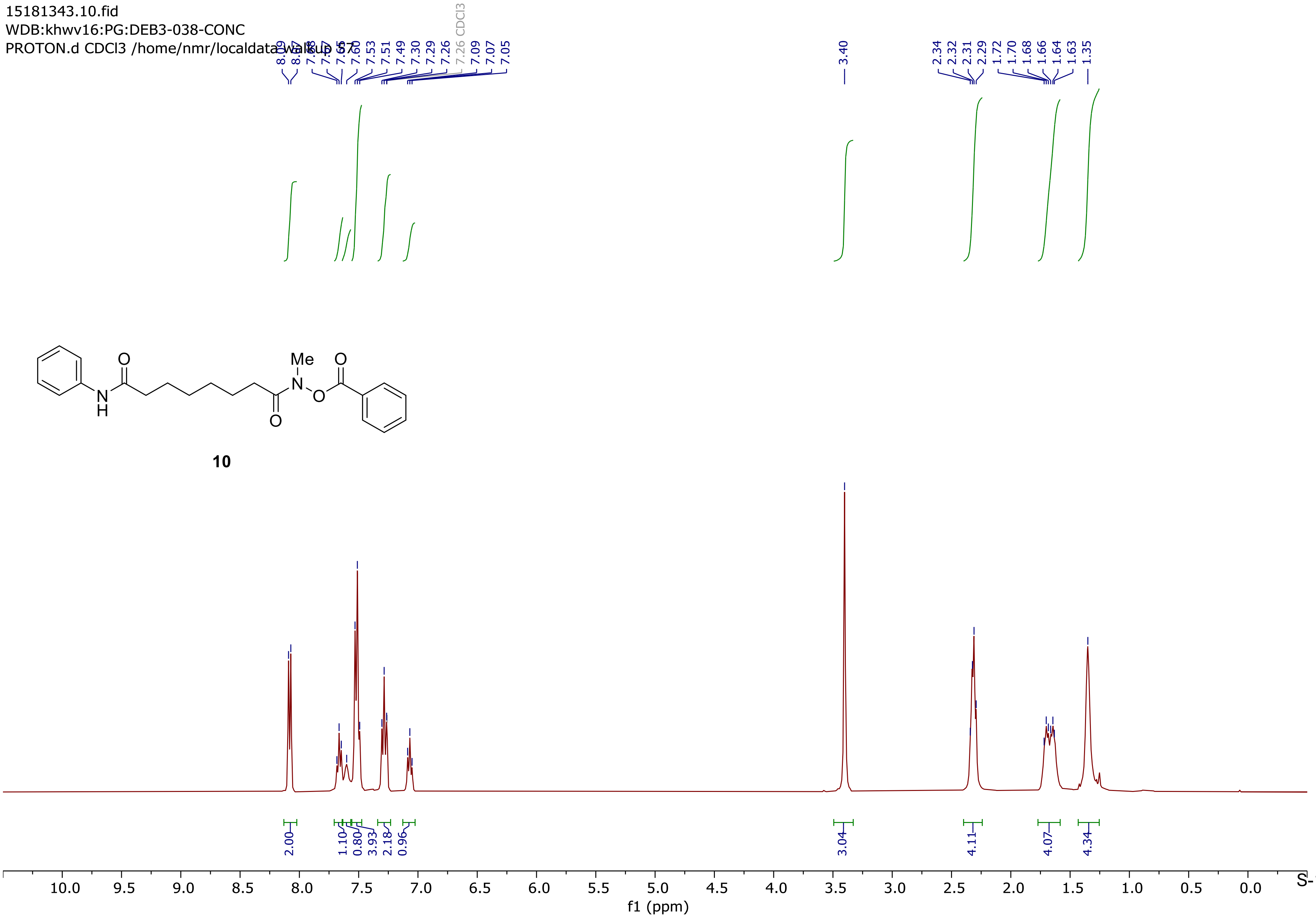

Figure S88; ^{1}H NMR (400 MHz, $CDCl_3$) for compound **10**.

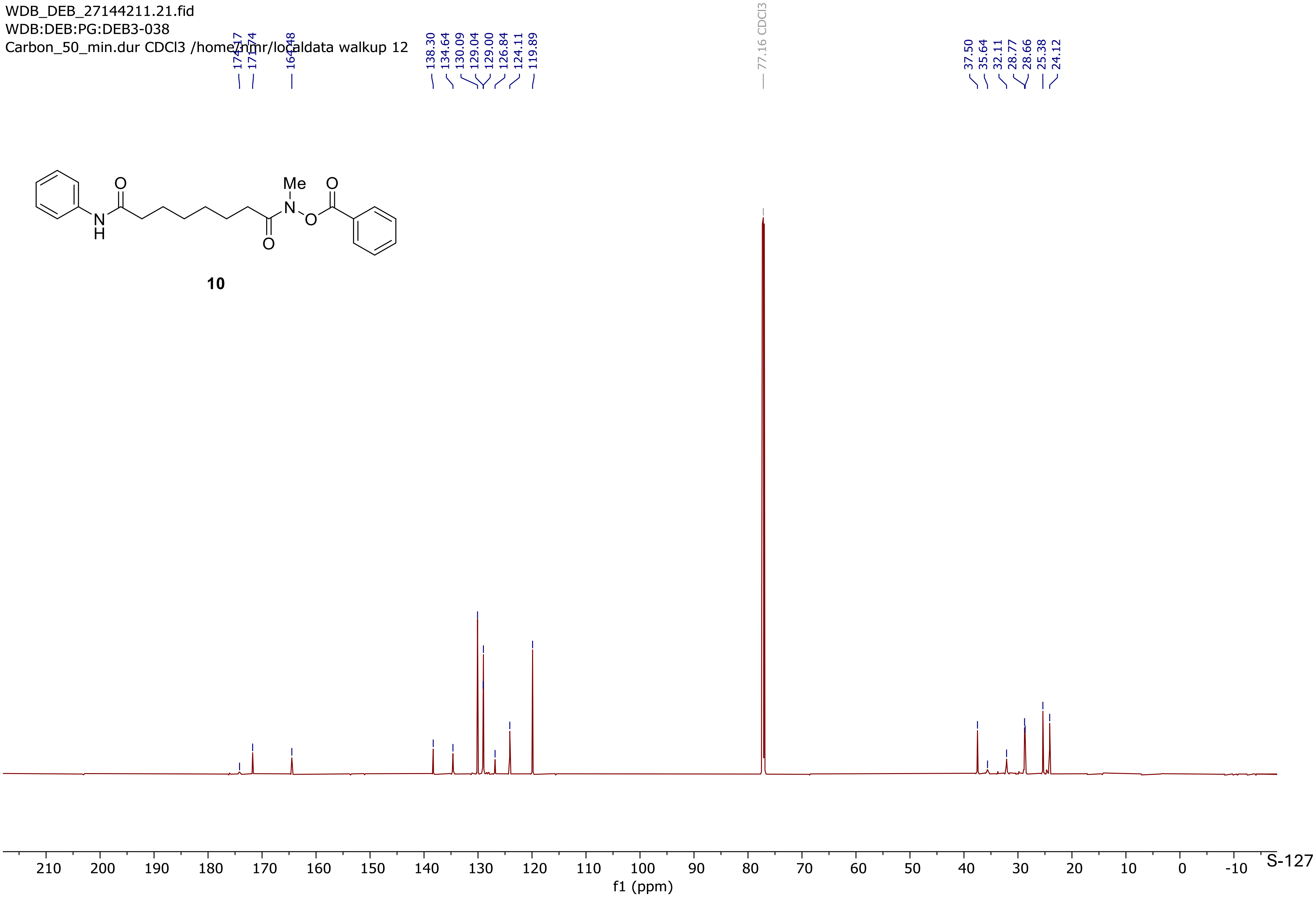

Figure S89; $^{13}C\{^{1}H\}$ NMR (176 MHz, $CDCl_3$) for compound **10**.

X-ray crystallography

Table S1; SCXRD data and structure refinement.

**Compound code**	**3a**	**4h**	**4j**	**4r**	**5h**
**Empirical formula**	$C_8H_{10}ClNO_2$	$C_{16}H_{15}NO_4$	$C_{17}H_{13}NO_3$	$C_{18}H_{16}N_2O_3$	$C_{10}H_9NO_2$
**Formula weight**	187.627	285.302	279.298	308.339	175.188
**Temperature/K**	120.0(2)	120.00	120.0(2)	120.0(2)	120.0(2)
**Crystal system**	monoclinic	orthorhombic	orthorhombic	monoclinic	monoclinic
**Space group**	$P2_1/n$	$P2_12_12_1$	$P2_12_12_1$	$P2_1$	Cc
**a/Å**	7.4778(2)	5.6865(1)	5.7762(1)	7.8128(3)	6.3064(3)
**b/Å**	6.5114(2)	7.4961(2)	7.1549(2)	11.6621(5)	16.7806(7)
**c/Å**	17.8798(4)	33.3761(8)	33.8182(9)	9.4364(4)	8.5242(4)
**α/°**	90	90	90	90	90
**β/°**	90.299(1)	90	90	104.821(2)	98.627(2)
**γ/°**	90	90	90	90	90
**Volume/Å^3**	870.57(4)	1422.71(6)	1397.64(6)	831.18(6)	891.87(7)
**Z**	4	4	4	2	4
**ρ_{calc}g/cm^3**	1.432	1.332	1.327	1.232	1.305
**μ/mm^{-1}**	0.395	0.800	0.752	0.085	0.092
**F(000)**	392.8	602.2	586.1	324.2	368.3
**Crystal size/mm^3**	0.207 × 0.101 × 0.069	0.292 × 0.281 × 0.186	0.148 × 0.097 × 0.035	0.442 × 0.111 × 0.108	0.188 × 0.081 × 0.041
**Radiation**	Mo Kα (λ = 0.71073 Å)	Cu Kα (λ = 1.54178 Å)	Cu Kα (λ = 1.54178 Å)	Mo Kα (λ = 0.71073 Å)	Mo Kα (λ = 0.71073 Å)
**2Θ range for data collection/°**	4.56 to 69.98	5.3 to 139.92	5.22 to 149.38	4.46 to 52.78	4.86 to 70.5
**Index ranges**	-12 ≤ h ≤ 12, -10 ≤ k ≤ 10, -28 ≤ l ≤ 28	-6 ≤ h ≤ 6, -8 ≤ k ≤ 9, -40 ≤ l ≤ 40	-7 ≤ h ≤ 7, -8 ≤ k ≤ 8, -41 ≤ l ≤ 37	-9 ≤ h ≤ 9, -14 ≤ k ≤ 14, -11 ≤ l ≤ 11	-10 ≤ h ≤ 10, -27 ≤ k ≤ 27, -14 ≤ l ≤ 14
**Reflections collected**	39120	24592	14824	17383	17234
**Independent reflections**	3820 [R_{int} = 0.0463, R_{sigma} = 0.0252]	2690 [R_{int} = 0.0405, R_{sigma} = 0.0234]	2850 [R_{int} = 0.1026, R_{sigma} = 0.0616]	3418 [R_{int} = 0.0529, R_{sigma} = 0.0393]	3955 [R_{int} = 0.0441, R_{sigma} = 0.0492]
**Data/restraints/parameters**	3820/0/149	2690/0/250	2850/0/242	3418/722/313	3955/2/154
**Goodness-of-fit on F^2**	1.043	1.168	1.071	1.081	1.076
**Final R indexes [I>=2σ (I)]**	R_1 = 0.0249, wR_2 = 0.0379	R_1 = 0.0177, wR_2 = 0.0409	R_1 = 0.0422, wR_2 = 0.0884	R_1 = 0.0297, wR_2 = 0.0541	R_1 = 0.0443, wR_2 = 0.0585
**Final R indexes [all data]**	R_1 = 0.0346, wR_2 = 0.0401	R_1 = 0.0182, wR_2 = 0.0412	R_1 = 0.0663, wR_2 = 0.1023	R_1 = 0.0380, wR_2 = 0.0572	R_1 = 0.0558, wR_2 = 0.0612
**Largest diff. peak/hole / e Å^{-3}**	0.29/-0.29	0.11/-0.11	0.26/-0.28	0.13/-0.13	0.25/-0.28
**Flack parameter**		0.01(5)	0.2(2)	-0.9(6)	-0.1(4)

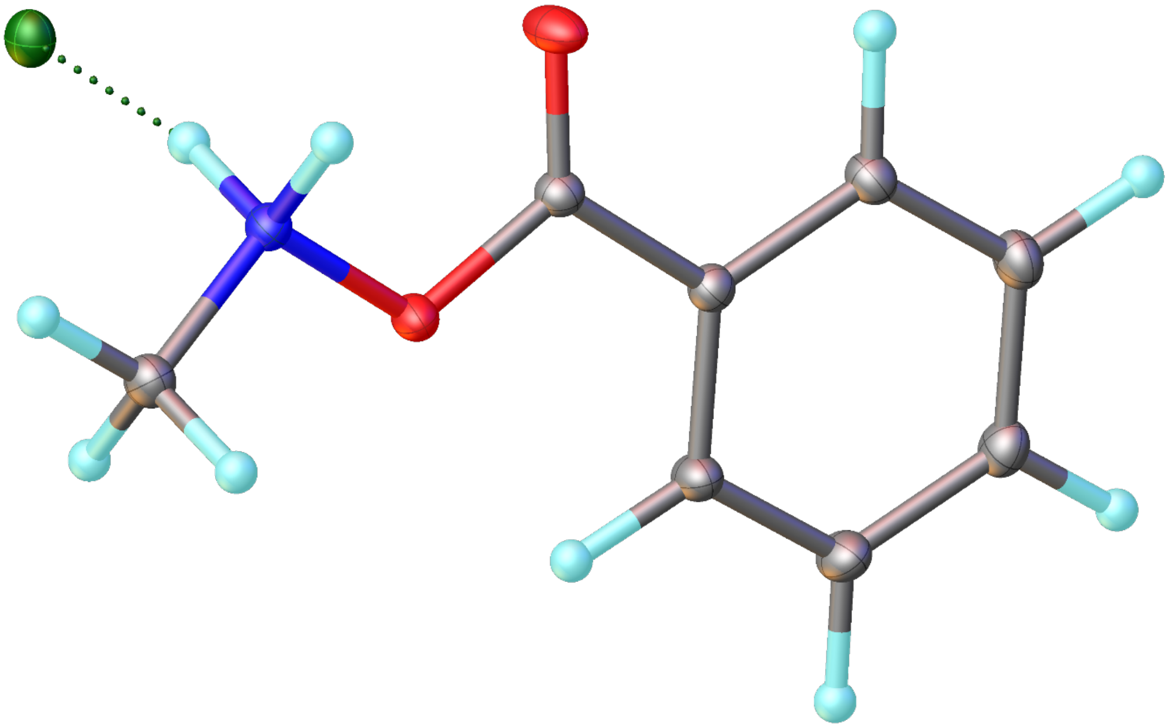

Figure S90; Molecular structure determined by SCXRD for compound **3a**, generated in Olex2 v1.5-beta with ellipsoids drawn at 50% probability.

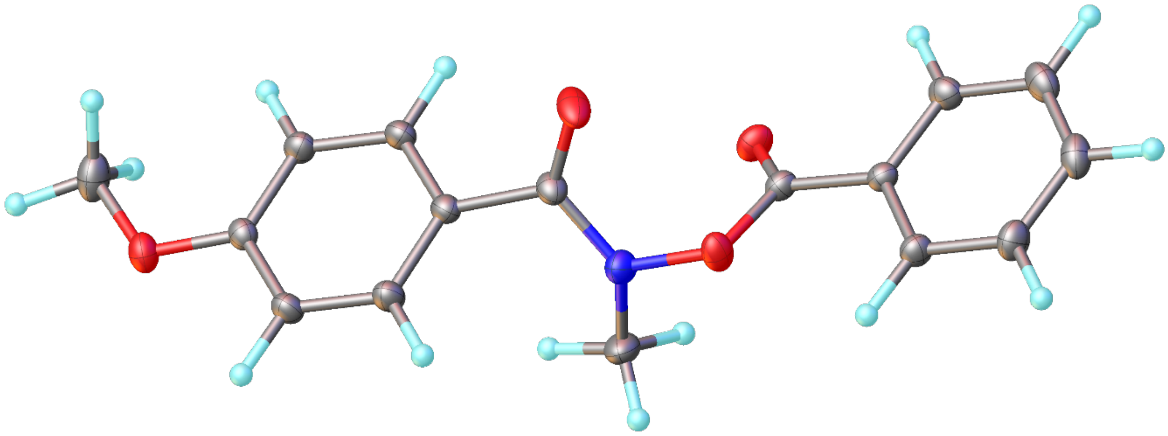

Figure S91; Molecular structure determined by SCXRD for compound **4h**, generated in Olex2 v1.5-beta with ellipsoids drawn at 50% probability.

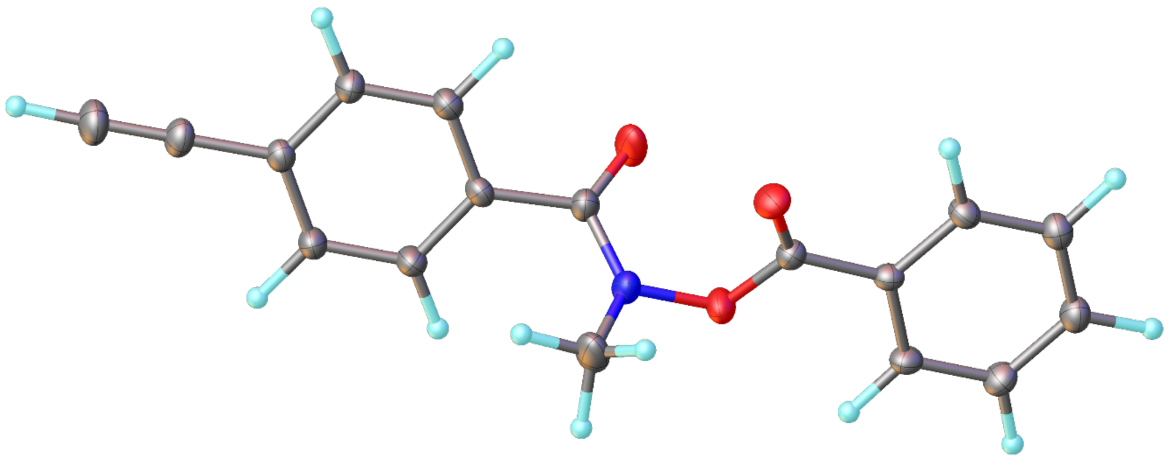

Figure S92; Molecular structure determined by SCXRD for compound **4j**, generated in Olex2 v1.5-beta with ellipsoids drawn at 50% probability.

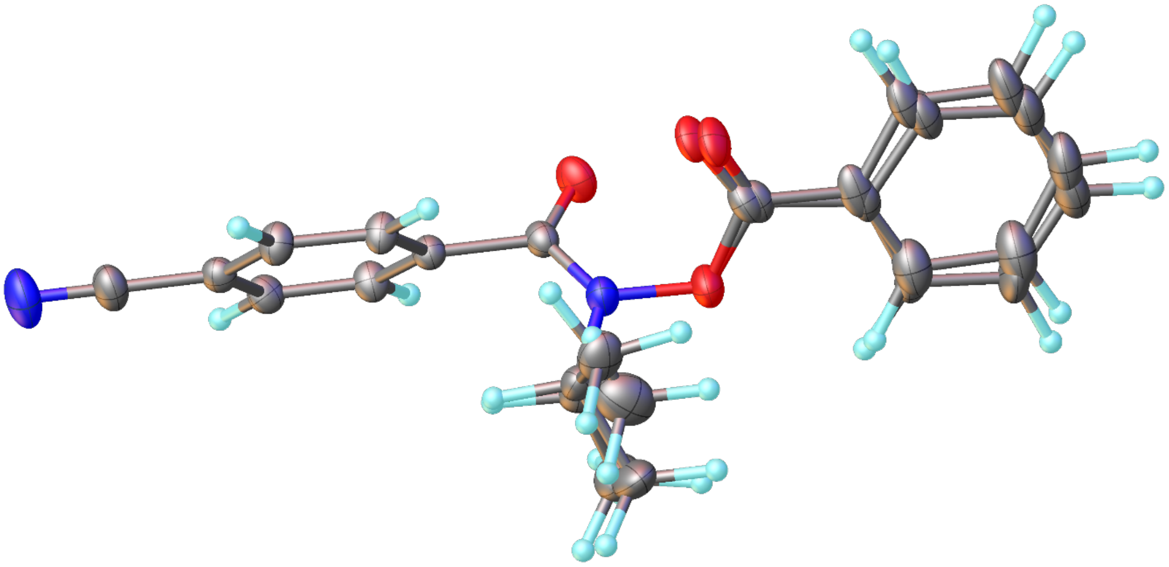

Figure S93; Molecular structure determined by SCXRD for compound **4r**, generated in Olex2 v1.5-beta with ellipsoids drawn at 50% probability.

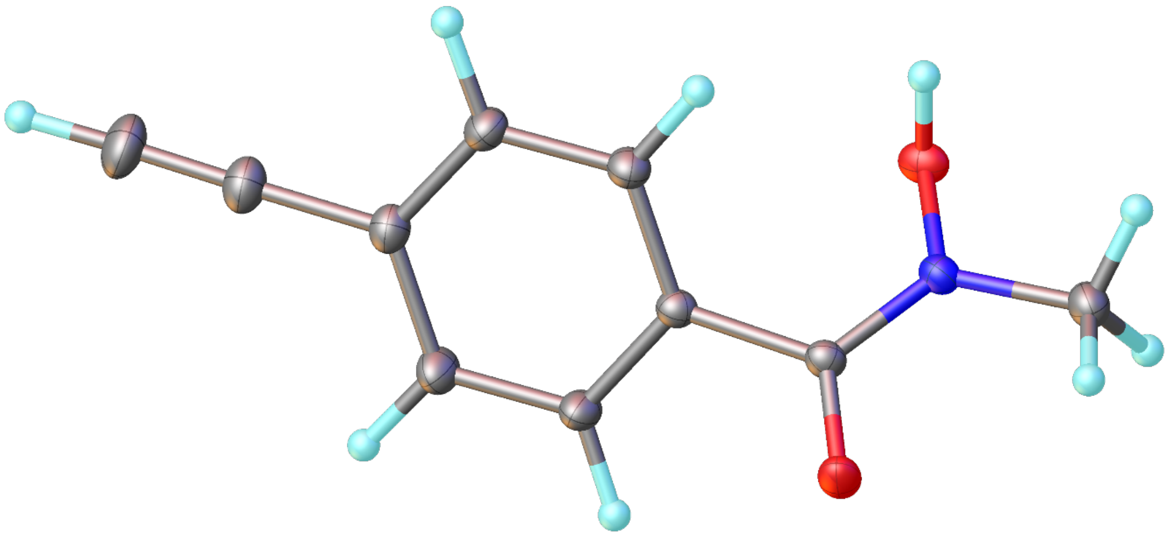

Figure S94; Molecular structure determined by SCXRD for compound **5h**, generated in Olex2 v1.5-beta with ellipsoids drawn at 50% probability.

References

[1] O. V. Dolomanov, L. J. Bourhis, R. J. Gildea, J. A. K. Howard, H. Puschmann, *J. Appl. Cryst.,* **2009**, *42*, 339-341.

[2] G. M. Sheldrick, *Acta Cryst.,* **2015**, *A71*, 3-8.

[3] L. J. Bourhis, O. V. Dolomanov, R. J. Gildea, J. A. K. Howard, H. Puschmann, *Acta Cryst.,* **2015**, *A71*, 59-75.

[4] F. Kleemiss, O. V. Dolomanov, M. Bodensteiner, N. Peyerimhoff, L. Midgley, L. J. Bourhis, A. Genoni, L. A. Malaspina, D. Jayatilaka, J. L. Spencer, F. White, B. Grundkötter-Stock, S. Steinhauer, D. Lentz, H. Puschmann, S. Grabowsky, *Chem. Sci.,* **2021**, *12*, 1675-1692.

[5] F. Neese, *Wiley Interdiscip. Rev.: Comput. Mol. Sci.,* **2025**, *15*, e70019.

[6] T. C. Jones, N. C. O. Tomkinson, *Org. Synth.,* **2007**, *84*, 233.

[7] C. S. Beshara, A. Hall, R. L. Jenkins, K. L. Jones, T. C. Jones, N. M. Killeen, P. H. Taylor, S. P. Thomas, N. C. O. Tomkinson, *Org. Lett.,* **2005**, *7*, 5729-5732.

[8] P. G. Mattingly, M. J. Miller, *J. Org. Chem.,* **1980**, *45*, 410-415.

[9] M. K. Gupta, Z. Li, T. S. Snowden, *Org. Lett.,* **2014**, *16*, 1602-1605.

[10] C. W. Muir, A. R. Kennedy, J. M. Redmond, A. J. B. Watson, *Org. Biomol. Chem.,* **2013**, *11*, 3337-3340.

[11] H. Wang, C. Zhou, Z. Gao, S. Li, G. Li, *Angew. Chem. Int. Ed.,* **2023**, *62*, e202300905.

[12] A. Bourboula, O. G. Mountanea, G. Krasakis, C. Mantzourani, M. G. Kokotou, C. G. Kokotos, G. Kokotos, *Eur. J. Org. Chem.,* **2023**, *26*, e202300008.

[13] A. Sanz-Marco, B. Saavedra, E. Erbing, J. Malmberg, M. J. Johansson, B. Martín-Matute, *Org. Lett.,* **2024**, *26*, 2800-2805.

[14] T. Niu, W. Zhang, D. Huang, C. Xu, H. Wang, Y. Hu, *Org. Lett.,* **2009**, *11*, 4474-4477.

[15] C. Yuan, B. Du, M.-M. Xun, B. Liu, *Tetrahedron,* **2017**, *73*, 3622-3628.

[16] A. W. Gann, J. W. Amoroso, V. J. Einck, W. P. Rice, J. J. Chambers, N. A. Schnarr, *Org. Lett.,* **2014**, *16*, 2003-2005.

[17] L. Ren, N. Jiao, *Chem. Commun.,* **2014**, *50*, 3706-3709.

[18] A. V. Bieliauskas, S. V. W. Weerasinghe, A. T. Negmeldin, M. K. H. Pflum, *Arch. Pharm. Chem. Life Sci.,* **2016**, *349*, 373-382.

[19] Y. Lu, D. Sun, D. Xiao, Y. Shao, M. Su, Y. Zhou, J. Li, S. Zhu, W. Lu, *Molecules,* **2021**, *26*, 7241.
